# Evidence of Gene–Environment Interactions between Common Breast Cancer Susceptibility Loci and Established Environmental Risk Factors

**DOI:** 10.1371/journal.pgen.1003284

**Published:** 2013-03-27

**Authors:** Stefan Nickels, Thérèse Truong, Rebecca Hein, Kristen Stevens, Katharina Buck, Sabine Behrens, Ursula Eilber, Martina Schmidt, Lothar Häberle, Alina Vrieling, Mia Gaudet, Jonine Figueroa, Nils Schoof, Amanda B. Spurdle, Anja Rudolph, Peter A. Fasching, John L. Hopper, Enes Makalic, Daniel F. Schmidt, Melissa C. Southey, Matthias W. Beckmann, Arif B. Ekici, Olivia Fletcher, Lorna Gibson, Isabel dos Santos Silva, Julian Peto, Manjeet K. Humphreys, Jean Wang, Emilie Cordina-Duverger, Florence Menegaux, Børge G. Nordestgaard, Stig E. Bojesen, Charlotte Lanng, Hoda Anton-Culver, Argyrios Ziogas, Leslie Bernstein, Christina A. Clarke, Hermann Brenner, Heiko Müller, Volker Arndt, Christa Stegmaier, Hiltrud Brauch, Thomas Brüning, Volker Harth, The GENICA Network, Arto Mannermaa, Vesa Kataja, Veli-Matti Kosma, Jaana M. Hartikainen, kConFab, AOCS Management Group, Diether Lambrechts, Dominiek Smeets, Patrick Neven, Robert Paridaens, Dieter Flesch-Janys, Nadia Obi, Shan Wang-Gohrke, Fergus J. Couch, Janet E. Olson, Celine M. Vachon, Graham G. Giles, Gianluca Severi, Laura Baglietto, Kenneth Offit, Esther M. John, Alexander Miron, Irene L. Andrulis, Julia A. Knight, Gord Glendon, Anna Marie Mulligan, Stephen J. Chanock, Jolanta Lissowska, Jianjun Liu, Angela Cox, Helen Cramp, Dan Connley, Sabapathy Balasubramanian, Alison M. Dunning, Mitul Shah, Amy Trentham-Dietz, Polly Newcomb, Linda Titus, Kathleen Egan, Elizabeth K. Cahoon, Preetha Rajaraman, Alice J. Sigurdson, Michele M. Doody, Pascal Guénel, Paul D. P. Pharoah, Marjanka K. Schmidt, Per Hall, Doug F. Easton, Montserrat Garcia-Closas, Roger L. Milne, Jenny Chang-Claude

**Affiliations:** 1 Division of Cancer Epidemiology, German Cancer Research Center (DKFZ), Heidelberg, Germany; 2 Inserm (National Institute of Health and Medical Research), CESP (Center for Research in Epidemiology and Population Health), U1018, Environmental Epidemiology of Cancer, Villejuif, France; 3 PMV Research Group at the Department of Child and Adolescent Psychiatry and Psychotherapy, University of Cologne, Cologne, Germany; 4 Department of Health Sciences Research, Mayo Clinic, Rochester, Minnesota, United States of America; 5 Department of Preventive Oncology, National Center of Tumor Diseases, Heidelberg, Germany; 6 Unit of Environmental Epidemiology, German Cancer Research Center (DKFZ), Heidelberg, Germany; 7 Department of Gynecology and Obstetrics, University Hospital, Friedrich-Alexander University Erlangen-Nuremberg, Erlangen, Germany; 8 Department for Health Evidence, Radboud University Medical Centre, Nijmegen, The Netherlands; 9 Epidemiology Research Program, Division of Cancer Epidemiology, American Cancer Society, Atlanta, Georgia, United States of America; 10 Division of Cancer Epidemiology and Genetics, National Cancer Institute, Rockville, Maryland, United States of America; 11 Department of Medical Epidemiology and Biostatistics, Karolinska Institutet, Stockholm, Sweden; 12 Queensland Institute of Medical Research, Herston, Queensland, Australia; 13 Department of Medicine, David Geffen School of Medicine, University of California Los Angeles, Los Angeles, California, United States of America; 14 Centre for Molecular, Environmental, Genetic and Analytic Epidemiology, University of Melbourne, Melbourne, Australia; 15 Department of Pathology, University of Melbourne, Melbourne, Australia; 16 Institute of Human Genetics, Friedrich Alexander University Erlangen-Nuremberg, Erlangen, Germany; 17 Breakthrough Breast Cancer Research Centre, Institute of Cancer Research, London, United Kingdom; 18 London School of Hygiene and Tropical Medicine, London, United Kingdom; 19 Centre for Cancer Genetic Epidemiology, Department of Public Health and Primary Care, University of Cambridge, Cambridge, United Kingdom; 20 Copenhagen General Population Study and Department of Clinical Biochemistry, Herlev University Hospital, University of Copenhagen, Copenhagen, Denmark; 21 Department of Breast Surgery, Herlev University Hospital, University of Copenhagen, Copenhagen, Denmark; 22 Department of Epidemiology, University of California Irvine, Irvine, California, United States of America; 23 Beckman Research Institute of the City of Hope, Duarte, California, United States of America; 24 Cancer Prevention Institute of California, Fremont, California, United States of America; 25 Division of Epidemiology, Department of Health Research and Policy, Stanford University School of Medicine, Stanford, California, United States of America; 26 Division of Clinical Epidemiology and Ageing Research, German Cancer Research Center (DKFZ), Heidelberg, Germany; 27 Saarland Cancer Registry, Saarbrücken, Germany; 28 Dr. Margarete Fischer-Bosch-Institute of Clinical Pharmacology, Stuttgart, Germany; 29 University of Tübingen, Tübingen, Germany; 30 Institute for Prevention and Occupational Medicine of the German Social Accident Insurance, Institute of the Ruhr-Universität Bochum (IPA), Bochum, Germany; 31 Institute and Outpatient Clinic of Occupational Medicine, Saarland University Medical Center and Saarland University Faculty of Medicine, Homburg, Germany; 32 Institute for Occupational Medicine and Maritime Medicine, University Medical Center Hamburg-Eppendorf, Hamburg, Germany; 33 Department of Internal Medicine, Evangelische Kliniken Bonn gGmbH, Johanniter Krankenhaus, Bonn, Germany; 34 Institute of Pathology, University of Bonn, Bonn, Germany; 35 Molecular Genetics of Breast Cancer, German Cancer Research Center (DKFZ), Heidelberg, Germany; 36 School of Medicine, Institute of Clinical Medicine, Department of Pathology and Forensic Medicine, University of Eastern Finland, Kuopio, Finland; 37 Biocenter Kuopio, Cancer Center of Eastern Finland, University of Eastern Finland, Kuopio, Finland; 38 School of Medicine, Institute of Clinical Medicine, Department of Oncology, University of Eastern Finland, Kuopio, Finland; 39 The Kathleen Cuningham Foundation for Resesarch into Familial Breast Cancer (kConFab), Peter MacCallum Cancer Centre, East Melbourne, Australia; 40 Vesalius Research Center (VRC), VIB, Leuven, Belgium; 41 Multidisciplinary Breast Center, University Hospital Gasthuisberg, Leuven, Belgium; 42 Department of Cancer Epidemiology/Clinical Cancer Registry and Institute for Medical Biometrics and Epidemiology, University Clinic Hamburg-Eppendorf, Hamburg, Germany; 43 Department of Obstetrics and Gynecology, University of Ulm, Ulm, Germany; 44 Department of Laboratory Medicine and Pathology, Mayo Clinic, Rochester, Minnesota, United States of America; 45 Cancer Epidemiology Centre, The Cancer Council Victoria, Melbourne, Australia; 46 Centre for Molecular, Environmental, Genetic, and Analytic Epidemiology, University of Melbourne, Australia; 47 Memorial Sloan-Kettering Cancer Center, New York, New York, United States of America; 48 Dana-Farber Cancer Institute, Boston, Massachusetts, United States of America; 49 Ontario Cancer Genetics Network, Fred A. Litwin Center for Cancer Genetics, Samuel Lunenfeld Research Institute, Mount Sinai Hospital, Toronto, Canada; 50 Department of Molecular Genetics, University of Toronto, Toronto, Canada; 51 Samuel Lunenfeld Research Institute, Mount Sinai Hospital, Toronto, Canada; 52 Division of Epidemiology, Dalla Lana School of Public Health, University of Toronto, Toronto, Canada; 53 Ontario Cancer Genetics Network, Samuel Lunenfeld Research Institute, Mount Sinai Hospital, Toronto, Canada; 54 Laboratory Medicine Program, University Health Network, Toronto, Canada; 55 Department of Laboratory Medicine and Pathobiology, University of Toronto, Toronto, Canada; 56 Division of Cancer Epidemiology and Genetics, National Cancer Institute, Rockville, Maryland, United States of America; 57 Department of Cancer Epidemiology and Prevention, M. Sklodowska-Curie Memorial Cancer Center and Institute of Oncology, Warsaw, Poland; 58 Human Genetics, Genome Institute of Singapore, Singapore, Singapore; 59 Institute for Cancer Studies, Department of Oncology, University of Sheffield, Sheffield, United Kingdom; 60 Academic Unit of Surgical Oncology, Department of Oncology, University of Sheffield, Sheffield, United Kingdom; 61 Department of Oncology, University of Cambridge, Cambridge, United Kingdom; 62 University of Wisconsin Carbone Cancer Center, Madison, Wisconsin, United States of America; 63 Fred Hutchinson Cancer Research Center, Seattle, Washington, United States of America; 64 Department of Community and Family Medicine, Department of Pediatrics, Dartmouth Medical School, Dartmouth-Hitchcock Medical Center, Lebanon, New Hampshire, United States of America; 65 Division of Population Sciences, Moffitt Cancer Center and Research Institute, Tampa, Florida, United States of America; 66 Radiation Epidemiology Branch, Division of Cancer Epidemiology and Genetics, National Cancer Institute, Rockville, Maryland, United States of America; 67 Department of Oncology and Department of Public Health and Primary Care, University of Cambridge, Cambridge, United Kingdom; 68 Division of Molecular Pathology and Division of Psychosocial Research and Epidemiology, Netherlands Cancer Institute, Amsterdam, The Netherlands; 69 Sections of Epidemiology and Genetics, Institute of Cancer Research and Breakthrough Breast Cancer Research Centre, London, United Kingdom; 70 Genetic and Molecular Epidemiology Group, Human Cancer Genetics Programme, Spanish National Cancer Research Centre (CNIO), Madrid, Spain; University of Washington, United States of America

## Abstract

Various common genetic susceptibility loci have been identified for breast cancer; however, it is unclear how they combine with lifestyle/environmental risk factors to influence risk. We undertook an international collaborative study to assess gene-environment interaction for risk of breast cancer. Data from 24 studies of the Breast Cancer Association Consortium were pooled. Using up to 34,793 invasive breast cancers and 41,099 controls, we examined whether the relative risks associated with 23 single nucleotide polymorphisms were modified by 10 established environmental risk factors (age at menarche, parity, breastfeeding, body mass index, height, oral contraceptive use, menopausal hormone therapy use, alcohol consumption, cigarette smoking, physical activity) in women of European ancestry. We used logistic regression models stratified by study and adjusted for age and performed likelihood ratio tests to assess gene–environment interactions. All statistical tests were two-sided. We replicated previously reported potential interactions between *LSP1-*rs3817198 and parity (P_interaction_ = 2.4×10^−6^) and between *CASP8*-rs17468277 and alcohol consumption (P_interaction_ = 3.1×10^−4^). Overall, the per-allele odds ratio (95% confidence interval) for *LSP1*-rs3817198 was 1.08 (1.01–1.16) in nulliparous women and ranged from 1.03 (0.96–1.10) in parous women with one birth to 1.26 (1.16–1.37) in women with at least four births. For *CASP8*-rs17468277, the per-allele OR was 0.91 (0.85–0.98) in those with an alcohol intake of <20 g/day and 1.45 (1.14–1.85) in those who drank ≥20 g/day. Additionally, interaction was found between 1p11.2-rs11249433 and ever being parous (P_interaction_ = 5.3×10^−5^), with a per-allele OR of 1.14 (1.11–1.17) in parous women and 0.98 (0.92–1.05) in nulliparous women. These data provide first strong evidence that the risk of breast cancer associated with some common genetic variants may vary with environmental risk factors.

## Author Summary

Breast cancer involves combined effects of numerous genetic, environmental, and behavioral risk factors that are unique to each individual. High risk genes, such as *BRCA1* and *BRCA2*, account for only a small proportion of disease occurrence. Recent genome-wide research has identified more than 20 common genetic variants, which individually alter breast cancer risk very moderately. We undertook an international collaborative study to determine whether the effect of these genetic variants vary with environmental factors, such as parity, body mass index (BMI), height, oral contraceptive use, menopausal hormone therapy use, alcohol consumption, cigarette smoking, and physical activity, which are known to affect risk of developing breast cancer. Using pooled data from 24 studies of the Breast Cancer Association Consortium (BCAC), we provide first convincing evidence that the breast cancer risk associated with a genetic variant in *LSP1* differs with the number of births and that the risk associated with a *CASP8* variant is altered by high alcohol consumption. The effect of an additional genetic variant might also be modified by reproductive factors. This knowledge will stimulate new research towards a better understanding of breast cancer development.

## Introduction

Both genetic and non-genetic factors are involved in the etiology of breast cancer. Known susceptibility variants include rare high-risk mutations, principally in *BRCA1* and *BRCA2*, more moderate susceptibility variants in genes such as *PALB2*, *CHEK2* and *ATM*, and more than 20 common genetic susceptibility variants conferring modest increased risks, principally identified through genome-wide association studies. Taken together, the known susceptibility variants have been estimated to explain about 20–25% of the observed familial breast cancer risk [1]. There is still limited knowledge about how the relative risks of common susceptibility loci might be modified by the established reproductive and lifestyle risk factors (referred to as environmental risk factors) for breast cancer. Such knowledge could provide insights into common biological pathways for cancer development and further our understanding of breast cancer etiology for specific tumor subtypes. Previous reports of a possible interaction between variants in *FGFR2* and use of menopausal hormone therapy (MHT) were not confirmed [2]–[6]. All recent large studies found no statistically significant evidence of multiplicative gene-environment interaction between several common susceptibility loci and established risk factors for breast cancer after allowing for multiple comparisons [2], [6], [7]. The strongest previously reported findings were for an interaction between *LSP1*-rs3817198 and number of births (P-value = 0.002), between *CASP8*-rs104585 and alcohol consumption (P-value = 0.003), and between 5p12-rs10941679 and use of estrogen-only MHT (P-value = 0.007) [2], [6], [7]. This lack of statistical evidence of interaction beyond that expected by chance may be partly due to limited power to detect weak gene-environment interactions and not having considered specific subtypes of breast cancer. We used pooled data from 24 studies participating in the Breast Cancer Association Consortium (BCAC) to evaluate whether the relative risks of single nucleotide polymorphisms (SNPs) at 23 published loci vary according to levels of 10 established environmental risk factors [8]. Since there is etiologic heterogeneity by subtypes of breast cancer, we also carried out these assessments for breast cancer with positive and negative estrogen receptor (ER) status [9].

## Results

Up to 34,793 invasive cases and 41,099 controls of self-reported European ancestry were included in these analyses (Table 1). Based on 18,532 cases and 25,341 controls from 16 population-based studies, we found the expected associations between the environmental risk factors and breast cancer risk (Table 2). As expected, significant effect heterogeneity by age (as a surrogate for menopausal status) was observed only for body mass index (BMI) (P-value = 0.007).

**Table 1:** List of participating studies and number of Caucasian subjects included in at least one GxE analysis.

Study acronym	Study Name	Country	Design category	Cases/controls used for GxE	ER+ cases	ER−cases	Mean age (range) in cases	Mean age (range) in controls
ABCFS	Australian Breast Cancer Family Study	Australia	Population-based^1^	1335/687	754	392	42.4 (23–69)	41.6 (20–68)
CECILE	CECILE Breast Cancer Study	France	Population-based	938/1026	768	143	54.4 (25–74)	54.7 (25–74)
CGPS	Copenhagen General Population Study	Denmark	Population-based	2388/6704	1800	357	62.0 (27–95)	55.7 (20–91)
CTS	California Teachers Study	USA	Prospective cohort^1^	1252/1226	No Info	No Info	61.8 (32–83)	56.2 (26–77)
ESTHER	ESTHER Breast Cancer Study	Germany	Population-based	433/511	293	85	60.3 (30–79)	62.3 (49–75)
GENICA	Gene Environment Interaction and Breast Cancer in Germany	Germany	Population-based	1021/1015	755	216	58.2 (23–80)	58.2 (24–80)
GESBC	Genetic Epidemiology Study of Breast Cancer by Age 50	Germany	Population-based	586/869	248	155	42.6 (20–50)	42.7 (24–52)
KBCP	Kuopio Breast Cancer Project	Finland	Population-based	466/523	328	98	59.0 (23–92)	52.9 (17–77)
MARIE	Mammary Carcinoma Risk Factor Investigation	Germany	Population-based	2583/5309	2008	533	62.5 (50–75)	61.9 (49–75)
MCCS	Melbourne Collaborative Cohort Study	Australia	Prospective cohort	703/766	424	141	61.4 (37–80)	57.2 (38–70)
NC-BCFR	Northern California Breast Cancer Family Registry	USA	Population-based	268/154	203	35	56.9 (26–65)	56.9 (51–65)
OFBCR	Ontario Familial Breast Cancer Registry	Canada	Population-based	1135/328	634	260	53.8 (22–81)	57.4 (40–69)
PBCS	NCI Polish Breast Cancer Study	Poland	Population-based	2009/2381	1204	622	55.7 (27–75)	55.7 (24–75)
SASBAC	Singapore and Sweden Breast Cancer Study	Sweden	Population-based	1246/1515	711	160	63.0 (50–75)	63.4 (49–76)
US3SS	US Three State Study	USA	Population-based	1444/1274	No Info	No Info	54.3 (29–69)	54.3 (27–75)
USRT	US Radiologic Technologists Study	USA	Population-based	725/1053	No Info	No info	48.9 (22–82)	62.8 (42–94)
BBCC	Bavarian Breast Cancer Cases and Controls	Germany	Mixed^2^	1432/1002	967	375	55.4 (22–96)	57.2 (18–100)
BBCS	British Breast Cancer Study	UK	Mixed	1381/1297	No Info	No Info	53.9 (25–77)	51.4 (21–81)
kConFab/AOCS	Kathleen Cuningham Foundation Consortium for research into Familial Breast Cancer/Australian Ovarian Cancer Study	Australia/New Zealand	Mixed	499/962	156	65	45.0 (20–76)	58.3 (20–83)
LMBC	Leuven Multidisciplinary Breast Centre	Belgium	Mixed	2890/1625	2290	416	56.6 (21–94)	44.1 (19–66)
MCBCS	Mayo Clinic Breast Cancer Study	USA	Mixed	1803/2452	1475	292	56.8 (22–93)	56.6 (19–91)
MSKCC	Memorial Sloan-Kettering Cancer Center Study	USA	Hospital-based^3^	425/455	256	66	47.1 (23–85)	47.0 (24–86)
SBCS	Sheffield Breast Cancer Study	UK	Mixed	1111/1283	533	175	59.0 (28–92)	57.7 (45–80)
SEARCH	Study of Epidemiology and Risk factors in Cancer Heredity	UK	Mixed	6720/6682	3758	977	53.3 (23–88)	58.4 (26–81)
Total				34793/41099	19565	5563		

1 Studies that included all, or a random sample of all cases occurring in a geographically defined population during a specified period of time, and controls that were a random sample of the same source population as cases, recruited during the same period of time.

2 Studies not strictly population-based or hospital-based.

3 Cases diagnosed in a given hospital or hospitals during a specified period of time, and controls that were selected from the recruitment area as the cases during the same period of time.

**Table 2:** Main effects for the epidemiologic variables included in the analyses, derived from population-based studies only^1^.

	All			<54 years			> = 54 years			
Variable	n (cases/controls)	OR (95% CI)	p-value	n (ca/co)	OR (95% CI)	p-value	n (ca/co)	OR (95% CI)	p-value	Studies included
Age at menarche (per 2 years)	17185/24136	0.93 (0.90–0.95)	7.8×10^−9^	6511/8987	0.90 (0.86–0.94)	1.0×10^−5^	10674/15149	0.93 (0.90–0.96)	3.3×10^−6^	ABCFS CECILE CGPS CTS ESTHER GENICA GESBC KBCP MARIE MCCS NC-BCFR OFBCR PBCS SASBAC US3SS USRTS
Parous (yes/no)	18265/25241	0.80 (0.76–0.85)	3.9×10^−15^	6807/9128	0.85 (0.78–0.93)	0.00051	11458/16113	0.77 (0.71–0.82)	3.7×10^−13^	ABCFS CECILE CGPS CTS ESTHER GENICA GESBC KBCP MARIE MCCS NC-BCFR OFBCR PBCS SASBAC US3SS USRTS
Number of births (among parous)	15046/21771	0.90 (0.88–0.92)	7.9×10^−24^	5397/7635	0.92 (0.89–0.96)	0.00023	9649/14136	0.89 (0.87–0.91)	6.5×10^−21^	ABCFS CECILE CGPS CTS ESTHER GENICA GESBC KBCP MARIE MCCS NC-BCFR OFBCR PBCS SASBAC US3SS USRTS
Age at first birth (per 5 years)	14671/21322	1.08 (1.06–1.11)	4.6×10^−11^	5327/7550	1.06 (1.02–1.11)	0.0031	9344/13772	1.10 (1.07–1.14)	3.4×10^−10^	ABCFS CECILE CGPS CTS GENICA GESBC KBCP MARIE MCCS NC-BCFR OFBCR PBCS SASBAC US3SS USRTS
Ever breastfed (yes/no)	11022/13182	0.90 (0.85–0.96)	0.0013	4174/4267	0.87 (0.79–0.97)	0.011	6848/8915	0.90 (0.83–0.97)	0.0073	ABCFS CECILE GENICA GESBC KBCP MARIE MCCS NC-BCFR OFBCR PBCS SASBAC US3SS
Usual adult BMI (per 5 units)	-	-	-	5051/4905	0.92 (0.88–0.97)	0.0010	7557/9832	1.01 (0.97–1.05)	0.550	<54: ABCFS CECILE GENICA GESBC KBCP MARIE NC-BCFR OFBCR PBCS SASBAC US3SS/> = 54: ABCFS CECILE GENICA KBCP MARIE NC-BCFR OFBCR PBCS SASBAC US3SS
Usual adult height (per 5 cm)	15861/18464	1.07 (1.05–1.09)	4.1×10^−13^	6096/5990	1.05 (1.02–1.08)	0.0017	9765/12474	1.08 (1.06–1.11)	3.4×10^−12^	ABCFS CECILE CTS ESTHER GENICA GESBC KBCP MARIE MCCS NC-BCFR OFBCR PBCS SASBAC US3SS USRTS
Ever use of oral contraceptives(yes/no)	12812/15667	0.99 (0.93–1.05)	0.687	4762/4961	1.01 (0.91–1.13)	0.831	8050/10706	0.99 (0.92–1.06)	0.688	ABCFS CECILE ESTHER GENICA GESBC KBCP MARIE MCCS NC-BCFR PBCS SASBAC US3SS
Duration of oral contraceptive use (per 5 years)	12671/15478	1.02 (1.00–1.04)	0.021	4714/4914	1.05	(1.01–1.08)	0.0067	7957/10564	1.01 (0.99–1.04) 0.336	ABCFS CECILE ESTHER GENICA GESBC KBCP MARIE MCCS NC-BCFR PBCS SASBAC US3SS
Current use of combined estrogen-progestagen therapy	-	-	-	-	-	-	6425/9225	1.76(1.61–1.94)	6.9×10^−33^	CECILE GENICA MARIE PBCS SASBAC US3SS
Current use of estrogen-only therapy	-	-	-	-	-	-	6689/9457	1.19 (1.07–1.33)	0.001	CECILE GENICA MARIE PBCS SASBAC US3SS
Duration of combined estrogen-progestagen therapy in current users (per 5 years)	-	-	-	-	-	-	6337/9130	1.25 (1.20–1.30)	9.6×10^−27^	CECILE GENICA MARIE PBCS SASBAC US3SS
Duration of estrogen-only therapy in current users (per 5 years)	-	-	-	-	-	-	6596/9332	1.07 (1.03–1.12)	9.8×10^−4^	CECILE GENICA MARIE PBCS SASBAC US3SS
Lifetime intake of alcohol^2^ (per 10 g/day)	6763/10273	1.03 (1.00–1.05)	0.035	2280/3162	1.05 (1.00–1.09)	0.0443	4483/7111	1.02 (0.99–1.05)	0.167	CECILE GESBC MARIE MCCS PBCS
Smoking (ever/never)	13725/16189	1.02 (0.98–1.07)	0.344	5292/5284	1.05 (0.97–1.14)	0.237	8433/10905	1.02 (0.96–1.08)	0.571	ABCFS CECILE ESTHER GENICA GESBC KBCP MARIE MCCS OFBCR PBCS SASBAC US3SS
Smoking amount(per 10 pack-years)	11890/14044	1.01 (0.99–1.03)	0.447	5030/5045	1.04 (1.00–1.08)	0.032	6860/8999	1.00 (0.98–1.03)	0.837	ABCFS CECILE GENICA GESBC KBCP MARIE MCCS OFBCR PBCS US3SS
Physical activity in year before reference date (square root of h/week)^3^	7211/1052	0.92 (0.87–0.97)	0.005	1759/1996	0.96 (0.89–1.02)	0.189	5452/8056	0.96 (0.93–1.00)	0.032	CECILE GENICA MARIE SASBAC US3SS

1 Model used for the assessment of epidemiologic main effects: logit(Pr(breast cancer|risk factor)) = β_0_+β_1_*study + β_2_*reference_age + β_3_*risk_factor.

2 Mean lifetime alcohol intake derived from duration and amount of alcohol intake in g/day at different age periods.

3 For physical activity, square root (hours/week) was used since this model gave the highest likelihood when modeling the marginal association using fractional polynomials (Royston P, Ambler G, Sauerbrei W. The use of fractional polynomials to model continuous risk variables in epidemiology. Int J Epidemiol 1999;28(5):964-74.) and was further adjusted for menopausal status.

Except for *TGFB1*-rs1982073, all SNPs showed highly significant associations with breast cancer overall (Table 3). Eleven SNPs showed evidence of heterogeneity in the OR by ER status at p<0.01. The per-allele OR overall and for subsets of women with information available for the risk factors considered were very similar to those previously published and provided no evidence of bias in OR estimates related to data availability (data not shown).

**Table 3:** Associations between selected SNPs and breast cancer risk in Caucasians, overall and by ER status (estimated per-allele odds ratios and 95% confidence intervals)^1^.

SNP	Locus	Gene	Allele	MAF^5^	N Cases/Controls	OR per allele (95%CI)	P trend	P het ER status^6^	ER+n (ca)	ER+OR (95%CI)	P trend	ER-n (ca)	ER-OR (95%CI)	P trend
rs11249433	1p11	-	T/C	0.401	29502/31361	1.11 (1.09–1.14)	5.5×10^−19^	3.6×10^−5^	17670	1.13 (1.10–1.16)	7.8×10^−18^	5030	1.03 (0.98–1.07)	0.223
rs17468277^2^	2q33	*CASP8*	C/T	0.127	29884/35245	0.94 (0.91–0.97)	0.00022	0.019	17589	0.97 (0.93–1.01)	0.090	4956	0.88 (0.83–0.94)	0.0025
rs13387042	2q35	*-*	A/G	0.484	29732/34911	0.88 (0.86–0.90)	1.3×10^−26^	0.00030	17859	0.87 (0.85–0.90)	1.8×10^−23^	5085	0.94 (0.90–0.98)	0.0053
rs4973768	3p24	*SLC4A7*	C/T	0.466	29300/33940	1.10 (1.08–1.13)	5.8×10^−17^	0.005	17643	1.11 (1.08–1.14)	5.7×10^−15^	5037	1.04 (1.00–1.09)	0.057
rs10941679	5p12	*-*	A/G	0.256	29511/34613	1.12 (1.09–1.15)	1.3×10^−18^	8.8×10^−5^	17688	1.14 (1.11–1.18)	7.2×10^−18^	5110	1.03 (0.98–1.08)	0.288
rs889312	5q11	*MAP3K1*	A/C	0.278	28387/29030	1.11 (1.08–1.14)	4.1×10^−15^	0.038	16446	1.12 (1.09–1.16)	1.7×10^−13^	4740	1.06 (1.01–1.11)	0.025
rs12662670	6q25	*ESR1*	T/G	0.076	16518/15659	1.16 (1.09–1.23)	4.8×10^−7^	0.073	10810	1.12 (1.05–1.20)	0.00061	2705	1.22 (1.10–1.35)	0.00023
rs2046210	6q25	*ESR1*	C/T	0.341	28196/29938	1.09 (1.06–1.12)	1.4×10^−11^	6.4×10^−7^	16713	1.06 (1.03–1.09)	6.42×10^−5^	4667	1.21 (1.16–1.27)	1.2×10^−15^
rs13281615	8q24	*-*	A/G	0.406	27252/26610	1.13 (1.10–1.16)	7.5×10^−23^	0.100	16067	1.14 (1.11–1.17)	1.2×10^−18^	4635	1.08 (1.03–1.13)	0.016
rs1011970	9p21	*CDKN2A/B*	G/T	0.162	23531/28641	1.09 (1.05–1.12)	2.2×10^−6^	0.073	14565	1.07 (1.03–1.11)	0.00010	4141	1.13 (1.06–1.21)	9.5×10^−5^
rs865686	9q31	*-*	T/G	0.381	28077/31963	0.90 (0.88–0.92)	1.2×10^−17^	6.1×10^−6^	17037	0.88 (0.86–0.91)	4.9×10^−17^	4505	0.99 (0.94–1.03)	0.541
rs10995190	10q21	*ZNF365*	G/A	0.159	22672/28655	0.88 (0.85–0.91)	1.6×10^−12^	0.218	13876	0.88 (0.84–0.91)	7.5×10^−10^	4028	0.91 (0.85–0.98)	0.0081
rs704010	10q22	*ZMIZ1*	G/A	0.383	23456/28651	1.06 (1.03–1.09)	2.4×10^−5^	0.150	14528	1.05 (1.02–1.09)	0.00079	4132	1.02 (0.97–1.07)	0.468
rs2981582	10q26	*FGFR2*	C/T	0.383	31807/33940	1.23 (1.20–1.26)	7.2×10^−73^	2.0×10^−18^	17973	1.28 (1.25–1.32)	2.1×10^−70^	5141	1.04 (1.00–1.09	0.053
rs614367	11q13	*-*	C/T	0.152	21068/22008	1.21 (1.16–1.25)	4.8×10^−23^	1.4×10^−9^	12749	1.26 (1.21–1.32)	8.0×10^−26^	3777	1.02 (0.96–1.10)	0.509
rs3817198	11p15	*LSP1*	T/C	0.312	28404/28438	1.09 (1.06–1.12)	5.6×10^−11^	0.543	16395	1.08 (1.04–1.11)	3.1×10^−6^	4743	1.07 (1.02–1.12)	0.0076
rs10771399^3^	12p11	*PTHLH*	T/C	0.117	21182/18129	0.84 (0.80–0.88)	1.4×10^−12^	0.590	14392	0.86 (0.82–0.91)	3.3×10^−8^	3455	0.82 (0.75–0.90)	3.08×10^−5^
rs1292011	12q24	*-*	T/C	0.415	17780/14298	0.94 (0.91–0.97)	0.00026	0.0056	12424	0.92 (0.89–0.96)	2.6×10^−5^	2935	1.00 (0.94–1.06)	0.887
rs999737^4^	14q24	*RAD51L1*	T/A	0.230	29189/31066	0.93 (0.91–0.96)	1.3×10^−6^	0.475	17493	0.93 (0.90–0.96)	1.8×10^−5^	4985	0.95 (0.90–1.00)	0.062
rs3803662	16q12	*TOX3*	C/T	0.262	27700/29192	1.24 (1.21–1.27)	8.3×10^−58^	0.0036	15802	1.26 (1.22–1.30)	1.0×10^−45^	4659	1.17 (1.12–1.23)	3.6×10^−10^
rs6504950	17q23	*COX11*	G/A	0.276	29787/34101	0.93 (0.91–0.96)	2.2×10^−7^	0.00057	18028	0.92 (0.89–0.95)	1.3×10^−7^	5100	1.01 (0.96–1.06)	0.791
rs1982073	19q13	*TGFB1*	T/C	0.376	17012/22985	1.04 (1.01–1.07)	0.020	0.314	9889	1.03 (1.00–1.07)	0.082	3032	1.07 (1.01–1.13)	0.018
rs2823093	21q21	*NRIP1*	G/A	0.267	18655/16443	0.95 (0.92–0.98)	0.0038	0.121	12927	0.94 (0.91–0.98)	0.0031	2972	1.00 (0.93–1.06)	0.898

1 model used for the assessment of SNP main effects: logit(Pr(breast cancer|SNP)) = β_0_+β_1_*study + β_2_*SNP.

2 or the highly correlated SNP rs1045485 (*r^2^* = 1 in HapMap CEU).

3 or the highly correlated SNP rs1975930 (*r^2^* = 1 in HapMap CEU).

4 or the highly correlated SNP rs10483813 (*r^2^* = 1 in HapMap CEU).

5 MAF: minor allele frequency among controls.

6 P-value for heterogeneity by ER-status: from case-case analysis.

**Figure 1:**
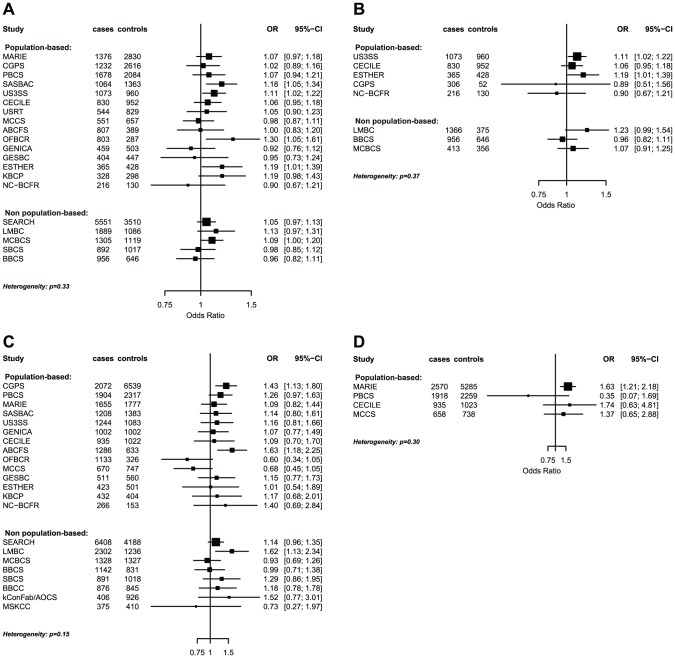
Odds ratios of gene-environment interaction for risk of breast cancer with p-value<10^−3^ by study. (A) *LSP1*-rs3817198 x Number of full-term births (among parous), (B) *LSP1*-rs3817198 x Number of full-term births (among parous), restricted to subjects not included in previous BCAC report, (C) 1p11-rs11249433 x Parous (yes/no), (D) *CASP8*-rs17468277 x mean lifetime intake of alcohol (<20 g/day versus > = 20 g/day).

**Figure 2:**
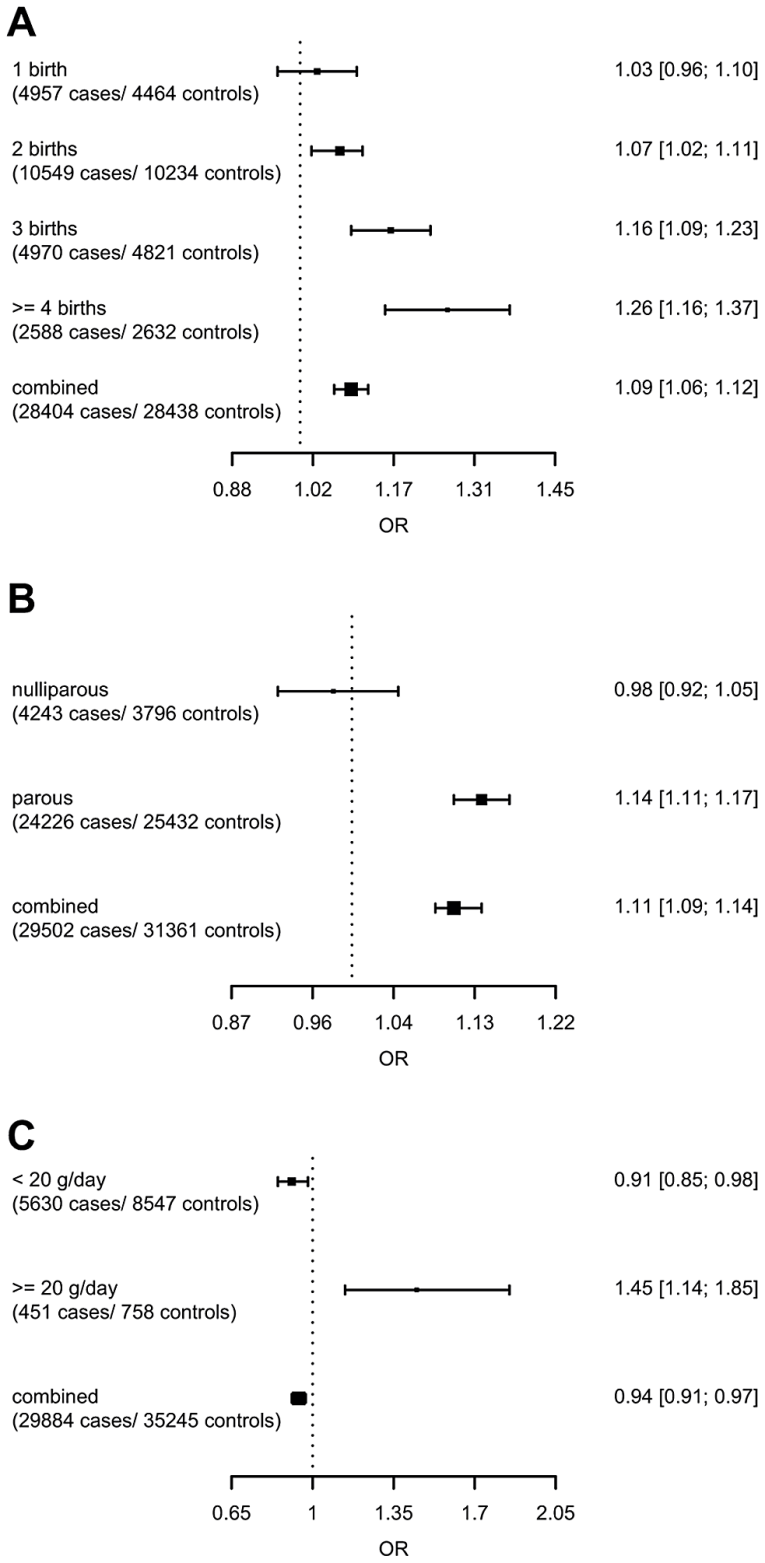
Per-allele SNP odds ratios and 95% confidence intervals stratified by environmental risk factors of breast cancer, and combined SNP main effect. (A) *LSP1*-rs3817198 x Number of full-term births (among parous), (B) 1p11-rs11249433 x Parous (yes/no), (C) *CASP8*-rs17468277 x mean lifetime intake of alcohol (<20 g/day versus > = 20 g/day).

The strongest evidence was found for modification of the association with *LSP1-*rs3817198 by number of births in parous women (P_interaction_ per birth increase in parous women = 2.4×10^−6^) (Table 4; Figure 1 showing individual study results). Since this interaction was previously assessed in BCAC, we reassessed the interaction in 6266 cases and 3899 controls not included in the previous report [7]. The SNP association still varied significantly with number of births in parous women (P_interaction_ = 1.6×10^−3^), thus independently replicating the previous finding. The results were consistent across studies (P_heterogeneity_ = 0.37) (Figure 1B). In the overall dataset, the per-allele OR (95% confidence interval) for rs3817198 ranged from 1.03 (0.96–1.10) in parous women with one birth to 1.26 (1.16–1.37) in women with four or more births (Figure 2) and in comparison was 1.08 (1.01–1.16) in nulliparous women (Table S4).

**Table 4:** Per-allele odds ratios and 95% confidence intervals for SNPs by environmental risk factors of breast cancer showing interaction P-value<10^−3^, overall and by estrogen receptor status.

			All				Estrogen receptor-positive			Estrogen receptor-negative		
SNP (Gene)	Variable	Category	N Cases/Controls	OR (95%CI)	P_interaction_ 1	P_het_ 2	N Cases	OR (95%CI)	P_interaction_ 1	N Cases	OR (95%CI)	P_interaction_ 1
rs3817198 (*LSP1*)	Number of births (among parous women)	1	4957/4464	1.03 (0.96–1.10)			2970	1.02 (0.95–1.10)		936	0.98 (0.87–1.10)	
		2	10549/10234	1.07 (1.02–1.11)			6044	1.05 (1.00–1.11)		1800	1.05 (0.97–1.14)	
		3	4970/4821	1.16 (1.09–1.23)			2871	1.15 (1.07–1.24)		780	1.13 (1.00–1.27)	
		> = 4	2588/2632	1.26 (1.16–1.37)	2.4×10^−6^	0.33	1453	1.26 (1.13–1.40)	5.6×10^−5^	416	1.26 (1.06–1.49)	5.7×10^−3^
rs11249433(1p11)	Parous	No	4243/3796	0.98 (0.92–1.05)			2543	0.97 (0.90–1.04)		720	0.96 (0.85–1.08)	
		Yes	24226/25432	1.14 (1.11–1.17)	5.3×10^−5^	0.15	14443	1.16 (1.13–1.20)	1.6×10^−5^	4203	1.04 (0.99–1.10)	0.19
rs17468277^3^(*CASP8*)	Mean lifetime intake of alcohol^4^ (g/day)	<20	5630/8547	0.91 (0.85–0.98)			3965	0.94 (0.87–1.02)		1315	0.88 (0.78–1.00)	
		> = 20	451/758	1.45 (1.14–1.85)	3.1×10^−4^	0.30	345	1.48 (1.14–1.91)	0.001	83	1.22 (0.77–1.94)	0.18

1 P-value for GxE interaction from logistic regression analysis stratified by study and adjusted for reference age. The interaction term was the product between the continuous SNP variable (number of risk alleles) and the risk factor variable (continuous for number of births and dichotomized for ever being parous and for mean alcohol intake): logit(Pr(breast cancer|risk factor, study, SNP)) = β_0_+β_1_* reference_age + β_2_*SNP + β_3_*risk_factor + β_4_*SNP* risk_factor.

2 P-value for study heterogeneity from fixed effects meta-analysis of case-control analyses per study.

3 or the highly correlated SNP rs1045485 (*r^2^* = 1 in HapMap CEU).

4 mean lifetime alcohol intake derived from duration and amount of alcohol intake in g/day at different age periods.

The polymorphism 1p11.2-rs11249433 was associated with breast cancer in parous (1.14, 1.11–1.17) but not nulliparous women (0.98, 0.92–1.05) (P_interaction_ = 5.3×10^−5^). The interaction was non-significantly stronger for risk of ER-positive than ER-negative tumours (P_heterogeneity_ = 0.13, Table S5, Table S6), corresponding to this SNP being more strongly associated with ER-positive disease (Table 3). When restricted to ER-positive breast cancer, the per-allele OR for rs11249433 was 1.16 (1.13–1.20) in parous women and 0.97 (0.90–1.04) in nulliparous women (P_interaction_ = 1.6×10^−5^) (Table 4). There was no significant heterogeneity in the interaction ORs by study (Figure 1C).

For the previously reported potential interaction between *CASP8*-rs1045485 (in complete LD with rs17468277) and alcohol consumption (<1 versus ≥1 drink/day) [6], we found moderate evidence when assessing effect modification by alcohol intake per 10 g/day increase (P_interaction_ per 10 g/day = 3.0×10^−3^) (Table S4). However, when alcohol intake was dichotomized at 20 g/day (approximately 2 drinks/day), the estimated per-allele OR for *CASP8*-rs17468277 was 0.91 (0.84–0.98) in those who drank <20 g/day and 1.45 (1.14–1.85) in those who drank ≥20 g/day (P_interaction_ = 3.1×10^−4^) (Table 4, Figure 1D).

We observed weaker evidence of differences in the associations with breast cancer for three further SNPs according to use of MHT and for one SNP according to age at first birth. These included rs13387042 and current use of combined estrogen/progestagen MHT (yes/no) (P_interaction_ = 2.4×10^−3^), rs2823093 and current use of estrogen only MHT (yes/no) (P_interaction_ = 6.6×10^−3^), rs999737 and duration of estrogen only MHT among current users (P_interaction_ = 4.0×10^−3^), and rs614367 and age at first birth among parous women (P_interaction_ = 9.1×10^−3^) (Table S4).

The observed SNP-environmental interaction ORs were not altered substantially (less than 8% change in the interaction ORs) when adjusting for additional covariates. These additional covariates included (when not the potentially effect-modifying variable of interest) ever parous (yes/no), number of births, BMI, age surrogate for postmenopausal status (≥54 years), interaction of BMI and postmenopausal status (≥54 years), current use of MHT, past use of MHT, duration of oral contraceptives (OC) use, lifetime alcohol intake, smoking (pack-years) (Table S7). Subjects with missing covariable information were excluded from these analyses, leading to considerably reduced sample sizes. Restricting the analyses to only 16 population-based studies did not change the results substantially (i.e., less than 3%) (Table S8).

The false-positive report probability (FPRP) was below 0.2 at a prior probability greater than 0.001 for the replicated effect modification of *LSP1-*rs3817198 by number of births and 1p11.2-rs11249433 and being ever parous. For the effect modification of *CASP8*-rs17468277 by alcohol intake ≥20 g/day, the FPRP was below 0.2 at a prior probability greater than 0.01. For the four potential interactions reported above, the FPRP was only below 0.2 at a prior probability greater than 0.05. (Table S9).

## Discussion

We carried out a comprehensive evaluation of potential gene-environment interactions between 23 established common susceptibility variants for breast cancer and 10 established environmental risk factors, using 18 variables. Compared to the previous analysis, the present dataset from BCAC included 5 new population-based studies as well as additional study participants from some studies [7]. We examined additional environmental risk factors (14 variables), and 11 additional recently identified common susceptibility loci.

In our previous report, the strongest evidence of effect modification (P-value = 0.002) was observed for *LSP1-*rs3817198 by number of births [7]. The highly consistent and significant finding based on the present analysis of only additional cases and controls provided clear independent replication. We also show that the interaction holds for both ER-positive and ER-negative disease. This lack of heterogeneity is biologically plausible since neither the SNP nor number of births show heterogeneity by ER status in association with breast cancer risk [9], [10]. Only ever parous versus nulliparous but not the number of births in parous women was assessed for gene-environment interaction in two previous studies [2], [6]. Consistent with our data indicating no differential effects by ever parous compared to never parous, they did not find evidence of interaction between *LSP1*-rs3817198 and ever being parous. The rs3817198 SNP is located on the short arm of chromosome 11 and lies within *LSP1*, encoding lymphocyte-specific protein 1, an intracellular F-actin binding protein, although the gene underlying the association has not been definitively identified. The SNP lies close to the H19/IGF2 imprinted region, and the association of breast cancer with rs3817198 has been reported to be restricted to the paternally inherited allele [11]. The effect heterogeneity of *LSP1*-rs3817198 by number of births appears to be partly due to a significant negative correlation between number of rs3817198 C alleles and number of births in parous women (P-value = 0.002), which was found both in the data of our previous report as well as the additional data for the present analysis. Although not statistically significant, the mean number of children was also reported to be lower in women carrying the CC genotype in the Million Women Study [6]. Also of interest is that *LSP1*-rs3817198 has been associated with mammographic density, consistent with the direction of the breast cancer association [12]. Mammographic density has also been found to be reduced after a full-term pregnancy, particularly with greater number of births [13], [14].

We also replicated the strongest finding reported in the Million Women Study based on 7,610 cases and 10,196 controls [6]. In that study, the per-allele OR of *CASP8*-rs1045485 (or rs17468277 in our dataset) was 0.99 (0.92–1.07) in those who reported <1 drink/day and 1.23 (1.09–1.38) in those who reported ≥1 drink/day (P-value = 0.003). Our observation of an increased per-allele OR of 1.45 (1.14–1.85) for those who reported high alcohol intake ≥20 g/day and 0.91 (0.84–0.98) for those who consume less provides independent replication of this SNP-environmental interaction. Although one drink corresponds to an intake of approximately 10 g alcohol, the Million Women study reported the strongest risk increase in breast cancer for women consuming at least 15 drinks per week (RR 1.29 (1.23–1.35)) [15], corresponding to approximately to 2 drinks per day (20 g alcohol). There is no known functional effect of *CASP8*-rs1045485, however, it is associated with a risk haplotype in *CASP8*, which is more strongly associated with breast cancer risk [16], [17]. Caspase 8 is an important initiator of apoptosis and is activated in response to DNA damage that can be caused by alcohol consumption through ethanol-related oxidative stress [18].

Ever being parous, but not number of births, was found to modify the effect of a different SNP, 1p11.2-rs11249433, in particular for ER-positive breast cancer. This SNP shows significantly stronger association with risk of ER-positive tumors than of ER-negative tumors [19]. In nulliparous women, rs11249433 was not associated with risk of ER-positive disease, whereas in parous women, the per-allele OR of 1.14 was slightly greater than the overall OR of 1.12. The Breast and Prostate Cancer Cohort Consortium evaluated interactions between 13 of the 23 genetic loci and 9 risk factors, including 1p11.2-rs11249433 and ever parous. They found no evidence for this interaction (P-value = 0.79), with per-allele OR of 1.09 (1.04–1.14) in parous and 1.11 (0.99–1.24) in nulliparous women [2]. These ORs are not in the same relative direction as our finding with respect to ever being parous. This may be in part due to misclassification of parity if information on parity for participants of the cohort studies was only available at time of recruitment and therefore incomplete with reference to the diagnosis of breast cancer. Their analysis was based on 8,576 cases and 11,892 controls, which had considerably lower statistical power than the present study. The SNP rs11249433 is located on the short arm of chromosome 1 close to the centromere, which makes it hard to map. The nearest known genes are *FCGR1B* (low-affinity Fc gamma receptor family) and *NOTCH2* (coding a transmembrane receptor protein). Recently, a study reported a positive association of *NOTCH2* mRNA expression with the breast cancer risk allele of rs11249433 [20]. This association was strongest with the subgroup of ER-positive breast tumors without TP53 mutation, providing some evidence that the increased risk of ER-positive breast cancer might be due to differences in *NOTCH2* expression [20].

The evidence for the other four potential interactions mentioned in the results was considerably weaker and confirmation of these findings in further studies is therefore required. Three of these involved effect modification by use of MHT. The effect modification of *RAD51L1*-rs999737 by duration of estrogen only MHT in current users is particularly interesting because this polymorphism has been associated with mammographic density in the same direction as the breast cancer association [12]. Mammographic density has also been found to be increased in postmenopausal women among users of MHT [21].

*RAD51L1* is a member of the Rad51-like proteins that play a crucial role in homologous recombinational repair [22]. Rare deleterious mutations in other genes of this pathway, including *BRCA1* and *BRCA2*, confer a high risk of breast cancer [1], [23]. Menopausal hormone therapy has been suggested to alter breast cancer risk in *BRCA1* mutation carriers although the evidence is still limited [24]. It is thus plausible that estrogen only MHT modifies the relative risk for genetic variants in *RAD51L1* on breast cancer risk.

*NRIP1* (nuclear receptor–interacting protein 1), also called *RIP140* (receptor-interacting protein 140), is known to interact with ERα, repress ER signaling and inhibit its mitogenic effects [25]. Exposure to exogenous estrogens through MHT, which stimulate ER signalling, could therefore alter the association of *NRIP1* rs2823093 with breast cancer.

It is less clear how 2q35-rs13387042 might be modified by current combined estrogen/progestagen MHT use since the gene involved at this locus is still unknown. The SNP is located on the short arm of chromosome 2 and lies in a linkage disequilibrium (LD) block containing no known gene(s) or non-coding RNAs. The closest known genes are *TNP1* (transition protein 1), *IGFBP5* (insulin-like growth factor binding protein 5), *IGFBP2* (insulin-like growth factor binding protein 2) and *TNS1* (tensin 1/matrix-remodelling-associated protein 6) [26]. The observed effect modification would suggest that the gene involved may be responsive to steroid hormones.

Both Campa et al. and the Million Women Study investigated potential interactions with MHT (overall use) [2], [6]. Neither study reported evidence for interaction between 2q35-rs13387042 or *RAD51L1*-rs999737 with MHT and breast cancer risk. However, neither study considered current use of MHT even though elevated risks for breast cancer have been clearly established for current use and not for past use [6], [27], [28]. Yet Campa et al. found differences in OR estimates for 2q35-rs13387042 by ever use of combined estrogen/progestagen MHT in the same direction as our results for current combined estrogen/progestagen MHT use, with a per-allele OR of 0.83 (0.78–0.89) in non-users and 0.77 (0.69–0.86) in ever combined estrogen/progestagen MHT users (P-value = 0.26) (in their Supplementary Table 5). We were not able to confirm the previously suggested possible interaction of 5p12-rs10941679 or *FGFR2* variants with MHT and other factors [2]–[5]. Our data suggest that age at first birth in parous women may modify the effect of 11q13-rs614367, which is located in a region containing no known genes [29]. This newly identified SNP has not been previously assessed for interaction with environmental risk factors.

One of the strengths of our study is the large sample size, required for assessing weak to moderate gene-environment interactions, particularly when marker SNPs instead of causal variants are used [30]. We assessed gene-environment interaction separately for ER-positive and ER-negative disease, thereby accounting for heterogeneity by ER status in risk associated with both genetic and environmental factors. However, statistical power was still limited to detect interactions in susceptibility to ER-negative disease. Although selection bias is likely to affect estimates of environmental main effects, under reasonable assumptions, it should not influence the assessment of multiplicative gene-environment interactions or estimates of SNP relative risks [31]. Furthermore, both non-differential and differential misclassification of exposure tend to underestimate the multiplicative interaction parameter rather than yield spurious evidence of interaction [32]. To reduce potential bias due to population stratification, we restricted our analyses to subjects of European ancestry and stratified by study in all analyses. The robustness of our findings to differences in study design was supported by sensitivity analyses considering only data from population-based studies. The interaction estimates also did not change substantially when adjusting for further covariates: the p-values were however higher due to the considerably reduced sample sizes. The absence of study heterogeneity in the estimates of gene-environment interactions provides further reassurance of the robustness of the findings.

The effect modifications identified in our study are relatively weak and should result in small differences in risk estimates of joint effects compared to those based on models assuming multiplicative effects. However, most of the SNPs investigated are only markers of the underlying causal variants and underestimate the effects of the causal variants if linkage disequilibrium is incomplete [33]. Thus, gene-environment interactions with the underlying causal variant could have a greater modifying effect on the relative risk [30]. These findings also underline the importance of investigating interactions separately for causally distinct subtypes of breast cancer in future assessments of gene-environment interaction.

In summary, we provide strong evidence of effect modification of *LSP1-*rs3817198 by number of births and of *CASP8*-rs1045485 by alcohol consumption. For some additional common genetic variants, the associations with breast cancer risk may vary with environmental factors. However, there is little evidence for multiplicative gene-environment interactions for most susceptibility loci and environmental risk factors. Understanding the biological implications of the observed interactions could provide further insight into the etiology of breast cancer. The potential impact of these results on risk prediction for breast cancer needs to be considered in future studies.

## Methods

### Study participants and risk factor data

We used primary data from the studies in BCAC. All studies had approval from the relevant ethics committees and all participants gave informed consent. A centralized BCAC database of information about common risk factors and tumor characteristics was constructed to facilitate studies of potential modifications of SNP associations by other risk factors. A multi-step data harmonization procedure was used to reconcile differences in individual study questionnaires. The reference age for cohort studies was calculated at time of enrollment and for case-control studies at date of diagnosis for cases and at date of interview for controls. All time-dependent variables were assessed at reference age. This analysis included only subjects of European ancestry that had genotype data for at least 3 SNPs and provided information on at least one of the established risk factors. Relevant data were available from 24 studies, including 16 population-based studies (14 case-control and 2 prospective cohort studies) and 8 non-population-based studies (Table 1, Table S1, Table S2). Subsets of data from 19 studies (with 11 population-based) were included in a previous report that assessed interactions between 12 susceptibility variants, reproductive history, BMI and breast cancer risk [7].

### SNP selection and genotyping

We included 21 SNPs found to be associated with overall breast cancer risk at genome-wide statistical significance (p<5×10^−7^) [10], [25], [34] and SNPs for *TGFB1* and *CASP8* from candidate gene studies [17] (Table S3). For three loci, 14q24.1/*RAD51L1*, 12p11, *CASP8*, a surrogate SNP in high linkage disequilibrium (LD) (*r^2^* = 1 in HapMap CEU) was genotyped in a subset (Table 3 footnote) [19], [25], [35].

Genotyping was performed in the framework of BCAC by Taqman and iPlex assays and underwent quality control as described previously [10], [19], [25], [34], [36], [37]. Genotype data were excluded from analysis on a study-by-study basis according to the following BCAC quality control (QC) guidelines: 1) any sample that consistently failed for >20% of the SNPs within a genotyping round, 2) all samples on any one plate that had a call rate <90% for any one SNP, 3) all genotype data for any SNP where overall call rate was <95%, 4) all genotype data for any SNP where duplicate concordance was <98%. In addition, for any SNP where the P-value for departure from Hardy-Weinberg proportions for controls was <0.005, clustering of the intensity plots was reviewed manually and the data excluded if clustering was judged to be poor.

### Statistical methods

We used logistic regression to assess the main effects of the SNP and environmental risk factors on invasive breast cancer risk. Analyses were adjusted for study as a categorical variable and reference age as a continuous variable. Odds ratios (OR) and their 95% confidence intervals (CI) were calculated for the SNP associations assuming a log-additive model and tested for association with a one degree of freedom trend test. All statistical tests were two-sided.

The assessment of associations with the environmental risk factors was based on data only from the 16 population-based studies to ensure unbiased estimates for comparison with established effect sizes. The variables considered were analyzed as continuous (age at menarche, number of births in parous women, age at first birth, usual BMI, height, duration of oral contraceptive use, duration of current use of estrogen-progestagen combined therapy, duration of current use of estrogen-only therapy, pack-years of cigarette smoking, mean lifetime daily grams of alcohol intake, recent physical activity in hours per week), or as dichotomous (ever parous, ever breastfed, ever OC use, ever smoked, current EPT use, current ET use) (Table 2). Analyses were performed for all women as well as separately for women aged <54 years and ≥54 years, considering the age groups as surrogates of pre- and postmenopausal status, Differential effects by menopausal status were assessed by adding an interaction term. For all categorical variables, the lowest level of exposure (or no use) was used as the reference. For evaluating current use of MHT by type, we used never use of MHT as the reference category and additionally adjusted for former use of MHT and other MHT type, as appropriate.

To test for interactions between SNPs and environmental risk factors, we fitted for each SNP two logistic models, a model with terms for the SNP and the risk factor of interest and another model with additionally an interaction term for the product between the SNP (number of risk alleles) and the risk factor variable. We modeled the interaction based on the risk factor variable definitions employed for the main effects. All analyses were stratified by study and adjusted for age as a continuous variable. The likelihood ratio test was used to compare the difference between the two models and departure from independent multiplicative effects of the SNP and the risk factor. BMI was the only variable found to show differential effects by menopausal status, which is consistent with the literature [38]. Therefore, interaction between SNPs and BMI was assessed separately for pre- and postmenopausal women whereas all other risk factors were evaluated regardless of menopausal status. To assess study heterogeneity, we calculated odds ratios for interaction for each individual study, adjusting for age, and reported P-values for heterogeneity using a Q-test. Subjects with missing data for a particular SNP or environmental factor were excluded from the respective analysis. We also calculated stratum specific per-allele ORs for each SNP: age at menarche (≤11, 12–13, ≥14 years), number of births (1,2,3, ≥4), age at first birth (<20, 20–24, 25–29, ≥30 years), usual BMI (<25, 25–29, ≥30), height (<160, 160–164, 165–169, ≥170 cm), duration of oral contraceptive use and of menopausal hormone use (0, >0–<5, 5–<10, ≥10 years), mean lifetime alcohol intake (0, 0–<10, 10–<20, ≥20 g/day), pack-years of smoking (0, 1–<10, 10–<20, ≥20), and physical activity (0, >0–<3.5, ≥3.5–<7, ≥7 h/week).

For SNP-environment interactions with associated P-value<10^−3^, we also compared results after adjusting for additional covariates. We performed a total of 414 (23 SNPs x 18 risk variables) tests. To account for chance findings due to multiple comparisons, we calculated the false positive report probability (FPRP) for SNP-environment interactions with associated P-value<10^−3^ [39]. The FPRP depends on the prior probability that the association between the SNP and breast cancer is modified by the environmental risk factor, the power of the present study, and the observed P-value. Since the prior probability of the assessed multiplicative interactions varies depending on subjective evaluation of existing evidence, we calculated the FPRPs for prior probabilities ranging from 0.05 to 0.0001. We considered findings with FPRP below 0.2 to be noteworthy results, as previously proposed [39].

In secondary analyses, we examined associations and effect modifications separately for women with ER-positive tumors and ER-negative tumors, each compared to all controls. Effect heterogeneity by ER status was tested using case-case analysis.

Data harmonization was performed using an ACCESS database and transformation of the data elements was performed using SAS (Release 9.2). All other data analyses were conducted using SAS (Release 9.2) and the R programming language [40].

## Supporting Information

Table S1 Description of BCAC studies included in the analysis of gene–environment interaction.
(PDF)

Table S2 Description of environmental risk factors by study.
(PDF)

Table S3 SNPs previously reported to be associated with breast cancer risk.
(PDF)

Table S4 Per-allele odds ratios (OR) and 95% confidence intervals (CI) for SNPs by environmental risk factors of breast cancer, overall.
(PDF)

Table S5 Per-allele odds ratios (OR) and 95% confidence intervals (CI) for SNPs by environmental risk factors of breast cancer, estrogen receptor positive.
(PDF)

Table S6 Per-allele odds ratios (OR) and 95% confidence intervals (CI) for SNPs by environmental risk factors of breast cancer, estrogen receptor negative.
(PDF)

Table S7 Gene-environment interactions between SNPs and breast cancer risk factors in Caucasians with interaction p-value<10^−4^, overall and by ER status, adjusted for additional covariates.
(PDF)

Table S8 Gene-environment interactions between SNPs and breast cancer risk factors in Caucasians with interaction p-value<10^−4^, overall and by ER status, restricted to population-based studies.
(PDF)

Table S9 False-positive reporting probability (FPRP) for interactions of SNPs and environmental risk factors of breast cancer showing interaction p-value<10^−2^.
(PDF)
