# Supplementary material for: Evidence of Gene–Environment Interactions between Common Breast Cancer Susceptibility Loci and Established Environmental Risk Factors
Source: PLoS Genet. 2013 Mar 27;9(3):e1003284. doi: 10.1371/journal.pgen.1003284 (PMC3609648; doi:10.1371/journal.pgen.1003284)
Supplement: Table S1 — Description of BCAC studies included in the analysis of gene–environment interaction. (PDF) [file pgen.1003284.s001.pdf]

Table S1. Description of BCAC studies included in the analysis of gene-environment interaction

Study Acronym	Study Name [Reference]	Country	Recruitment base		Sample size	
			Cases	Controls	Cases	Controls
ABCFS	Australian Breast Cancer Family Study [1]	Australia	Cancer registries in Victoria and New South Wales (1992-1999): all cases from Melbourne and Sydney diagnosed before age 40 plus a random sample of those diagnosed at ages 40-59.	Identified between 1992-1999 from the electoral rolls in Melbourne and Sydney (enrolling to vote is compulsory); frequency matched to cases by age in-5 year categories.	1335	687
BBCC	Bavarian Breast Cancer Cases and Controls [2]	Germany	Consecutive, unselected cases with invasive breast cancer recruited at the University Breast Centre, Franconia in Northern Bavaria from 2002-2006.	Healthy women aged 55 or older with no diagnosis of cancer. Invited by a newspaper advertisement in Northern Bavaria between 2002-2006.	1432	1002
BBCS	British Breast Cancer Study [3]	U.K.	(i) English & Scottish Cancer Registries: all breast cancer cases who developed a first primary before age 66 in 1971 or later and who subsequently developed a second primary cancer.<br>(ii) Breast Cancer Clinics: all breast cancer cases who developed a first primary before age 71 in 1967 or later and who either subsequently developed a second primary or had at least two affected female first-degree relatives.<br>All recruited from 2001-2008.	A friend, sister-in-law, daughter-in-law or other non-blood relative of cases, recruited from 2001-2008.	1381	1297
CECILE	CECILE Breast cancer study [4]	France	All cases diagnosed with breast cancer in 2005-2007 among women <75 years of age residing in the *départements* of Ille-et-Vilaine and Côte d'Or . Cases were recruited from the main cancer treatment center (Centre Eugène-Marquis in Rennes and Centre Georges-François-Leclerc in Dijon) and from other private or public hospitals in each area.	General population control women residing in the same areas as the cases (Ille-et-Vilaine and Côte d'Or). Controls were frequency-matched to the cases by 5-year age groups. They were recruited in 2005-2007 using a random digit dialing procedure and quotas by socioeconomic status to reflect the distribution by SES of the population in each area.	938	1026

Study Acronym	Study Name [Reference]	Country	Recruitment base		Sample size	
			Cases	Controls	Cases	Controls
CGPS	Copenhagen General Population Study [5]	Denmark	Consecutive, incident cases from one hospital with centralized care for a population of 400,000 women in Copenhagen (2001-present).	Women with no history of breast cancer residing in the same region as cases identified from the Copenhagen General Population Study (2003-2007).	2388	6704
CTS	California Teachers Study [6]	USA	Nested case-control study conducted within a cohort of California teachers (113,590) who were under age 80 years at baseline, had no prior history of invasive or *in situ* breast cancer. Cases are women newly diagnosed with a histologically confirmed invasive primary adenocarcinoma of the breast at age 80 years or younger from 1998 to 2008.	Controls are a probability sample of at-risk cohort members, frequency matched to cases on age at baseline (5-year age groups), self-reported race/ethnicity (white, African American, Latina,Asian, other), and broad geographic region within California Controls were selected without replacement, using an assigned reference date.	1252	1226
ESTHER	ESTHER Breast Cancer Study [7]	Germany	Breast cancer cases in all hospitals in the state of Saarland, from 2001-2003 (ESTHER) and 1996-1998 (VERDI).	Random sample of women a routine health check-up in Saarland, in 2000-2002; frequency matched to cases by age in-5 year categories.	433	511
GENICA	Gene Environment Interaction & Breast Cancer in Germany [8,9]	Germany	Incident breast cancer cases enrolled at hospitals in the Greater Bonn area between 2000-2004.	Random address sample selected in 2001-2004 from 31 population registries in the greater Bonn area; frequency matched to cases on year of birth in 5-year categories.	1021	1015
GESBC	Genetic Epidemiologic Study of Breast Cancer by Age 50[10]	Germany	All incident cases diagnosed <50 years of age in 1992-5 in two regions: Rhein-Neckar-Odenwald and Freiburg, by surveying the 38 clinics serving these regions	Selected from random lists of residents of the study regions supplied by population registries; two controls were selected for each case, matched by age and study region. Recruitment was carried out 1992-1998.	586	869

Study Acronym	Study Name [Reference]	Country	Recruitment base		Sample size	
			Cases	Controls	Cases	Controls
KBCP	Kuopio Breast Cancer Project [11]	Finland	Women seen at Kuopio University Hospital between 1990-1995 because of a breast lump, mammographic abnormality, or other breast symptom and who were found to have breast cancer.	Selected from the National Population Register between 1990-1995; age and long-term area-of-residence matched to cases.	466	523
LMBC	Leuven Multidisciplinary Breast Centre [12]	Belgium	All patients diagnosed with breast cancer and seen in the Multidisciplinary Breast Center in Leuven (Gashuisberg) since June 2007 plus retrospective collection of cases diagnosed since 2000.	Blood donors at Gasthuisberg Hospital (200-2008).	2890	1625
MARIE	Mammary Carcinoma Risk Factor Investigation [13]	Germany	Incident cases diagnosed from 2001-2005 in the study region Hamburg in Northern Germany, and from 2002-2005 in the study region Rhein-Neckar-Karlsruhe in Southern Germany.	2 controls per case were randomly drawn from population registries and frequency matched by birth year and study region to the case. Controls were recruited from 2002 to 2006.	2583	5309
MCBCS	Mayo Clinic Breast Cancer Study [14]	USA	Incident cases residing in 6 states (MN, WI, IA, IL, ND, SD) seen at the Mayo Clinic in Rochester, MN from 2002-2010.	Women presenting for general medical examination at the Mayo Clinic from 2002-2010; frequency matched to cases on age, ethnicity and county/state.	1803	2452
MCCS	Melbourne Collaborative Cohort Study [15]	Australia	Incident cases from the cohort of 24,469 women, diagnosed during the follow-up from baseline (1990-1994) to 2008.	Random sample of the initial cohort.	703	766
MSKCC	Memorial Sloan-Kettering Cancer Center Study[16]	USA	Incident and prevalent cases of histologically-confirmed breast cancer referred to the Clinical Genetics Service at MSKCC since July 1996. All cases tested negative for BRCA1/2 mutations.	Women who have not been diagnosed with breast cancer, but who were referred to the Clinical Genetics Service at MSKCC. All controls tested negative for BRCA1/2 mutations.	425	455

Study Acronym	Study Name [Reference]	Country	Recruitment base		Sample size	
			Cases	Controls	Cases	Controls
NC-BCFR	Northern California Breast Cancer Family Registry [17]	USA	Incident cases aged <65 years diagnosed from 1995-2003 were identified through the SEER cancer registry of the Greater San Francisco Bay Area. All cases at increased genetic risk were eligible (dx at age <35 yrs, personal history of ovarian or childhood cancer, bilateral breast cancer with 1st dx at age <50, family history of breast or ovarian cancer in first-degree relatives). Cases not meeting these criteria were randomly sampled (2.5% of whites, 32% of others).	Identified through random digit dialing, frequency matched to cases on 5-year age group and race/ethnicity, at a ratio of 1 control per 2 cases.	268	154
OFBCR	Ontario Familial Breast Cancer Registry [17]	Canada	Invasive cases aged 20-54 years identified from the Ontario Cancer Registry from 1996-1998. All those at high genetic risk were eligible; random samples of women not meeting these criteria were also asked to participate.	Identified by calling randomly selected residential telephone numbers in the same geographical region from 1998-2001; frequency matched to cases by age in 5 year categories.	1135	328
PBCS	NCI Polish Breast Cancer Study [18]	Poland	Incident cases identified through a rapid identification system in participating hospitals covering ~ 90% of all eligible cases, and cancer registries in Warsaw and Łódź covering 100% of all eligible cases (2000-2003).	Randomly selected from population lists of all residents of Poland from 2000-2003, stratified and frequency matched to cases on city and age in 5-year categories.	2009	2381
SASBAC	Singapore and Sweden Breast Cancer Study [19]	Sweden	Women diagnosed in Sweden aged 50-74 in 1993-1995.	Population-based controls frequency matched by age to the cases.	1246	1515
SBCS	Sheffield Breast Cancer Study [20]	U.K.	Women with breast cancer recruited in 1998-2005 at surgical outpatient clinics at the Royal Hallamshire Hospital, Sheffield.	Unselected women attending the Sheffield Mammography Screening Service in 2000-2004 with no evidence of a breast lesion.	1111	1283
SEARCH	Study of Epidemiology & Risk Factors in Cancer Heredity [21]	U.K.	Identified through the East Anglian Cancer Registry: (i) 1991-1996: alive, prevalent cases diagnosed before age 55; (ii) since 1996: incident cases diagnosed before age 70 diagnosed after 1996.	(a) Women from the same geographic region selected from the EPIC-Norfolk cohort study, 1992-1994 (b) women attending GP practices, frequency matched to cases by age and geographic region (2003-present).	6720	6682

Study Acronym	Study Name [Reference]	Country	Recruitment base		Sample size	
			**Cases**	**Controls**	**Cases**	**Controls**
US3SS	US Three State Study [22]	USA	Eligible cases were all English-speaking female residents of Massachusetts (excluding metropolitan Boston), New Hampshire and Wisconsin, with a new diagnosis of invasive (aged 20–69 years) or in situ breast cancer (aged 20–74 years, MA and NH only) reported to each state's mandatory cancer registry during 1998-2001.	Controls were randomly selected from driver’s license lists for women aged 20–64 years and from Medicare beneficiary lists for women aged 65–74 years in each state, and frequency-matched to cases by age in 5-year categories. Recruited 1998-2001.	1444	1274
USRTS	US Radiologic Technologists Study[23–26]	USA	Prevalent cases identified through mailed surveys in 1983-8 and 1994-8, incident cases between surveys; blood collected from 1999-2004; unselected for family cancer history or any other characteristics; most cases sampled more than 5 years after diagnosis	Selected from women within the cohort without breast cancer as of 1999, blood collected between 2000-2004; matched to cases on year of birth in 5-year strata	725	1053
kConFab/ AOCS	Kathleen Cuningham Foundation Consortium for Research into Familial Breast Cancer / Australian Ovarian Cancer Study [27]	Australia	Index (youngest affected) cases from *BRCA1*- and *BRCA2*-mutation-negative multiple-case breast and breast-ovarian families recruited though family cancer clinics from across Australia and New Zealand from 1998-present.	Identified from the electoral rolls from across Australia as part of the Australian Ovarian Cancer Study in 2002-2006.	499	962

Reference List

1. Dite GS, Jenkins MA, Southey MC, Hocking JS, Giles GG et al. (2003) Familial risks, early-onset breast cancer, and BRCA1 and BRCA2 germline mutations. J Natl Cancer Inst 19;95: 448-457.

2. Schrauder M, Frank S, Strissel PL, Lux MP, Bani MR et al. (2008) Single nucleotide polymorphism D1853N of the ATM gene may alter the risk for breast cancer. J Cancer Res Clin Oncol 134: 873-882.

3. Fletcher O, Johnson N, Palles C, dos SS, I, McCormack V et al. (2006) Inconsistent association between the STK15 F31I genetic polymorphism and breast cancer risk. J Natl Cancer Inst 98: 1014-1018.

4. Menegaux F, Truong T, Anger A, Cordina-Duverger E, Lamkarkach F et al. (2012) Night work and breast cancer: A population-based case-control study in France (the CECILE study). Int J Cancer .

5. Weischer M, Bojesen SE, Tybjaerg-Hansen A, Axelsson CK, Nordestgaard BG (2007) Increased risk of breast cancer associated with CHEK2*1100delC. J Clin Oncol 25: 57-63.

6. Seal S, Thompson D, Renwick A, Elliott A, Kelly P et al. (2006) Truncating mutations in the Fanconi anemia J gene BRIP1 are low-penetrance breast cancer susceptibility alleles. Nat Genet 38: 1239-1241.

7. Widschwendter M, Apostolidou S, Raum E, Rothenbacher D, Fiegl H et al. (2008) Epigenotyping in peripheral blood cell DNA and breast cancer risk: a proof of principle study. PLoS One 3: e2656.

8. Justenhoven C, Pierl CB, Haas S, Fischer HP, Baisch C et al. (2008) The CYP1B1_1358_GG genotype is associated with estrogen receptor-negative breast cancer. Breast Cancer Res Treat 111: 171-177.

9. Pesch B, Ko Y, Brauch H, Hamann U, Harth V et al. (2005) Factors modifying the association between hormone-replacement therapy and breast cancer risk. Eur J Epidemiol 20: 699-711.

10. Chang-Claude J, Eby N, Kiechle M, Bastert G, Becher H (2000) Breastfeeding and breast cancer risk by age 50 among women in Germany. Cancer Causes Control 11: 687-695.

11. Hartikainen JM, Tuhkanen H, Kataja V, Dunning AM, Antoniou A et al. (2005) An autosome-wide scan for linkage disequilibrium-based association in sporadic breast cancer cases in eastern Finland: three candidate regions found. Cancer Epidemiol Biomarkers Prev 14: 75-80.

12. De ML, Van LE, De NK, Moerman P, Pochet N et al. (2008) Does estrogen receptor negative/progesterone receptor positive breast carcinoma exist? J Clin Oncol 26: 335-336.

13. Flesch-Janys D, Slanger T, Mutschelknauss E, Kropp S, Obi N et al. (2008) Risk of different histological types of postmenopausal breast cancer by type and regimen of menopausal hormone therapy. Int J Cancer 123: 933-941.

14. Olson JE, Ma CX, Pelleymounter LL, Schaid DJ, Pankratz VS et al. (2007) A comprehensive examination of CYP19 variation and breast density. Cancer Epidemiol Biomarkers Prev 16: 623-625.

15. Giles GG, English DR (2002) The Melbourne Collaborative Cohort Study. IARC Sci Publ 156: 69-70.

16. Comen E, Balistreri L, Gonen M, Dutra-Clarke A, Fazio M et al. (2011) Discriminatory accuracy and potential clinical utility of genomic profiling for breast cancer risk in BRCA-negative women. Breast Cancer Res Treat 127: 479-487.

17. John EM, Hopper JL, Beck JC, Knight JA, Neuhausen SL et al. (2004) The Breast Cancer Family Registry: an infrastructure for cooperative multinational, interdisciplinary and translational studies of the genetic epidemiology of breast cancer. Breast Cancer Res 6: R375-R389.

18. Garcia-Closas M, Brinton LA, Lissowska J, Chatterjee N, Peplonska B et al. (2006) Established breast cancer risk factors by clinically important tumour characteristics. Br J Cancer 95: 123-129.

19. Wedren S, Lovmar L, Humphreys K, Magnusson C, Melhus H et al. (2004) Oestrogen receptor alpha gene haplotype and postmenopausal breast cancer risk: a case control study. Breast Cancer Res 6: R437-R449.

20. MacPherson G, Healey CS, Teare MD, Balasubramanian SP, Reed MW et al. (2004) Association of a common variant of the CASP8 gene with reduced risk of breast cancer. J Natl Cancer Inst 96: 1866-1869.

21. Lesueur F, Pharoah PD, Laing S, Ahmed S, Jordan C et al. (2005) Allelic association of the human homologue of the mouse modifier Ptprj with breast cancer. Hum Mol Genet 14: 2349-2356.

22. Garcia-Closas M, Egan KM, Newcomb PA, Brinton LA, Titus-Ernstoff L et al. (2006) Polymorphisms in DNA double-strand break repair genes and risk of breast cancer: two population-based studies in USA and Poland, and meta-analyses. Hum Genet 119: 376-388.

23. Sigurdson AJ, Doody MM, Rao RS, Freedman DM, Alexander BH et al. (2003) Cancer incidence in the US radiologic technologists health study, 1983-1998. Cancer 97: 3080-3089.

24. Bhatti P, Doody MM, Alexander BH, Yuenger J, Simon SL et al. (2008) Breast cancer risk polymorphisms and interaction with ionizing radiation among U.S. radiologic technologists. Cancer Epidemiol Biomarkers Prev 17: 2007-2011.

25. Rajaraman P, Bhatti P, Doody MM, Simon SL, Weinstock RM et al. (2008) Nucleotide excision repair polymorphisms may modify ionizing radiation-related breast cancer risk in US radiologic technologists. Int J Cancer 123: 2713-2716.

26. Sigurdson AJ, Bhatti P, Chang SC, Rajaraman P, Doody MM et al. (2009) Polymorphisms in estrogen biosynthesis and metabolism-related genes, ionizing radiation exposure, and risk of breast cancer among US radiologic technologists. Breast Cancer Res Treat 118: 177-184.

27. Beesley J, Jordan SJ, Spurdle AB, Song H, Ramus SJ et al. (2007) Association between single-nucleotide polymorphisms in hormone metabolism and DNA repair genes and epithelial ovarian cancer: results from two Australian studies and an additional validation set. Cancer Epidemiol Biomarkers Prev 16: 2557-2565.
