# Supplementary material for: Evidence of Gene–Environment Interactions between Common Breast Cancer Susceptibility Loci and Established Environmental Risk Factors
Source: PLoS Genet. 2013 Mar 27;9(3):e1003284. doi: 10.1371/journal.pgen.1003284 (PMC3609648; doi:10.1371/journal.pgen.1003284)
Supplement: Table S2 — Description of environmental risk factors by study. (PDF) [file pgen.1003284.s002.pdf]

Table S2. Description of environmental risk factors by study

	Population-based studies															
	ABCFS		CECILE		CGPS		CTS		ESTHER		GENICA		GESBC		KBCP	
Variable	cases	controls	cases	controls	cases	controls	cases	controls	cases	controls	cases	controls	cases	controls	cases	controls
Total number	1335	687	938	1026	2388	6704	1252	1226	428	511	1021	1015	586	869	466	523
Reference age:																
mean	42.4	41.6	54.4	54.7	62.0	55.7	61.8	56.2	60.3	62.3	58.2	58.2	42.6	42.7	59.0	52.9
range	23-69	20-68	25-74	25-74	27-95	20-91	32-83	26-77	30-79	49-75	23-80	24-80	20-50	24-52	23-92	17-77
Age at Menarche:																
mean	12.8	12.9	12.9	13.1	13.6	13.5	12.5	12.5	13.5	13.7	13.4	13.6	13.1	13.1	13.8	13.6
range	8-18	9-21	9-18	9-21	8-22	8-20	10-17	10-17	9-18	9-19	8-21	8-20	9-19	9-20	10-19	10-18
unknown	6	1	11	10	52	117	16	15	5	9	1	3	3	1	42	50
Parous																
no	296	166	104	70	271	761	269	233	49	43	182	177	127	182	99	78
yes	1039	521	834	956	1900	5899	968	976	378	461	839	837	459	687	341	419
unknown	0	0	0	0	217	44	15	17	1	7	0	1	0	0	26	26
Numbers of births:																
mean	2.3	2.4	2.7	3.0	2.1	2.1	2.4	2.5	2.3	2.3	2.1	2.1	1.8	1.9	2.6	2.6
range	1-9	1-6	1-15	1-12	1-6	1-8	1-9	1-9	1-11	1-12	1-11	1-12	1-6	1-8	1-9	1-12
unknown	0	0	0	0	274	51	15	17	11	34	0	1	0	0	26	26
Age at first birth:																
mean	25.6	25.8	24.8	24.1	24.8	24.9	26.1	25.8			25.7	25.5	24.3	24.3	24.8	24.5
range	14-40	14-42	15-42	14-42	15-42	15-43	14-41	16-42			15-41	16-45	15-39	14-41	17-47	16-42
unknown	0	0	0	1	263	61	15	17			1	2	0	0	28	26
Ever breastfed:																
no	148	60	275	291							287	274	149	207	16	16
yes	891	461	417	512							547	560	310	480	325	403
unknown	0	0	142	153							5	4	0	0	26	26
Usual adult BMI, age <54:																
mean	24.3	24.5	23.0	24.4							24.7	24.8	24.2	24.2	26.0	26.1
range	15-44	17-44	15-38	15-55							17-47	17-52	14-44	17-48	17-44	17-48
unknown	0	0	9	9							0	0	1	0	20	36
Usual adult BMI, age >=54:																
mean	25.9	26.5	25.9	25.5							25.6	25.7			27.2	26.8
range	18-44	19-41	15-48	15-52							17-53	17-43			15-52	17-46
unknown	1	0	28	48							0	1			32	26
Adult height:																
mean (cm):	164	163	162	161			165	165	163	162	166	165	165	165	162	161
range	145-185	145-183	134-180	140-180			145-193	145-186	140-184	143-180	147-184	144-183	140-184	142-182	144-177	143-176
unknown	1	0	36	55			30	42	3	6	0	1	0	0	31	50
Oral contraceptives:																
never	220	104	310	316					179	233	399	387	73	118	283	240
ever	1115	583	628	709					245	266	622	627	403	564	130	198
Unknown	0	0	0	1					4	12	0	1	110	187	53	85
Duration of OC use:																
mean (years):	8.3	7.7	11.3	10.7					11.6	11.2	11.2	10.9	11.4	11.5	5.1	4.5
range	1-28	1-26	1-35	1-35					1-35	1-40	1-35	1-33	1-28	1-31	1-18	1-21
unknown	3	3	14	17					13	24	4	4	120	194	53	85
Current use of estrogen/ progestagen MHT:																
no			406	469							582	612				

Population-based studies

Variable	ABCFS		CECILE		CGPS		CTS		ESTHER		GENICA		GESBC		KBCP	
	cases	controls	cases	controls	cases	controls	cases	controls	cases	controls	cases	controls	cases	controls	cases	controls
yes			58	48							92	63				
unknown			42	52							0	1				
Current use of estrogen only MHT:																
no			444	497							606	584			243	203
yes			22	21							67	91			1	1
unknown			40	51							1	1			24	31
Duration of estrogen/ progestagen MHT in current users:																
mean (years):			7.7	3.5							9.7	8.8				
range			4-13	1-6							1-33	1-24				
unknown			114	128							7	7				
Duration of estrogen only MHT in current users:																
mean (years):			4.3	4.0							13.4	9.6			14.0	18.0
range			1-11	1-10							1-34	1-23			14-14	18-18
unknown			72	100							6	11			32	43
Mean lifetime intake of alcohol[1]:																
mean (g/day):			6.6	7.0									10.9	8.2		
range			0-117	0-90									0-128	0-89		
unknown			0	0									0	0		
Smoking:																
never	627	339	569	613					263	335	586	555	273	401	361	394
ever	707	348	369	413					162	164	434	460	312	468	105	129
unknown	1	0	0	0					3	12	1	0	1	0	0	0
Pack-years of smoking:																
mean	14.0	12.2	12.9	12.3							20.2	20.4	15.3	13.3	10.2	8.3
range	0-129	0-57	0-76	0-123							0-100	0-92	0-78	0-56	0-52	0-50
unknown	14	12	10	10							61	66	6	7	1	0
Physical activity in recent year:																
mean (h/week):			2.9	2.6							2.3	2.2				
range			0-25	0-12							1-4	1-4				
unknown			1	0							0	1				

Population-based studies (continued)

Variable	MARIE		MCCS		NC_BCFR		OFBCR		PBCS		SASBAC		US3SS		USRT	
	cases	controls	cases	controls	cases	controls	cases	controls	cases	controls	cases	controls	cases	controls	cases	controls
Total numbers	2583	5309	703	766	268	154	1135	328	2009	2381	1246	1515	1444	1274	725	1053
Reference age:																
mean	62.5	61.9	61.4	57.2	56.9	56.9	53.8	57.4	55.7	55.7	63.0	63.4	54.3	54.3	48.9	62.8
range	50-75	49-75	37-80	38-70	26-65	51-65	22-81	40-69	27-75	24-75	50-75	49-76	29-69	27-75	22-82	42-94
Age at Menarche:																
mean	13.6	13.7	13.0	13.0	12.7	12.6	12.7	12.7	13.5	13.7	13.5	13.5	12.6	12.8	12.5	12.6
range	9-20	8-20	9-20	9-21	9-18	8-16	8-18	9-21	9-21	8-20	9-18	8-21	7-18	8-22	8-18	8-35
unknown	279	819	2	3	1	0	167	2	15	28	108	128	17	10	17	9
Parous:																
no	432	783	131	105	51	24	318	39	289	260	177	148	195	165	161	202
yes	2151	4526	572	661	217	130	817	289	1720	2121	1069	1367	1247	1106	563	849
unknown	0	0	0	0	0	0	0	0	0	0	0	0	2	3	1	2
Numbers of births:																
mean	2.0	2.0	2.8	2.9	2.5	2.3	2.6	2.5	1.7	1.9	2.2	2.4	2.8	2.9	2.5	2.6
range	1-7	1-9	1-11	1-11	1-7	1-5	1-8	1-6	1-10	1-7	1-8	1-13	1-10	1-11	1-6	1-12
unknown	0	0	0	0	0	0	1	0	0	0	0	0	2	3	1	2
Age at first birth																
mean	24.4	24.3	25.5	25.2	24.0	24.0	25.0	24.0	24.5	23.7	25.5	24.7	24.1	23.8	25.8	25.1
range	14-46	14-44	17-43	15-46	14-43	15-41	15-41	16-38	15-45	15-42	16-42	16-51	14-44	16-41	14-45	13-42
unknown	2	1	2	0	0	0	8	0	0	0	2	0	4	4	1	3
Ever breastfed:																
no	530	964	39	54	78	55	262	99	375	405	48	53	658	579		
yes	1621	3562	521	594	139	75	555	190	1345	1716	899	1049	587	523		
unknown	0	0	12	13	0	0	0	0	0	0	122	265	4	7		
Usual adult BMI, age <54																
mean	23.0	23.3			25.9	26.6	25.1	26.3	22.2	22.5	24.4	24.3	25.8	25.7		
range	17-43	16-52			17-47	18-45	14-68	7-45	16-36	16-38	17-39	18-31	17-57	17-57		
unknown	1	15			0	0	136	1	1	4	2	2	1	5		
Usual adult BMI, age >=54:																
mean	23.3	23.3			26.2	28.5	26.3	26.5	23.5	23.6	25.7	25.5	27.1	26.7		
range	16-50	16-45			18-43	20-62	16-51	14-48	15-44	17-39	16-53	16-65	14-58	15-51		
unknown	40	96			2	1	74	1	14	19	53	74	8	6		
Adult height:																
mean (cm):	165	164	161	160	166	164	162	163	162	161	164	164	165	164	164	163
range	139-187	143-186	142-181	140-188	150-183	150-180	99-186	150-196	136-185	130-189	147-190	128-183	145-200	142-191	130-183	132-198
unknown	0	0	0	1	0	0	167	0	1	3	4	10	0	1	5	4
Oral contraceptives OC:																
never	958,	1888	310	353	81	31			1758	2108	847	999	627	553		
ever	1605	3373	391	413	187	123			231	250	395	511	815	720		
unknown	20	48	2	0	0	0			20	23	4	5	2	1		
Duration of OC use:																
mean (years):	10.5	10.1	7.1	7.4	7.1	5.2			4.4	4.3	6.3	6.2	6.3	6.3		
range	1-35	1-36	1-40	1-29	1-30	1-20			1-34	1-23	1-25	1-25	1-30	1-35		
unknown	27	70	17	22	0	0			37	40	60	85	8	8		
Current use of estrogen/ progestagen MHT:																
no	1669	3832							945	1179	918	1215	548	550		
yes	691	921							104	61	209	173	218	130		

Population-based studies (continued)

Variable	MARIE		MCCS		NC_BCFR		OFBCR		PBCS		SASBAC		US3SS		USRT	
	cases	controls	cases	controls	cases	controls	cases	controls	cases	controls	cases	controls	cases	controls	cases	controls
unknown	7	25							58	67	26	41	0	1		
Current use of estrogen only MHT:																
no	2088	4157							1044	1227	884	1185	607	540		
yes	273	602							6	12	262	227	159	140		
unknown	6	19							57	68	7	17	0	1		
Duration of estrogen/ progestagen MHT in current users:																
mean (years):	10.6	10.5							6.3	6.6	7.3	6.0	8.2	6.0		
range	1-34	1-37							1-20	1-26	1-19	1-20	1-27	1-25		
unknown	14	38							60	67	26	41	0	1		
Duration of estrogen only MHT in current users:																
mean (years):	10.4	9.8							3.5	2.7	8.5	7.6	13.2	13.9		
range	1-29	1-33							1-10	1-6	1-29	1-32	1-37	1-40		
unknown	7	25							60	69	8	20	26	30		
Mean lifetime intake of alcohol[1]:																
mean (g/day):	10.1	9.6	13.2	11.8					5.8	6.2						
range	0-430	0-195	0-130	0-78					0-197	0-233						
unknown	10	22	1	0					45	56						
Smoking:																
never	1401	2793	488	509			466	162	855	1102	707	867	667	605		
ever	1182	2514	215	257			509	166	1152	1278	539	648	776	669		
unknown	0	2	0	0			160	0	2	1	0	0	1	0		
Pack-years of smoking:																
mean	14.9	14.3	21.4	19.9			21.0	20.9	10.3	11.1			20.8	20.9		
range	0-104	0-101	0-135	0-112			0-154	0-66	0-84	0-90			0-180	0-140		
unknown	5	23	1	1			209	0	21	13			2	2		
Physical activity in recent year:																
mean (h/week):	9.4	9.7									2.7	2.7	3.8	3.8		
range	0-55	0-62									1-4	1-4	0-41	0-41		
unknown	35	63									8	54	0	0		

Studies of mixed design or hospital-based studies

variable	BBCC		BBCS		kConFab/AOCS		LMBC		MCBCS		MSKCC		SBCS		SEARCH	
	cases	controls	cases	controls	cases	controls	cases	controls	cases	controls	cases	controls	cases	controls	cases	controls
Reference age: n	1432	1002	1381	1297	499	962	2779	1621	1803	2452	425	455	1111	1283	6719	6503
mean	55.4	57.2	53.9	51.4	45.0	58.3	56.6	44.1	56.8	56.6	47.1	47.0	59.0	57.7	53.3	58.4
range	22-96	18-100	25-77	21-81	20-76	20-83	21-94	19-66	22-93	19-91	23-85	24-86	28-92	45-80	23-88	26-81
Age at Menarche:																
mean	13.6	13.4	12.8	12.8					12.7	12.8	12.5	12.7	13.1	13.0	12.8	13.0
range	9-21	9-22	8-20	8-18					9-19	9-18	8-21	9-19	9-20	9-18	8-19	8-21
n unknown	85	106	19	344					204	309	7	3	17	14	369	1762
Parous:																
no	181	152	209	240	57	100	330	125	221	456	12	24	172	132	861	706
yes	1177	772	1155	1023	442	862	2010	1130	1520	1851	365	390	939	1151	5765	4293
unknown	74	78	17	34	0	0	439	366	62	145	48	41	0	0	93	1504
Numbers of births:																
mean	2.0	2.4	2.3	2.3			2.2	2.0	2.8	2.8			2.3	2.3	2.3	2.4
range	1-9	1-8	1-13	1-12			1-12	1-8	1-13	1-14			1-8	1-8	1-11	1-10
unknown	77	78	17	34			468	368	62	145			0	0	93	1506
Age at first birth:																
mean	25.5	24.5	25.0	25.7					24.3	23.9	28.1	29.3	24.6	23.6	24.1	25.3
range	16-44	15-41	15-42	15-47					14-51	15-42	16-43	16-44	15-41	13-43	11-45	16-46
unknown	101	117	17	59					79	158	121	92	24	5	257	1522
Ever breastfed:																
no					72	23										
yes					368	730										
unknown					2	109										
Usual adult BMI, age <54:																
mean					26.1	26.6										
range					17-46	18-53										
unknown					87	8										
Usual adult BMI, age >=54:																
mean					25.7	26.9										
range					16-47	16-65										
unknown					17	22										
Adult height:																
mean (cm):	164	164	163	163	164	163	163	164	165	164			162	162		
range	140-188	146-181	142-191	142-201	120-189	124-183	135-198	146-186	130-212	122-201			142-183	137-183		
unknown	87	123	13	42	3	7	646	438	156	169			19	1		
Oral contraceptives OC:																
never			540	574	75	197			548	475			654	500		
ever			835	661	423	765			1085	1454			438	780		
unknown			6	62	1	0			170	523			19	3		
Duration of OC use:																
mean (years):			7.0	8.4	8.3	9.4			7.4	7.1			6.9	6.6		
range			1-33	1-30	1-30	1-36			1-30	1-35			1-29	1-40		
unknown			49	153	11	4			204	566			28	9		
Current use of estrogen/ progestagen MHT:																
no																

Studies of mixed design or hospital-based studies

variable	BBCC		BBCS		kConFab/AOCS		LMBC		MCBCS		MSKCC		SBCS		SEARCH	
	cases	controls	cases	controls	cases	controls	cases	controls	cases	controls	cases	controls	cases	controls	cases	controls
yes																
unknown																
Current use of estrogen only MHT:																
no																
yes																
unknown																
Duration of estrogen/ progestagen MHT in current users:																
mean (years):																
range																
unknown																
Duration of estrogen only MHT in current users:																
mean (years):																
range																
unknown																
Mean lifetime intake of alcohol[1]:																
mean (g/day):																
range																
unknown																
Smoking:																
never	1024	512	629	647	293	562			994	1245						
ever	332	360	724	611	202	397			673	698						
unknown	76	130	28	39	4	3			136	509						
Pack-years of smoking:						4										
mean	15.4	12.5	17.7	13.3	15.7	14.3			17.7	15.5						
range	0-80	0-80	0-92	0-100	0-179	0-72			0-117	0-114						
unknown	272	207	66	88	11	4			473	642						
Physical activity in recent year:																
mean (h/week):																
range																
unknown																

[1] Mean lifetime alcohol intake derived from duration and amount of alcohol intake in g/day at different age periods
