# Supplementary material for: Evidence of Gene–Environment Interactions between Common Breast Cancer Susceptibility Loci and Established Environmental Risk Factors
Source: PLoS Genet. 2013 Mar 27;9(3):e1003284. doi: 10.1371/journal.pgen.1003284 (PMC3609648; doi:10.1371/journal.pgen.1003284)
Supplement: Table S3 — SNPs previously reported to be associated with breast cancer risk. (PDF) [file pgen.1003284.s003.pdf]

Table S3. SNPs previously reported to be associated with breast cancer risk

SNP	Locus	Gene	Original reference<br>orig. Reference.	RR per allele	P trend	BCAC replication<br>BCAC ref.	RR per allele	P trend
rs11249433	1p11	-	Thomas et al. 2009 [1]	1.16	$6.74x10^{-10}$	Figueroa et al. 2011 [2]	1.1	$2.7x10^{-17}$
rs13387042	2q35	-	Stacey et al. 2007 [3]	1.2	$1.3x10^{-13}$	Milne et al. 2011 [4]	1.12	$1.0x10^{-19}$
rs17468277[1]	2q33	CASP8	Cox et al. 2007 [5]	0.88	$1.1x10^{-7}$	Milne et al. 2010 [6]	0.88	$5.7x10^{-7}$
rs4973768	3p24	SLC4A7	Ahmed et al. 2009 [7]	1.11	$4.1x10^{-23}$	Broeks et al 2011 [8]	1.11	$1.1x10^{-17}$
rs889312	5q11	MAP3K1	Easton et al. 2007 [9]	1.13	$7x10^{-20}$	Turnbull et al. 2010 [10]	1.22	$4.6x10^{-9}$
rs10941679	5p12	-	Stacey et al. 2008 [11]	1.19	$2.9x10^{-11}$	Milne et al. 2011 [12]	1.11	$7x10^{-18}$
rs2046210	6q25	ESR1	Zheng et al. 2009 [13]	1.29	$2.0x10^{-15}$	Turnbull et al. 2010[2] [10]	1.15	$1.8x10^{-5}$
rs12662670	6q25	ESR1	Turnbull et al. 2010[3] [10]	1.30	$2.9x10^{-6}$	Hein et al. 2012 [14]	1.12	$3.8x10^{-9}$
rs13281615	8q24	-	Easton et al. 2007 [9]	1.08	$5x10^{-12}$	Broeks et al 2011 [8]	1.11	$3.5x10^{-15}$
rs1011970	9p.21	CDKN2A/ B	Turnbull et al. 2010 [10]	1.09	$2.5x10^{-8}$	Lambrechts et al. 2012 [15]		$< 3x10^{-9}$
rs865686	9q31	-	Fletcher et al. 2011 [16]	0.98	$1.75x10^{-10}$			
rs2981582	10q26	FGFR2	Easton et al. 2007 [9]	1.26	$2x10^{-76}$	Turnbull et al. 2010 [10]	1.43	$3.6x10^{-31}$
rs10995190	10q21	ZNF365	Turnbull et al. 2010 [10]	0.86	$5.1x10^{-15}$	Lambrechts et al. 2012 [15]		$< 3x10^{-9}$
rs704010	10q22	ZMIZ1	Turnbull et al. 2010 [10]	1.07	$3.7x10^{-9}$	Lambrechts et al. 2012 [15]		$< 3x10^{-9}$
rs3817198	11p15	LSP1	Easton et al. 2007 [9]	1.07	$3x10^{-9}$	Broeks et al 2011 [8]	1.06	$1.0x10^{-5}$
rs614367	11q13	-	Turnbull et al. 2010 [10]	1.15	$3.2x10^{-15}$	Lambrechts et al. 2012 [15]		$< 3x10^{-9}$
rs1975930[4]	12p11	PTHLH	Ghoussaini et al 2012 [17]	0.85	$2.7x10^{-35}$			
rs1292011	12q24	-	Ghoussaini et al 2012 [17]	0.92	$4.3x10^{-19}$			
rs999737[5]	14q24	RAD51L1	Thomas et al. 2009 [1]	0.94	$1.74x^{10-7}$	Figueroa et al. 2011 [2]	0.92	$8.3x10^{-14}$
rs3803662	16q12	TOX3	Easton et al. 2007 [9]	1.2	$1.00x10^{-37}$	Broeks et al 2011 [8]	1.24	$3.0x10^{-59}$
rs6504950	17q23	COX11	Ahmed et al. 2009 [7]	0.95	$1.4x10^{-8}$	Broeks et al 2011 [8]	0.94	$3.2x10^{-5}$
rs1982073	19q13	TGFB1	Cox et al. 2007 [5]	1.08	$1.5x10^{-4}$	Broeks et al 2011 [8]	1.04	0.003
rs2823093	21q21	-	Ghoussaini et al 2012 [17]	0.94	$1.1x10^{-12}$			

[1] or highly correlated SNP rs1045485 (r^2= 1 in HapMap CEU)
[2] highly correlated surrogate SNP rs6900157 (r^2= 0.96 in HapMap CEU)
[3] rs3757318 used by Turnbull et al. , highly correlated with SNP rs12662670 (r^2= 0.9, Hein et. al.)
[4]or highly correlated SNP rs10771399 (r^2= 1 in HapMap CEU)
[5] or highly correlated SNP rs10483813 (r^2= 1 in HapMap CEU)

Reference List

1. Thomas G, Jacobs KB, Kraft P, Yeager M, Wacholder S et al. (2009) A multistage genome-wide association study in breast cancer identifies two new risk alleles at 1p11.2 and 14q24.1 (RAD51L1). Nat Genet 41: 579-584.

2. Figueroa JD, Garcia-Closas M, Humphreys M, Platte R, Hopper JL et al. (2011) Associations of common variants at 1p11.2 and 14q24.1 (RAD51L1) with breast cancer risk and heterogeneity by tumor subtype: findings from the Breast Cancer Association Consortium. Hum Mol Genet 20: 4693-4706.

3. Stacey SN, Manolescu A, Sulem P, Rafnar T, Gudmundsson J et al. (2007) Common variants on chromosomes 2q35 and 16q12 confer susceptibility to estrogen receptor-positive breast cancer. Nat Genet 39: 865-869.

4. Milne RL, Benitez J, Nevanlinna H, Heikkinen T, Aittomaki K et al. (2009) Risk of estrogen receptor-positive and -negative breast cancer and single-nucleotide polymorphism 2q35-rs13387042. J Natl Cancer Inst 101: 1012-1018.

5. Cox A, Dunning AM, Garcia-Closas M, Balasubramanian S, Reed MW et al. (2007) A common coding variant in CASP8 is associated with breast cancer risk. Nat Genet 39: 352-358.

6. Milne RL, Gaudet MM, Spurdle AB, Fasching PA, Couch FJ et al. (2010) Assessing interactions between the associations of common genetic susceptibility variants, reproductive history and body mass index with breast cancer risk in the breast cancer association consortium: a combined case-control study. Breast Cancer Res 12: R110.

7. Ahmed S, Thomas G, Ghoussaini M, Healey CS, Humphreys MK et al. (2009) Newly discovered breast cancer susceptibility loci on 3p24 and 17q23.2. Nat Genet 41: 585-590.

8. Broeks A, Schmidt MK, Sherman ME, Couch FJ, Hopper JL et al. (2011) Low penetrance breast cancer susceptibility loci are associated with specific breast tumor subtypes: findings from the Breast Cancer Association Consortium. Hum Mol Genet 20: 3289-3303.

9. Easton DF, Pooley KA, Dunning AM, Pharoah PD, Thompson D et al. (2007) Genome-wide association study identifies novel breast cancer susceptibility loci. Nature 447: 1087-1093.

10. Turnbull C, Ahmed S, Morrison J, Pernet D, Renwick A et al. (2010) Genome-wide association study identifies five new breast cancer susceptibility loci. Nat Genet 42: 504-507.

11. Stacey SN, Manolescu A, Sulem P, Thorlacius S, Gudjonsson SA et al. (2008) Common variants on chromosome 5p12 confer susceptibility to estrogen receptor-positive breast cancer. Nat Genet 40: 703-706.

12. Milne RL, Goode EL, Garcia-Closas M, Couch FJ, Severi G et al. (2011) Confirmation of 5p12 as a susceptibility locus for progesterone-receptor-positive, lower grade breast cancer. Cancer Epidemiol Biomarkers Prev 20: 2222-2231.

13. Zheng W, Long J, Gao YT, Li C, Zheng Y et al. (2009) Genome-wide association study identifies a new breast cancer susceptibility locus at 6q25.1. Nat Genet 41: 324-328.

14. Hein R, Maranian M, Hopper JL, Kapuscinski MK, Southey MC et al. (2012) Comparison of 6q25 Breast Cancer Hits from Asian and European Genome Wide Association Studies in the Breast Cancer Association Consortium (BCAC). PLoS One 7: e42380.

15. Lambrechts D, Truong T, Justenhoven C, Humphreys MK, Wang J et al. (2012) 11q13 is a Susceptibility Locus for Hormone Receptor Positive Breast Cancer. Hum Mutat 10.

16. Fletcher O, Johnson N, Orr N, Hosking FJ, Gibson LJ et al. (2011) Novel breast cancer susceptibility locus at 9q31.2: results of a genome-wide association study. J Natl Cancer Inst 103: 425-435.

17. Ghoussaini M, Fletcher O, Michailidou K, Turnbull C, Schmidt MK et al. (2012) Genome-wide association analysis identifies three new breast cancer susceptibility loci. Nat Genet 44: 312-318.
