# Supplementary material for: Evidence of Gene–Environment Interactions between Common Breast Cancer Susceptibility Loci and Established Environmental Risk Factors
Source: PLoS Genet. 2013 Mar 27;9(3):e1003284. doi: 10.1371/journal.pgen.1003284 (PMC3609648; doi:10.1371/journal.pgen.1003284)
Supplement: Table S4 — Per-allele odds ratios (OR) and 95% confidence intervals (CI) for SNPs by environmental risk factors of breast cancer, overall. (PDF) [file pgen.1003284.s004.pdf]

Table S4. Per-allele odds ratios (OR) and 95% confidence intervals (CI) for SNPs by environmental risk factors of breast cancer, overall

SNP	Variable	Stratum	N (cases/ controls)	OR (95% CI)[1]	OR int[2]	P int[3]
rs1011970	Age at menarche (years)	<=11	2586/ 2920	1.06 (0.96-1.18)		
rs1011970	Age at menarche (years)	12-13	8561/10797	1.09 (1.03-1.16)		
rs1011970	Age at menarche (years)	>=14	7473/10850	1.07 (1.01-1.14)		
rs1011970	Age at menarche (years/2)	combined	18620/24567		1.01	5.6E-01
rs1011970	Parous	no	3651/ 3808	1.07 (0.98-1.17)		
rs1011970	Parous	yes	18939/24161	1.08 (1.04-1.12)		
rs1011970	Parous (yes/no)	combined	22590/27969		1.01	8.1E-01
rs1011970	Number of births (among parous)	1	4464/ 5136	1.13 (1.04-1.22)		
rs1011970	Number of births (among parous)	2	8253/11012	1.04 (0.98-1.10)		
rs1011970	Number of births (among parous)	3	3778/ 4819	1.13 (1.04-1.23)		
rs1011970	Number of births (among parous)	>=4	1930/ 2321	1.07 (0.95-1.20)		
rs1011970	Number of births (among parous)	combined	18425/23288		1.00	8.2E-01
rs1011970	Age at first birth (among parous, years)	<20	1722/ 2480	1.08 (0.96-1.22)		
rs1011970	Age at first birth (among parous, years)	20-24	6543/ 9285	1.07 (1.00-1.14)		
rs1011970	Age at first birth (among parous, years)	25-29	5184/ 6903	1.08 (1.01-1.16)		
rs1011970	Age at first birth (among parous, years)	>=30	2587/ 2996	1.08 (0.97-1.20)		
rs1011970	Age at first birth (among parous, years/5)	combined	16036/21664		1.00	8.6E-01
rs1011970	Ever breastfed (among parous, yes/no)	no	2118/ 2303	1.10 (0.98-1.24)		
rs1011970	Ever breastfed (among parous, yes/no)	yes	7201/ 9425	1.09 (1.03-1.16)		
rs1011970	Ever breastfed (among parous, yes/no)	combined	9319/11728		0.99	8.8E-01
rs1011970	Usual adult BMI, age<54	<25	2917/ 2725	1.09 (0.99-1.21)		
rs1011970	Usual adult BMI, age<54	25-<30	938/ 880	1.08 (0.90-1.29)		
rs1011970	Usual adult BMI, age<54	>=30	422/ 417	1.26 (0.97-1.64)		
rs1011970	Usual adult BMI (BMI/5), age<54	combined	4277/ 4022		1.03	5.5E-01
rs1011970	Usual adult BMI, age>=54	<25	3992/ 5944	1.14 (1.06-1.23)		
rs1011970	Usual adult BMI, age>=54	25-<30	1817/ 2490	0.99 (0.88-1.12)		
rs1011970	Usual adult BMI, age>=54	>=30	663/ 811	0.94 (0.78-1.15)		
rs1011970	Usual adult BMI (BMI/5), age>=54	combined	6472/ 9245		0.94	1.1E-01
rs1011970	Usual adult height (cm)	<160	4091/ 4896	0.99 (0.91-1.07)		
rs1011970	Usual adult height (cm)	160-<165	5006/ 5901	1.17 (1.09-1.26)		
rs1011970	Usual adult height (cm)	165-<170	4354/ 4857	1.10 (1.01-1.19)		
rs1011970	Usual adult height (cm)	>=170	3127/ 3206	1.07 (0.97-1.18)		
rs1011970	Usual adult height (cm/5)	combined	16578/18860		1.01	4.1E-01
rs1011970	Ever use of oral contraceptives	no	6526/ 7376	1.05 (0.99-1.13)		
rs1011970	Ever use of oral contraceptives	yes	7876/10197	1.12 (1.06-1.18)		
rs1011970	Ever use of oral contraceptives (yes/no)	combined	14402/17573		1.06	2.0E-01
rs1011970	Duration of oral contraceptive use (years)	0	6526/ 7376	1.05 (0.99-1.13)		
rs1011970	Duration of oral contraceptive use (years)	>0-<5	2624/ 3384	1.11 (1.01-1.22)		
rs1011970	Duration of oral contraceptive use (years)	5-<10	1926/ 2484	1.08 (0.97-1.22)		
rs1011970	Duration of oral contraceptive use (years)	>=10	3125/ 4047	1.15 (1.05-1.26)		
rs1011970	Duration of oral contraceptive use (years/5)	combined	14201/17291		1.02	1.6E-01
rs1011970	Current use of combined estrogen/ progestagen MHT	never	2594/ 4067	1.13 (1.03-1.25)		
rs1011970	Current use of combined estrogen/ progestagen MHT	EPCurrent	1084/ 1186	1.05 (0.90-1.23)		
rs1011970	Current use of combined estrogen/ progestagen MHT (yes/no)	combined	5331/ 8108		0.95	5.5E-01
rs1011970	Current use of estrogen only MHT	never	2736/ 4138	1.12 (1.02-1.23)		
rs1011970	Current use of estrogen only MHT	ECurrent	616/ 916	1.05 (0.86-1.27)		
rs1011970	Current use of estrogen only MHT (yes/no)	combined	5583/ 8272		0.96	6.9E-01

SNP	Variable	Stratum	N (cases/ controls)	OR (95% CI)[1]	OR int[2]	P int[3]
rs1011970	Duration of combined estrogen/progestagen MHT among current users (years)	never	2594/ 4067	1.13 (1.03-1.25)		
rs1011970	Duration of combined estrogen/progestagen MHT among current users (years)	>0-<5	240/ 314	1.19 (0.86-1.64)		
rs1011970	Duration of combined estrogen/progestagen MHT among current users (years)	5-<10	347/ 352	1.23 (0.92-1.63)		
rs1011970	Duration of combined estrogen/progestagen MHT among current users (years)	>=10	433/ 462	0.87 (0.68-1.12)		
rs1011970	Duration of combined estrogen/progestagen MHT among current users (years/5)	combined	5244/ 8013		0.94	1.1E-01
rs1011970	Duration of estrogen only MHT among current users (years)	never	2736/ 4138	1.12 (1.02-1.23)		
rs1011970	Duration of estrogen only MHT among current users (years)	>0-<5	155/ 279	1.01 (0.69-1.48)		
rs1011970	Duration of estrogen only MHT among current users (years)	5-<10	185/ 254	1.28 (0.87-1.87)		
rs1011970	Duration of estrogen only MHT among current users (years)	>=10	259/ 360	0.96 (0.71-1.30)		
rs1011970	Duration of estrogen only MHT among current users (years/5)	combined	5534/ 8197		0.97	5.2E-01
rs1011970	Mean lifetime intake of alcohol (g/day)	0	2067/ 2803	1.15 (1.02-1.28)		
rs1011970	Mean lifetime intake of alcohol (g/day)	>0-<10	2864/ 4654	1.15 (1.05-1.25)		
rs1011970	Mean lifetime intake of alcohol (g/day)	>=10-<20	578/ 1059	1.08 (0.89-1.31)		
rs1011970	Mean lifetime intake of alcohol (g/day)	>=20	482/ 741	1.08 (0.86-1.34)		
rs1011970	Mean lifetime intake of alcohol (10g/day)	combined	5991/ 9257		0.98	3.3E-01
rs1011970	Smoking (ever)	no	8610/ 9654	1.12 (1.06-1.19)		
rs1011970	Smoking (ever)	yes	6928/ 7798	1.05 (0.98-1.11)		
rs1011970	Smoking (ever/never)	combined	15538/17452		0.93	9.3E-02
rs1011970	Smoking (pack-years)	0	7675/ 8476	1.13 (1.07-1.20)		
rs1011970	Smoking (pack-years)	0-<10	2600/ 3379	1.12 (1.01-1.24)		
rs1011970	Smoking (pack-years)	10-<20	1243/ 1477	1.01 (0.88-1.16)		
rs1011970	Smoking (pack-years)	>=20	1696/ 1849	1.00 (0.88-1.13)		
rs1011970	Smoking (pack-years/10)	combined	13214/15181		0.96	1.5E-02
rs1011970	Physical activity during year before reference date (h/week)	0	643/ 680	1.25 (1.02-1.53)		
rs1011970	Physical activity during year before reference date (h/week)	0-<3.5	1950/ 2413	1.11 (0.99-1.24)		
rs1011970	Physical activity during year before reference date (h/week)	3.5-<7	1348/ 1962	0.99 (0.87-1.13)		
rs1011970	Physical activity during year before reference date (h/week)	>=7	1609/ 3343	1.15 (1.03-1.28)		
rs1011970	Physical activity during year before reference date (square root of h/week)	combined	5550/ 8398		0.99	7.0E-01
rs10771399[6]	Age at menarche (years)	<=11	2687/ 2111	0.75 (0.66-0.86)		
rs10771399	Age at menarche (years)	12-13	7565/ 5902	0.88 (0.81-0.95)		
rs10771399	Age at menarche (years)	>=14	6281/ 5310	0.85 (0.77-0.92)		
rs10771399	Age at menarche (years/2)	combined	16533/13323		1.02	6.2E-01
rs10771399	Parous	no	3016/ 2187	0.75 (0.66-0.85)		
rs10771399	Parous	yes	17312/13944	0.86 (0.81-0.90)		
rs10771399	Parous (yes/no)	combined	20328/16131		1.15	5.7E-02
rs10771399	Number of births (among parous)	1	3523/ 2515	0.94 (0.83-1.06)		
rs10771399	Number of births (among parous)	2	7826/ 5872	0.83 (0.77-0.90)		
rs10771399	Number of births (among parous)	3	3738/ 3033	0.86 (0.77-0.96)		
rs10771399	Number of births (among parous)	>=4	1831/ 1720	0.84 (0.72-0.98)		
rs10771399	Number of births (among parous)	combined	16918/13140		0.99	6.3E-01
rs10771399	Age at first birth (among parous, years)	<20	1871/ 1162	0.86 (0.72-1.02)		
rs10771399	Age at first birth (among parous, years)	20-24	6028/ 5115	0.87 (0.79-0.94)		
rs10771399	Age at first birth (among parous, years)	25-29	4443/ 3796	0.84 (0.76-0.94)		
rs10771399	Age at first birth (among parous, years)	>=30	2164/ 1567	0.90 (0.77-1.05)		
rs10771399	Age at first birth (among parous, years/5)	combined	14506/11640		1.03	4.3E-01
rs10771399	Ever breastfed (among parous, yes/no)	no	1261/ 1045	0.80 (0.66-0.96)		

SNP	Variable	Stratum	N (cases/ controls)	OR (95% CI)[1]	OR int[2]	P int[3]
rs10771399	Ever breastfed (among parous, yes/no)	yes	4639/ 4980	0.84 (0.77-0.93)		
rs10771399	Ever breastfed (among parous, yes/no)	combined	5900/ 6025		1.06	6.1E-01
rs10771399	Usual adult BMI, age<54	<25	1688/ 1232	0.75 (0.63-0.89)		
rs10771399	Usual adult BMI, age<54	25-<30	578/ 468	0.98 (0.73-1.32)		
rs10771399	Usual adult BMI, age<54	>=30	270/ 275	0.51 (0.33-0.77)		
rs10771399	Usual adult BMI (BMI/5), age<54	combined	2536/ 1975		0.94	4.5E-01
rs10771399	Usual adult BMI, age>=54	<25	2570/ 2851	0.83 (0.73-0.93)		
rs10771399	Usual adult BMI, age>=54	25-<30	1278/ 1409	0.90 (0.75-1.07)		
rs10771399	Usual adult BMI, age>=54	>=30	487/ 558	0.77 (0.57-1.03)		
rs10771399	Usual adult BMI (BMI/5), age>=54	combined	4335/ 4818		1.02	7.2E-01
rs10771399	Usual adult height (cm)	<160	2456/ 2834	0.84 (0.74-0.95)		
rs10771399	Usual adult height (cm)	160-<165	2971/ 3310	0.82 (0.73-0.92)		
rs10771399	Usual adult height (cm)	165-<170	2664/ 2748	0.88 (0.77-0.99)		
rs10771399	Usual adult height (cm)	>=170	1922/ 1816	0.81 (0.70-0.94)		
rs10771399	Usual adult height (cm/5)	combined	10013/10708		1.00	1.0E+00
rs10771399	Ever use of oral contraceptives	no	3959/ 3904	0.86 (0.78-0.95)		
rs10771399	Ever use of oral contraceptives	yes	5060/ 6211	0.85 (0.78-0.93)		
rs10771399	Ever use of oral contraceptives (yes/no)	combined	9019/10115		0.99	8.7E-01
rs10771399	Duration of oral contraceptive use (years)	0	3959/ 3904	0.86 (0.78-0.95)		
rs10771399	Duration of oral contraceptive use (years)	>0-<5	1659/ 2097	0.87 (0.75-1.01)		
rs10771399	Duration of oral contraceptive use (years)	5-<10	1245/ 1531	0.88 (0.74-1.04)		
rs10771399	Duration of oral contraceptive use (years)	>=10	2019/ 2384	0.82 (0.72-0.94)		
rs10771399	Duration of oral contraceptive use (years/5)	combined	8882/ 9916		0.97	2.7E-01
rs10771399	Current use of combined estrogen/ progestagen MHT	never	1675/ 1972	0.77 (0.66-0.89)		
rs10771399	Current use of combined estrogen/ progestagen MHT	EPCurrent	746/ 603	0.96 (0.75-1.21)		
rs10771399	Current use of combined estrogen/ progestagen MHT (yes/no)	combined	3601/ 3929		1.15	2.9E-01
rs10771399	Current use of estrogen only MHT	never	1805/ 2010	0.77 (0.67-0.89)		
rs10771399	Current use of estrogen only MHT	ECurrent	499/ 488	1.00 (0.76-1.32)		
rs10771399	Current use of estrogen only MHT (yes/no)	combined	3833/ 4031		1.19	2.4E-01
rs10771399	Duration of combined estrogen/progestagen MHT among current users (years)	never	1675/ 1972	0.77 (0.66-0.89)		
rs10771399	Duration of combined estrogen/progestagen MHT among current users (years)	>0-<5	161/ 168	1.22 (0.73-2.03)		
rs10771399	Duration of combined estrogen/progestagen MHT among current users (years)	5-<10	234/ 176	0.70 (0.47-1.06)		
rs10771399	Duration of combined estrogen/progestagen MHT among current users (years)	>=10	291/ 209	1.03 (0.69-1.52)		
rs10771399	Duration of combined estrogen/progestagen MHT among current users (years/5)	combined	3520/ 3851		1.05	4.9E-01
rs10771399	Duration of estrogen only MHT among current users (years)	never	1805/ 2010	0.77 (0.67-0.89)		
rs10771399	Duration of estrogen only MHT among current users (years)	>0-<5	124/ 150	0.83 (0.49-1.42)		
rs10771399	Duration of estrogen only MHT among current users (years)	5-<10	161/ 141	0.81 (0.50-1.31)		
rs10771399	Duration of estrogen only MHT among current users (years)	>=10	198/ 181	1.55 (0.95-2.51)		
rs10771399	Duration of estrogen only MHT among current users (years/5)	combined	3791/ 3974		1.16	4.2E-02
rs10771399	Mean lifetime intake of alcohol (g/day)	0	950/ 1072	0.83 (0.68-1.02)		
rs10771399	Mean lifetime intake of alcohol (g/day)	>0-<10	1750/ 1926	0.91 (0.79-1.06)		
rs10771399	Mean lifetime intake of alcohol (g/day)	>=10-<20	348/ 417	0.69 (0.49-0.98)		
rs10771399	Mean lifetime intake of alcohol (g/day)	>=20	291/ 313	0.71 (0.50-1.00)		
rs10771399	Mean lifetime intake of alcohol (10g/day)	combined	3339/ 3728		0.96	2.7E-01
rs10771399	Smoking (ever)	no	5250/ 5606	0.85 (0.78-0.93)		
rs10771399	Smoking (ever)	yes	4098/ 4071	0.82 (0.74-0.91)		
rs10771399	Smoking (ever/never)	combined	9348/ 9677		0.96	5.7E-01

SNP	Variable	Stratum	N (cases/ controls)	OR (95% CI)[1]	OR int[2]	P int[3]
rs10771399	Smoking (pack-years)	0	4332/ 4483	0.86 (0.78-0.95)		
rs10771399	Smoking (pack-years)	0-<10	1417/ 1589	0.86 (0.72-1.02)		
rs10771399	Smoking (pack-years)	10-<20	672/ 689	0.86 (0.67-1.11)		
rs10771399	Smoking (pack-years)	>=20	921/ 891	0.75 (0.60-0.93)		
rs10771399	Smoking (pack-years/10)	combined	7342/ 7652		0.98	4.5E-01
rs10771399	Physical activity during year before reference date (h/week)	0	595/ 595	0.84 (0.64-1.10)		
rs10771399	Physical activity during year before reference date (h/week)	0-<3.5	1441/ 1607	0.85 (0.73-1.00)		
rs10771399	Physical activity during year before reference date (h/week)	3.5-<7	966/ 1113	0.83 (0.69-1.00)		
rs10771399	Physical activity during year before reference date (h/week)	>=7	1207/ 1290	0.89 (0.75-1.06)		
rs10771399	Physical activity during year before reference date (square root of h/week)	combined	4209/ 4605		1.03	5.1E-01
rs10941679	Age at menarche (years)	<=11	4086/ 3760	1.18 (1.10-1.27)		
rs10941679	Age at menarche (years)	12-13	11587/12457	1.14 (1.09-1.19)		
rs10941679	Age at menarche (years)	>=14	9662/12787	1.12 (1.07-1.17)		
rs10941679	Age at menarche (years/2)	combined	25335/29004		0.98	1.9E-01
rs10941679	Parous	no	4377/ 4299	1.11 (1.04-1.19)		
rs10941679	Parous	yes	24418/28290	1.13 (1.10-1.16)		
rs10941679	Parous (yes/no)	combined	28795/32589		1.02	6.7E-01
rs10941679	Number of births (among parous)	1	5266/ 5763	1.13 (1.06-1.20)		
rs10941679	Number of births (among parous)	2	11057/13017	1.14 (1.09-1.19)		
rs10941679	Number of births (among parous)	3	5081/ 5783	1.13 (1.06-1.20)		
rs10941679	Number of births (among parous)	>=4	2565/ 2883	1.11 (1.01-1.21)		
rs10941679	Number of births (among parous)	combined	23969/27446		1.00	7.4E-01
rs10941679	Age at first birth (among parous, years)	<20	2711/ 2834	1.09 (1.00-1.19)		
rs10941679	Age at first birth (among parous, years)	20-24	9223/11195	1.15 (1.10-1.20)		
rs10941679	Age at first birth (among parous, years)	25-29	6789/ 8275	1.18 (1.12-1.25)		
rs10941679	Age at first birth (among parous, years)	>=30	3287/ 3551	1.07 (0.99-1.16)		
rs10941679	Age at first birth (among parous, years/5)	combined	22010/25855		1.00	9.7E-01
rs10941679	Ever breastfed (among parous, yes/no)	no	2777/ 2886	1.08 (0.99-1.18)		
rs10941679	Ever breastfed (among parous, yes/no)	yes	8217/10347	1.12 (1.07-1.18)		
rs10941679	Ever breastfed (among parous, yes/no)	combined	10994/13233		1.04	4.8E-01
rs10941679	Usual adult BMI, age<54	<25	3418/ 3124	1.09 (1.01-1.18)		
rs10941679	Usual adult BMI, age<54	25-<30	1150/ 1055	1.03 (0.89-1.18)		
rs10941679	Usual adult BMI, age<54	>=30	538/ 521	1.24 (1.01-1.51)		
rs10941679	Usual adult BMI (BMI/5), age<54	combined	5106/ 4700		1.03	4.8E-01
rs10941679	Usual adult BMI, age>=54	<25	4494/ 6461	1.11 (1.04-1.18)		
rs10941679	Usual adult BMI, age>=54	25-<30	2089/ 2735	1.18 (1.07-1.29)		
rs10941679	Usual adult BMI, age>=54	>=30	847/ 942	1.15 (0.98-1.35)		
rs10941679	Usual adult BMI (BMI/5), age>=54	combined	7430/10138		1.03	2.8E-01
rs10941679	Usual adult height (cm)	<160	4494/ 5328	1.08 (1.01-1.15)		
rs10941679	Usual adult height (cm)	160-<165	5613/ 6388	1.13 (1.06-1.19)		
rs10941679	Usual adult height (cm)	165-<170	4780/ 5263	1.17 (1.09-1.24)		
rs10941679	Usual adult height (cm)	>=170	3525/ 3480	1.08 (1.00-1.16)		
rs10941679	Usual adult height (cm/5)	combined	18412/20459		1.00	9.8E-01
rs10941679	Ever use of oral contraceptives	no	7491/ 8189	1.11 (1.05-1.17)		
rs10941679	Ever use of oral contraceptives	yes	8934/10716	1.11 (1.06-1.16)		
rs10941679	Ever use of oral contraceptives (yes/no)	combined	16425/18905		1.00	9.6E-01
rs10941679	Duration of oral contraceptive use (years)	0	7491/ 8189	1.11 (1.05-1.17)		
rs10941679	Duration of oral contraceptive use (years)	>0-<5	3095/ 3540	1.17 (1.08-1.27)		
rs10941679	Duration of oral contraceptive use (years)	5-<10	2180/ 2644	1.02 (0.93-1.12)		

SNP	Variable	Stratum	N (cases/ controls)	OR (95% CI)[1]	OR int[2]	P int[3]
rs10941679	Duration of oral contraceptive use (years)	>=10	3440/ 4247	1.11 (1.03-1.19)		
rs10941679	Duration of oral contraceptive use (years/5)	combined	16206/18620		1.00	9.9E-01
rs10941679	Current use of combined estrogen/ progestagen MHT	never	3015/ 4486	1.12 (1.04-1.21)		
rs10941679	Current use of combined estrogen/ progestagen MHT	EPCurrent	1324/ 1365	1.14 (1.00-1.29)		
rs10941679	Current use of combined estrogen/ progestagen MHT (yes/no)	combined	6253/ 8973		1.01	8.7E-01
rs10941679	Current use of estrogen only MHT	never	3156/ 4568	1.12 (1.04-1.21)		
rs10941679	Current use of estrogen only MHT	ECurrent	758/ 1055	1.15 (0.98-1.34)		
rs10941679	Current use of estrogen only MHT (yes/no)	combined	6504/ 9164		1.02	8.3E-01
rs10941679	Duration of combined estrogen/progestagen MHT among current users (years)	never	3015/ 4486	1.12 (1.04-1.21)		
rs10941679	Duration of combined estrogen/progestagen MHT among current users (years)	>0-<5	295/ 373	1.21 (0.94-1.55)		
rs10941679	Duration of combined estrogen/progestagen MHT among current users (years)	5-<10	440/ 411	1.13 (0.91-1.41)		
rs10941679	Duration of combined estrogen/progestagen MHT among current users (years)	>=10	525/ 523	1.09 (0.89-1.33)		
rs10941679	Duration of combined estrogen/progestagen MHT among current users (years/5)	combined	6165/ 8878		0.99	7.1E-01
rs10941679	Duration of estrogen only MHT among current users (years)	never	3156/ 4568	1.12 (1.04-1.21)		
rs10941679	Duration of estrogen only MHT among current users (years)	>0-<5	170/ 298	1.15 (0.85-1.56)		
rs10941679	Duration of estrogen only MHT among current users (years)	5-<10	208/ 286	1.09 (0.81-1.46)		
rs10941679	Duration of estrogen only MHT among current users (years)	>=10	344/ 432	1.18 (0.93-1.50)		
rs10941679	Duration of estrogen only MHT among current users (years/5)	combined	6418/ 9047		1.00	9.2E-01
rs10941679	Mean lifetime intake of alcohol (g/day)	0	2336/ 3017	1.13 (1.03-1.23)		
rs10941679	Mean lifetime intake of alcohol (g/day)	>0-<10	3109/ 4933	1.08 (1.01-1.16)		
rs10941679	Mean lifetime intake of alcohol (g/day)	>=10-<20	646/ 1128	1.09 (0.93-1.28)		
rs10941679	Mean lifetime intake of alcohol (g/day)	>=20	525/ 801	1.12 (0.94-1.34)		
rs10941679	Mean lifetime intake of alcohol (10g/day)	combined	6616/ 9879		1.00	9.3E-01
rs10941679	Smoking (ever)	no	9324/10276	1.14 (1.08-1.19)		
rs10941679	Smoking (ever)	yes	7832/ 8592	1.11 (1.06-1.17)		
rs10941679	Smoking (ever/never)	combined	17156/18868		0.98	5.2E-01
rs10941679	Smoking (pack-years)	0	8387/ 9154	1.12 (1.07-1.18)		
rs10941679	Smoking (pack-years)	0-<10	3024/ 3682	1.15 (1.06-1.25)		
rs10941679	Smoking (pack-years)	10-<20	1442/ 1652	1.04 (0.92-1.17)		
rs10941679	Smoking (pack-years)	>=20	2068/ 2162	1.08 (0.98-1.20)		
rs10941679	Smoking (pack-years/10)	combined	14921/16650		0.98	1.8E-01
rs10941679	Physical activity during year before reference date (h/week)	0	1098/ 1068	1.26 (1.10-1.45)		
rs10941679	Physical activity during year before reference date (h/week)	0-<3.5	2352/ 2758	1.11 (1.02-1.22)		
rs10941679	Physical activity during year before reference date (h/week)	3.5-<7	1607/ 2183	1.15 (1.04-1.28)		
rs10941679	Physical activity during year before reference date (h/week)	>=7	1864/ 3684	1.07 (0.97-1.17)		
rs10941679	Physical activity during year before reference date (square root of h/week)	combined	6921/ 9693		0.98	2.6E-01
rs10995190	Age at menarche (years)	<=11	2584/ 2927	0.92 (0.83-1.03)		
rs10995190	Age at menarche (years)	12-13	8563/10817	0.87 (0.82-0.92)		
rs10995190	Age at menarche (years)	>=14	7474/10884	0.89 (0.83-0.94)		
rs10995190	Age at menarche (years/2)	combined	18621/24628		0.98	4.7E-01
rs10995190	Parous	no	3566/ 3814	0.88 (0.80-0.96)		
rs10995190	Parous	yes	18397/24215	0.89 (0.85-0.92)		
rs10995190	Parous (yes/no)	combined	21963/28029		1.01	8.4E-01
rs10995190	Number of births (among parous)	1	4303/ 5150	0.90 (0.82-0.97)		
rs10995190	Number of births (among parous)	2	8036/11034	0.85 (0.81-0.91)		
rs10995190	Number of births (among parous)	3	3683/ 4831	0.91 (0.83-0.99)		

SNP	Variable	Stratum	N (cases/ controls)	OR (95% CI)[1]	OR int[2]	P int[3]
rs10995190	Number of births (among parous)	>=4	1881/ 2327	0.96 (0.85-1.09)		
rs10995190	Number of births (among parous)	combined	17903/23342		1.02	2.8E-01
rs10995190	Age at first birth (among parous, years)	<20	1720/ 2483	0.85 (0.75-0.96)		
rs10995190	Age at first birth (among parous, years)	20-24	6546/ 9312	0.94 (0.88-1.00)		
rs10995190	Age at first birth (among parous, years)	25-29	5182/ 6920	0.88 (0.81-0.94)		
rs10995190	Age at first birth (among parous, years)	>=30	2589/ 3005	0.85 (0.76-0.95)		
rs10995190	Age at first birth (among parous, years/5)	combined	16037/21720		0.98	3.0E-01
rs10995190	Ever breastfed (among parous, yes/no)	no	2113/ 2302	0.83 (0.73-0.93)		
rs10995190	Ever breastfed (among parous, yes/no)	yes	7201/ 9423	0.92 (0.86-0.98)		
rs10995190	Ever breastfed (among parous, yes/no)	combined	9314/11725		1.11	1.2E-01
rs10995190	Usual adult BMI, age<54	<25	2912/ 2728	0.87 (0.78-0.96)		
rs10995190	Usual adult BMI, age<54	25-<30	937/ 881	0.90 (0.75-1.08)		
rs10995190	Usual adult BMI, age<54	>=30	422/ 417	1.03 (0.80-1.33)		
rs10995190	Usual adult BMI (BMI/5), age<54	combined	4271/ 4026		1.01	8.3E-01
rs10995190	Usual adult BMI, age>=54	<25	3993/ 5945	0.91 (0.84-0.99)		
rs10995190	Usual adult BMI, age>=54	25-<30	1812/ 2487	0.85 (0.75-0.96)		
rs10995190	Usual adult BMI, age>=54	>=30	662/ 811	0.79 (0.63-0.98)		
rs10995190	Usual adult BMI (BMI/5), age>=54	combined	6467/ 9243		0.98	6.5E-01
rs10995190	Usual adult height (cm)	<160	4088/ 4897	0.85 (0.78-0.93)		
rs10995190	Usual adult height (cm)	160-<165	5004/ 5900	0.89 (0.82-0.96)		
rs10995190	Usual adult height (cm)	165-<170	4351/ 4855	0.87 (0.80-0.94)		
rs10995190	Usual adult height (cm)	>=170	3125/ 3207	0.93 (0.84-1.03)		
rs10995190	Usual adult height (cm/5)	combined	16568/18859		1.02	3.2E-01
rs10995190	Ever use of oral contraceptives	no	6520/ 7379	0.83 (0.78-0.89)		
rs10995190	Ever use of oral contraceptives	yes	7876/10198	0.92 (0.87-0.98)		
rs10995190	Ever use of oral contraceptives (yes/no)	combined	14396/17577		1.11	1.6E-02
rs10995190	Duration of oral contraceptive use (years)	0	6520/ 7379	0.83 (0.78-0.89)		
rs10995190	Duration of oral contraceptive use (years)	>0-<5	2628/ 3383	0.97 (0.88-1.08)		
rs10995190	Duration of oral contraceptive use (years)	5-<10	1921/ 2482	0.87 (0.77-0.99)		
rs10995190	Duration of oral contraceptive use (years)	>=10	3129/ 4051	0.91 (0.82-1.00)		
rs10995190	Duration of oral contraceptive use (years/5)	combined	14198/17295		1.02	3.3E-01
rs10995190	Current use of combined estrogen/ progestagen MHT	never	2590/ 4070	0.86 (0.78-0.94)		
rs10995190	Current use of combined estrogen/ progestagen MHT	EPCurrent	1088/ 1189	0.77 (0.66-0.91)		
rs10995190	Current use of combined estrogen/ progestagen MHT (yes/no)	combined	5330/ 8113		0.88	1.7E-01
rs10995190	Current use of estrogen only MHT	never	2730/ 4140	0.85 (0.78-0.94)		
rs10995190	Current use of estrogen only MHT	ECurrent	616/ 918	0.85 (0.69-1.04)		
rs10995190	Current use of estrogen only MHT (yes/no)	combined	5580/ 8275		0.97	7.7E-01
rs10995190	Duration of combined estrogen/progestagen MHT among current users (years)	never	2590/ 4070	0.86 (0.78-0.94)		
rs10995190	Duration of combined estrogen/progestagen MHT among current users (years)	>0-<5	241/ 314	0.88 (0.63-1.21)		
rs10995190	Duration of combined estrogen/progestagen MHT among current users (years)	5-<10	349/ 353	0.72 (0.53-0.96)		
rs10995190	Duration of combined estrogen/progestagen MHT among current users (years)	>=10	434/ 464	0.78 (0.60-1.01)		
rs10995190	Duration of combined estrogen/progestagen MHT among current users (years/5)	combined	5243/ 8018		0.95	2.5E-01
rs10995190	Duration of estrogen only MHT among current users (years)	never	2730/ 4140	0.86 (0.78-0.94)		
rs10995190	Duration of estrogen only MHT among current users (years)	>0-<5	155/ 280	0.79 (0.54-1.18)		
rs10995190	Duration of estrogen only MHT among current users (years)	5-<10	185/ 255	0.92 (0.64-1.33)		
rs10995190	Duration of estrogen only MHT among current users (years)	>=10	259/ 360	0.82 (0.59-1.13)		
rs10995190	Duration of estrogen only MHT among current users (years/5)	combined	5531/ 8200		0.98	6.4E-01

SNP	Variable	Stratum	N (cases/ controls)	OR (95% CI)[1]	OR int[2]	P int[3]
rs10995190	Mean lifetime intake of alcohol (g/day)	0	2061/ 2798	0.78 (0.70-0.87)		
rs10995190	Mean lifetime intake of alcohol (g/day)	>0-<10	2863/ 4659	0.90 (0.82-0.98)		
rs10995190	Mean lifetime intake of alcohol (g/day)	>=10-<20	577/ 1059	0.86 (0.70-1.06)		
rs10995190	Mean lifetime intake of alcohol (g/day)	>=20	484/ 740	0.96 (0.76-1.21)		
rs10995190	Mean lifetime intake of alcohol (10g/day)	combined	5985/ 9256		1.05	6.2E-02
rs10995190	Smoking (ever)	no	8609/ 9654	0.89 (0.84-0.94)		
rs10995190	Smoking (ever)	yes	6917/ 7795	0.86 (0.80-0.91)		
rs10995190	Smoking (ever/never)	combined	15526/17449		0.96	4.2E-01
rs10995190	Smoking (pack-years)	0	7675/ 8475	0.89 (0.84-0.95)		
rs10995190	Smoking (pack-years)	0-<10	2596/ 3374	0.90 (0.81-0.99)		
rs10995190	Smoking (pack-years)	10-<20	1236/ 1472	0.90 (0.78-1.05)		
rs10995190	Smoking (pack-years)	>=20	1696/ 1854	0.84 (0.74-0.96)		
rs10995190	Smoking (pack-years/10)	combined	13203/15175		0.99	6.2E-01
rs10995190	Physical activity during year before reference date (h/week)	0	643/ 679	0.97 (0.78-1.20)		
rs10995190	Physical activity during year before reference date (h/week)	0-<3.5	1950/ 2417	0.87 (0.77-0.97)		
rs10995190	Physical activity during year before reference date (h/week)	3.5-<7	1348/ 1969	0.89 (0.77-1.02)		
rs10995190	Physical activity during year before reference date (h/week)	>=7	1610/ 3342	0.90 (0.79-1.01)		
rs10995190	Physical activity during year before reference date (square root of h/week)	combined	5551/ 8407		1.00	9.6E-01
rs11249433	Age at menarche (years)	<=11	4017/ 3510	1.10 (1.03-1.18)		
rs11249433	Age at menarche (years)	12-13	11315/11480	1.14 (1.10-1.19)		
rs11249433	Age at menarche (years)	>=14	9134/11182	1.07 (1.03-1.12)		
rs11249433	Age at menarche (years/2)	combined	24466/26172		0.97	5.9E-02
rs11249433	Parous	no	4243/ 3796	0.98 (0.92-1.05)		
rs11249433	Parous	yes	24226/25432	1.14 (1.11-1.17)		
rs11249433	Parous (yes/no)	combined	28469/29228		1.16	5.3E-05
rs11249433	Number of births (among parous)	1	5107/ 4880	1.13 (1.06-1.20)		
rs11249433	Number of births (among parous)	2	10798/11511	1.15 (1.11-1.20)		
rs11249433	Number of births (among parous)	3	4988/ 5187	1.14 (1.08-1.21)		
rs11249433	Number of births (among parous)	>=4	2517/ 2605	1.06 (0.98-1.15)		
rs11249433	Number of births (among parous)	combined	23410/24183		0.99	4.0E-01
rs11249433	Age at first birth (among parous, years)	<20	2586/ 2412	1.13 (1.04-1.23)		
rs11249433	Age at first birth (among parous, years)	20-24	8810/ 9784	1.15 (1.10-1.20)		
rs11249433	Age at first birth (among parous, years)	25-29	6554/ 7415	1.11 (1.05-1.16)		
rs11249433	Age at first birth (among parous, years)	>=30	3219/ 3266	1.15 (1.07-1.23)		
rs11249433	Age at first birth (among parous, years/5)	combined	21169/22877		1.00	9.1E-01
rs11249433	Ever breastfed (among parous, yes/no)	no	2590/ 2254	1.14 (1.05-1.24)		
rs11249433	Ever breastfed (among parous, yes/no)	yes	7596/ 7952	1.12 (1.07-1.17)		
rs11249433	Ever breastfed (among parous, yes/no)	combined	10186/10206		0.98	6.4E-01
rs11249433	Usual adult BMI, age<54	<25	3370/ 2863	1.10 (1.02-1.18)		
rs11249433	Usual adult BMI, age<54	25-<30	1144/ 993	1.13 (0.99-1.27)		
rs11249433	Usual adult BMI, age<54	>=30	541/ 501	1.00 (0.83-1.19)		
rs11249433	Usual adult BMI (BMI/5), age<54	combined	5055/ 4357		0.97	4.0E-01
rs11249433	Usual adult BMI, age>=54	<25	3789/ 4064	1.07 (1.01-1.15)		
rs11249433	Usual adult BMI, age>=54	25-<30	1921/ 2098	1.15 (1.05-1.26)		
rs11249433	Usual adult BMI, age>=54	>=30	829/ 840	1.11 (0.96-1.28)		
rs11249433	Usual adult BMI (BMI/5), age>=54	combined	6539/ 7002		1.00	9.0E-01
rs11249433	Usual adult height (cm)	<160	4218/ 4519	1.10 (1.04-1.17)		
rs11249433	Usual adult height (cm)	160-<165	5182/ 5243	1.13 (1.07-1.19)		
rs11249433	Usual adult height (cm)	165-<170	4422/ 4167	1.10 (1.03-1.17)		

SNP	Variable	Stratum	N (cases/ controls)	OR (95% CI)[1]	OR int[2]	P int[3]
rs11249433	Usual adult height (cm)	>=170	3206/ 2739	1.05 (0.97-1.13)		
rs11249433	Usual adult height (cm/5)	combined	17028/16668		0.99	4.2E-01
rs11249433	Ever use of oral contraceptives	no	6915/ 6821	1.11 (1.06-1.16)		
rs11249433	Ever use of oral contraceptives	yes	8159/ 8286	1.08 (1.04-1.13)		
rs11249433	Ever use of oral contraceptives (yes/no)	combined	15074/15107		0.98	4.7E-01
rs11249433	Duration of oral contraceptive use (years)	0	6915/ 6821	1.11 (1.06-1.16)		
rs11249433	Duration of oral contraceptive use (years)	>0-<5	2867/ 2821	1.08 (1.00-1.17)		
rs11249433	Duration of oral contraceptive use (years)	5-<10	2011/ 2053	1.05 (0.96-1.14)		
rs11249433	Duration of oral contraceptive use (years)	>=10	3074/ 3145	1.10 (1.02-1.18)		
rs11249433	Duration of oral contraceptive use (years/5)	combined	14867/14840		1.00	9.0E-01
rs11249433	Current use of combined estrogen/ progestagen MHT	never	2688/ 3247	1.07 (0.99-1.15)		
rs11249433	Current use of combined estrogen/ progestagen MHT	EPCurrent	1043/ 769	1.16 (1.02-1.33)		
rs11249433	Current use of combined estrogen/ progestagen MHT (yes/no)	combined	5343/ 5801		1.10	2.0E-01
rs11249433	Current use of estrogen only MHT	never	2832/ 3329	1.06 (0.99-1.14)		
rs11249433	Current use of estrogen only MHT	ECurrent	686/ 648	1.12 (0.96-1.30)		
rs11249433	Current use of estrogen only MHT (yes/no)	combined	5602/ 5988		1.04	6.1E-01
rs11249433	Duration of combined estrogen/progestagen MHT among current users (years)	never	2688/ 3247	1.07 (0.99-1.15)		
rs11249433	Duration of combined estrogen/progestagen MHT among current users (years)	>0-<5	246/ 245	1.20 (0.94-1.55)		
rs11249433	Duration of combined estrogen/progestagen MHT among current users (years)	5-<10	353/ 230	1.21 (0.95-1.54)		
rs11249433	Duration of combined estrogen/progestagen MHT among current users (years)	>=10	380/ 243	1.09 (0.87-1.38)		
rs11249433	Duration of combined estrogen/progestagen MHT among current users (years/5)	combined	5255/ 5717		1.03	4.5E-01
rs11249433	Duration of estrogen only MHT among current users (years)	never	2832/ 3329	1.06 (0.99-1.14)		
rs11249433	Duration of estrogen only MHT among current users (years)	>0-<5	155/ 180	1.10 (0.80-1.50)		
rs11249433	Duration of estrogen only MHT among current users (years)	5-<10	186/ 175	0.93 (0.70-1.24)		
rs11249433	Duration of estrogen only MHT among current users (years)	>=10	309/ 259	1.24 (0.98-1.57)		
rs11249433	Duration of estrogen only MHT among current users (years/5)	combined	5516/ 5875		1.03	4.5E-01
rs11249433	Mean lifetime intake of alcohol (g/day)	0	2126/ 2523	1.09 (1.00-1.19)		
rs11249433	Mean lifetime intake of alcohol (g/day)	>0-<10	2548/ 2858	1.10 (1.02-1.19)		
rs11249433	Mean lifetime intake of alcohol (g/day)	>=10-<20	535/ 590	1.21 (1.02-1.43)		
rs11249433	Mean lifetime intake of alcohol (g/day)	>=20	420/ 393	1.16 (0.95-1.41)		
rs11249433	Mean lifetime intake of alcohol (10g/day)	combined	5629/ 6364		1.02	3.5E-01
rs11249433	Smoking (ever)	no	8674/ 8382	1.10 (1.05-1.15)		
rs11249433	Smoking (ever)	yes	7277/ 6896	1.10 (1.05-1.16)		
rs11249433	Smoking (ever/never)	combined	15951/15278		1.00	9.7E-01
rs11249433	Smoking (pack-years)	0	7738/ 7256	1.10 (1.05-1.15)		
rs11249433	Smoking (pack-years)	0-<10	2773/ 2884	1.11 (1.03-1.20)		
rs11249433	Smoking (pack-years)	10-<20	1330/ 1300	1.08 (0.96-1.20)		
rs11249433	Smoking (pack-years)	>=20	1916/ 1633	1.08 (0.98-1.19)		
rs11249433	Smoking (pack-years/10)	combined	13757/13073		1.00	9.0E-01
rs11249433	Physical activity during year before reference date (h/week)	0	1082/ 1004	1.04 (0.92-1.17)		
rs11249433	Physical activity during year before reference date (h/week)	0-<3.5	2240/ 2387	1.09 (1.01-1.19)		
rs11249433	Physical activity during year before reference date (h/week)	3.5-<7	1390/ 1463	1.09 (0.98-1.21)		
rs11249433	Physical activity during year before reference date (h/week)	>=7	1301/ 1341	1.05 (0.94-1.17)		
rs11249433	Physical activity during year before reference date (square root of h/week)	combined	6013/ 6195		1.00	9.5E-01
rs12662670	Age at menarche (years)	<=11	2181/ 1719	1.08 (0.91-1.28)		
rs12662670	Age at menarche (years)	12-13	5744/ 4831	1.12 (1.01-1.24)		

SNP	Variable	Stratum	N (cases/ controls)	OR (95% CI)[1]	OR int[2]	P int[3]
rs12662670	Age at menarche (years)	>=14	4492/ 4336	1.19 (1.07-1.34)		
rs12662670	Age at menarche (years/2)	combined	12417/10886		1.07	1.3E-01
rs12662670	Parous	no	2335/ 1880	1.26 (1.06-1.49)		
rs12662670	Parous	yes	13503/11781	1.14 (1.07-1.22)		
rs12662670	Parous (yes/no)	combined	15838/13661		0.91	3.0E-01
rs12662670	Number of births (among parous)	1	2702/ 2151	1.13 (0.97-1.31)		
rs12662670	Number of births (among parous)	2	6041/ 5068	1.24 (1.13-1.37)		
rs12662670	Number of births (among parous)	3	2930/ 2461	1.04 (0.90-1.20)		
rs12662670	Number of births (among parous)	>=4	1486/ 1296	0.98 (0.81-1.19)		
rs12662670	Number of births (among parous)	combined	13159/10976		0.93	2.6E-02
rs12662670	Age at first birth (among parous, years)	<20	1491/ 948	1.11 (0.89-1.37)		
rs12662670	Age at first birth (among parous, years)	20-24	4500/ 4156	1.22 (1.08-1.36)		
rs12662670	Age at first birth (among parous, years)	25-29	3206/ 3080	1.01 (0.89-1.16)		
rs12662670	Age at first birth (among parous, years)	>=30	1542/ 1294	1.16 (0.95-1.41)		
rs12662670	Age at first birth (among parous, years/5)	combined	10739/ 9478		0.98	6.5E-01
rs12662670	Ever breastfed (among parous, yes/no)	no	803/ 598	1.22 (0.92-1.61)		
rs12662670	Ever breastfed (among parous, yes/no)	yes	3170/ 3418	1.11 (0.97-1.27)		
rs12662670	Ever breastfed (among parous, yes/no)	combined	3973/ 4016		0.91	5.7E-01
rs12662670	Usual adult BMI, age<54	<25	707/ 539	1.12 (0.84-1.50)		
rs12662670	Usual adult BMI, age<54	25-<30	313/ 252	1.59 (0.96-2.65)		
rs12662670	Usual adult BMI, age<54	>=30	157/ 156	2.05 (1.03-4.09)		
rs12662670	Usual adult BMI (BMI/5), age<54	combined	1177/ 947		1.16	2.7E-01
rs12662670	Usual adult BMI, age>=54	<25	2033/ 2269	1.15 (0.97-1.36)		
rs12662670	Usual adult BMI, age>=54	25-<30	1022/ 1153	1.13 (0.89-1.44)		
rs12662670	Usual adult BMI, age>=54	>=30	397/ 452	1.52 (1.02-2.28)		
rs12662670	Usual adult BMI (BMI/5), age>=54	combined	3452/ 3874		1.11	1.7E-01
rs12662670	Usual adult height (cm)	<160	1716/ 1999	1.19 (1.00-1.43)		
rs12662670	Usual adult height (cm)	160-<165	2209/ 2530	1.22 (1.04-1.43)		
rs12662670	Usual adult height (cm)	165-<170	2080/ 2244	1.26 (1.08-1.48)		
rs12662670	Usual adult height (cm)	>=170	1543/ 1525	0.96 (0.79-1.16)		
rs12662670	Usual adult height (cm/5)	combined	7548/ 8298		0.93	3.9E-02
rs12662670	Ever use of oral contraceptives	no	2972/ 2907	1.24 (1.08-1.42)		
rs12662670	Ever use of oral contraceptives	yes	3552/ 4747	1.12 (0.99-1.26)		
rs12662670	Ever use of oral contraceptives (yes/no)	combined	6524/ 7654		0.90	2.7E-01
rs12662670	Duration of oral contraceptive use (years)	0	2972/ 2907	1.24 (1.08-1.43)		
rs12662670	Duration of oral contraceptive use (years)	>0-<5	1243/ 1658	1.10 (0.90-1.35)		
rs12662670	Duration of oral contraceptive use (years)	5-<10	828/ 1138	1.06 (0.83-1.34)		
rs12662670	Duration of oral contraceptive use (years)	>=10	1370/ 1778	1.16 (0.96-1.40)		
rs12662670	Duration of oral contraceptive use (years/5)	combined	6413/ 7481		0.97	3.9E-01
rs12662670	Current use of combined estrogen/ progestagen MHT	never	1181/ 1476	1.37 (1.12-1.69)		
rs12662670	Current use of combined estrogen/ progestagen MHT	EPCurrent	627/ 509	0.84 (0.61-1.15)		
rs12662670	Current use of combined estrogen/ progestagen MHT (yes/no)	combined	2753/ 3069		0.68	2.8E-02
rs12662670	Current use of estrogen only MHT	never	1312/ 1514	1.36 (1.11-1.66)		
rs12662670	Current use of estrogen only MHT	ECurrent	460/ 455	0.93 (0.65-1.32)		
rs12662670	Current use of estrogen only MHT (yes/no)	combined	2987/ 3171		0.76	1.7E-01
rs12662670	Duration of combined estrogen/progestagen MHT among current users (years)	never	1181/ 1476	1.37 (1.12-1.69)		
rs12662670	Duration of combined estrogen/progestagen MHT among current users (years)	>0-<5	140/ 153	0.85 (0.44-1.62)		
rs12662670	Duration of combined estrogen/progestagen MHT among current users (years)	5-<10	211/ 165	0.61 (0.35-1.06)		

SNP	Variable	Stratum	N (cases/ controls)	OR (95% CI)[1]	OR int[2]	P int[3]
rs12662670	Duration of combined estrogen/progestagen MHT among current users (years)	>=10	270/ 187	0.99 (0.61-1.62)		
rs12662670	Duration of combined estrogen/progestagen MHT among current users (years/5)	combined	2743/ 3064		0.88	1.3E-01
rs12662670	Duration of estrogen only MHT among current users (years)	never	1312/ 1514	1.36 (1.11-1.66)		
rs12662670	Duration of estrogen only MHT among current users (years)	>0-<5	115/ 143	0.98 (0.53-1.80)		
rs12662670	Duration of estrogen only MHT among current users (years)	5-<10	155/ 138	0.83 (0.44-1.56)		
rs12662670	Duration of estrogen only MHT among current users (years)	>=10	186/ 172	1.01 (0.56-1.84)		
rs12662670	Duration of estrogen only MHT among current users (years/5)	combined	2975/ 3158		0.95	5.3E-01
rs12662670	Mean lifetime intake of alcohol (g/day)	0	235/ 268	1.58 (1.02-2.46)		
rs12662670	Mean lifetime intake of alcohol (g/day)	>0-<10	951/ 1057	0.98 (0.77-1.24)		
rs12662670	Mean lifetime intake of alcohol (g/day)	>=10-<20	204/ 236	1.86 (1.12-3.09)		
rs12662670	Mean lifetime intake of alcohol (g/day)	>=20	193/ 198	1.43 (0.84-2.43)		
rs12662670	Mean lifetime intake of alcohol (10g/day)	combined	1583/ 1759		1.01	8.3E-01
rs12662670	Smoking (ever)	no	3923/ 4219	1.15 (1.02-1.29)		
rs12662670	Smoking (ever)	yes	2920/ 2996	1.17 (1.01-1.35)		
rs12662670	Smoking (ever/never)	combined	6843/ 7215		1.02	8.7E-01
rs12662670	Smoking (pack-years)	0	3006/ 3096	1.18 (1.03-1.36)		
rs12662670	Smoking (pack-years)	0-<10	795/ 1035	1.11 (0.84-1.45)		
rs12662670	Smoking (pack-years)	10-<20	414/ 440	1.64 (1.13-2.37)		
rs12662670	Smoking (pack-years)	>=20	639/ 640	0.98 (0.73-1.31)		
rs12662670	Smoking (pack-years/10)	combined	4854/ 5211		1.00	9.1E-01
rs12662670	Physical activity during year before reference date (h/week)	0	35/ 32	2.44 (0.67-8.89)		
rs12662670	Physical activity during year before reference date (h/week)	0-<3.5	1222/ 1320	1.17 (0.94-1.44)		
rs12662670	Physical activity during year before reference date (h/week)	3.5-<7	856/ 991	1.33 (1.05-1.70)		
rs12662670	Physical activity during year before reference date (h/week)	>=7	1069/ 1148	1.09 (0.87-1.37)		
rs12662670	Physical activity during year before reference date (square root of h/week)	combined	3182/ 3491		0.96	4.9E-01
rs1292011	Age at menarche (years)	<=11	2356/ 1784	0.89 (0.81-0.98)		
rs1292011	Age at menarche (years)	12-13	6378/ 4745	0.95 (0.89-1.00)		
rs1292011	Age at menarche (years)	>=14	4926/ 3975	0.98 (0.92-1.04)		
rs1292011	Age at menarche (years/2)	combined	13660/10504		1.02	4.6E-01
rs1292011	Parous	no	2487/ 1737	0.95 (0.86-1.04)		
rs1292011	Parous	yes	14468/10569	0.94 (0.90-0.98)		
rs1292011	Parous (yes/no)	combined	16955/12306		0.99	8.9E-01
rs1292011	Number of births (among parous)	1	2877/ 1987	0.96 (0.88-1.05)		
rs1292011	Number of births (among parous)	2	6715/ 4688	0.95 (0.90-1.00)		
rs1292011	Number of births (among parous)	3	3220/ 2428	0.92 (0.85-1.00)		
rs1292011	Number of births (among parous)	>=4	1572/ 1437	0.93 (0.83-1.03)		
rs1292011	Number of births (among parous)	combined	14384/10540		1.00	9.3E-01
rs1292011	Age at first birth (among parous, years)	<20	1633/ 868	0.98 (0.86-1.11)		
rs1292011	Age at first birth (among parous, years)	20-24	5004/ 3984	0.94 (0.88-1.00)		
rs1292011	Age at first birth (among parous, years)	25-29	3625/ 2977	0.96 (0.89-1.04)		
rs1292011	Age at first birth (among parous, years)	>=30	1736/ 1219	0.85 (0.76-0.96)		
rs1292011	Age at first birth (among parous, years/5)	combined	11998/ 9048		0.98	3.2E-01
rs1292011	Ever breastfed (among parous, yes/no)	no	1002/ 847	0.94 (0.83-1.08)		
rs1292011	Ever breastfed (among parous, yes/no)	yes	2912/ 2851	0.94 (0.87-1.01)		
rs1292011	Ever breastfed (among parous, yes/no)	combined	3914/ 3698		0.99	9.1E-01
rs1292011	Usual adult BMI, age<54	<25	1326/ 884	0.97 (0.85-1.10)		
rs1292011	Usual adult BMI, age<54	25-<30	405/ 290	1.13 (0.89-1.42)		
rs1292011	Usual adult BMI, age<54	>=30	168/ 157	0.79 (0.58-1.10)		

SNP	Variable	Stratum	N (cases/ controls)	OR (95% CI)[1]	OR int[2]	P int[3]
rs1292011	Usual adult BMI (BMI/5), age<54	combined	1899/ 1331		0.99	8.2E-01
rs1292011	Usual adult BMI, age>=54	<25	1733/ 1702	0.92 (0.84-1.02)		
rs1292011	Usual adult BMI, age>=54	25-<30	665/ 627	0.87 (0.74-1.02)		
rs1292011	Usual adult BMI, age>=54	>=30	230/ 215	0.96 (0.74-1.26)		
rs1292011	Usual adult BMI (BMI/5), age>=54	combined	2628/ 2544		0.96	4.4E-01
rs1292011	Usual adult height (cm)	<160	1652/ 1850	0.92 (0.84-1.02)		
rs1292011	Usual adult height (cm)	160-<165	2016/ 2101	0.95 (0.87-1.04)		
rs1292011	Usual adult height (cm)	165-<170	1727/ 1756	1.03 (0.93-1.13)		
rs1292011	Usual adult height (cm)	>=170	1281/ 1208	0.93 (0.82-1.04)		
rs1292011	Usual adult height (cm/5)	combined	6676/ 6915		1.02	3.7E-01
rs1292011	Ever use of oral contraceptives	no	2177/ 2240	0.88 (0.81-0.96)		
rs1292011	Ever use of oral contraceptives	yes	3611/ 4089	1.01 (0.95-1.08)		
rs1292011	Ever use of oral contraceptives (yes/no)	combined	5788/ 6329		1.15	1.1E-02
rs1292011	Duration of oral contraceptive use (years)	0	2177/ 2240	0.88 (0.81-0.96)		
rs1292011	Duration of oral contraceptive use (years)	>0-<5	1104/ 1335	1.02 (0.90-1.14)		
rs1292011	Duration of oral contraceptive use (years)	5-<10	910/ 1043	1.00 (0.87-1.13)		
rs1292011	Duration of oral contraceptive use (years)	>=10	1525/ 1595	0.98 (0.88-1.08)		
rs1292011	Duration of oral contraceptive use (years/5)	combined	5716/ 6213		1.02	2.2E-01
rs1292011	Current use of combined estrogen/ progestagen MHT	never	944/ 1079	0.92 (0.81-1.04)		
rs1292011	Current use of combined estrogen/ progestagen MHT	EPCurrent	478/ 381	1.02 (0.84-1.23)		
rs1292011	Current use of combined estrogen/ progestagen MHT (yes/no)	combined	2124/ 2267		1.12	2.9E-01
rs1292011	Current use of estrogen only MHT	never	944/ 1079	0.92 (0.81-1.04)		
rs1292011	Current use of estrogen only MHT	ECurrent	208/ 221	0.79 (0.60-1.04)		
rs1292011	Current use of estrogen only MHT (yes/no)	combined	2126/ 2270		0.83	2.2E-01
rs1292011	Duration of combined estrogen/progestagen MHT among current users (years)	never	944/ 1079	0.92 (0.81-1.04)		
rs1292011	Duration of combined estrogen/progestagen MHT among current users (years)	>0-<5	89/ 71	0.92 (0.57-1.48)		
rs1292011	Duration of combined estrogen/progestagen MHT among current users (years)	5-<10	114/ 106	1.25 (0.87-1.82)		
rs1292011	Duration of combined estrogen/progestagen MHT among current users (years)	>=10	218/ 157	1.04 (0.78-1.38)		
rs1292011	Duration of combined estrogen/progestagen MHT among current users (years/5)	combined	2050/ 2192		1.03	5.8E-01
rs1292011	Duration of estrogen only MHT among current users (years)	never	944/ 1079	0.92 (0.81-1.04)		
rs1292011	Duration of estrogen only MHT among current users (years)	>0-<5	48/ 46	0.84 (0.45-1.55)		
rs1292011	Duration of estrogen only MHT among current users (years)	5-<10	53/ 63	0.61 (0.35-1.06)		
rs1292011	Duration of estrogen only MHT among current users (years)	>=10	94/ 96	0.93 (0.62-1.39)		
rs1292011	Duration of estrogen only MHT among current users (years/5)	combined	2095/ 2223		0.96	5.5E-01
rs1292011	Mean lifetime intake of alcohol (g/day)	0	936/ 1055	0.92 (0.81-1.05)		
rs1292011	Mean lifetime intake of alcohol (g/day)	>0-<10	1665/ 1848	0.94 (0.86-1.04)		
rs1292011	Mean lifetime intake of alcohol (g/day)	>=10-<20	332/ 392	0.80 (0.65-1.00)		
rs1292011	Mean lifetime intake of alcohol (g/day)	>=20	272/ 297	1.15 (0.91-1.46)		
rs1292011	Mean lifetime intake of alcohol (10g/day)	combined	3205/ 3592		1.07	1.8E-02
rs1292011	Smoking (ever)	no	3660/ 3889	0.92 (0.86-0.98)		
rs1292011	Smoking (ever)	yes	3079/ 2777	1.01 (0.94-1.09)		
rs1292011	Smoking (ever/never)	combined	6739/ 6666		1.10	6.8E-02
rs1292011	Smoking (pack-years)	0	3414/ 3558	0.90 (0.84-0.97)		
rs1292011	Smoking (pack-years)	0-<10	1220/ 1269	0.98 (0.87-1.10)		
rs1292011	Smoking (pack-years)	10-<20	563/ 547	1.06 (0.89-1.26)		
rs1292011	Smoking (pack-years)	>=20	768/ 662	1.04 (0.89-1.21)		
rs1292011	Smoking (pack-years/10)	combined	5965/ 6036		1.03	2.1E-01

SNP	Variable	Stratum	N (cases/ controls)	OR (95% CI)[1]	OR int[2]	P int[3]
rs1292011	Physical activity during year before reference date (h/week)	0	595/ 593	0.82 (0.69-0.96)		
rs1292011	Physical activity during year before reference date (h/week)	0-<3.5	377/ 469	1.03 (0.85-1.25)		
rs1292011	Physical activity during year before reference date (h/week)	3.5-<7	380/ 467	1.07 (0.88-1.29)		
rs1292011	Physical activity during year before reference date (h/week)	>=7	1102/ 1182	0.87 (0.77-0.98)		
rs1292011	Physical activity during year before reference date (square root of h/week)	combined	2454/ 2711		1.00	8.8E-01
rs13281615	Age at menarche (years)	<=11	3732/ 3094	1.09 (1.01-1.16)		
rs13281615	Age at menarche (years)	12-13	10408/ 9457	1.18 (1.13-1.23)		
rs13281615	Age at menarche (years)	>=14	8363/ 9023	1.09 (1.05-1.14)		
rs13281615	Age at menarche (years/2)	combined	22503/21574		0.99	5.1E-01
rs13281615	Parous	no	3987/ 3254	1.12 (1.05-1.20)		
rs13281615	Parous	yes	22450/21325	1.13 (1.10-1.17)		
rs13281615	Parous (yes/no)	combined	26437/24579		1.01	7.2E-01
rs13281615	Number of births (among parous)	1	4775/ 4083	1.12 (1.06-1.19)		
rs13281615	Number of births (among parous)	2	10127/ 9535	1.14 (1.09-1.18)		
rs13281615	Number of births (among parous)	3	4662/ 4480	1.08 (1.02-1.15)		
rs13281615	Number of births (among parous)	>=4	2424/ 2390	1.28 (1.18-1.39)		
rs13281615	Number of births (among parous)	combined	21988/20488		1.02	1.0E-01
rs13281615	Age at first birth (among parous, years)	<20	2442/ 2024	1.19 (1.10-1.30)		
rs13281615	Age at first birth (among parous, years)	20-24	8227/ 8350	1.14 (1.09-1.19)		
rs13281615	Age at first birth (among parous, years)	25-29	5991/ 6004	1.10 (1.04-1.16)		
rs13281615	Age at first birth (among parous, years)	>=30	2862/ 2504	1.12 (1.03-1.21)		
rs13281615	Age at first birth (among parous, years/5)	combined	19522/18882		0.98	1.4E-01
rs13281615	Ever breastfed (among parous, yes/no)	no	2338/ 2134	1.04 (0.96-1.13)		
rs13281615	Ever breastfed (among parous, yes/no)	yes	6856/ 7613	1.15 (1.09-1.20)		
rs13281615	Ever breastfed (among parous, yes/no)	combined	9194/ 9747		1.10	4.8E-02
rs13281615	Usual adult BMI, age<54	<25	3048/ 2779	1.09 (1.01-1.17)		
rs13281615	Usual adult BMI, age<54	25-<30	974/ 970	1.17 (1.03-1.33)		
rs13281615	Usual adult BMI, age<54	>=30	443/ 465	1.21 (1.01-1.47)		
rs13281615	Usual adult BMI (BMI/5), age<54	combined	4465/ 4214		1.04	2.3E-01
rs13281615	Usual adult BMI, age>=54	<25	3448/ 3866	1.03 (0.97-1.10)		
rs13281615	Usual adult BMI, age>=54	25-<30	1720/ 1968	1.24 (1.13-1.36)		
rs13281615	Usual adult BMI, age>=54	>=30	730/ 781	1.18 (1.02-1.37)		
rs13281615	Usual adult BMI (BMI/5), age>=54	combined	5898/ 6615		1.07	2.0E-02
rs13281615	Usual adult height (cm)	<160	4017/ 4440	1.09 (1.03-1.16)		
rs13281615	Usual adult height (cm)	160-<165	4909/ 5068	1.16 (1.10-1.23)		
rs13281615	Usual adult height (cm)	165-<170	4128/ 3990	1.12 (1.05-1.19)		
rs13281615	Usual adult height (cm)	>=170	3030/ 2623	1.12 (1.04-1.21)		
rs13281615	Usual adult height (cm/5)	combined	16084/16121		1.00	7.9E-01
rs13281615	Ever use of oral contraceptives	no	6665/ 6696	1.08 (1.02-1.13)		
rs13281615	Ever use of oral contraceptives	yes	7459/ 7978	1.15 (1.10-1.20)		
rs13281615	Ever use of oral contraceptives (yes/no)	combined	14124/14674		1.07	5.5E-02
rs13281615	Duration of oral contraceptive use (years)	0	6665/ 6696	1.08 (1.03-1.13)		
rs13281615	Duration of oral contraceptive use (years)	>0-<5	2642/ 2738	1.08 (1.00-1.17)		
rs13281615	Duration of oral contraceptive use (years)	5-<10	1835/ 1992	1.20 (1.09-1.31)		
rs13281615	Duration of oral contraceptive use (years)	>=10	2772/ 2991	1.18 (1.09-1.27)		
rs13281615	Duration of oral contraceptive use (years/5)	combined	13914/14417		1.03	2.9E-02
rs13281615	Current use of combined estrogen/ progestagen MHT	never	2500/ 3116	1.11 (1.03-1.19)		
rs13281615	Current use of combined estrogen/ progestagen MHT	EPCurrent	977/ 729	1.16 (1.01-1.33)		
rs13281615	Current use of combined estrogen/ progestagen MHT (yes/no)	combined	4899/ 5486		1.07	4.0E-01

SNP	Variable	Stratum	N (cases/ controls)	OR (95% CI)[1]	OR int[2]	P int[3]
rs13281615	Current use of estrogen only MHT	never	2644/ 3187	1.10 (1.02-1.18)		
rs13281615	Current use of estrogen only MHT	ECurrent	638/ 601	1.08 (0.92-1.27)		
rs13281615	Current use of estrogen only MHT (yes/no)	combined	5153/ 5640		0.98	8.0E-01
rs13281615	Duration of combined estrogen/progestagen MHT among current users (years)	never	2500/ 3116	1.11 (1.03-1.19)		
rs13281615	Duration of combined estrogen/progestagen MHT among current users (years)	>0-<5	239/ 238	1.20 (0.92-1.56)		
rs13281615	Duration of combined estrogen/progestagen MHT among current users (years)	5-<10	335/ 212	1.36 (1.06-1.76)		
rs13281615	Duration of combined estrogen/progestagen MHT among current users (years)	>=10	341/ 229	0.93 (0.74-1.17)		
rs13281615	Duration of combined estrogen/progestagen MHT among current users (years/5)	combined	4817/ 5404		0.99	8.3E-01
rs13281615	Duration of estrogen only MHT among current users (years)	never	2644/ 3187	1.10 (1.02-1.18)		
rs13281615	Duration of estrogen only MHT among current users (years)	>0-<5	141/ 173	1.16 (0.83-1.62)		
rs13281615	Duration of estrogen only MHT among current users (years)	5-<10	183/ 157	0.92 (0.68-1.25)		
rs13281615	Duration of estrogen only MHT among current users (years)	>=10	279/ 238	1.08 (0.85-1.38)		
rs13281615	Duration of estrogen only MHT among current users (years/5)	combined	5069/ 5535		0.97	4.0E-01
rs13281615	Mean lifetime intake of alcohol (g/day)	0	2149/ 2524	1.16 (1.07-1.26)		
rs13281615	Mean lifetime intake of alcohol (g/day)	>0-<10	2484/ 2810	1.09 (1.01-1.17)		
rs13281615	Mean lifetime intake of alcohol (g/day)	>=10-<20	515/ 580	1.16 (0.98-1.37)		
rs13281615	Mean lifetime intake of alcohol (g/day)	>=20	410/ 378	0.88 (0.72-1.08)		
rs13281615	Mean lifetime intake of alcohol (10g/day)	combined	5558/ 6292		0.96	1.5E-01
rs13281615	Smoking (ever)	no	8136/ 8020	1.13 (1.08-1.18)		
rs13281615	Smoking (ever)	yes	6807/ 6612	1.11 (1.06-1.17)		
rs13281615	Smoking (ever/never)	combined	14943/14632		0.99	6.9E-01
rs13281615	Smoking (pack-years)	0	7224/ 6895	1.12 (1.07-1.17)		
rs13281615	Smoking (pack-years)	0-<10	2612/ 2779	1.08 (1.00-1.17)		
rs13281615	Smoking (pack-years)	10-<20	1215/ 1224	1.13 (1.01-1.27)		
rs13281615	Smoking (pack-years)	>=20	1740/ 1547	1.08 (0.98-1.20)		
rs13281615	Smoking (pack-years/10)	combined	12791/12445		1.01	7.3E-01
rs13281615	Physical activity during year before reference date (h/week)	0	1080/ 999	1.10 (0.97-1.24)		
rs13281615	Physical activity during year before reference date (h/week)	0-<3.5	1962/ 2151	1.15 (1.05-1.25)		
rs13281615	Physical activity during year before reference date (h/week)	3.5-<7	1141/ 1321	1.03 (0.92-1.16)		
rs13281615	Physical activity during year before reference date (h/week)	>=7	1208/ 1276	1.01 (0.90-1.12)		
rs13281615	Physical activity during year before reference date (square root of h/week)	combined	5391/ 5747		0.97	2.3E-01
rs13387042	Age at menarche (years)	<=11	4026/ 3720	0.91 (0.85-0.97)		
rs13387042	Age at menarche (years)	12-13	11354/12415	0.90 (0.86-0.93)		
rs13387042	Age at menarche (years)	>=14	9488/12786	0.88 (0.84-0.91)		
rs13387042	Age at menarche (years/2)	combined	24868/28921		0.99	3.5E-01
rs13387042	Parous	no	4361/ 4330	0.86 (0.80-0.91)		
rs13387042	Parous	yes	24533/28506	0.89 (0.87-0.91)		
rs13387042	Parous (yes/no)	combined	28894/32836		1.04	2.9E-01
rs13387042	Number of births (among parous)	1	5385/ 5813	0.89 (0.84-0.94)		
rs13387042	Number of births (among parous)	2	11068/13131	0.90 (0.87-0.94)		
rs13387042	Number of births (among parous)	3	5053/ 5779	0.88 (0.83-0.93)		
rs13387042	Number of births (among parous)	>=4	2585/ 2908	0.85 (0.79-0.92)		
rs13387042	Number of births (among parous)	combined	24091/27631		0.99	2.7E-01
rs13387042	Age at first birth (among parous, years)	<20	2652/ 2863	0.90 (0.83-0.97)		
rs13387042	Age at first birth (among parous, years)	20-24	9087/11201	0.85 (0.82-0.89)		
rs13387042	Age at first birth (among parous, years)	25-29	6639/ 8361	0.92 (0.87-0.96)		

SNP	Variable	Stratum	N (cases/ controls)	OR (95% CI)[1]	OR int[2]	P int[3]
rs13387042	Age at first birth (among parous, years)	>=30	3250/ 3600	0.93 (0.87-1.00)		
rs13387042	Age at first birth (among parous, years/5)	combined	21628/26025		1.02	1.1E-01
rs13387042	Ever breastfed (among parous, yes/no)	no	2752/ 2884	0.84 (0.78-0.90)		
rs13387042	Ever breastfed (among parous, yes/no)	yes	8022/10331	0.88 (0.85-0.92)		
rs13387042	Ever breastfed (among parous, yes/no)	combined	10774/13215		1.05	2.4E-01
rs13387042	Usual adult BMI, age<54	<25	3248/ 3046	0.84 (0.78-0.90)		
rs13387042	Usual adult BMI, age<54	25-<30	1118/ 1028	0.82 (0.73-0.93)		
rs13387042	Usual adult BMI, age<54	>=30	511/ 508	0.96 (0.80-1.15)		
rs13387042	Usual adult BMI (BMI/5), age<54	combined	4877/ 4582		1.04	1.9E-01
rs13387042	Usual adult BMI, age>=54	<25	4464/ 6492	0.89 (0.85-0.95)		
rs13387042	Usual adult BMI, age>=54	25-<30	2094/ 2766	0.83 (0.76-0.90)		
rs13387042	Usual adult BMI, age>=54	>=30	843/ 954	0.86 (0.74-0.99)		
rs13387042	Usual adult BMI (BMI/5), age>=54	combined	7401/10212		1.00	8.7E-01
rs13387042	Usual adult height (cm)	<160	4474/ 5394	0.86 (0.82-0.92)		
rs13387042	Usual adult height (cm)	160-<165	5602/ 6464	0.88 (0.84-0.93)		
rs13387042	Usual adult height (cm)	165-<170	4778/ 5332	0.85 (0.81-0.90)		
rs13387042	Usual adult height (cm)	>=170	3467/ 3532	0.93 (0.87-0.99)		
rs13387042	Usual adult height (cm/5)	combined	18321/20722		1.02	1.0E-01
rs13387042	Ever use of oral contraceptives	no	7384/ 8529	0.85 (0.82-0.89)		
rs13387042	Ever use of oral contraceptives	yes	8677/10511	0.91 (0.87-0.94)		
rs13387042	Ever use of oral contraceptives (yes/no)	combined	16061/19040		1.06	5.9E-02
rs13387042	Duration of oral contraceptive use (years)	0	7384/ 8529	0.85 (0.82-0.89)		
rs13387042	Duration of oral contraceptive use (years)	>0-<5	3042/ 3488	0.88 (0.82-0.94)		
rs13387042	Duration of oral contraceptive use (years)	5-<10	2119/ 2562	0.88 (0.81-0.96)		
rs13387042	Duration of oral contraceptive use (years)	>=10	3293/ 4155	0.95 (0.89-1.01)		
rs13387042	Duration of oral contraceptive use (years/5)	combined	15838/18734		1.02	5.6E-02
rs13387042	Current use of combined estrogen/ progestagen MHT	never	3008/ 4533	0.89 (0.83-0.95)		
rs13387042	Current use of combined estrogen/ progestagen MHT	EPCurrent	1329/ 1373	0.76 (0.68-0.84)		
rs13387042	Current use of combined estrogen/ progestagen MHT (yes/no)	combined	6247/ 9043		0.83	2.4E-03
rs13387042	Current use of estrogen only MHT	never	3150/ 4614	0.89 (0.84-0.95)		
rs13387042	Current use of estrogen only MHT	ECurrent	763/ 1064	0.87 (0.76-1.00)		
rs13387042	Current use of estrogen only MHT (yes/no)	combined	6500/ 9229		0.99	8.5E-01
rs13387042	Duration of combined estrogen/progestagen MHT among current users (years)	never	3008/ 4533	0.89 (0.83-0.95)		
rs13387042	Duration of combined estrogen/progestagen MHT among current users (years)	>0-<5	300/ 373	0.62 (0.50-0.78)		
rs13387042	Duration of combined estrogen/progestagen MHT among current users (years)	5-<10	440/ 415	0.79 (0.65-0.96)		
rs13387042	Duration of combined estrogen/progestagen MHT among current users (years)	>=10	525/ 527	0.81 (0.68-0.96)		
rs13387042	Duration of combined estrogen/progestagen MHT among current users (years/5)	combined	6159/ 8948		0.96	1.2E-01
rs13387042	Duration of estrogen only MHT among current users (years)	never	3150/ 4614	0.89 (0.83-0.95)		
rs13387042	Duration of estrogen only MHT among current users (years)	>0-<5	174/ 299	0.74 (0.56-0.97)		
rs13387042	Duration of estrogen only MHT among current users (years)	5-<10	209/ 288	0.95 (0.73-1.22)		
rs13387042	Duration of estrogen only MHT among current users (years)	>=10	344/ 438	0.90 (0.73-1.10)		
rs13387042	Duration of estrogen only MHT among current users (years/5)	combined	6414/ 9112		1.00	8.7E-01
rs13387042	Mean lifetime intake of alcohol (g/day)	0	2322/ 3007	0.82 (0.76-0.89)		
rs13387042	Mean lifetime intake of alcohol (g/day)	>0-<10	3111/ 4942	0.94 (0.88-1.00)		
rs13387042	Mean lifetime intake of alcohol (g/day)	>=10-<20	645/ 1134	0.94 (0.82-1.08)		
rs13387042	Mean lifetime intake of alcohol (g/day)	>=20	526/ 801	0.85 (0.73-1.00)		
rs13387042	Mean lifetime intake of alcohol (10g/day)	combined	6604/ 9884		1.01	5.0E-01

SNP	Variable	Stratum	N (cases/ controls)	OR (95% CI)[1]	OR int[2]	P int[3]
rs13387042	Smoking (ever)	no	9404/10467	0.87 (0.84-0.91)		
rs13387042	Smoking (ever)	yes	7762/ 8786	0.90 (0.86-0.94)		
rs13387042	Smoking (ever/never)	combined	17166/19253		1.03	3.1E-01
rs13387042	Smoking (pack-years)	0	8453/ 9289	0.87 (0.83-0.91)		
rs13387042	Smoking (pack-years)	0-<10	2956/ 3731	0.91 (0.84-0.97)		
rs13387042	Smoking (pack-years)	10-<20	1432/ 1682	0.89 (0.81-0.99)		
rs13387042	Smoking (pack-years)	>=20	2038/ 2211	0.89 (0.82-0.98)		
rs13387042	Smoking (pack-years/10)	combined	14879/16913		1.00	1.0E+00
rs13387042	Physical activity during year before reference date (h/week)	0	1107/ 1071	0.97 (0.87-1.10)		
rs13387042	Physical activity during year before reference date (h/week)	0-<3.5	2356/ 2827	0.86 (0.80-0.93)		
rs13387042	Physical activity during year before reference date (h/week)	3.5-<7	1611/ 2211	0.85 (0.78-0.93)		
rs13387042	Physical activity during year before reference date (h/week)	>=7	1867/ 3685	0.91 (0.84-0.98)		
rs13387042	Physical activity during year before reference date (square root of h/week)	combined	6941/ 9794		0.99	5.6E-01
rs17468277[7]	Age at menarche (years)	<=11	4116/ 3868	0.99 (0.90-1.09)		
rs17468277	Age at menarche (years)	12-13	11488/12749	0.95 (0.89-1.00)		
rs17468277	Age at menarche (years)	>=14	9366/12811	0.92 (0.87-0.98)		
rs17468277	Age at menarche (years/2)	combined	24970/29428		0.98	4.1E-01
rs17468277	Parous	no	4434/ 4395	1.00 (0.91-1.09)		
rs17468277	Parous	yes	24623/28798	0.94 (0.91-0.98)		
rs17468277	Parous (yes/no)	combined	29057/33193		0.95	2.7E-01
rs17468277	Number of births (among parous)	1	5304/ 5804	0.96 (0.88-1.04)		
rs17468277	Number of births (among parous)	2	11018/13175	0.94 (0.89-0.99)		
rs17468277	Number of births (among parous)	3	5171/ 5942	0.97 (0.89-1.05)		
rs17468277	Number of births (among parous)	>=4	2658/ 3035	0.91 (0.81-1.01)		
rs17468277	Number of births (among parous)	combined	24151/27956		0.99	6.4E-01
rs17468277	Age at first birth (among parous, years)	<20	2587/ 2792	0.88 (0.78-0.99)		
rs17468277	Age at first birth (among parous, years)	20-24	9116/11439	0.94 (0.88-1.00)		
rs17468277	Age at first birth (among parous, years)	25-29	6704/ 8457	0.91 (0.85-0.98)		
rs17468277	Age at first birth (among parous, years)	>=30	3273/ 3653	1.06 (0.96-1.18)		
rs17468277	Age at first birth (among parous, years/5)	combined	21680/26341		1.03	2.0E-01
rs17468277	Ever breastfed (among parous, yes/no)	no	2649/ 2759	1.00 (0.89-1.13)		
rs17468277	Ever breastfed (among parous, yes/no)	yes	7968/10127	0.90 (0.84-0.96)		
rs17468277	Ever breastfed (among parous, yes/no)	combined	10617/12886		0.90	1.2E-01
rs17468277	Usual adult BMI, age<54	<25	3099/ 2777	0.88 (0.78-0.98)		
rs17468277	Usual adult BMI, age<54	25-<30	1050/ 925	0.96 (0.79-1.17)		
rs17468277	Usual adult BMI, age<54	>=30	493/ 478	0.93 (0.71-1.23)		
rs17468277	Usual adult BMI (BMI/5), age<54	combined	4642/ 4180		1.02	7.0E-01
rs17468277	Usual adult BMI, age>=54	<25	4475/ 6505	0.98 (0.90-1.06)		
rs17468277	Usual adult BMI, age>=54	25-<30	2093/ 2767	0.97 (0.86-1.10)		
rs17468277	Usual adult BMI, age>=54	>=30	856/ 960	0.96 (0.78-1.17)		
rs17468277	Usual adult BMI (BMI/5), age>=54	combined	7424/10232		1.03	4.0E-01
rs17468277	Usual adult height (cm)	<160	4443/ 5296	1.01 (0.92-1.10)		
rs17468277	Usual adult height (cm)	160-<165	5490/ 6255	0.92 (0.85-1.00)		
rs17468277	Usual adult height (cm)	165-<170	4640/ 5140	0.90 (0.83-0.98)		
rs17468277	Usual adult height (cm)	>=170	3425/ 3433	0.95 (0.86-1.05)		
rs17468277	Usual adult height (cm/5)	combined	17998/20124		0.98	2.2E-01
rs17468277	Ever use of oral contraceptives	no	7453/ 8361	0.91 (0.85-0.97)		
rs17468277	Ever use of oral contraceptives	yes	8661/10344	0.96 (0.90-1.02)		
rs17468277	Ever use of oral contraceptives (yes/no)	combined	16114/18705		1.05	2.9E-01

SNP	Variable	Stratum	N (cases/ controls)	OR (95% CI)[1]	OR int[2]	P int[3]
rs17468277	Duration of oral contraceptive use (years)	0	7453/ 8361	0.91 (0.85-0.97)		
rs17468277	Duration of oral contraceptive use (years)	>0-<5	3055/ 3493	1.00 (0.90-1.10)		
rs17468277	Duration of oral contraceptive use (years)	5-<10	2129/ 2546	0.89 (0.78-1.01)		
rs17468277	Duration of oral contraceptive use (years)	>=10	3263/ 4015	0.97 (0.88-1.07)		
rs17468277	Duration of oral contraceptive use (years/5)	combined	15900/18415		1.01	7.0E-01
rs17468277	Current use of combined estrogen/ progestagen MHT	never	3000/ 4542	0.97 (0.88-1.07)		
rs17468277	Current use of combined estrogen/ progestagen MHT	EPCurrent	1327/ 1373	1.00 (0.85-1.17)		
rs17468277	Current use of combined estrogen/ progestagen MHT (yes/no)	combined	6242/ 9060		1.03	7.7E-01
rs17468277	Current use of estrogen only MHT	never	3138/ 4629	0.96 (0.87-1.06)		
rs17468277	Current use of estrogen only MHT	ECurrent	766/ 1063	0.90 (0.74-1.09)		
rs17468277	Current use of estrogen only MHT (yes/no)	combined	6492/ 9253		0.91	3.7E-01
rs17468277	Duration of combined estrogen/progestagen MHT among current users (years)	never	3000/ 4542	0.97 (0.88-1.07)		
rs17468277	Duration of combined estrogen/progestagen MHT among current users (years)	>0-<5	300/ 376	1.17 (0.85-1.60)		
rs17468277	Duration of combined estrogen/progestagen MHT among current users (years)	5-<10	441/ 411	0.95 (0.71-1.27)		
rs17468277	Duration of combined estrogen/progestagen MHT among current users (years)	>=10	522/ 528	1.00 (0.78-1.28)		
rs17468277	Duration of combined estrogen/progestagen MHT among current users (years/5)	combined	6154/ 8965		1.00	9.4E-01
rs17468277	Duration of estrogen only MHT among current users (years)	never	3138/ 4629	0.96 (0.87-1.06)		
rs17468277	Duration of estrogen only MHT among current users (years)	>0-<5	175/ 301	0.90 (0.61-1.32)		
rs17468277	Duration of estrogen only MHT among current users (years)	5-<10	212/ 286	0.89 (0.60-1.31)		
rs17468277	Duration of estrogen only MHT among current users (years)	>=10	343/ 437	0.83 (0.61-1.11)		
rs17468277	Duration of estrogen only MHT among current users (years/5)	combined	6408/ 9136		0.95	2.1E-01
rs17468277	Mean lifetime intake of alcohol (g/day)	0	2226/ 2928	0.89 (0.79-1.00)		
rs17468277	Mean lifetime intake of alcohol (g/day)	>0-<10	2832/ 4586	0.93 (0.84-1.03)		
rs17468277	Mean lifetime intake of alcohol (g/day)	>=10-<20	572/ 1033	0.91 (0.73-1.14)		
rs17468277	Mean lifetime intake of alcohol (g/day)	>=20	451/ 758	1.45 (1.14-1.85)		
rs17468277	Mean lifetime intake of alcohol (10g/day)	combined	6081/ 9305		1.07	2.7E-02
rs17468277	Smoking (ever)	no	9141/10101	0.92 (0.87-0.98)		
rs17468277	Smoking (ever)	yes	7581/ 8408	0.96 (0.90-1.03)		
rs17468277	Smoking (ever/never)	combined	16722/18509		1.04	3.9E-01
rs17468277	Smoking (pack-years)	0	8183/ 8913	0.93 (0.87-0.99)		
rs17468277	Smoking (pack-years)	0-<10	2925/ 3572	0.91 (0.82-1.01)		
rs17468277	Smoking (pack-years)	10-<20	1370/ 1583	0.95 (0.81-1.11)		
rs17468277	Smoking (pack-years)	>=20	1990/ 2097	0.96 (0.84-1.09)		
rs17468277	Smoking (pack-years/10)	combined	14468/16165		1.00	8.7E-01
rs17468277	Physical activity during year before reference date (h/week)	0	1097/ 1069	1.03 (0.86-1.23)		
rs17468277	Physical activity during year before reference date (h/week)	0-<3.5	2345/ 2830	0.97 (0.87-1.09)		
rs17468277	Physical activity during year before reference date (h/week)	3.5-<7	1612/ 2219	0.96 (0.84-1.10)		
rs17468277	Physical activity during year before reference date (h/week)	>=7	1865/ 3684	1.01 (0.90-1.14)		
rs17468277	Physical activity during year before reference date (square root of h/week)	combined	6919/ 9802		0.99	5.8E-01
rs1982073	Age at menarche (years)	<=11	2542/ 2588	1.09 (1.00-1.18)		
rs1982073	Age at menarche (years)	12-13	7130/ 8764	1.07 (1.02-1.12)		
rs1982073	Age at menarche (years)	>=14	5754/ 8570	0.99 (0.94-1.05)		
rs1982073	Age at menarche (years/2)	combined	15426/19922		0.96	7.4E-02
rs1982073	Parous	no	2476/ 2852	0.96 (0.88-1.04)		
rs1982073	Parous	yes	14300/18339	1.05 (1.01-1.08)		
rs1982073	Parous (yes/no)	combined	16776/21191		1.09	4.6E-02

SNP	Variable	Stratum	N (cases/ controls)	OR (95% CI)[1]	OR int[2]	P int[3]
rs1982073	Number of births (among parous)	1	2933/ 3460	1.07 (1.00-1.16)		
rs1982073	Number of births (among parous)	2	6652/ 8602	1.03 (0.98-1.09)		
rs1982073	Number of births (among parous)	3	2927/ 3855	1.08 (1.00-1.16)		
rs1982073	Number of births (among parous)	>=4	1362/ 1841	1.03 (0.92-1.14)		
rs1982073	Number of births (among parous)	combined	13874/17758		0.99	4.0E-01
rs1982073	Age at first birth (among parous, years)	<20	1760/ 1736	0.99 (0.90-1.10)		
rs1982073	Age at first birth (among parous, years)	20-24	5710/ 7541	1.08 (1.03-1.14)		
rs1982073	Age at first birth (among parous, years)	25-29	4144/ 5891	1.07 (1.01-1.14)		
rs1982073	Age at first birth (among parous, years)	>=30	2078/ 2528	0.97 (0.89-1.06)		
rs1982073	Age at first birth (among parous, years/5)	combined	13692/17696		0.98	2.3E-01
rs1982073	Ever breastfed (among parous, yes/no)	no	654/ 727	1.11 (0.96-1.29)		
rs1982073	Ever breastfed (among parous, yes/no)	yes	3887/ 4875	1.05 (0.99-1.12)		
rs1982073	Ever breastfed (among parous, yes/no)	combined	4541/ 5602		0.95	5.0E-01
rs1982073	Usual adult BMI, age<54	<25	1779/ 1953	1.06 (0.97-1.17)		
rs1982073	Usual adult BMI, age<54	25-<30	519/ 600	1.25 (1.05-1.48)		
rs1982073	Usual adult BMI, age<54	>=30	219/ 271	0.88 (0.67-1.15)		
rs1982073	Usual adult BMI (BMI/5), age<54	combined	2517/ 2824		0.99	8.9E-01
rs1982073	Usual adult BMI, age>=54	<25	1347/ 1824	1.06 (0.95-1.17)		
rs1982073	Usual adult BMI, age>=54	25-<30	738/ 1042	1.03 (0.89-1.18)		
rs1982073	Usual adult BMI, age>=54	>=30	250/ 357	0.98 (0.76-1.26)		
rs1982073	Usual adult BMI (BMI/5), age>=54	combined	2335/ 3223		0.97	5.7E-01
rs1982073	Usual adult height (cm)	<160	1937/ 2386	1.02 (0.93-1.11)		
rs1982073	Usual adult height (cm)	160-<165	2433/ 2855	1.03 (0.95-1.11)		
rs1982073	Usual adult height (cm)	165-<170	1949/ 2058	1.03 (0.94-1.13)		
rs1982073	Usual adult height (cm)	>=170	1446/ 1311	1.07 (0.96-1.19)		
rs1982073	Usual adult height (cm/5)	combined	7765/ 8610		1.01	7.6E-01
rs1982073	Ever use of oral contraceptives	no	3566/ 4141	1.02 (0.95-1.09)		
rs1982073	Ever use of oral contraceptives	yes	3178/ 3466	1.02 (0.95-1.09)		
rs1982073	Ever use of oral contraceptives (yes/no)	combined	6744/ 7607		1.00	9.7E-01
rs1982073	Duration of oral contraceptive use (years)	0	3566/ 4141	1.02 (0.95-1.09)		
rs1982073	Duration of oral contraceptive use (years)	>0-<5	1131/ 1237	1.05 (0.93-1.18)		
rs1982073	Duration of oral contraceptive use (years)	5-<10	814/ 889	1.01 (0.88-1.16)		
rs1982073	Duration of oral contraceptive use (years)	>=10	1110/ 1189	0.96 (0.85-1.09)		
rs1982073	Duration of oral contraceptive use (years/5)	combined	6621/ 7456		0.99	6.1E-01
rs1982073	Current use of combined estrogen/ progestagen MHT	never	1291/ 1765	1.08 (0.97-1.20)		
rs1982073	Current use of combined estrogen/ progestagen MHT	EPCurrent	299/ 224	0.87 (0.68-1.10)		
rs1982073	Current use of combined estrogen/ progestagen MHT (yes/no)	combined	2037/ 2542		0.80	8.1E-02
rs1982073	Current use of estrogen only MHT	never	1425/ 1856	1.06 (0.95-1.17)		
rs1982073	Current use of estrogen only MHT	ECurrent	266/ 234	0.95 (0.74-1.22)		
rs1982073	Current use of estrogen only MHT (yes/no)	combined	2280/ 2743		0.90	4.2E-01
rs1982073	Duration of combined estrogen/progestagen MHT among current users (years)	never	1291/ 1765	1.08 (0.97-1.20)		
rs1982073	Duration of combined estrogen/progestagen MHT among current users (years)	>0-<5	103/ 112	0.76 (0.53-1.09)		
rs1982073	Duration of combined estrogen/progestagen MHT among current users (years)	5-<10	135/ 73	0.90 (0.61-1.33)		
rs1982073	Duration of combined estrogen/progestagen MHT among current users (years)	>=10	60/ 39	1.10 (0.59-2.04)		
rs1982073	Duration of combined estrogen/progestagen MHT among current users (years/5)	combined	2036/ 2542		0.95	5.8E-01
rs1982073	Duration of estrogen only MHT among current users (years)	never	1425/ 1856	1.06 (0.96-1.17)		
rs1982073	Duration of estrogen only MHT among current users (years)	>0-<5	78/ 105	0.75 (0.50-1.12)		

SNP	Variable	Stratum	N (cases/ controls)	OR (95% CI)[1]	OR int[2]	P int[3]
rs1982073	Duration of estrogen only MHT among current users (years)	5-<10	103/ 69	0.90 (0.59-1.38)		
rs1982073	Duration of estrogen only MHT among current users (years)	>=10	84/ 60	1.67 (0.97-2.88)		
rs1982073	Duration of estrogen only MHT among current users (years/5)	combined	2269/ 2728		1.06	4.1E-01
rs1982073	Mean lifetime intake of alcohol (g/day)	0	1482/ 1879	0.98 (0.89-1.08)		
rs1982073	Mean lifetime intake of alcohol (g/day)	>0-<10	1010/ 1324	1.16 (1.03-1.30)		
rs1982073	Mean lifetime intake of alcohol (g/day)	>=10-<20	249/ 272	1.33 (1.03-1.72)		
rs1982073	Mean lifetime intake of alcohol (g/day)	>=20	173/ 158	0.85 (0.62-1.17)		
rs1982073	Mean lifetime intake of alcohol (10g/day)	combined	2914/ 3633		1.05	1.7E-01
rs1982073	Smoking (ever)	no	4397/ 4677	1.02 (0.96-1.09)		
rs1982073	Smoking (ever)	yes	3406/ 3781	1.05 (0.98-1.12)		
rs1982073	Smoking (ever/never)	combined	7803/ 8458		1.03	5.8E-01
rs1982073	Smoking (pack-years)	0	3704/ 3827	1.01 (0.94-1.08)		
rs1982073	Smoking (pack-years)	0-<10	1326/ 1562	1.05 (0.94-1.17)		
rs1982073	Smoking (pack-years)	10-<20	558/ 671	1.03 (0.87-1.21)		
rs1982073	Smoking (pack-years)	>=20	626/ 713	1.10 (0.94-1.28)		
rs1982073	Smoking (pack-years/10)	combined	6214/ 6773		1.02	4.9E-01
rs1982073	Physical activity during year before reference date (h/week)	0	./ .			
rs1982073	Physical activity during year before reference date (h/week)	0-<3.5	822/ 918	1.02 (0.89-1.16)		
rs1982073	Physical activity during year before reference date (h/week)	3.5-<7	405/ 521	1.10 (0.91-1.33)		
rs1982073	Physical activity during year before reference date (h/week)	>=7	./ .			
rs1982073	Physical activity during year before reference date (square root of h/week)	combined	1227/ 1439		0.82	2.4E-01
rs2046210	Age at menarche (years)	<=11	3937/ 3411	1.07 (1.00-1.15)		
rs2046210	Age at menarche (years)	12-13	10812/10928	1.08 (1.04-1.13)		
rs2046210	Age at menarche (years)	>=14	8510/10540	1.11 (1.06-1.16)		
rs2046210	Age at menarche (years/2)	combined	23259/24879		1.01	5.8E-01
rs2046210	Parous	no	4089/ 3622	1.09 (1.02-1.17)		
rs2046210	Parous	yes	23130/24261	1.09 (1.06-1.12)		
rs2046210	Parous (yes/no)	combined	27219/27883		1.00	9.7E-01
rs2046210	Number of births (among parous)	1	4731/ 4597	1.11 (1.05-1.19)		
rs2046210	Number of births (among parous)	2	10345/11054	1.08 (1.04-1.13)		
rs2046210	Number of births (among parous)	3	4825/ 4916	1.07 (1.01-1.14)		
rs2046210	Number of births (among parous)	>=4	2451/ 2487	1.06 (0.97-1.16)		
rs2046210	Number of births (among parous)	combined	22352/23054		0.98	2.2E-01
rs2046210	Age at first birth (among parous, years)	<20	2538/ 2343	1.09 (0.99-1.18)		
rs2046210	Age at first birth (among parous, years)	20-24	8559/ 9507	1.13 (1.08-1.18)		
rs2046210	Age at first birth (among parous, years)	25-29	6305/ 7182	1.03 (0.98-1.09)		
rs2046210	Age at first birth (among parous, years)	>=30	3089/ 3184	1.07 (0.99-1.16)		
rs2046210	Age at first birth (among parous, years/5)	combined	20491/22216		0.98	2.0E-01
rs2046210	Ever breastfed (among parous, yes/no)	no	2649/ 2313	1.10 (1.02-1.20)		
rs2046210	Ever breastfed (among parous, yes/no)	yes	7609/ 7961	1.10 (1.05-1.16)		
rs2046210	Ever breastfed (among parous, yes/no)	combined	10258/10274		1.00	9.8E-01
rs2046210	Usual adult BMI, age<54	<25	3385/ 2887	1.14 (1.06-1.23)		
rs2046210	Usual adult BMI, age<54	25-<30	1151/ 1000	1.13 (0.99-1.29)		
rs2046210	Usual adult BMI, age<54	>=30	543/ 501	1.01 (0.84-1.21)		
rs2046210	Usual adult BMI (BMI/5), age<54	combined	5079/ 4388		0.96	1.7E-01
rs2046210	Usual adult BMI, age>=54	<25	3818/ 4084	1.07 (1.00-1.14)		
rs2046210	Usual adult BMI, age>=54	25-<30	1945/ 2118	1.09 (1.00-1.20)		
rs2046210	Usual adult BMI, age>=54	>=30	840/ 854	1.16 (1.00-1.34)		
rs2046210	Usual adult BMI (BMI/5), age>=54	combined	6603/ 7056		1.00	9.9E-01

SNP	Variable	Stratum	N (cases/ controls)	OR (95% CI)[1]	OR int[2]	P int[3]
rs2046210	Usual adult height (cm)	<160	3967/ 4210	1.08 (1.01-1.16)		
rs2046210	Usual adult height (cm)	160-<165	4789/ 4896	1.07 (1.01-1.14)		
rs2046210	Usual adult height (cm)	165-<170	4089/ 3814	1.16 (1.08-1.24)		
rs2046210	Usual adult height (cm)	>=170	2991/ 2523	1.04 (0.96-1.12)		
rs2046210	Usual adult height (cm/5)	combined	15836/15443		1.00	7.2E-01
rs2046210	Ever use of oral contraceptives	no	6785/ 6631	1.09 (1.03-1.15)		
rs2046210	Ever use of oral contraceptives	yes	7966/ 8053	1.09 (1.04-1.14)		
rs2046210	Ever use of oral contraceptives (yes/no)	combined	14751/14684		1.00	9.8E-01
rs2046210	Duration of oral contraceptive use (years)	0	6785/ 6631	1.09 (1.03-1.15)		
rs2046210	Duration of oral contraceptive use (years)	>0-<5	2816/ 2781	1.06 (0.98-1.14)		
rs2046210	Duration of oral contraceptive use (years)	5-<10	1994/ 2012	1.17 (1.07-1.29)		
rs2046210	Duration of oral contraceptive use (years)	>=10	2961/ 3005	1.08 (1.00-1.16)		
rs2046210	Duration of oral contraceptive use (years/5)	combined	14556/14429		1.00	9.7E-01
rs2046210	Current use of combined estrogen/ progestagen MHT	never	2718/ 3278	1.12 (1.03-1.20)		
rs2046210	Current use of combined estrogen/ progestagen MHT	EPCurrent	1071/ 783	1.05 (0.92-1.21)		
rs2046210	Current use of combined estrogen/ progestagen MHT (yes/no)	combined	5415/ 5875		0.96	6.3E-01
rs2046210	Current use of estrogen only MHT	never	2861/ 3360	1.11 (1.03-1.20)		
rs2046210	Current use of estrogen only MHT	ECurrent	700/ 673	1.00 (0.85-1.17)		
rs2046210	Current use of estrogen only MHT (yes/no)	combined	5673/ 6060		0.91	2.5E-01
rs2046210	Duration of combined estrogen/progestagen MHT among current users (years)	never	2718/ 3278	1.11 (1.03-1.20)		
rs2046210	Duration of combined estrogen/progestagen MHT among current users (years)	>0-<5	256/ 253	1.16 (0.91-1.50)		
rs2046210	Duration of combined estrogen/progestagen MHT among current users (years)	5-<10	357/ 232	0.94 (0.74-1.21)		
rs2046210	Duration of combined estrogen/progestagen MHT among current users (years)	>=10	394/ 247	1.06 (0.83-1.35)		
rs2046210	Duration of combined estrogen/progestagen MHT among current users (years/5)	combined	5327/ 5791		1.00	9.0E-01
rs2046210	Duration of estrogen only MHT among current users (years)	never	2861/ 3360	1.11 (1.03-1.20)		
rs2046210	Duration of estrogen only MHT among current users (years)	>0-<5	154/ 185	1.11 (0.79-1.55)		
rs2046210	Duration of estrogen only MHT among current users (years)	5-<10	194/ 183	0.94 (0.70-1.26)		
rs2046210	Duration of estrogen only MHT among current users (years)	>=10	315/ 272	1.04 (0.82-1.32)		
rs2046210	Duration of estrogen only MHT among current users (years/5)	combined	5585/ 5947		0.98	5.2E-01
rs2046210	Mean lifetime intake of alcohol (g/day)	0	2100/ 2503	1.14 (1.04-1.24)		
rs2046210	Mean lifetime intake of alcohol (g/day)	>0-<10	2546/ 2857	1.07 (0.99-1.16)		
rs2046210	Mean lifetime intake of alcohol (g/day)	>=10-<20	536/ 593	1.38 (1.16-1.64)		
rs2046210	Mean lifetime intake of alcohol (g/day)	>=20	420/ 389	1.15 (0.94-1.40)		
rs2046210	Mean lifetime intake of alcohol (10g/day)	combined	5602/ 6342		1.02	3.3E-01
rs2046210	Smoking (ever)	no	7780/ 7648	1.09 (1.04-1.15)		
rs2046210	Smoking (ever)	yes	6981/ 6416	1.11 (1.06-1.17)		
rs2046210	Smoking (ever/never)	combined	14761/14064		1.02	6.0E-01
rs2046210	Smoking (pack-years)	0	7103/ 6856	1.09 (1.03-1.14)		
rs2046210	Smoking (pack-years)	0-<10	2745/ 2747	1.09 (1.00-1.18)		
rs2046210	Smoking (pack-years)	10-<20	1322/ 1240	1.13 (1.01-1.27)		
rs2046210	Smoking (pack-years)	>=20	1934/ 1587	1.13 (1.03-1.25)		
rs2046210	Smoking (pack-years/10)	combined	13104/12430		1.02	2.4E-01
rs2046210	Physical activity during year before reference date (h/week)	0	1151/ 1051	1.10 (0.97-1.24)		
rs2046210	Physical activity during year before reference date (h/week)	0-<3.5	2278/ 2439	1.09 (1.00-1.19)		
rs2046210	Physical activity during year before reference date (h/week)	3.5-<7	1419/ 1492	1.02 (0.91-1.13)		
rs2046210	Physical activity during year before reference date (h/week)	>=7	1318/ 1359	1.03 (0.92-1.15)		

SNP	Variable	Stratum	N (cases/ controls)	OR (95% CI)[1]	OR int[2]	P int[3]
rs2046210	Physical activity during year before reference date (square root of h/week)	combined	6166/ 6341		0.98	2.7E-01
rs2823093	Age at menarche (years)	<=11	2312/ 1810	0.88 (0.80-0.98)		
rs2823093	Age at menarche (years)	12-13	6479/ 5221	0.97 (0.91-1.03)		
rs2823093	Age at menarche (years)	>=14	5467/ 4696	0.91 (0.85-0.97)		
rs2823093	Age at menarche (years/2)	combined	14258/11727		1.00	9.3E-01
rs2823093	Parous	no	2467/ 1972	0.91 (0.83-1.01)		
rs2823093	Parous	yes	15364/12491	0.95 (0.92-0.99)		
rs2823093	Parous (yes/no)	combined	17831/14463		1.04	4.3E-01
rs2823093	Number of births (among parous)	1	3150/ 2264	0.91 (0.83-1.00)		
rs2823093	Number of births (among parous)	2	6999/ 5182	1.01 (0.95-1.07)		
rs2823093	Number of births (among parous)	3	3271/ 2700	0.91 (0.83-0.99)		
rs2823093	Number of births (among parous)	>=4	1554/ 1559	0.91 (0.81-1.02)		
rs2823093	Number of births (among parous)	combined	14974/11705		1.00	8.3E-01
rs2823093	Age at first birth (among parous, years)	<20	1677/ 965	0.93 (0.81-1.05)		
rs2823093	Age at first birth (among parous, years)	20-24	5211/ 4435	0.94 (0.88-1.01)		
rs2823093	Age at first birth (among parous, years)	25-29	3805/ 3382	0.96 (0.88-1.04)		
rs2823093	Age at first birth (among parous, years)	>=30	1899/ 1434	0.96 (0.85-1.08)		
rs2823093	Age at first birth (among parous, years/5)	combined	12592/10216		1.02	5.1E-01
rs2823093	Ever breastfed (among parous, yes/no)	no	963/ 899	0.95 (0.82-1.10)		
rs2823093	Ever breastfed (among parous, yes/no)	yes	3685/ 4441	0.93 (0.87-1.00)		
rs2823093	Ever breastfed (among parous, yes/no)	combined	4648/ 5340		0.98	8.1E-01
rs2823093	Usual adult BMI, age<54	<25	1353/ 1108	0.91 (0.80-1.04)		
rs2823093	Usual adult BMI, age<54	25-<30	423/ 391	1.01 (0.80-1.28)		
rs2823093	Usual adult BMI, age<54	>=30	189/ 220	1.13 (0.83-1.55)		
rs2823093	Usual adult BMI (BMI/5), age<54	combined	1965/ 1719		1.05	4.0E-01
rs2823093	Usual adult BMI, age>=54	<25	2139/ 2552	0.93 (0.85-1.02)		
rs2823093	Usual adult BMI, age>=54	25-<30	972/ 1252	0.91 (0.79-1.04)		
rs2823093	Usual adult BMI, age>=54	>=30	342/ 481	0.96 (0.77-1.20)		
rs2823093	Usual adult BMI (BMI/5), age>=54	combined	3453/ 4285		1.01	8.0E-01
rs2823093	Usual adult height (cm)	<160	1735/ 2338	0.94 (0.85-1.04)		
rs2823093	Usual adult height (cm)	160-<165	2345/ 2812	0.92 (0.84-1.01)		
rs2823093	Usual adult height (cm)	165-<170	2101/ 2359	0.95 (0.86-1.04)		
rs2823093	Usual adult height (cm)	>=170	1550/ 1585	0.90 (0.80-1.01)		
rs2823093	Usual adult height (cm/5)	combined	7731/ 9094		0.98	2.6E-01
rs2823093	Ever use of oral contraceptives	no	3154/ 3401	0.97 (0.89-1.04)		
rs2823093	Ever use of oral contraceptives	yes	4501/ 5435	0.90 (0.84-0.96)		
rs2823093	Ever use of oral contraceptives (yes/no)	combined	7655/ 8836		0.93	1.5E-01
rs2823093	Duration of oral contraceptive use (years)	0	3154/ 3401	0.97 (0.89-1.04)		
rs2823093	Duration of oral contraceptive use (years)	>0-<5	1418/ 1770	0.94 (0.84-1.06)		
rs2823093	Duration of oral contraceptive use (years)	5-<10	1115/ 1343	0.93 (0.81-1.06)		
rs2823093	Duration of oral contraceptive use (years)	>=10	1838/ 2128	0.84 (0.76-0.93)		
rs2823093	Duration of oral contraceptive use (years/5)	combined	7525/ 8642		0.96	4.7E-02
rs2823093	Current use of combined estrogen/ progestagen MHT	never	1600/ 1890	0.88 (0.79-0.98)		
rs2823093	Current use of combined estrogen/ progestagen MHT	EPCurrent	692/ 553	1.01 (0.84-1.20)		
rs2823093	Current use of combined estrogen/ progestagen MHT (yes/no)	combined	3393/ 3709		1.09	3.8E-01
rs2823093	Current use of estrogen only MHT	never	1600/ 1890	0.88 (0.79-0.98)		
rs2823093	Current use of estrogen only MHT	ECurrent	466/ 454	1.22 (0.99-1.48)		
rs2823093	Current use of estrogen only MHT (yes/no)	combined	3413/ 3735		1.35	6.6E-03

SNP	Variable	Stratum	N (cases/ controls)	OR (95% CI)[1]	OR int[2]	P int[3]
rs2823093	Duration of combined estrogen/progestagen MHT among current users (years)	never	1600/ 1890	0.88 (0.79-0.98)		
rs2823093	Duration of combined estrogen/progestagen MHT among current users (years)	>0-<5	148/ 156	1.10 (0.78-1.56)		
rs2823093	Duration of combined estrogen/progestagen MHT among current users (years)	5-<10	211/ 162	1.15 (0.83-1.59)		
rs2823093	Duration of combined estrogen/progestagen MHT among current users (years)	>=10	274/ 186	1.00 (0.75-1.33)		
rs2823093	Duration of combined estrogen/progestagen MHT among current users (years/5)	combined	3314/ 3631		1.04	4.3E-01
rs2823093	Duration of estrogen only MHT among current users (years)	never	1600/ 1890	0.88 (0.79-0.98)		
rs2823093	Duration of estrogen only MHT among current users (years)	>0-<5	121/ 143	1.11 (0.78-1.59)		
rs2823093	Duration of estrogen only MHT among current users (years)	5-<10	149/ 129	1.45 (0.99-2.12)		
rs2823093	Duration of estrogen only MHT among current users (years)	>=10	180/ 165	1.09 (0.78-1.51)		
rs2823093	Duration of estrogen only MHT among current users (years/5)	combined	3378/ 3681		1.08	1.2E-01
rs2823093	Mean lifetime intake of alcohol (g/day)	0	921/ 1044	0.88 (0.76-1.02)		
rs2823093	Mean lifetime intake of alcohol (g/day)	>0-<10	1632/ 1799	0.94 (0.84-1.05)		
rs2823093	Mean lifetime intake of alcohol (g/day)	>=10-<20	324/ 381	0.91 (0.71-1.17)		
rs2823093	Mean lifetime intake of alcohol (g/day)	>=20	261/ 285	1.07 (0.82-1.40)		
rs2823093	Mean lifetime intake of alcohol (10g/day)	combined	3138/ 3509		1.02	6.0E-01
rs2823093	Smoking (ever)	no	4369/ 5150	0.94 (0.88-1.01)		
rs2823093	Smoking (ever)	yes	3422/ 3706	0.90 (0.83-0.97)		
rs2823093	Smoking (ever/never)	combined	7791/ 8856		0.95	3.3E-01
rs2823093	Smoking (pack-years)	0	3461/ 4042	0.93 (0.87-1.01)		
rs2823093	Smoking (pack-years)	0-<10	1155/ 1424	0.92 (0.81-1.05)		
rs2823093	Smoking (pack-years)	10-<20	541/ 620	0.97 (0.81-1.17)		
rs2823093	Smoking (pack-years)	>=20	697/ 773	0.87 (0.74-1.03)		
rs2823093	Smoking (pack-years/10)	combined	5854/ 6859		1.00	9.9E-01
rs2823093	Physical activity during year before reference date (h/week)	0	589/ 587	0.82 (0.68-0.98)		
rs2823093	Physical activity during year before reference date (h/week)	0-<3.5	1409/ 1578	0.85 (0.76-0.96)		
rs2823093	Physical activity during year before reference date (h/week)	3.5-<7	912/ 1061	1.03 (0.90-1.19)		
rs2823093	Physical activity during year before reference date (h/week)	>=7	1081/ 1139	0.99 (0.86-1.13)		
rs2823093	Physical activity during year before reference date (square root of h/week)	combined	3991/ 4365		1.05	9.0E-02
rs2981582	Age at menarche (years)	<=11	4478/ 3999	1.20 (1.12-1.27)		
rs2981582	Age at menarche (years)	12-13	12446/12586	1.24 (1.20-1.29)		
rs2981582	Age at menarche (years)	>=14	9775/11554	1.24 (1.19-1.29)		
rs2981582	Age at menarche (years/2)	combined	26699/28139		1.02	2.3E-01
rs2981582	Parous	no	4794/ 4453	1.27 (1.20-1.35)		
rs2981582	Parous	yes	26141/27332	1.23 (1.20-1.26)		
rs2981582	Parous (yes/no)	combined	30935/31785		0.96	2.7E-01
rs2981582	Number of births (among parous)	1	5617/ 5461	1.23 (1.17-1.30)		
rs2981582	Number of births (among parous)	2	11687/12159	1.21 (1.17-1.26)		
rs2981582	Number of births (among parous)	3	5501/ 5717	1.25 (1.18-1.32)		
rs2981582	Number of births (among parous)	>=4	2828/ 3108	1.20 (1.12-1.30)		
rs2981582	Number of births (among parous)	combined	25633/26445		1.00	7.6E-01
rs2981582	Age at first birth (among parous, years)	<20	2739/ 2638	1.18 (1.09-1.28)		
rs2981582	Age at first birth (among parous, years)	20-24	9627/10922	1.21 (1.16-1.26)		
rs2981582	Age at first birth (among parous, years)	25-29	7193/ 7909	1.26 (1.20-1.32)		
rs2981582	Age at first birth (among parous, years)	>=30	3543/ 3356	1.26 (1.17-1.35)		
rs2981582	Age at first birth (among parous, years/5)	combined	23102/24825		1.02	2.2E-01
rs2981582	Ever breastfed (among parous, yes/no)	no	2744/ 2872	1.20 (1.11-1.30)		

SNP	Variable	Stratum	N (cases/ controls)	OR (95% CI)[1]	OR int[2]	P int[3]
rs2981582	Ever breastfed (among parous, yes/no)	yes	7997/10162	1.21 (1.16-1.26)		
rs2981582	Ever breastfed (among parous, yes/no)	combined	10741/13034		1.01	8.5E-01
rs2981582	Usual adult BMI, age<54	<25	3254/ 3042	1.22 (1.13-1.31)		
rs2981582	Usual adult BMI, age<54	25-<30	1099/ 1039	1.26 (1.11-1.43)		
rs2981582	Usual adult BMI, age<54	>=30	507/ 506	1.18 (0.98-1.42)		
rs2981582	Usual adult BMI (BMI/5), age<54	combined	4860/ 4587		0.99	8.4E-01
rs2981582	Usual adult BMI, age>=54	<25	4389/ 6309	1.25 (1.18-1.32)		
rs2981582	Usual adult BMI, age>=54	25-<30	2055/ 2739	1.24 (1.14-1.35)		
rs2981582	Usual adult BMI, age>=54	>=30	830/ 950	1.18 (1.02-1.36)		
rs2981582	Usual adult BMI (BMI/5), age>=54	combined	7274/ 9998		1.01	8.1E-01
rs2981582	Usual adult height (cm)	<160	4525/ 5459	1.25 (1.17-1.32)		
rs2981582	Usual adult height (cm)	160-<165	5632/ 6538	1.18 (1.12-1.25)		
rs2981582	Usual adult height (cm)	165-<170	4830/ 5400	1.25 (1.18-1.32)		
rs2981582	Usual adult height (cm)	>=170	3515/ 3618	1.27 (1.19-1.37)		
rs2981582	Usual adult height (cm/5)	combined	18502/21015		1.01	3.4E-01
rs2981582	Ever use of oral contraceptives	no	7451/ 8278	1.25 (1.20-1.31)		
rs2981582	Ever use of oral contraceptives	yes	8819/11154	1.22 (1.17-1.27)		
rs2981582	Ever use of oral contraceptives (yes/no)	combined	16270/19432		0.97	3.4E-01
rs2981582	Duration of oral contraceptive use (years)	0	7451/ 8278	1.25 (1.20-1.31)		
rs2981582	Duration of oral contraceptive use (years)	>0-<5	3097/ 3815	1.24 (1.15-1.32)		
rs2981582	Duration of oral contraceptive use (years)	5-<10	2143/ 2739	1.21 (1.12-1.32)		
rs2981582	Duration of oral contraceptive use (years)	>=10	3355/ 4299	1.21 (1.13-1.29)		
rs2981582	Duration of oral contraceptive use (years/5)	combined	16046/19131		0.99	2.9E-01
rs2981582	Current use of combined estrogen/ progestagen MHT	never	2983/ 4470	1.25 (1.17-1.34)		
rs2981582	Current use of combined estrogen/ progestagen MHT	EPCurrent	1289/ 1320	1.24 (1.11-1.39)		
rs2981582	Current use of combined estrogen/ progestagen MHT (yes/no)	combined	6146/ 8831		1.00	9.8E-01
rs2981582	Current use of estrogen only MHT	never	3107/ 4558	1.26 (1.18-1.35)		
rs2981582	Current use of estrogen only MHT	ECurrent	752/ 1039	1.33 (1.16-1.53)		
rs2981582	Current use of estrogen only MHT (yes/no)	combined	6365/ 9021		1.07	3.6E-01
rs2981582	Duration of combined estrogen/progestagen MHT among current users (years)	never	2983/ 4470	1.25 (1.17-1.34)		
rs2981582	Duration of combined estrogen/progestagen MHT among current users (years)	>0-<5	292/ 369	1.23 (0.97-1.56)		
rs2981582	Duration of combined estrogen/progestagen MHT among current users (years)	5-<10	430/ 397	1.23 (1.00-1.51)		
rs2981582	Duration of combined estrogen/progestagen MHT among current users (years)	>=10	503/ 496	1.25 (1.05-1.50)		
rs2981582	Duration of combined estrogen/progestagen MHT among current users (years/5)	combined	6058/ 8736		1.00	8.9E-01
rs2981582	Duration of estrogen only MHT among current users (years)	never	3107/ 4558	1.26 (1.18-1.35)		
rs2981582	Duration of estrogen only MHT among current users (years)	>0-<5	171/ 295	1.69 (1.25-2.27)		
rs2981582	Duration of estrogen only MHT among current users (years)	5-<10	206/ 282	1.10 (0.85-1.43)		
rs2981582	Duration of estrogen only MHT among current users (years)	>=10	339/ 423	1.38 (1.12-1.70)		
rs2981582	Duration of estrogen only MHT among current users (years/5)	combined	6281/ 8905		1.02	6.0E-01
rs2981582	Mean lifetime intake of alcohol (g/day)	0	2324/ 2986	1.23 (1.13-1.33)		
rs2981582	Mean lifetime intake of alcohol (g/day)	>0-<10	3048/ 4796	1.25 (1.17-1.34)		
rs2981582	Mean lifetime intake of alcohol (g/day)	>=10-<20	628/ 1086	1.03 (0.89-1.19)		
rs2981582	Mean lifetime intake of alcohol (g/day)	>=20	508/ 766	1.27 (1.08-1.49)		
rs2981582	Mean lifetime intake of alcohol (10g/day)	combined	6508/ 9634		0.98	2.4E-01
rs2981582	Smoking (ever)	no	9501/10701	1.24 (1.19-1.29)		
rs2981582	Smoking (ever)	yes	7777/ 8723	1.21 (1.16-1.27)		
rs2981582	Smoking (ever/never)	combined	17278/19424		0.98	5.0E-01

SNP	Variable	Stratum	N (cases/ controls)	OR (95% CI)[1]	OR int[2]	P int[3]
rs2981582	Smoking (pack-years)	0	8553/ 9508	1.25 (1.19-1.30)		
rs2981582	Smoking (pack-years)	0-<10	2933/ 3747	1.16 (1.08-1.25)		
rs2981582	Smoking (pack-years)	10-<20	1411/ 1650	1.21 (1.09-1.34)		
rs2981582	Smoking (pack-years)	>=20	2026/ 2170	1.32 (1.20-1.44)		
rs2981582	Smoking (pack-years/10)	combined	14923/17075		1.02	1.6E-01
rs2981582	Physical activity during year before reference date (h/week)	0	1100/ 1069	1.30 (1.15-1.47)		
rs2981582	Physical activity during year before reference date (h/week)	0-<3.5	2374/ 2820	1.23 (1.13-1.33)		
rs2981582	Physical activity during year before reference date (h/week)	3.5-<7	1602/ 2175	1.24 (1.13-1.37)		
rs2981582	Physical activity during year before reference date (h/week)	>=7	1755/ 3491	1.21 (1.11-1.32)		
rs2981582	Physical activity during year before reference date (square root of h/week)	combined	6831/ 9555		1.00	9.3E-01
rs3803662	Age at menarche (years)	<=11	3997/ 3391	1.24 (1.16-1.34)		
rs3803662	Age at menarche (years)	12-13	11040/10489	1.27 (1.21-1.32)		
rs3803662	Age at menarche (years)	>=14	8715/ 9962	1.21 (1.15-1.26)		
rs3803662	Age at menarche (years/2)	combined	23752/23842		0.98	2.4E-01
rs3803662	Parous	no	4125/ 3545	1.34 (1.24-1.44)		
rs3803662	Parous	yes	22991/23652	1.23 (1.20-1.27)		
rs3803662	Parous (yes/no)	combined	27116/27197		0.92	4.7E-02
rs3803662	Number of births (among parous)	1	4804/ 4601	1.26 (1.18-1.35)		
rs3803662	Number of births (among parous)	2	10333/10467	1.24 (1.18-1.29)		
rs3803662	Number of births (among parous)	3	4876/ 5009	1.26 (1.18-1.34)		
rs3803662	Number of births (among parous)	>=4	2533/ 2729	1.16 (1.06-1.27)		
rs3803662	Number of births (among parous)	combined	22546/22806		0.98	2.3E-01
rs3803662	Age at first birth (among parous, years)	<20	2530/ 2278	1.10 (1.00-1.20)		
rs3803662	Age at first birth (among parous, years)	20-24	8680/ 9459	1.26 (1.20-1.32)		
rs3803662	Age at first birth (among parous, years)	25-29	6330/ 6675	1.23 (1.16-1.30)		
rs3803662	Age at first birth (among parous, years)	>=30	3053/ 2771	1.21 (1.11-1.31)		
rs3803662	Age at first birth (among parous, years/5)	combined	20593/21183		1.01	5.5E-01
rs3803662	Ever breastfed (among parous, yes/no)	no	2482/ 2484	1.22 (1.11-1.33)		
rs3803662	Ever breastfed (among parous, yes/no)	yes	7194/ 8636	1.20 (1.14-1.26)		
rs3803662	Ever breastfed (among parous, yes/no)	combined	9676/11120		0.99	7.8E-01
rs3803662	Usual adult BMI, age<54	<25	3128/ 2793	1.23 (1.13-1.33)		
rs3803662	Usual adult BMI, age<54	25-<30	1060/ 955	1.09 (0.95-1.25)		
rs3803662	Usual adult BMI, age<54	>=30	475/ 467	1.08 (0.88-1.32)		
rs3803662	Usual adult BMI (BMI/5), age<54	combined	4663/ 4215		0.97	4.0E-01
rs3803662	Usual adult BMI, age>=54	<25	3636/ 4873	1.26 (1.18-1.35)		
rs3803662	Usual adult BMI, age>=54	25-<30	1823/ 2338	1.17 (1.06-1.29)		
rs3803662	Usual adult BMI, age>=54	>=30	772/ 834	1.25 (1.06-1.47)		
rs3803662	Usual adult BMI (BMI/5), age>=54	combined	6231/ 8045		0.97	3.7E-01
rs3803662	Usual adult height (cm)	<160	4207/ 4876	1.28 (1.20-1.37)		
rs3803662	Usual adult height (cm)	160-<165	5139/ 5547	1.23 (1.15-1.30)		
rs3803662	Usual adult height (cm)	165-<170	4274/ 4423	1.21 (1.13-1.30)		
rs3803662	Usual adult height (cm)	>=170	3092/ 2844	1.25 (1.16-1.36)		
rs3803662	Usual adult height (cm/5)	combined	16712/17690		0.99	5.1E-01
rs3803662	Ever use of oral contraceptives	no	6954/ 7377	1.18 (1.12-1.24)		
rs3803662	Ever use of oral contraceptives	yes	7796/ 8827	1.25 (1.19-1.31)		
rs3803662	Ever use of oral contraceptives (yes/no)	combined	14750/16204		1.06	1.3E-01
rs3803662	Duration of oral contraceptive use (years)	0	6954/ 7377	1.18 (1.12-1.24)		
rs3803662	Duration of oral contraceptive use (years)	>0-<5	2801/ 3027	1.23 (1.13-1.34)		
rs3803662	Duration of oral contraceptive use (years)	5-<10	1928/ 2174	1.26 (1.14-1.39)		

SNP	Variable	Stratum	N (cases/ controls)	OR (95% CI)[1]	OR int[2]	P int[3]
rs3803662	Duration of oral contraceptive use (years)	>=10	2852/ 3353	1.25 (1.16-1.36)		
rs3803662	Duration of oral contraceptive use (years/5)	combined	14535/15931		1.03	7.8E-02
rs3803662	Current use of combined estrogen/ progestagen MHT	never	2614/ 3756	1.24 (1.14-1.34)		
rs3803662	Current use of combined estrogen/ progestagen MHT	EPCurrent	983/ 924	1.22 (1.07-1.41)		
rs3803662	Current use of combined estrogen/ progestagen MHT (yes/no)	combined	5081/ 6921		1.01	9.3E-01
rs3803662	Current use of estrogen only MHT	never	2749/ 3829	1.24 (1.14-1.34)		
rs3803662	Current use of estrogen only MHT	ECurrent	654/ 798	1.28 (1.08-1.51)		
rs3803662	Current use of estrogen only MHT (yes/no)	combined	5323/ 7082		1.06	5.4E-01
rs3803662	Duration of combined estrogen/progestagen MHT among current users (years)	never	2614/ 3756	1.24 (1.14-1.34)		
rs3803662	Duration of combined estrogen/progestagen MHT among current users (years)	>0-<5	249/ 293	1.18 (0.90-1.54)		
rs3803662	Duration of combined estrogen/progestagen MHT among current users (years)	5-<10	337/ 281	1.22 (0.96-1.55)		
rs3803662	Duration of combined estrogen/progestagen MHT among current users (years)	>=10	338/ 299	1.31 (1.03-1.68)		
rs3803662	Duration of combined estrogen/progestagen MHT among current users (years/5)	combined	5002/ 6836		1.02	5.6E-01
rs3803662	Duration of estrogen only MHT among current users (years)	never	2749/ 3829	1.24 (1.14-1.34)		
rs3803662	Duration of estrogen only MHT among current users (years)	>0-<5	157/ 235	1.46 (1.05-2.04)		
rs3803662	Duration of estrogen only MHT among current users (years)	5-<10	179/ 214	0.96 (0.69-1.32)		
rs3803662	Duration of estrogen only MHT among current users (years)	>=10	283/ 314	1.27 (0.98-1.65)		
rs3803662	Duration of estrogen only MHT among current users (years/5)	combined	5240/ 6976		0.98	6.9E-01
rs3803662	Mean lifetime intake of alcohol (g/day)	0	2217/ 2784	1.15 (1.05-1.25)		
rs3803662	Mean lifetime intake of alcohol (g/day)	>0-<10	2523/ 3707	1.27 (1.17-1.37)		
rs3803662	Mean lifetime intake of alcohol (g/day)	>=10-<20	515/ 773	1.12 (0.93-1.33)		
rs3803662	Mean lifetime intake of alcohol (g/day)	>=20	405/ 544	1.10 (0.89-1.34)		
rs3803662	Mean lifetime intake of alcohol (10g/day)	combined	5660/ 7808		0.99	6.1E-01
rs3803662	Smoking (ever)	no	8515/ 8933	1.25 (1.19-1.31)		
rs3803662	Smoking (ever)	yes	6960/ 7215	1.23 (1.17-1.30)		
rs3803662	Smoking (ever/never)	combined	15475/16148		0.98	6.5E-01
rs3803662	Smoking (pack-years)	0	7556/ 7745	1.24 (1.18-1.31)		
rs3803662	Smoking (pack-years)	0-<10	2680/ 3076	1.18 (1.09-1.28)		
rs3803662	Smoking (pack-years)	10-<20	1241/ 1332	1.24 (1.09-1.40)		
rs3803662	Smoking (pack-years)	>=20	1784/ 1706	1.28 (1.15-1.42)		
rs3803662	Smoking (pack-years/10)	combined	13261/13859		1.01	4.2E-01
rs3803662	Physical activity during year before reference date (h/week)	0	1092/ 1055	1.29 (1.13-1.48)		
rs3803662	Physical activity during year before reference date (h/week)	0-<3.5	2087/ 2477	1.27 (1.16-1.39)		
rs3803662	Physical activity during year before reference date (h/week)	3.5-<7	1256/ 1704	1.07 (0.96-1.20)		
rs3803662	Physical activity during year before reference date (h/week)	>=7	1125/ 2104	1.19 (1.06-1.33)		
rs3803662	Physical activity during year before reference date (square root of h/week)	combined	5560/ 7340		0.97	2.2E-01
rs3817198	Age at menarche (years)	<=11	4001/ 3338	1.09 (1.01-1.17)		
rs3817198	Age at menarche (years)	12-13	11054/10153	1.13 (1.08-1.17)		
rs3817198	Age at menarche (years)	>=14	8694/ 9624	1.07 (1.02-1.12)		
rs3817198	Age at menarche (years/2)	combined	23749/23115		0.99	7.2E-01
rs3817198	Parous	no	4189/ 3435	1.08 (1.01-1.16)		
rs3817198	Parous	yes	23398/23015	1.10 (1.07-1.13)		
rs3817198	Parous (yes/no)	combined	27587/26450		1.02	6.8E-01
rs3817198	Number of births (among parous)	1	4957/ 4464	1.03 (0.96-1.10)		
rs3817198	Number of births (among parous)	2	10549/10234	1.07 (1.02-1.11)		
rs3817198	Number of births (among parous)	3	4970/ 4821	1.16 (1.09-1.23)		

SNP	Variable	Stratum	N (cases/ controls)	OR (95% CI)[1]	OR int[2]	P int[3]
rs3817198	Number of births (among parous)	>=4	2588/ 2632	1.26 (1.16-1.37)		
rs3817198	Number of births (among parous)	combined	23064/22151		1.06	2.4E-06
rs3817198	Age at first birth (among parous, years)	<20	2533/ 2202	1.11 (1.02-1.22)		
rs3817198	Age at first birth (among parous, years)	20-24	8710/ 9203	1.10 (1.05-1.15)		
rs3817198	Age at first birth (among parous, years)	25-29	6308/ 6484	1.10 (1.04-1.16)		
rs3817198	Age at first birth (among parous, years)	>=30	3047/ 2691	1.09 (1.00-1.18)		
rs3817198	Age at first birth (among parous, years/5)	combined	20598/20580		0.99	4.5E-01
rs3817198	Ever breastfed (among parous, yes/no)	no	2477/ 2499	1.07 (0.98-1.16)		
rs3817198	Ever breastfed (among parous, yes/no)	yes	7090/ 8692	1.11 (1.05-1.16)		
rs3817198	Ever breastfed (among parous, yes/no)	combined	9567/11191		1.04	4.8E-01
rs3817198	Usual adult BMI, age<54	<25	3073/ 2813	1.08 (1.00-1.17)		
rs3817198	Usual adult BMI, age<54	25-<30	1033/ 965	1.11 (0.97-1.28)		
rs3817198	Usual adult BMI, age<54	>=30	459/ 466	1.01 (0.83-1.22)		
rs3817198	Usual adult BMI (BMI/5), age<54	combined	4565/ 4244		0.98	6.3E-01
rs3817198	Usual adult BMI, age>=54	<25	3636/ 4886	1.07 (1.00-1.15)		
rs3817198	Usual adult BMI, age>=54	25-<30	1830/ 2354	1.11 (1.01-1.22)		
rs3817198	Usual adult BMI, age>=54	>=30	779/ 841	1.17 (1.00-1.37)		
rs3817198	Usual adult BMI (BMI/5), age>=54	combined	6245/ 8081		1.02	4.5E-01
rs3817198	Usual adult height (cm)	<160	4188/ 4746	1.10 (1.03-1.18)		
rs3817198	Usual adult height (cm)	160-<165	5087/ 5363	1.09 (1.02-1.15)		
rs3817198	Usual adult height (cm)	165-<170	4217/ 4235	1.12 (1.05-1.20)		
rs3817198	Usual adult height (cm)	>=170	3045/ 2686	1.00 (0.92-1.08)		
rs3817198	Usual adult height (cm/5)	combined	16537/17030		0.98	1.9E-01
rs3817198	Ever use of oral contraceptives	no	6948/ 7403	1.07 (1.02-1.13)		
rs3817198	Ever use of oral contraceptives	yes	7674/ 8856	1.08 (1.03-1.14)		
rs3817198	Ever use of oral contraceptives (yes/no)	combined	14622/16259		1.01	8.2E-01
rs3817198	Duration of oral contraceptive use (years)	0	6948/ 7403	1.07 (1.02-1.13)		
rs3817198	Duration of oral contraceptive use (years)	>0-<5	2764/ 3047	1.15 (1.06-1.25)		
rs3817198	Duration of oral contraceptive use (years)	5-<10	1885/ 2176	1.00 (0.90-1.10)		
rs3817198	Duration of oral contraceptive use (years)	>=10	2810/ 3361	1.07 (0.99-1.15)		
rs3817198	Duration of oral contraceptive use (years/5)	combined	14407/15987		0.98	9.9E-02
rs3817198	Current use of combined estrogen/ progestagen MHT	never	2624/ 3759	1.09 (1.01-1.17)		
rs3817198	Current use of combined estrogen/ progestagen MHT	EPCurrent	984/ 928	1.07 (0.93-1.23)		
rs3817198	Current use of combined estrogen/ progestagen MHT (yes/no)	combined	5099/ 6942		0.99	9.4E-01
rs3817198	Current use of estrogen only MHT	never	2768/ 3836	1.09 (1.01-1.18)		
rs3817198	Current use of estrogen only MHT	ECurrent	655/ 808	1.04 (0.89-1.22)		
rs3817198	Current use of estrogen only MHT (yes/no)	combined	5350/ 7105		0.97	7.0E-01
rs3817198	Duration of combined estrogen/progestagen MHT among current users (years)	never	2624/ 3759	1.09 (1.01-1.17)		
rs3817198	Duration of combined estrogen/progestagen MHT among current users (years)	>0-<5	248/ 295	0.95 (0.73-1.25)		
rs3817198	Duration of combined estrogen/progestagen MHT among current users (years)	5-<10	338/ 282	1.01 (0.80-1.28)		
rs3817198	Duration of combined estrogen/progestagen MHT among current users (years)	>=10	339/ 300	1.24 (0.97-1.58)		
rs3817198	Duration of combined estrogen/progestagen MHT among current users (years/5)	combined	5020/ 6857		1.03	4.3E-01
rs3817198	Duration of estrogen only MHT among current users (years)	never	2768/ 3836	1.09 (1.01-1.18)		
rs3817198	Duration of estrogen only MHT among current users (years)	>0-<5	157/ 236	0.90 (0.65-1.24)		
rs3817198	Duration of estrogen only MHT among current users (years)	5-<10	180/ 216	1.32 (0.97-1.79)		
rs3817198	Duration of estrogen only MHT among current users (years)	>=10	283/ 320	1.04 (0.81-1.33)		
rs3817198	Duration of estrogen only MHT among current users (years/5)	combined	5266/ 6998		1.00	9.2E-01

SNP	Variable	Stratum	N (cases/ controls)	OR (95% CI)[1]	OR int[2]	P int[3]
rs3817198	Mean lifetime intake of alcohol (g/day)	0	2217/ 2796	1.09 (1.00-1.19)		
rs3817198	Mean lifetime intake of alcohol (g/day)	>0-<10	2524/ 3712	1.07 (0.99-1.15)		
rs3817198	Mean lifetime intake of alcohol (g/day)	>=10-<20	508/ 777	1.28 (1.07-1.52)		
rs3817198	Mean lifetime intake of alcohol (g/day)	>=20	404/ 545	0.90 (0.74-1.09)		
rs3817198	Mean lifetime intake of alcohol (10g/day)	combined	5653/ 7830		0.99	7.9E-01
rs3817198	Smoking (ever)	no	8390/ 8570	1.08 (1.03-1.13)		
rs3817198	Smoking (ever)	yes	6911/ 6964	1.11 (1.05-1.16)		
rs3817198	Smoking (ever/never)	combined	15301/15534		1.03	4.6E-01
rs3817198	Smoking (pack-years)	0	7434/ 7378	1.08 (1.03-1.14)		
rs3817198	Smoking (pack-years)	0-<10	2650/ 2975	1.11 (1.02-1.20)		
rs3817198	Smoking (pack-years)	10-<20	1239/ 1283	1.12 (0.99-1.26)		
rs3817198	Smoking (pack-years)	>=20	1770/ 1663	1.13 (1.01-1.25)		
rs3817198	Smoking (pack-years/10)	combined	13093/13299		1.00	7.9E-01
rs3817198	Physical activity during year before reference date (h/week)	0	1097/ 1061	1.19 (1.05-1.35)		
rs3817198	Physical activity during year before reference date (h/week)	0-<3.5	2095/ 2492	1.04 (0.95-1.14)		
rs3817198	Physical activity during year before reference date (h/week)	3.5-<7	1261/ 1709	1.08 (0.97-1.21)		
rs3817198	Physical activity during year before reference date (h/week)	>=7	1126/ 2106	1.04 (0.94-1.17)		
rs3817198	Physical activity during year before reference date (square root of h/week)	combined	5579/ 7368		0.98	3.2E-01
rs4973768	Age at menarche (years)	<=11	3981/ 3680	1.13 (1.05-1.20)		
rs4973768	Age at menarche (years)	12-13	11237/12211	1.08 (1.04-1.12)		
rs4973768	Age at menarche (years)	>=14	9270/12465	1.11 (1.06-1.15)		
rs4973768	Age at menarche (years/2)	combined	24488/28356		0.99	3.7E-01
rs4973768	Parous	no	4299/ 4238	1.12 (1.05-1.19)		
rs4973768	Parous	yes	24185/27677	1.10 (1.07-1.13)		
rs4973768	Parous (yes/no)	combined	28484/31915		0.99	6.8E-01
rs4973768	Number of births (among parous)	1	5273/ 5633	1.08 (1.02-1.15)		
rs4973768	Number of births (among parous)	2	10930/12765	1.10 (1.06-1.14)		
rs4973768	Number of births (among parous)	3	5005/ 5653	1.13 (1.07-1.20)		
rs4973768	Number of births (among parous)	>=4	2518/ 2815	1.10 (1.02-1.19)		
rs4973768	Number of births (among parous)	combined	23726/26866		1.01	5.9E-01
rs4973768	Age at first birth (among parous, years)	<20	2666/ 2811	1.14 (1.06-1.24)		
rs4973768	Age at first birth (among parous, years)	20-24	9067/11142	1.09 (1.04-1.13)		
rs4973768	Age at first birth (among parous, years)	25-29	6650/ 8223	1.11 (1.06-1.17)		
rs4973768	Age at first birth (among parous, years)	>=30	3289/ 3538	1.11 (1.03-1.19)		
rs4973768	Age at first birth (among parous, years/5)	combined	21672/25714		1.01	7.3E-01
rs4973768	Ever breastfed (among parous, yes/no)	no	2723/ 2865	1.07 (0.99-1.16)		
rs4973768	Ever breastfed (among parous, yes/no)	yes	8074/10281	1.12 (1.08-1.17)		
rs4973768	Ever breastfed (among parous, yes/no)	combined	10797/13146		1.05	2.8E-01
rs4973768	Usual adult BMI, age<54	<25	3361/ 3090	1.11 (1.03-1.19)		
rs4973768	Usual adult BMI, age<54	25-<30	1144/ 1042	1.05 (0.93-1.18)		
rs4973768	Usual adult BMI, age<54	>=30	536/ 519	1.28 (1.08-1.52)		
rs4973768	Usual adult BMI (BMI/5), age<54	combined	5041/ 4651		1.02	4.3E-01
rs4973768	Usual adult BMI, age>=54	<25	4408/ 6445	1.07 (1.01-1.13)		
rs4973768	Usual adult BMI, age>=54	25-<30	2048/ 2743	1.14 (1.05-1.24)		
rs4973768	Usual adult BMI, age>=54	>=30	832/ 956	1.10 (0.96-1.25)		
rs4973768	Usual adult BMI (BMI/5), age>=54	combined	7288/10144		0.99	7.7E-01
rs4973768	Usual adult height (cm)	<160	4361/ 5110	1.21 (1.14-1.28)		
rs4973768	Usual adult height (cm)	160-<165	5474/ 6190	1.07 (1.02-1.13)		
rs4973768	Usual adult height (cm)	165-<170	4669/ 5106	1.09 (1.03-1.15)		

SNP	Variable	Stratum	N (cases/ controls)	OR (95% CI)[1]	OR int[2]	P int[3]
rs4973768	Usual adult height (cm)	>=170	3466/ 3415	1.10 (1.02-1.17)		
rs4973768	Usual adult height (cm/5)	combined	17970/19821		0.97	1.7E-02
rs4973768	Ever use of oral contraceptives	no	7184/ 7878	1.08 (1.03-1.13)		
rs4973768	Ever use of oral contraceptives	yes	8624/10403	1.13 (1.09-1.18)		
rs4973768	Ever use of oral contraceptives (yes/no)	combined	15808/18281		1.05	9.7E-02
rs4973768	Duration of oral contraceptive use (years)	0	7184/ 7878	1.08 (1.03-1.13)		
rs4973768	Duration of oral contraceptive use (years)	>0-<5	3001/ 3464	1.10 (1.02-1.18)		
rs4973768	Duration of oral contraceptive use (years)	5-<10	2125/ 2582	1.17 (1.08-1.28)		
rs4973768	Duration of oral contraceptive use (years)	>=10	3286/ 4087	1.13 (1.05-1.20)		
rs4973768	Duration of oral contraceptive use (years/5)	combined	15596/18011		1.01	3.5E-01
rs4973768	Current use of combined estrogen/ progestagen MHT	never	2965/ 4500	1.07 (1.01-1.15)		
rs4973768	Current use of combined estrogen/ progestagen MHT	EPCurrent	1328/ 1368	1.11 (1.00-1.24)		
rs4973768	Current use of combined estrogen/ progestagen MHT (yes/no)	combined	6196/ 9007		1.03	6.2E-01
rs4973768	Current use of estrogen only MHT	never	3105/ 4577	1.07 (1.00-1.14)		
rs4973768	Current use of estrogen only MHT	ECurrent	761/ 1063	1.04 (0.91-1.19)		
rs4973768	Current use of estrogen only MHT (yes/no)	combined	6449/ 9189		0.95	5.1E-01
rs4973768	Duration of combined estrogen/progestagen MHT among current users (years)	never	2965/ 4500	1.07 (1.00-1.15)		
rs4973768	Duration of combined estrogen/progestagen MHT among current users (years)	>0-<5	297/ 374	0.97 (0.78-1.22)		
rs4973768	Duration of combined estrogen/progestagen MHT among current users (years)	5-<10	441/ 412	1.11 (0.91-1.35)		
rs4973768	Duration of combined estrogen/progestagen MHT among current users (years)	>=10	526/ 524	1.19 (1.00-1.41)		
rs4973768	Duration of combined estrogen/progestagen MHT among current users (years/5)	combined	6108/ 8912		1.02	4.0E-01
rs4973768	Duration of estrogen only MHT among current users (years)	never	3105/ 4577	1.07 (1.00-1.14)		
rs4973768	Duration of estrogen only MHT among current users (years)	>0-<5	172/ 303	1.28 (0.98-1.68)		
rs4973768	Duration of estrogen only MHT among current users (years)	5-<10	211/ 288	1.01 (0.78-1.31)		
rs4973768	Duration of estrogen only MHT among current users (years)	>=10	342/ 433	0.95 (0.78-1.16)		
rs4973768	Duration of estrogen only MHT among current users (years/5)	combined	6363/ 9072		0.96	1.9E-01
rs4973768	Mean lifetime intake of alcohol (g/day)	0	2266/ 2940	1.09 (1.01-1.18)		
rs4973768	Mean lifetime intake of alcohol (g/day)	>0-<10	3076/ 4912	1.11 (1.04-1.18)		
rs4973768	Mean lifetime intake of alcohol (g/day)	>=10-<20	636/ 1127	1.16 (1.01-1.33)		
rs4973768	Mean lifetime intake of alcohol (g/day)	>=20	525/ 787	1.08 (0.92-1.26)		
rs4973768	Mean lifetime intake of alcohol (10g/day)	combined	6503/ 9766		1.02	2.2E-01
rs4973768	Smoking (ever)	no	9156/ 9880	1.07 (1.02-1.11)		
rs4973768	Smoking (ever)	yes	7581/ 8383	1.14 (1.09-1.20)		
rs4973768	Smoking (ever/never)	combined	16737/18263		1.07	2.8E-02
rs4973768	Smoking (pack-years)	0	8469/ 9028	1.07 (1.03-1.12)		
rs4973768	Smoking (pack-years)	0-<10	2968/ 3647	1.10 (1.03-1.18)		
rs4973768	Smoking (pack-years)	10-<20	1423/ 1620	1.17 (1.06-1.30)		
rs4973768	Smoking (pack-years)	>=20	2011/ 2137	1.19 (1.09-1.30)		
rs4973768	Smoking (pack-years/10)	combined	14871/16432		1.03	4.2E-02
rs4973768	Physical activity during year before reference date (h/week)	0	1106/ 1072	1.12 (1.00-1.26)		
rs4973768	Physical activity during year before reference date (h/week)	0-<3.5	2347/ 2807	1.16 (1.08-1.26)		
rs4973768	Physical activity during year before reference date (h/week)	3.5-<7	1599/ 2211	1.07 (0.97-1.17)		
rs4973768	Physical activity during year before reference date (h/week)	>=7	1863/ 3683	1.15 (1.06-1.24)		
rs4973768	Physical activity during year before reference date (square root of h/week)	combined	6915/ 9773		1.00	9.6E-01
rs614367	Age at menarche (years)	<=11	2462/ 2358	1.22 (1.10-1.36)		
rs614367	Age at menarche (years)	12-13	7836/ 8055	1.27 (1.19-1.35)		

SNP	Variable	Stratum	N (cases/ controls)	OR (95% CI)[1]	OR int[2]	P int[3]
rs614367	Age at menarche (years)	>=14	6540/ 7651	1.16 (1.09-1.24)		
rs614367	Age at menarche (years/2)	combined	16838/18064		0.98	4.0E-01
rs614367	Parous	no	3366/ 3050	1.28 (1.17-1.41)		
rs614367	Parous	yes	16978/18334	1.19 (1.15-1.25)		
rs614367	Parous (yes/no)	combined	20344/21384		0.93	1.8E-01
rs614367	Number of births (among parous)	1	4069/ 3980	1.21 (1.11-1.31)		
rs614367	Number of births (among parous)	2	7213/ 7768	1.21 (1.13-1.28)		
rs614367	Number of births (among parous)	3	3410/ 3706	1.19 (1.09-1.31)		
rs614367	Number of births (among parous)	>=4	1833/ 2015	1.19 (1.05-1.34)		
rs614367	Number of births (among parous)	combined	16525/17469		0.99	5.3E-01
rs614367	Age at first birth (among parous, years)	<20	1507/ 1857	1.40 (1.22-1.59)		
rs614367	Age at first birth (among parous, years)	20-24	5787/ 6999	1.22 (1.14-1.30)		
rs614367	Age at first birth (among parous, years)	25-29	4558/ 4913	1.21 (1.12-1.31)		
rs614367	Age at first birth (among parous, years)	>=30	2291/ 2093	1.08 (0.97-1.22)		
rs614367	Age at first birth (among parous, years/5)	combined	14143/15862		0.94	9.1E-03
rs614367	Ever breastfed (among parous, yes/no)	no	2112/ 2304	1.31 (1.17-1.46)		
rs614367	Ever breastfed (among parous, yes/no)	yes	7190/ 9427	1.21 (1.14-1.28)		
rs614367	Ever breastfed (among parous, yes/no)	combined	9302/11731		0.92	2.2E-01
rs614367	Usual adult BMI, age<54	<25	2907/ 2720	1.18 (1.07-1.31)		
rs614367	Usual adult BMI, age<54	25-<30	933/ 880	1.24 (1.04-1.48)		
rs614367	Usual adult BMI, age<54	>=30	422/ 418	1.15 (0.89-1.49)		
rs614367	Usual adult BMI (BMI/5), age<54	combined	4262/ 4018		1.04	3.8E-01
rs614367	Usual adult BMI, age>=54	<25	3990/ 5948	1.32 (1.22-1.42)		
rs614367	Usual adult BMI, age>=54	25-<30	1816/ 2494	1.21 (1.07-1.36)		
rs614367	Usual adult BMI, age>=54	>=30	662/ 811	1.23 (1.01-1.51)		
rs614367	Usual adult BMI (BMI/5), age>=54	combined	6468/ 9253		0.94	1.2E-01
rs614367	Usual adult height (cm)	<160	4084/ 4895	1.30 (1.20-1.41)		
rs614367	Usual adult height (cm)	160-<165	4982/ 5892	1.14 (1.06-1.23)		
rs614367	Usual adult height (cm)	165-<170	4335/ 4851	1.22 (1.13-1.32)		
rs614367	Usual adult height (cm)	>=170	3110/ 3204	1.22 (1.11-1.34)		
rs614367	Usual adult height (cm/5)	combined	16511/18842		0.99	6.2E-01
rs614367	Ever use of oral contraceptives	no	6502/ 7377	1.26 (1.18-1.34)		
rs614367	Ever use of oral contraceptives	yes	7832/10179	1.21 (1.14-1.28)		
rs614367	Ever use of oral contraceptives (yes/no)	combined	14334/17556		0.96	3.2E-01
rs614367	Duration of oral contraceptive use (years)	0	6502/ 7377	1.26 (1.18-1.34)		
rs614367	Duration of oral contraceptive use (years)	>0-<5	2612/ 3377	1.18 (1.07-1.31)		
rs614367	Duration of oral contraceptive use (years)	5-<10	1913/ 2481	1.17 (1.04-1.32)		
rs614367	Duration of oral contraceptive use (years)	>=10	3109/ 4044	1.25 (1.14-1.37)		
rs614367	Duration of oral contraceptive use (years/5)	combined	14136/17279		0.99	7.0E-01
rs614367	Current use of combined estrogen/ progestagen MHT	never	2590/ 4073	1.27 (1.16-1.39)		
rs614367	Current use of combined estrogen/ progestagen MHT	EPCurrent	1085/ 1189	1.39 (1.19-1.63)		
rs614367	Current use of combined estrogen/ progestagen MHT (yes/no)	combined	5326/ 8117		1.10	2.8E-01
rs614367	Current use of estrogen only MHT	never	2732/ 4143	1.28 (1.17-1.40)		
rs614367	Current use of estrogen only MHT	ECurrent	615/ 916	1.38 (1.14-1.68)		
rs614367	Current use of estrogen only MHT (yes/no)	combined	5578/ 8280		1.08	4.4E-01
rs614367	Duration of combined estrogen/progestagen MHT among current users (years)	never	2590/ 4073	1.27 (1.16-1.39)		
rs614367	Duration of combined estrogen/progestagen MHT among current users (years)	>0-<5	240/ 314	1.54 (1.09-2.15)		
rs614367	Duration of combined estrogen/progestagen MHT among current users (years)	5-<10	348/ 353	1.37 (1.04-1.80)		

SNP	Variable	Stratum	N (cases/ controls)	OR (95% CI)[1]	OR int[2]	P int[3]
rs614367	Duration of combined estrogen/progestagen MHT among current users (years)	>=10	433/ 464	1.42 (1.11-1.83)		
rs614367	Duration of combined estrogen/progestagen MHT among current users (years/5)	combined	5239/ 8022		1.06	1.3E-01
rs614367	Duration of estrogen only MHT among current users (years)	never	2732/ 4143	1.28 (1.17-1.41)		
rs614367	Duration of estrogen only MHT among current users (years)	>0-<5	155/ 280	1.41 (0.98-2.02)		
rs614367	Duration of estrogen only MHT among current users (years)	5-<10	185/ 255	1.15 (0.78-1.70)		
rs614367	Duration of estrogen only MHT among current users (years)	>=10	258/ 358	1.51 (1.13-2.03)		
rs614367	Duration of estrogen only MHT among current users (years/5)	combined	5529/ 8205		1.03	4.9E-01
rs614367	Mean lifetime intake of alcohol (g/day)	0	2058/ 2800	1.26 (1.14-1.40)		
rs614367	Mean lifetime intake of alcohol (g/day)	>0-<10	2867/ 4659	1.23 (1.12-1.34)		
rs614367	Mean lifetime intake of alcohol (g/day)	>=10-<20	577/ 1060	1.36 (1.11-1.66)		
rs614367	Mean lifetime intake of alcohol (g/day)	>=20	481/ 742	1.26 (1.01-1.58)		
rs614367	Mean lifetime intake of alcohol (10g/day)	combined	5983/ 9261		1.01	6.6E-01
rs614367	Smoking (ever)	no	8579/ 9641	1.24 (1.17-1.31)		
rs614367	Smoking (ever)	yes	6893/ 7791	1.20 (1.12-1.27)		
rs614367	Smoking (ever/never)	combined	15472/17432		0.97	4.3E-01
rs614367	Smoking (pack-years)	0	7646/ 8465	1.23 (1.16-1.31)		
rs614367	Smoking (pack-years)	0-<10	2584/ 3368	1.17 (1.06-1.29)		
rs614367	Smoking (pack-years)	10-<20	1235/ 1473	1.16 (1.01-1.35)		
rs614367	Smoking (pack-years)	>=20	1686/ 1853	1.27 (1.12-1.45)		
rs614367	Smoking (pack-years/10)	combined	13151/15159		1.01	6.0E-01
rs614367	Physical activity during year before reference date (h/week)	0	643/ 680	1.14 (0.92-1.42)		
rs614367	Physical activity during year before reference date (h/week)	0-<3.5	1949/ 2410	1.33 (1.18-1.49)		
rs614367	Physical activity during year before reference date (h/week)	3.5-<7	1348/ 1969	1.26 (1.10-1.44)		
rs614367	Physical activity during year before reference date (h/week)	>=7	1608/ 3344	1.29 (1.16-1.45)		
rs614367	Physical activity during year before reference date (square root of h/week)	combined	5548/ 8403		1.00	9.7E-01
rs6504950	Age at menarche (years)	<=11	3994/ 3700	0.92 (0.85-0.99)		
rs6504950	Age at menarche (years)	12-13	11385/12333	0.94 (0.90-0.98)		
rs6504950	Age at menarche (years)	>=14	9480/12711	0.95 (0.91-0.99)		
rs6504950	Age at menarche (years/2)	combined	24859/28744		1.02	2.9E-01
rs6504950	Parous	no	4343/ 4200	0.91 (0.85-0.98)		
rs6504950	Parous	yes	24599/27832	0.94 (0.91-0.96)		
rs6504950	Parous (yes/no)	combined	28942/32032		1.03	4.8E-01
rs6504950	Number of births (among parous)	1	5398/ 5733	0.94 (0.89-1.00)		
rs6504950	Number of births (among parous)	2	11063/12902	0.93 (0.89-0.97)		
rs6504950	Number of births (among parous)	3	5082/ 5719	0.96 (0.90-1.02)		
rs6504950	Number of births (among parous)	>=4	2585/ 2866	0.93 (0.85-1.01)		
rs6504950	Number of births (among parous)	combined	24128/27220		1.00	8.5E-01
rs6504950	Age at first birth (among parous, years)	<20	2665/ 2802	0.96 (0.88-1.04)		
rs6504950	Age at first birth (among parous, years)	20-24	9080/11124	0.96 (0.92-1.01)		
rs6504950	Age at first birth (among parous, years)	25-29	6637/ 8202	0.90 (0.85-0.95)		
rs6504950	Age at first birth (among parous, years)	>=30	3266/ 3535	0.94 (0.87-1.02)		
rs6504950	Age at first birth (among parous, years/5)	combined	21648/25663		0.99	3.6E-01
rs6504950	Ever breastfed (among parous, yes/no)	no	2750/ 2839	0.98 (0.90-1.07)		
rs6504950	Ever breastfed (among parous, yes/no)	yes	8018/10042	0.92 (0.88-0.97)		
rs6504950	Ever breastfed (among parous, yes/no)	combined	10768/12881		0.94	1.9E-01
rs6504950	Usual adult BMI, age<54	<25	3233/ 2970	0.99 (0.91-1.07)		
rs6504950	Usual adult BMI, age<54	25-<30	1116/ 1001	0.96 (0.84-1.10)		
rs6504950	Usual adult BMI, age<54	>=30	509/ 493	0.98 (0.81-1.20)		

SNP	Variable	Stratum	N (cases/ controls)	OR (95% CI)[1]	OR int[2]	P int[3]
rs6504950	Usual adult BMI (BMI/5), age<54	combined	4858/ 4464		1.01	7.6E-01
rs6504950	Usual adult BMI, age>=54	<25	4450/ 6375	0.90 (0.85-0.96)		
rs6504950	Usual adult BMI, age>=54	25-<30	2089/ 2689	0.90 (0.82-0.99)		
rs6504950	Usual adult BMI, age>=54	>=30	846/ 907	0.90 (0.77-1.05)		
rs6504950	Usual adult BMI (BMI/5), age>=54	combined	7385/ 9971		0.99	8.2E-01
rs6504950	Usual adult height (cm)	<160	4461/ 5173	0.95 (0.89-1.01)		
rs6504950	Usual adult height (cm)	160-<165	5610/ 6257	0.92 (0.87-0.98)		
rs6504950	Usual adult height (cm)	165-<170	4758/ 5136	0.94 (0.88-1.00)		
rs6504950	Usual adult height (cm)	>=170	3497/ 3404	0.97 (0.90-1.05)		
rs6504950	Usual adult height (cm/5)	combined	18326/19970		1.00	9.5E-01
rs6504950	Ever use of oral contraceptives	no	7357/ 8070	0.94 (0.89-0.99)		
rs6504950	Ever use of oral contraceptives	yes	8689/10355	0.94 (0.90-0.99)		
rs6504950	Ever use of oral contraceptives (yes/no)	combined	16046/18425		1.01	8.6E-01
rs6504950	Duration of oral contraceptive use (years)	0	7357/ 8070	0.94 (0.89-0.99)		
rs6504950	Duration of oral contraceptive use (years)	>0-<5	3033/ 3444	0.99 (0.91-1.07)		
rs6504950	Duration of oral contraceptive use (years)	5-<10	2121/ 2530	0.92 (0.84-1.01)		
rs6504950	Duration of oral contraceptive use (years)	>=10	3318/ 4103	0.91 (0.84-0.98)		
rs6504950	Duration of oral contraceptive use (years/5)	combined	15829/18147		0.98	1.5E-01
rs6504950	Current use of combined estrogen/ progestagen MHT	never	2992/ 4514	0.88 (0.82-0.95)		
rs6504950	Current use of combined estrogen/ progestagen MHT	EPCurrent	1327/ 1373	0.86 (0.76-0.97)		
rs6504950	Current use of combined estrogen/ progestagen MHT (yes/no)	combined	6224/ 9028		0.96	5.4E-01
rs6504950	Current use of estrogen only MHT	never	3132/ 4590	0.89 (0.83-0.96)		
rs6504950	Current use of estrogen only MHT	ECurrent	767/ 1067	1.00 (0.86-1.16)		
rs6504950	Current use of estrogen only MHT (yes/no)	combined	6477/ 9209		1.13	1.2E-01
rs6504950	Duration of combined estrogen/progestagen MHT among current users (years)	never	2992/ 4514	0.88 (0.82-0.95)		
rs6504950	Duration of combined estrogen/progestagen MHT among current users (years)	>0-<5	299/ 376	0.73 (0.57-0.94)		
rs6504950	Duration of combined estrogen/progestagen MHT among current users (years)	5-<10	439/ 415	0.80 (0.64-1.00)		
rs6504950	Duration of combined estrogen/progestagen MHT among current users (years)	>=10	525/ 525	0.94 (0.77-1.14)		
rs6504950	Duration of combined estrogen/progestagen MHT among current users (years/5)	combined	6136/ 8934		1.00	9.3E-01
rs6504950	Duration of estrogen only MHT among current users (years)	never	3132/ 4590	0.89 (0.83-0.96)		
rs6504950	Duration of estrogen only MHT among current users (years)	>0-<5	173/ 303	1.07 (0.80-1.43)		
rs6504950	Duration of estrogen only MHT among current users (years)	5-<10	211/ 288	0.84 (0.63-1.13)		
rs6504950	Duration of estrogen only MHT among current users (years)	>=10	347/ 437	1.03 (0.82-1.30)		
rs6504950	Duration of estrogen only MHT among current users (years/5)	combined	6392/ 9092		1.04	2.7E-01
rs6504950	Mean lifetime intake of alcohol (g/day)	0	2278/ 2945	0.95 (0.87-1.04)		
rs6504950	Mean lifetime intake of alcohol (g/day)	>0-<10	3091/ 4928	0.93 (0.87-1.00)		
rs6504950	Mean lifetime intake of alcohol (g/day)	>=10-<20	638/ 1129	0.85 (0.72-0.99)		
rs6504950	Mean lifetime intake of alcohol (g/day)	>=20	523/ 794	0.98 (0.82-1.17)		
rs6504950	Mean lifetime intake of alcohol (10g/day)	combined	6530/ 9796		1.01	5.2E-01
rs6504950	Smoking (ever)	no	9385/10012	0.94 (0.89-0.98)		
rs6504950	Smoking (ever)	yes	7701/ 8375	0.93 (0.89-0.98)		
rs6504950	Smoking (ever/never)	combined	17086/18387		1.00	9.1E-01
rs6504950	Smoking (pack-years)	0	8435/ 8830	0.94 (0.89-0.98)		
rs6504950	Smoking (pack-years)	0-<10	2936/ 3548	0.94 (0.86-1.01)		
rs6504950	Smoking (pack-years)	10-<20	1405/ 1599	0.94 (0.83-1.05)		
rs6504950	Smoking (pack-years)	>=20	2022/ 2095	0.94 (0.85-1.04)		
rs6504950	Smoking (pack-years/10)	combined	14798/16072		1.00	7.7E-01

SNP	Variable	Stratum	N (cases/ controls)	OR (95% CI)[1]	OR int[2]	P int[3]
rs6504950	Physical activity during year before reference date (h/week)	0	1105/ 1064	0.90 (0.79-1.03)		
rs6504950	Physical activity during year before reference date (h/week)	0-<3.5	2368/ 2818	0.93 (0.86-1.02)		
rs6504950	Physical activity during year before reference date (h/week)	3.5-<7	1611/ 2223	0.86 (0.77-0.95)		
rs6504950	Physical activity during year before reference date (h/week)	>=7	1867/ 3681	0.91 (0.83-0.99)		
rs6504950	Physical activity during year before reference date (square root of h/week)	combined	6951/ 9786		0.99	6.8E-01
rs704010	Age at menarche (years)	<=11	2575/ 2918	1.09 (1.01-1.18)		
rs704010	Age at menarche (years)	12-13	8537/10806	1.03 (0.99-1.08)		
rs704010	Age at menarche (years)	>=14	7459/10866	1.05 (1.01-1.10)		
rs704010	Age at menarche (years/2)	combined	18571/24590		1.00	8.5E-01
rs704010	Parous	no	3638/ 3814	1.10 (1.03-1.18)		
rs704010	Parous	yes	18879/24165	1.05 (1.02-1.08)		
rs704010	Parous (yes/no)	combined	22517/27979		0.95	2.2E-01
rs704010	Number of births (among parous)	1	4447/ 5140	1.03 (0.97-1.09)		
rs704010	Number of births (among parous)	2	8229/11018	1.06 (1.01-1.10)		
rs704010	Number of births (among parous)	3	3769/ 4815	1.05 (0.99-1.12)		
rs704010	Number of births (among parous)	>=4	1925/ 2319	1.05 (0.96-1.15)		
rs704010	Number of births (among parous)	combined	18370/23292		1.01	7.0E-01
rs704010	Age at first birth (among parous, years)	<20	1717/ 2477	1.07 (0.98-1.18)		
rs704010	Age at first birth (among parous, years)	20-24	6527/ 9293	1.05 (1.00-1.10)		
rs704010	Age at first birth (among parous, years)	25-29	5172/ 6910	1.03 (0.98-1.09)		
rs704010	Age at first birth (among parous, years)	>=30	2579/ 2997	0.97 (0.90-1.06)		
rs704010	Age at first birth (among parous, years/5)	combined	15995/21677		0.98	1.8E-01
rs704010	Ever breastfed (among parous, yes/no)	no	2105/ 2296	1.15 (1.05-1.25)		
rs704010	Ever breastfed (among parous, yes/no)	yes	7183/ 9410	1.01 (0.97-1.06)		
rs704010	Ever breastfed (among parous, yes/no)	combined	9288/11706		0.88	1.6E-02
rs704010	Usual adult BMI, age<54	<25	2908/ 2719	1.04 (0.97-1.13)		
rs704010	Usual adult BMI, age<54	25-<30	932/ 879	1.09 (0.95-1.25)		
rs704010	Usual adult BMI, age<54	>=30	418/ 413	1.14 (0.93-1.39)		
rs704010	Usual adult BMI (BMI/5), age<54	combined	4258/ 4011		1.04	2.3E-01
rs704010	Usual adult BMI, age>=54	<25	3986/ 5940	1.04 (0.98-1.10)		
rs704010	Usual adult BMI, age>=54	25-<30	1808/ 2486	1.03 (0.94-1.13)		
rs704010	Usual adult BMI, age>=54	>=30	656/ 806	0.96 (0.81-1.13)		
rs704010	Usual adult BMI (BMI/5), age>=54	combined	6450/ 9232		0.97	3.7E-01
rs704010	Usual adult height (cm)	<160	4081/ 4892	1.04 (0.98-1.11)		
rs704010	Usual adult height (cm)	160-<165	4991/ 5891	1.06 (1.00-1.12)		
rs704010	Usual adult height (cm)	165-<170	4331/ 4848	1.02 (0.96-1.09)		
rs704010	Usual adult height (cm)	>=170	3112/ 3199	1.08 (1.00-1.16)		
rs704010	Usual adult height (cm/5)	combined	16515/18830		1.00	8.0E-01
rs704010	Ever use of oral contraceptives	no	6503/ 7376	1.07 (1.01-1.12)		
rs704010	Ever use of oral contraceptives	yes	7855/10185	1.04 (1.00-1.09)		
rs704010	Ever use of oral contraceptives (yes/no)	combined	14358/17561		0.98	4.9E-01
rs704010	Duration of oral contraceptive use (years)	0	6503/ 7376	1.06 (1.01-1.12)		
rs704010	Duration of oral contraceptive use (years)	>0-<5	2617/ 3383	1.01 (0.93-1.09)		
rs704010	Duration of oral contraceptive use (years)	5-<10	1919/ 2477	1.07 (0.98-1.17)		
rs704010	Duration of oral contraceptive use (years)	>=10	3118/ 4043	1.07 (1.00-1.14)		
rs704010	Duration of oral contraceptive use (years/5)	combined	14157/17279		1.00	9.3E-01
rs704010	Current use of combined estrogen/ progestagen MHT	never	2586/ 4067	1.09 (1.02-1.18)		
rs704010	Current use of combined estrogen/ progestagen MHT	EPCurrent	1086/ 1187	1.04 (0.92-1.18)		
rs704010	Current use of combined estrogen/ progestagen MHT (yes/no)	combined	5323/ 8109		1.00	9.8E-01

SNP	Variable	Stratum	N (cases/ controls)	OR (95% CI)[1]	OR int[2]	P int[3]
rs704010	Current use of estrogen only MHT	never	2728/ 4138	1.09 (1.02-1.17)		
rs704010	Current use of estrogen only MHT	ECurrent	615/ 916	1.11 (0.96-1.29)		
rs704010	Current use of estrogen only MHT (yes/no)	combined	5575/ 8272		1.08	3.7E-01
rs704010	Duration of combined estrogen/progestagen MHT among current users (years)	never	2586/ 4067	1.09 (1.02-1.18)		
rs704010	Duration of combined estrogen/progestagen MHT among current users (years)	>0-<5	241/ 313	1.07 (0.83-1.38)		
rs704010	Duration of combined estrogen/progestagen MHT among current users (years)	5-<10	348/ 353	0.95 (0.76-1.20)		
rs704010	Duration of combined estrogen/progestagen MHT among current users (years)	>=10	433/ 463	1.08 (0.89-1.31)		
rs704010	Duration of combined estrogen/progestagen MHT among current users (years/5)	combined	5236/ 8014		1.01	6.4E-01
rs704010	Duration of estrogen only MHT among current users (years)	never	2728/ 4138	1.09 (1.02-1.17)		
rs704010	Duration of estrogen only MHT among current users (years)	>0-<5	155/ 279	1.19 (0.90-1.59)		
rs704010	Duration of estrogen only MHT among current users (years)	5-<10	184/ 255	1.06 (0.80-1.40)		
rs704010	Duration of estrogen only MHT among current users (years)	>=10	259/ 359	1.08 (0.86-1.37)		
rs704010	Duration of estrogen only MHT among current users (years/5)	combined	5526/ 8197		1.01	8.4E-01
rs704010	Mean lifetime intake of alcohol (g/day)	0	2058/ 2802	1.08 (0.99-1.17)		
rs704010	Mean lifetime intake of alcohol (g/day)	>0-<10	2863/ 4653	1.04 (0.97-1.11)		
rs704010	Mean lifetime intake of alcohol (g/day)	>=10-<20	578/ 1060	1.08 (0.93-1.25)		
rs704010	Mean lifetime intake of alcohol (g/day)	>=20	483/ 741	0.98 (0.82-1.17)		
rs704010	Mean lifetime intake of alcohol (10g/day)	combined	5982/ 9256		0.98	3.5E-01
rs704010	Smoking (ever)	no	8585/ 9642	1.09 (1.05-1.14)		
rs704010	Smoking (ever)	yes	6899/ 7785	1.02 (0.97-1.07)		
rs704010	Smoking (ever/never)	combined	15484/17427		0.93	3.8E-02
rs704010	Smoking (pack-years)	0	7652/ 8468	1.09 (1.04-1.15)		
rs704010	Smoking (pack-years)	0-<10	2584/ 3371	1.03 (0.95-1.11)		
rs704010	Smoking (pack-years)	10-<20	1239/ 1474	0.96 (0.86-1.07)		
rs704010	Smoking (pack-years)	>=20	1691/ 1846	1.10 (0.99-1.21)		
rs704010	Smoking (pack-years/10)	combined	13166/15159		0.99	4.5E-01
rs704010	Physical activity during year before reference date (h/week)	0	642/ 678	0.91 (0.78-1.07)		
rs704010	Physical activity during year before reference date (h/week)	0-<3.5	1949/ 2413	1.07 (0.98-1.16)		
rs704010	Physical activity during year before reference date (h/week)	3.5-<7	1348/ 1966	0.98 (0.89-1.09)		
rs704010	Physical activity during year before reference date (h/week)	>=7	1608/ 3342	1.10 (1.01-1.20)		
rs704010	Physical activity during year before reference date (square root of h/week)	combined	5547/ 8399		1.02	2.2E-01
rs865686	Age at menarche (years)	<=11	3791/ 3572	0.85 (0.79-0.91)		
rs865686	Age at menarche (years)	12-13	10600/11574	0.89 (0.86-0.93)		
rs865686	Age at menarche (years)	>=14	8467/11081	0.92 (0.88-0.96)		
rs865686	Age at menarche (years/2)	combined	22858/26227		1.02	2.0E-01
rs865686	Parous	no	4190/ 4121	0.90 (0.84-0.96)		
rs865686	Parous	yes	22856/25672	0.90 (0.88-0.93)		
rs865686	Parous (yes/no)	combined	27046/29793		1.00	9.2E-01
rs865686	Number of births (among parous)	1	4673/ 5006	0.94 (0.88-1.00)		
rs865686	Number of births (among parous)	2	10379/11760	0.89 (0.85-0.92)		
rs865686	Number of births (among parous)	3	4912/ 5381	0.94 (0.88-1.00)		
rs865686	Number of births (among parous)	>=4	2412/ 2657	0.87 (0.80-0.94)		
rs865686	Number of births (among parous)	combined	22376/24804		0.99	5.8E-01
rs865686	Age at first birth (among parous, years)	<20	2418/ 2406	1.01 (0.92-1.10)		
rs865686	Age at first birth (among parous, years)	20-24	8052/ 9712	0.89 (0.85-0.93)		
rs865686	Age at first birth (among parous, years)	25-29	6292/ 7727	0.88 (0.84-0.93)		

SNP	Variable	Stratum	N (cases/ controls)	OR (95% CI)[1]	OR int[2]	P int[3]
rs865686	Age at first birth (among parous, years)	>=30	3063/ 3325	0.88 (0.82-0.95)		
rs865686	Age at first birth (among parous, years/5)	combined	19825/23170		0.97	4.8E-02
rs865686	Ever breastfed (among parous, yes/no)	no	1743/ 1895	0.85 (0.77-0.94)		
rs865686	Ever breastfed (among parous, yes/no)	yes	5910/ 7795	0.91 (0.87-0.96)		
rs865686	Ever breastfed (among parous, yes/no)	combined	7653/ 9690		1.07	2.3E-01
rs865686	Usual adult BMI, age<54	<25	2178/ 1921	0.84 (0.76-0.92)		
rs865686	Usual adult BMI, age<54	25-<30	801/ 728	0.88 (0.75-1.02)		
rs865686	Usual adult BMI, age<54	>=30	398/ 400	0.89 (0.72-1.10)		
rs865686	Usual adult BMI (BMI/5), age<54	combined	3377/ 3049		1.02	6.7E-01
rs865686	Usual adult BMI, age>=54	<25	3239/ 4973	0.92 (0.86-0.98)		
rs865686	Usual adult BMI, age>=54	25-<30	1561/ 2174	0.90 (0.82-1.00)		
rs865686	Usual adult BMI, age>=54	>=30	625/ 766	0.93 (0.79-1.10)		
rs865686	Usual adult BMI (BMI/5), age>=54	combined	5425/ 7913		1.02	5.2E-01
rs865686	Usual adult height (cm)	<160	3507/ 4115	0.86 (0.80-0.92)		
rs865686	Usual adult height (cm)	160-<165	4267/ 5003	0.92 (0.87-0.98)		
rs865686	Usual adult height (cm)	165-<170	3966/ 4409	0.93 (0.87-0.99)		
rs865686	Usual adult height (cm)	>=170	2892/ 2983	0.86 (0.80-0.93)		
rs865686	Usual adult height (cm/5)	combined	14632/16510		1.01	4.4E-01
rs865686	Ever use of oral contraceptives	no	4953/ 5440	0.88 (0.83-0.93)		
rs865686	Ever use of oral contraceptives	yes	7532/ 9780	0.90 (0.86-0.94)		
rs865686	Ever use of oral contraceptives (yes/no)	combined	12485/15220		1.02	5.8E-01
rs865686	Duration of oral contraceptive use (years)	0	4953/ 5440	0.88 (0.83-0.93)		
rs865686	Duration of oral contraceptive use (years)	>0-<5	2463/ 3184	0.91 (0.84-0.98)		
rs865686	Duration of oral contraceptive use (years)	5-<10	1856/ 2409	0.87 (0.80-0.96)		
rs865686	Duration of oral contraceptive use (years)	>=10	3027/ 3926	0.91 (0.85-0.98)		
rs865686	Duration of oral contraceptive use (years/5)	combined	12299/14959		1.00	7.2E-01
rs865686	Current use of combined estrogen/ progestagen MHT	never	1872/ 3082	0.93 (0.86-1.02)		
rs865686	Current use of combined estrogen/ progestagen MHT	EPCurrent	988/ 1112	0.94 (0.82-1.06)		
rs865686	Current use of combined estrogen/ progestagen MHT (yes/no)	combined	4337/ 6820		1.03	7.3E-01
rs865686	Current use of estrogen only MHT	never	2013/ 3152	0.95 (0.87-1.03)		
rs865686	Current use of estrogen only MHT	ECurrent	585/ 875	0.97 (0.83-1.14)		
rs865686	Current use of estrogen only MHT (yes/no)	combined	4587/ 6985		1.07	4.4E-01
rs865686	Duration of combined estrogen/progestagen MHT among current users (years)	never	1872/ 3082	0.93 (0.86-1.02)		
rs865686	Duration of combined estrogen/progestagen MHT among current users (years)	>0-<5	200/ 277	0.94 (0.72-1.23)		
rs865686	Duration of combined estrogen/progestagen MHT among current users (years)	5-<10	302/ 330	0.94 (0.75-1.19)		
rs865686	Duration of combined estrogen/progestagen MHT among current users (years)	>=10	424/ 450	0.95 (0.78-1.16)		
rs865686	Duration of combined estrogen/progestagen MHT among current users (years/5)	combined	4252/ 6728		1.03	3.3E-01
rs865686	Duration of estrogen only MHT among current users (years)	never	2013/ 3152	0.95 (0.87-1.03)		
rs865686	Duration of estrogen only MHT among current users (years)	>0-<5	147/ 263	0.98 (0.72-1.32)		
rs865686	Duration of estrogen only MHT among current users (years)	5-<10	179/ 240	1.08 (0.81-1.45)		
rs865686	Duration of estrogen only MHT among current users (years)	>=10	243/ 351	0.92 (0.71-1.18)		
rs865686	Duration of estrogen only MHT among current users (years/5)	combined	4541/ 6914		1.01	8.6E-01
rs865686	Mean lifetime intake of alcohol (g/day)	0	929/ 1380	0.95 (0.84-1.08)		
rs865686	Mean lifetime intake of alcohol (g/day)	>0-<10	2297/ 3999	0.89 (0.82-0.96)		
rs865686	Mean lifetime intake of alcohol (g/day)	>=10-<20	499/ 964	0.98 (0.83-1.14)		
rs865686	Mean lifetime intake of alcohol (g/day)	>=20	445/ 693	0.85 (0.71-1.01)		
rs865686	Mean lifetime intake of alcohol (10g/day)	combined	4170/ 7036		0.98	3.8E-01

SNP	Variable	Stratum	N (cases/ controls)	OR (95% CI)[1]	OR int[2]	P int[3]
rs865686	Smoking (ever)	no	7766/ 8586	0.87 (0.83-0.91)		
rs865686	Smoking (ever)	yes	5829/ 6552	0.94 (0.89-0.99)		
rs865686	Smoking (ever/never)	combined	13595/15138		1.08	3.4E-02
rs865686	Smoking (pack-years)	0	6820/ 7418	0.87 (0.83-0.92)		
rs865686	Smoking (pack-years)	0-<10	1966/ 2671	0.95 (0.87-1.04)		
rs865686	Smoking (pack-years)	10-<20	1023/ 1199	0.96 (0.84-1.09)		
rs865686	Smoking (pack-years)	>=20	1472/ 1600	0.90 (0.81-1.01)		
rs865686	Smoking (pack-years/10)	combined	11281/12888		1.01	4.7E-01
rs865686	Physical activity during year before reference date (h/week)	0	636/ 671	0.86 (0.73-1.01)		
rs865686	Physical activity during year before reference date (h/week)	0-<3.5	1915/ 2375	0.86 (0.78-0.94)		
rs865686	Physical activity during year before reference date (h/week)	3.5-<7	1299/ 1942	0.92 (0.83-1.02)		
rs865686	Physical activity during year before reference date (h/week)	>=7	1579/ 3241	0.94 (0.86-1.03)		
rs865686	Physical activity during year before reference date (square root of h/week)	combined	5429/ 8229		1.02	4.1E-01
rs889312	Age at menarche (years)	<=11	4005/ 3407	1.13 (1.05-1.21)		
rs889312	Age at menarche (years)	12-13	11058/10500	1.09 (1.05-1.14)		
rs889312	Age at menarche (years)	>=14	8726/ 9970	1.12 (1.07-1.17)		
rs889312	Age at menarche (years/2)	combined	23789/23877		1.00	9.2E-01
rs889312	Parous	no	4192/ 3512	1.11 (1.04-1.20)		
rs889312	Parous	yes	23380/23500	1.11 (1.08-1.14)		
rs889312	Parous (yes/no)	combined	27572/27012		1.00	9.4E-01
rs889312	Number of births (among parous)	1	4976/ 4631	1.06 (0.99-1.13)		
rs889312	Number of births (among parous)	2	10565/10499	1.14 (1.09-1.19)		
rs889312	Number of births (among parous)	3	4977/ 4998	1.12 (1.05-1.19)		
rs889312	Number of births (among parous)	>=4	2579/ 2723	1.08 (0.99-1.17)		
rs889312	Number of births (among parous)	combined	23097/22851		1.00	9.0E-01
rs889312	Age at first birth (among parous, years)	<20	2526/ 2280	1.15 (1.05-1.26)		
rs889312	Age at first birth (among parous, years)	20-24	8703/ 9480	1.13 (1.08-1.18)		
rs889312	Age at first birth (among parous, years)	25-29	6338/ 6690	1.11 (1.05-1.18)		
rs889312	Age at first birth (among parous, years)	>=30	3062/ 2783	1.04 (0.96-1.13)		
rs889312	Age at first birth (among parous, years/5)	combined	20629/21233		0.98	1.9E-01
rs889312	Ever breastfed (among parous, yes/no)	no	2465/ 2473	1.09 (1.00-1.19)		
rs889312	Ever breastfed (among parous, yes/no)	yes	7064/ 8517	1.12 (1.07-1.18)		
rs889312	Ever breastfed (among parous, yes/no)	combined	9529/10990		1.03	5.4E-01
rs889312	Usual adult BMI, age<54	<25	3056/ 2771	1.13 (1.04-1.23)		
rs889312	Usual adult BMI, age<54	25-<30	1019/ 941	1.07 (0.93-1.24)		
rs889312	Usual adult BMI, age<54	>=30	453/ 454	1.20 (0.97-1.49)		
rs889312	Usual adult BMI (BMI/5), age<54	combined	4528/ 4166		1.00	1.0E+00
rs889312	Usual adult BMI, age>=54	<25	3641/ 4812	1.12 (1.05-1.20)		
rs889312	Usual adult BMI, age>=54	25-<30	1824/ 2300	1.12 (1.01-1.23)		
rs889312	Usual adult BMI, age>=54	>=30	772/ 810	1.10 (0.94-1.30)		
rs889312	Usual adult BMI (BMI/5), age>=54	combined	6237/ 7922		0.99	7.1E-01
rs889312	Usual adult height (cm)	<160	4169/ 4818	1.13 (1.06-1.21)		
rs889312	Usual adult height (cm)	160-<165	5096/ 5487	1.07 (1.00-1.13)		
rs889312	Usual adult height (cm)	165-<170	4226/ 4378	1.16 (1.09-1.25)		
rs889312	Usual adult height (cm)	>=170	3041/ 2803	1.07 (0.98-1.16)		
rs889312	Usual adult height (cm/5)	combined	16532/17486		0.99	4.6E-01
rs889312	Ever use of oral contraceptives	no	6961/ 7370	1.12 (1.07-1.18)		
rs889312	Ever use of oral contraceptives	yes	7613/ 8656	1.10 (1.05-1.15)		
rs889312	Ever use of oral contraceptives (yes/no)	combined	14574/16026		0.98	5.5E-01

SNP	Variable	Stratum	N (cases/ controls)	OR (95% CI)[1]	OR int[2]	P int[3]
rs889312	Duration of oral contraceptive use (years)	0	6961/ 7370	1.12 (1.07-1.18)		
rs889312	Duration of oral contraceptive use (years)	>0-<5	2750/ 2994	1.14 (1.05-1.24)		
rs889312	Duration of oral contraceptive use (years)	5-<10	1863/ 2124	1.08 (0.98-1.20)		
rs889312	Duration of oral contraceptive use (years)	>=10	2787/ 3271	1.07 (0.99-1.16)		
rs889312	Duration of oral contraceptive use (years/5)	combined	14361/15759		0.98	2.6E-01
rs889312	Current use of combined estrogen/ progestagen MHT	never	2632/ 3776	1.09 (1.01-1.18)		
rs889312	Current use of combined estrogen/ progestagen MHT	EPCurrent	984/ 923	1.21 (1.04-1.39)		
rs889312	Current use of combined estrogen/ progestagen MHT (yes/no)	combined	5102/ 6950		1.10	2.5E-01
rs889312	Current use of estrogen only MHT	never	2778/ 3853	1.09 (1.01-1.18)		
rs889312	Current use of estrogen only MHT	ECurrent	649/ 802	1.18 (1.00-1.39)		
rs889312	Current use of estrogen only MHT (yes/no)	combined	5359/ 7117		1.06	4.9E-01
rs889312	Duration of combined estrogen/progestagen MHT among current users (years)	never	2632/ 3776	1.09 (1.01-1.18)		
rs889312	Duration of combined estrogen/progestagen MHT among current users (years)	>0-<5	248/ 292	1.13 (0.86-1.49)		
rs889312	Duration of combined estrogen/progestagen MHT among current users (years)	5-<10	338/ 280	1.26 (0.98-1.63)		
rs889312	Duration of combined estrogen/progestagen MHT among current users (years)	>=10	339/ 300	1.11 (0.87-1.43)		
rs889312	Duration of combined estrogen/progestagen MHT among current users (years/5)	combined	5023/ 6865		1.04	3.8E-01
rs889312	Duration of estrogen only MHT among current users (years)	never	2778/ 3853	1.09 (1.01-1.18)		
rs889312	Duration of estrogen only MHT among current users (years)	>0-<5	156/ 235	1.15 (0.84-1.58)		
rs889312	Duration of estrogen only MHT among current users (years)	5-<10	178/ 213	1.33 (0.97-1.82)		
rs889312	Duration of estrogen only MHT among current users (years)	>=10	280/ 318	1.21 (0.93-1.58)		
rs889312	Duration of estrogen only MHT among current users (years/5)	combined	5275/ 7010		1.03	4.8E-01
rs889312	Mean lifetime intake of alcohol (g/day)	0	2237/ 2807	1.11 (1.02-1.22)		
rs889312	Mean lifetime intake of alcohol (g/day)	>0-<10	2531/ 3719	1.10 (1.02-1.19)		
rs889312	Mean lifetime intake of alcohol (g/day)	>=10-<20	513/ 775	1.18 (0.99-1.40)		
rs889312	Mean lifetime intake of alcohol (g/day)	>=20	406/ 544	1.16 (0.95-1.42)		
rs889312	Mean lifetime intake of alcohol (10g/day)	combined	5687/ 7845		1.02	5.4E-01
rs889312	Smoking (ever)	no	8416/ 8828	1.11 (1.06-1.17)		
rs889312	Smoking (ever)	yes	6887/ 7115	1.10 (1.04-1.16)		
rs889312	Smoking (ever/never)	combined	15303/15943		0.99	7.2E-01
rs889312	Smoking (pack-years)	0	7458/ 7637	1.11 (1.05-1.16)		
rs889312	Smoking (pack-years)	0-<10	2642/ 3024	1.13 (1.04-1.23)		
rs889312	Smoking (pack-years)	10-<20	1229/ 1308	1.19 (1.05-1.35)		
rs889312	Smoking (pack-years)	>=20	1763/ 1692	1.07 (0.97-1.20)		
rs889312	Smoking (pack-years/10)	combined	13092/13661		0.99	6.8E-01
rs889312	Physical activity during year before reference date (h/week)	0	1094/ 1059	1.17 (1.02-1.33)		
rs889312	Physical activity during year before reference date (h/week)	0-<3.5	2093/ 2480	1.08 (0.99-1.18)		
rs889312	Physical activity during year before reference date (h/week)	3.5-<7	1256/ 1708	1.12 (1.00-1.26)		
rs889312	Physical activity during year before reference date (h/week)	>=7	1124/ 2104	1.12 (1.01-1.26)		
rs889312	Physical activity during year before reference date (square root of h/week)	combined	5567/ 7351		0.98	4.4E-01
rs999737[8]	Age at menarche (years)	<=11	4002/ 3486	0.92 (0.85-1.00)		
rs999737	Age at menarche (years)	12-13	11214/11398	0.97 (0.93-1.02)		
rs999737	Age at menarche (years)	>=14	9000/11067	0.92 (0.87-0.96)		
rs999737	Age at menarche (years/2)	combined	24216/25951		0.99	5.5E-01
rs999737	Parous	no	4205/ 3775	0.87 (0.80-0.94)		
rs999737	Parous	yes	23958/25161	0.95 (0.92-0.98)		
rs999737	Parous (yes/no)	combined	28163/28936		1.09	3.6E-02

SNP	Variable	Stratum	N (cases/ controls)	OR (95% CI)[1]	OR int[2]	P int[3]
rs999737	Number of births (among parous)	1	5030/ 4849	0.93 (0.87-1.00)		
rs999737	Number of births (among parous)	2	10694/11402	0.97 (0.93-1.02)		
rs999737	Number of births (among parous)	3	4933/ 5135	0.91 (0.85-0.97)		
rs999737	Number of births (among parous)	>=4	2496/ 2573	0.98 (0.89-1.08)		
rs999737	Number of births (among parous)	combined	23153/23959		1.00	9.7E-01
rs999737	Age at first birth (among parous, years)	<20	2569/ 2385	0.93 (0.85-1.03)		
rs999737	Age at first birth (among parous, years)	20-24	8720/ 9682	0.96 (0.91-1.01)		
rs999737	Age at first birth (among parous, years)	25-29	6477/ 7344	0.96 (0.90-1.01)		
rs999737	Age at first birth (among parous, years)	>=30	3156/ 3243	0.96 (0.88-1.05)		
rs999737	Age at first birth (among parous, years/5)	combined	20922/22654		0.99	6.1E-01
rs999737	Ever breastfed (among parous, yes/no)	no	2573/ 2253	1.00 (0.91-1.10)		
rs999737	Ever breastfed (among parous, yes/no)	yes	7399/ 7767	0.93 (0.88-0.98)		
rs999737	Ever breastfed (among parous, yes/no)	combined	9972/10020		0.93	1.7E-01
rs999737	Usual adult BMI, age<54	<25	3367/ 2862	0.96 (0.88-1.05)		
rs999737	Usual adult BMI, age<54	25-<30	1139/ 989	0.89 (0.77-1.03)		
rs999737	Usual adult BMI, age<54	>=30	538/ 497	0.88 (0.72-1.08)		
rs999737	Usual adult BMI (BMI/5), age<54	combined	5044/ 4348		0.93	5.1E-02
rs999737	Usual adult BMI, age>=54	<25	3679/ 3954	0.93 (0.86-1.00)		
rs999737	Usual adult BMI, age>=54	25-<30	1816/ 1988	1.01 (0.90-1.13)		
rs999737	Usual adult BMI, age>=54	>=30	804/ 822	0.87 (0.74-1.03)		
rs999737	Usual adult BMI (BMI/5), age>=54	combined	6299/ 6764		1.00	9.0E-01
rs999737	Usual adult height (cm)	<160	4156/ 4444	0.92 (0.85-0.99)		
rs999737	Usual adult height (cm)	160-<165	5099/ 5155	0.95 (0.89-1.01)		
rs999737	Usual adult height (cm)	165-<170	4331/ 4091	0.94 (0.87-1.01)		
rs999737	Usual adult height (cm)	>=170	3158/ 2706	0.96 (0.88-1.05)		
rs999737	Usual adult height (cm/5)	combined	16744/16396		1.02	1.8E-01
rs999737	Ever use of oral contraceptives	no	6746/ 6663	0.95 (0.89-1.00)		
rs999737	Ever use of oral contraceptives	yes	8061/ 8157	0.94 (0.90-0.99)		
rs999737	Ever use of oral contraceptives (yes/no)	combined	14807/14820		1.00	9.3E-01
rs999737	Duration of oral contraceptive use (years)	0	6746/ 6663	0.95 (0.89-1.00)		
rs999737	Duration of oral contraceptive use (years)	>0-<5	2812/ 2778	0.95 (0.87-1.04)		
rs999737	Duration of oral contraceptive use (years)	5-<10	1997/ 2016	0.99 (0.89-1.10)		
rs999737	Duration of oral contraceptive use (years)	>=10	3058/ 3112	0.90 (0.83-0.99)		
rs999737	Duration of oral contraceptive use (years/5)	combined	14613/14569		0.99	6.3E-01
rs999737	Current use of combined estrogen/ progestagen MHT	never	2547/ 3115	0.94 (0.86-1.03)		
rs999737	Current use of combined estrogen/ progestagen MHT	EPCurrent	1012/ 755	0.91 (0.77-1.07)		
rs999737	Current use of combined estrogen/ progestagen MHT (yes/no)	combined	5104/ 5600		0.94	5.2E-01
rs999737	Current use of estrogen only MHT	never	2691/ 3196	0.94 (0.86-1.02)		
rs999737	Current use of estrogen only MHT	ECurrent	649/ 640	1.12 (0.93-1.35)		
rs999737	Current use of estrogen only MHT (yes/no)	combined	5356/ 5779		1.19	7.6E-02
rs999737	Duration of combined estrogen/progestagen MHT among current users (years)	never	2547/ 3115	0.94 (0.86-1.03)		
rs999737	Duration of combined estrogen/progestagen MHT among current users (years)	>0-<5	226/ 234	0.98 (0.71-1.35)		
rs999737	Duration of combined estrogen/progestagen MHT among current users (years)	5-<10	348/ 229	0.89 (0.68-1.17)		
rs999737	Duration of combined estrogen/progestagen MHT among current users (years)	>=10	374/ 241	0.80 (0.61-1.06)		
rs999737	Duration of combined estrogen/progestagen MHT among current users (years/5)	combined	5016/ 5516		0.97	4.5E-01
rs999737	Duration of estrogen only MHT among current users (years)	never	2691/ 3196	0.94 (0.86-1.02)		
rs999737	Duration of estrogen only MHT among current users (years)	>0-<5	133/ 175	0.79 (0.52-1.19)		

SNP	Variable	Stratum	N (cases/ controls)	OR (95% CI)[1]	OR int[2]	P int[3]
rs999737	Duration of estrogen only MHT among current users (years)	5-<10	181/ 173	0.95 (0.66-1.37)		
rs999737	Duration of estrogen only MHT among current users (years)	>=10	300/ 258	1.39 (1.06-1.83)		
rs999737	Duration of estrogen only MHT among current users (years/5)	combined	5271/ 5667		1.13	4.0E-03
rs999737	Mean lifetime intake of alcohol (g/day)	0	2133/ 2533	0.96 (0.87-1.06)		
rs999737	Mean lifetime intake of alcohol (g/day)	>0-<10	2553/ 2864	0.93 (0.85-1.02)		
rs999737	Mean lifetime intake of alcohol (g/day)	>=10-<20	537/ 590	0.73 (0.60-0.89)		
rs999737	Mean lifetime intake of alcohol (g/day)	>=20	421/ 394	1.21 (0.96-1.53)		
rs999737	Mean lifetime intake of alcohol (10g/day)	combined	5644/ 6381		1.02	5.3E-01
rs999737	Smoking (ever)	no	8494/ 8263	0.93 (0.88-0.98)		
rs999737	Smoking (ever)	yes	7183/ 6774	0.95 (0.90-1.01)		
rs999737	Smoking (ever/never)	combined	15677/15037		1.02	5.7E-01
rs999737	Smoking (pack-years)	0	7724/ 7263	0.94 (0.89-1.00)		
rs999737	Smoking (pack-years)	0-<10	2769/ 2881	0.94 (0.86-1.03)		
rs999737	Smoking (pack-years)	10-<20	1339/ 1292	0.97 (0.85-1.11)		
rs999737	Smoking (pack-years)	>=20	1918/ 1629	0.91 (0.82-1.02)		
rs999737	Smoking (pack-years/10)	combined	13750/13065		1.01	6.8E-01
rs999737	Physical activity during year before reference date (h/week)	0	1080/ 1004	1.00 (0.87-1.16)		
rs999737	Physical activity during year before reference date (h/week)	0-<3.5	2061/ 2238	0.99 (0.90-1.09)		
rs999737	Physical activity during year before reference date (h/week)	3.5-<7	1308/ 1392	0.89 (0.78-1.01)		
rs999737	Physical activity during year before reference date (h/week)	>=7	1298/ 1342	0.99 (0.87-1.13)		
rs999737	Physical activity during year before reference date (square root of h/week)	combined	5747/ 5976		0.98	3.2E-01

[1] Per-allele SNP effect in each stratum of environmental risk factors using case-control analysis adjusted for study and reference age
[2] OR for GxE interaction from case-control analysis stratified by study and adjusted for reference age
[3] p value for case-control analysis stratified by study and adjusted for reference age
[4] model used never use of MHT (menopausal hormone therapy) as the reference category and adjusted for former use of MHT and current use of other MHT type, as appropriate
[5] mean lifetime alcohol intake derived from duration and amount of alcohol intake in g/day at different age periods
[6] or the highly correlated SNP rs1975930 (r^2= 1 in HapMap CEU)
[7] or the highly correlated SNP rs1045485 (r^2= 1 in HapMap CEU)
[8] or the highly correlated SNP rs10483813 (r^2= 1 in HapMap CEU)
