# Supplementary material for: Evidence of Gene–Environment Interactions between Common Breast Cancer Susceptibility Loci and Established Environmental Risk Factors
Source: PLoS Genet. 2013 Mar 27;9(3):e1003284. doi: 10.1371/journal.pgen.1003284 (PMC3609648; doi:10.1371/journal.pgen.1003284)
Supplement: Table S5 — Per-allele odds ratios (OR) and 95% confidence intervals (CI) for SNPs by environmental risk factors of breast cancer, estrogen receptor positive. (PDF) [file pgen.1003284.s005.pdf]

Table S5. Per-allele odds ratios (OR) and 95% confidence intervals (CI) for SNPs by environmental risk factors of breast cancer, estrogen receptor positive

SNP	Variable	Stratum	N (cases/ controls)	OR (95% CI)[1]	OR int[2]	P int[3]
rs1011970	Age at menarche (years)	<=11	1357/ 2920	1.04 (0.92-1.18)		
rs1011970	Age at menarche (years)	12-13	5021/10797	1.09 (1.01-1.16)		
rs1011970	Age at menarche (years)	>=14	4707/10850	1.06 (0.99-1.13)		
rs1011970	Age at menarche (years/2)	combined	11085/24567		1.02	5.6E-01
rs1011970	Parous	no	2190/ 3808	1.02 (0.92-1.14)		
rs1011970	Parous	yes	11766/24161	1.07 (1.02-1.12)		
rs1011970	Parous (yes/no)	combined	13956/27969		1.04	4.6E-01
rs1011970	Number of births (among parous)	1	2885/ 5136	1.10 (1.00-1.21)		
rs1011970	Number of births (among parous)	2	5084/11012	1.02 (0.95-1.09)		
rs1011970	Number of births (among parous)	3	2347/ 4819	1.15 (1.04-1.27)		
rs1011970	Number of births (among parous)	>=4	1247/ 2321	1.07 (0.94-1.23)		
rs1011970	Number of births (among parous)	combined	11563/23288		1.00	9.1E-01
rs1011970	Age at first birth (among parous, years)	<20	1043/ 2480	1.03 (0.89-1.19)		
rs1011970	Age at first birth (among parous, years)	20-24	3982/ 9285	1.06 (0.98-1.14)		
rs1011970	Age at first birth (among parous, years)	25-29	3061/ 6903	1.06 (0.98-1.16)		
rs1011970	Age at first birth (among parous, years)	>=30	1567/ 2996	1.08 (0.95-1.22)		
rs1011970	Age at first birth (among parous, years/5)	combined	9653/21664		1.00	8.7E-01
rs1011970	Ever breastfed (among parous, yes/no)	no	1407/ 2303	1.11 (0.98-1.27)		
rs1011970	Ever breastfed (among parous, yes/no)	yes	4608/ 9425	1.07 (1.00-1.15)		
rs1011970	Ever breastfed (among parous, yes/no)	combined	6015/11728		0.96	6.1E-01
rs1011970	Usual adult BMI, age<54	<25	1672/ 2725	1.05 (0.93-1.18)		
rs1011970	Usual adult BMI, age<54	25-<30	535/ 880	1.15 (0.94-1.41)		
rs1011970	Usual adult BMI, age<54	>=30	236/ 417	1.39 (1.03-1.87)		
rs1011970	Usual adult BMI (BMI/5), age<54	combined	2443/ 4022		1.09	1.2E-01
rs1011970	Usual adult BMI, age>=54	<25	2900/ 5944	1.13 (1.03-1.23)		
rs1011970	Usual adult BMI, age>=54	25-<30	1261/ 2490	0.96 (0.84-1.10)		
rs1011970	Usual adult BMI, age>=54	>=30	479/ 811	0.89 (0.72-1.11)		
rs1011970	Usual adult BMI (BMI/5), age>=54	combined	4640/ 9245		0.93	1.0E-01
rs1011970	Usual adult height (cm)	<160	2465/ 4896	0.98 (0.89-1.08)		
rs1011970	Usual adult height (cm)	160-<165	3120/ 5901	1.18 (1.09-1.29)		
rs1011970	Usual adult height (cm)	165-<170	2694/ 4857	1.04 (0.95-1.14)		
rs1011970	Usual adult height (cm)	>=170	1969/ 3206	1.04 (0.93-1.16)		
rs1011970	Usual adult height (cm/5)	combined	10248/18860		1.00	9.4E-01
rs1011970	Ever use of oral contraceptives	no	4091/ 7376	1.06 (0.99-1.15)		
rs1011970	Ever use of oral contraceptives	yes	4748/10197	1.07 (1.00-1.14)		
rs1011970	Ever use of oral contraceptives (yes/no)	combined	8839/17573		1.00	9.3E-01
rs1011970	Duration of oral contraceptive use (years)	0	4091/ 7376	1.06 (0.99-1.15)		
rs1011970	Duration of oral contraceptive use (years)	>0-<5	1573/ 3384	1.08 (0.96-1.21)		
rs1011970	Duration of oral contraceptive use (years)	5-<10	1135/ 2484	1.06 (0.93-1.22)		
rs1011970	Duration of oral contraceptive use (years)	>=10	1935/ 4047	1.05 (0.95-1.17)		
rs1011970	Duration of oral contraceptive use (years/5)	combined	8734/17291		1.00	8.6E-01
rs1011970	Current use of combined estrogen/ progestagen MHT	never	1777/ 4067	1.17 (1.05-1.30)		
rs1011970	Current use of combined estrogen/ progestagen MHT	EPCurrent	831/ 1186	1.01 (0.85-1.20)		
rs1011970	Current use of combined estrogen/ progestagen MHT (yes/no)	combined	3833/ 8108		0.91	3.2E-01
rs1011970	Current use of estrogen only MHT	never	1878/ 4138	1.15 (1.04-1.28)		
rs1011970	Current use of estrogen only MHT	ECurrent	427/ 916	1.04 (0.84-1.30)		
rs1011970	Current use of estrogen only MHT (yes/no)	combined	4013/ 8272		0.96	7.5E-01

SNP	Variable	Stratum	N (cases/ controls)	OR (95% CI)[1]	OR int[2]	P int[3]
rs1011970	Duration of combined estrogen/progestagen MHT among current users (years)	never	1777/ 4067	1.17 (1.05-1.30)		
rs1011970	Duration of combined estrogen/progestagen MHT among current users (years)	>0-<5	166/ 314	1.29 (0.91-1.82)		
rs1011970	Duration of combined estrogen/progestagen MHT among current users (years)	5-<10	264/ 352	1.25 (0.92-1.70)		
rs1011970	Duration of combined estrogen/progestagen MHT among current users (years)	>=10	345/ 462	0.77 (0.58-1.01)		
rs1011970	Duration of combined estrogen/progestagen MHT among current users (years/5)	combined	3756/ 8013		0.91	3.5E-02
rs1011970	Duration of estrogen only MHT among current users (years)	never	1878/ 4138	1.15 (1.04-1.28)		
rs1011970	Duration of estrogen only MHT among current users (years)	>0-<5	95/ 279	1.30 (0.85-1.99)		
rs1011970	Duration of estrogen only MHT among current users (years)	5-<10	126/ 254	1.09 (0.70-1.72)		
rs1011970	Duration of estrogen only MHT among current users (years)	>=10	191/ 360	0.93 (0.67-1.29)		
rs1011970	Duration of estrogen only MHT among current users (years/5)	combined	3972/ 8197		0.95	3.3E-01
rs1011970	Mean lifetime intake of alcohol (g/day)	0	1343/ 2803	1.20 (1.06-1.36)		
rs1011970	Mean lifetime intake of alcohol (g/day)	>0-<10	2062/ 4654	1.14 (1.03-1.26)		
rs1011970	Mean lifetime intake of alcohol (g/day)	>=10-<20	419/ 1059	1.09 (0.87-1.36)		
rs1011970	Mean lifetime intake of alcohol (g/day)	>=20	341/ 741	1.01 (0.79-1.30)		
rs1011970	Mean lifetime intake of alcohol (10g/day)	combined	4165/ 9257		0.98	4.5E-01
rs1011970	Smoking (ever)	no	5548/ 9654	1.10 (1.03-1.18)		
rs1011970	Smoking (ever)	yes	4144/ 7798	1.03 (0.96-1.11)		
rs1011970	Smoking (ever/never)	combined	9692/17452		0.94	1.9E-01
rs1011970	Smoking (pack-years)	0	4963/ 8476	1.11 (1.04-1.19)		
rs1011970	Smoking (pack-years)	0-<10	1529/ 3379	1.08 (0.96-1.22)		
rs1011970	Smoking (pack-years)	10-<20	725/ 1477	1.02 (0.86-1.20)		
rs1011970	Smoking (pack-years)	>=20	1014/ 1849	1.01 (0.87-1.18)		
rs1011970	Smoking (pack-years/10)	combined	8231/15181		0.96	9.1E-02
rs1011970	Physical activity during year before reference date (h/week)	0	515/ 680	1.20 (0.97-1.50)		
rs1011970	Physical activity during year before reference date (h/week)	0-<3.5	1335/ 2413	1.14 (1.00-1.29)		
rs1011970	Physical activity during year before reference date (h/week)	3.5-<7	952/ 1962	0.92 (0.80-1.07)		
rs1011970	Physical activity during year before reference date (h/week)	>=7	1266/ 3343	1.11 (0.98-1.26)		
rs1011970	Physical activity during year before reference date (square root of h/week)	combined	4068/ 8398		0.98	4.6E-01
rs10771399[6]	Age at menarche (years)	<=11	1734/ 2111	0.76 (0.66-0.89)		
rs10771399	Age at menarche (years)	12-13	5048/ 5902	0.89 (0.81-0.97)		
rs10771399	Age at menarche (years)	>=14	4294/ 5310	0.87 (0.79-0.95)		
rs10771399	Age at menarche (years/2)	combined	11076/13323		1.02	6.1E-01
rs10771399	Parous	no	2056/ 2187	0.77 (0.67-0.89)		
rs10771399	Parous	yes	11764/13944	0.88 (0.83-0.93)		
rs10771399	Parous (yes/no)	combined	13820/16131		1.14	1.0E-01
rs10771399	Number of births (among parous)	1	2437/ 2515	0.92 (0.81-1.05)		
rs10771399	Number of births (among parous)	2	5320/ 5872	0.85 (0.78-0.93)		
rs10771399	Number of births (among parous)	3	2559/ 3033	0.93 (0.82-1.05)		
rs10771399	Number of births (among parous)	>=4	1290/ 1720	0.80 (0.68-0.95)		
rs10771399	Number of births (among parous)	combined	11606/13140		1.00	9.9E-01
rs10771399	Age at first birth (among parous, years)	<20	1201/ 1162	0.81 (0.66-0.99)		
rs10771399	Age at first birth (among parous, years)	20-24	4080/ 5115	0.92 (0.83-1.01)		
rs10771399	Age at first birth (among parous, years)	25-29	2944/ 3796	0.86 (0.76-0.96)		
rs10771399	Age at first birth (among parous, years)	>=30	1475/ 1567	0.86 (0.73-1.03)		
rs10771399	Age at first birth (among parous, years/5)	combined	9700/11640		1.01	8.2E-01
rs10771399	Ever breastfed (among parous, yes/no)	no	879/ 1045	0.85 (0.70-1.05)		

SNP	Variable	Stratum	N (cases/ controls)	OR (95% CI)[1]	OR int[2]	P int[3]
rs10771399	Ever breastfed (among parous, yes/no)	yes	3150/ 4980	0.88 (0.79-0.97)		
rs10771399	Ever breastfed (among parous, yes/no)	combined	4029/ 6025		1.03	8.2E-01
rs10771399	Usual adult BMI, age<54	<25	1087/ 1232	0.80 (0.66-0.96)		
rs10771399	Usual adult BMI, age<54	25-<30	362/ 468	1.02 (0.73-1.41)		
rs10771399	Usual adult BMI, age<54	>=30	159/ 275	0.56 (0.34-0.91)		
rs10771399	Usual adult BMI (BMI/5), age<54	combined	1608/ 1975		0.94	4.8E-01
rs10771399	Usual adult BMI, age>=54	<25	1943/ 2851	0.86 (0.75-0.98)		
rs10771399	Usual adult BMI, age>=54	25-<30	903/ 1409	0.93 (0.77-1.13)		
rs10771399	Usual adult BMI, age>=54	>=30	358/ 558	0.71 (0.51-1.00)		
rs10771399	Usual adult BMI (BMI/5), age>=54	combined	3204/ 4818		1.02	7.7E-01
rs10771399	Usual adult height (cm)	<160	1690/ 2834	0.88 (0.76-1.01)		
rs10771399	Usual adult height (cm)	160-<165	2099/ 3310	0.87 (0.76-0.99)		
rs10771399	Usual adult height (cm)	165-<170	1830/ 2748	0.87 (0.75-1.00)		
rs10771399	Usual adult height (cm)	>=170	1324/ 1816	0.88 (0.75-1.03)		
rs10771399	Usual adult height (cm/5)	combined	6943/10708		1.00	9.2E-01
rs10771399	Ever use of oral contraceptives	no	2905/ 3904	0.90 (0.80-1.00)		
rs10771399	Ever use of oral contraceptives	yes	3434/ 6211	0.89 (0.80-0.97)		
rs10771399	Ever use of oral contraceptives (yes/no)	combined	6339/10115		0.99	8.5E-01
rs10771399	Duration of oral contraceptive use (years)	0	2905/ 3904	0.90 (0.80-1.00)		
rs10771399	Duration of oral contraceptive use (years)	>0-<5	1140/ 2097	0.91 (0.77-1.07)		
rs10771399	Duration of oral contraceptive use (years)	5-<10	831/ 1531	0.92 (0.76-1.11)		
rs10771399	Duration of oral contraceptive use (years)	>=10	1371/ 2384	0.86 (0.73-1.00)		
rs10771399	Duration of oral contraceptive use (years/5)	combined	6247/ 9916		0.97	3.3E-01
rs10771399	Current use of combined estrogen/ progestagen MHT	never	1248/ 1972	0.80 (0.69-0.94)		
rs10771399	Current use of combined estrogen/ progestagen MHT	EPCurrent	562/ 603	0.98 (0.76-1.27)		
rs10771399	Current use of combined estrogen/ progestagen MHT (yes/no)	combined	2693/ 3929		1.15	3.4E-01
rs10771399	Current use of estrogen only MHT	never	1341/ 2010	0.81 (0.70-0.95)		
rs10771399	Current use of estrogen only MHT	ECurrent	342/ 488	1.11 (0.82-1.50)		
rs10771399	Current use of estrogen only MHT (yes/no)	combined	2860/ 4031		1.30	1.1E-01
rs10771399	Duration of combined estrogen/progestagen MHT among current users (years)	never	1248/ 1972	0.80 (0.69-0.94)		
rs10771399	Duration of combined estrogen/progestagen MHT among current users (years)	>0-<5	113/ 168	1.35 (0.78-2.35)		
rs10771399	Duration of combined estrogen/progestagen MHT among current users (years)	5-<10	171/ 176	0.75 (0.49-1.17)		
rs10771399	Duration of combined estrogen/progestagen MHT among current users (years)	>=10	226/ 209	0.98 (0.65-1.49)		
rs10771399	Duration of combined estrogen/progestagen MHT among current users (years/5)	combined	2622/ 3851		1.03	7.0E-01
rs10771399	Duration of estrogen only MHT among current users (years)	never	1341/ 2010	0.81 (0.70-0.95)		
rs10771399	Duration of estrogen only MHT among current users (years)	>0-<5	76/ 150	0.91 (0.49-1.67)		
rs10771399	Duration of estrogen only MHT among current users (years)	5-<10	112/ 141	0.92 (0.54-1.54)		
rs10771399	Duration of estrogen only MHT among current users (years)	>=10	139/ 181	1.70 (1.01-2.84)		
rs10771399	Duration of estrogen only MHT among current users (years/5)	combined	2822/ 3974		1.18	3.1E-02
rs10771399	Mean lifetime intake of alcohol (g/day)	0	800/ 1072	0.85 (0.69-1.06)		
rs10771399	Mean lifetime intake of alcohol (g/day)	>0-<10	1395/ 1926	0.94 (0.81-1.10)		
rs10771399	Mean lifetime intake of alcohol (g/day)	>=10-<20	278/ 417	0.76 (0.53-1.10)		
rs10771399	Mean lifetime intake of alcohol (g/day)	>=20	229/ 313	0.79 (0.55-1.13)		
rs10771399	Mean lifetime intake of alcohol (10g/day)	combined	2702/ 3728		0.98	5.4E-01
rs10771399	Smoking (ever)	no	3760/ 5606	0.90 (0.82-0.99)		
rs10771399	Smoking (ever)	yes	2874/ 4071	0.83 (0.75-0.93)		
rs10771399	Smoking (ever/never)	combined	6634/ 9677		0.93	3.1E-01

SNP	Variable	Stratum	N (cases/ controls)	OR (95% CI)[1]	OR int[2]	P int[3]
rs10771399	Smoking (pack-years)	0	3186/ 4483	0.90 (0.81-1.00)		
rs10771399	Smoking (pack-years)	0-<10	1019/ 1589	0.88 (0.73-1.06)		
rs10771399	Smoking (pack-years)	10-<20	483/ 689	0.86 (0.66-1.14)		
rs10771399	Smoking (pack-years)	>=20	672/ 891	0.81 (0.64-1.03)		
rs10771399	Smoking (pack-years/10)	combined	5360/ 7652		0.99	7.5E-01
rs10771399	Physical activity during year before reference date (h/week)	0	481/ 595	0.87 (0.65-1.15)		
rs10771399	Physical activity during year before reference date (h/week)	0-<3.5	977/ 1607	0.89 (0.75-1.06)		
rs10771399	Physical activity during year before reference date (h/week)	3.5-<7	661/ 1113	0.82 (0.66-1.01)		
rs10771399	Physical activity during year before reference date (h/week)	>=7	932/ 1290	0.96 (0.79-1.15)		
rs10771399	Physical activity during year before reference date (square root of h/week)	combined	3051/ 4605		1.03	4.2E-01
rs10941679	Age at menarche (years)	<=11	2221/ 3760	1.17 (1.07-1.28)		
rs10941679	Age at menarche (years)	12-13	6715/12457	1.18 (1.12-1.23)		
rs10941679	Age at menarche (years)	>=14	5895/12787	1.15 (1.09-1.21)		
rs10941679	Age at menarche (years/2)	combined	14831/29004		0.98	3.6E-01
rs10941679	Parous	no	2642/ 4299	1.11 (1.02-1.20)		
rs10941679	Parous	yes	14592/28290	1.15 (1.12-1.19)		
rs10941679	Parous (yes/no)	combined	17234/32589		1.04	3.8E-01
rs10941679	Number of births (among parous)	1	3255/ 5763	1.16 (1.08-1.25)		
rs10941679	Number of births (among parous)	2	6595/13017	1.17 (1.12-1.23)		
rs10941679	Number of births (among parous)	3	3053/ 5783	1.15 (1.06-1.23)		
rs10941679	Number of births (among parous)	>=4	1508/ 2883	1.09 (0.98-1.21)		
rs10941679	Number of births (among parous)	combined	14411/27446		0.99	3.9E-01
rs10941679	Age at first birth (among parous, years)	<20	1534/ 2834	1.11 (1.00-1.23)		
rs10941679	Age at first birth (among parous, years)	20-24	5371/11195	1.17 (1.11-1.24)		
rs10941679	Age at first birth (among parous, years)	25-29	3977/ 8275	1.21 (1.14-1.29)		
rs10941679	Age at first birth (among parous, years)	>=30	2015/ 3551	1.09 (0.99-1.19)		
rs10941679	Age at first birth (among parous, years/5)	combined	12897/25855		1.00	9.6E-01
rs10941679	Ever breastfed (among parous, yes/no)	no	1480/ 2886	1.12 (1.00-1.24)		
rs10941679	Ever breastfed (among parous, yes/no)	yes	4958/10347	1.14 (1.08-1.20)		
rs10941679	Ever breastfed (among parous, yes/no)	combined	6438/13233		1.02	7.5E-01
rs10941679	Usual adult BMI, age<54	<25	1776/ 3124	1.10 (1.00-1.21)		
rs10941679	Usual adult BMI, age<54	25-<30	560/ 1055	1.02 (0.86-1.22)		
rs10941679	Usual adult BMI, age<54	>=30	249/ 521	1.23 (0.96-1.59)		
rs10941679	Usual adult BMI (BMI/5), age<54	combined	2585/ 4700		1.04	4.1E-01
rs10941679	Usual adult BMI, age>=54	<25	3073/ 6461	1.10 (1.02-1.18)		
rs10941679	Usual adult BMI, age>=54	25-<30	1296/ 2735	1.26 (1.13-1.41)		
rs10941679	Usual adult BMI, age>=54	>=30	494/ 942	1.14 (0.95-1.38)		
rs10941679	Usual adult BMI (BMI/5), age>=54	combined	4863/10138		1.06	8.1E-02
rs10941679	Usual adult height (cm)	<160	2583/ 5328	1.14 (1.05-1.23)		
rs10941679	Usual adult height (cm)	160-<165	3243/ 6388	1.12 (1.05-1.20)		
rs10941679	Usual adult height (cm)	165-<170	2747/ 5263	1.12 (1.04-1.21)		
rs10941679	Usual adult height (cm)	>=170	2038/ 3480	1.11 (1.01-1.21)		
rs10941679	Usual adult height (cm/5)	combined	10611/20459		0.99	3.7E-01
rs10941679	Ever use of oral contraceptives	no	4346/ 8189	1.12 (1.06-1.19)		
rs10941679	Ever use of oral contraceptives	yes	5001/10716	1.11 (1.05-1.18)		
rs10941679	Ever use of oral contraceptives (yes/no)	combined	9347/18905		0.99	8.0E-01
rs10941679	Duration of oral contraceptive use (years)	0	4346/ 8189	1.12 (1.06-1.19)		
rs10941679	Duration of oral contraceptive use (years)	>0-<5	1683/ 3540	1.12 (1.02-1.24)		
rs10941679	Duration of oral contraceptive use (years)	5-<10	1184/ 2644	0.99 (0.88-1.11)		

SNP	Variable	Stratum	N (cases/ controls)	OR (95% CI)[1]	OR int[2]	P int[3]
rs10941679	Duration of oral contraceptive use (years)	>=10	2019/ 4247	1.16 (1.06-1.27)		
rs10941679	Duration of oral contraceptive use (years/5)	combined	9232/18620		1.01	4.4E-01
rs10941679	Current use of combined estrogen/ progestagen MHT	never	1879/ 4486	1.15 (1.06-1.26)		
rs10941679	Current use of combined estrogen/ progestagen MHT	EPCurrent	882/ 1365	1.10 (0.95-1.27)		
rs10941679	Current use of combined estrogen/ progestagen MHT (yes/no)	combined	4039/ 8973		0.96	5.7E-01
rs10941679	Current use of estrogen only MHT	never	1979/ 4568	1.15 (1.06-1.25)		
rs10941679	Current use of estrogen only MHT	ECurrent	434/ 1055	1.14 (0.95-1.38)		
rs10941679	Current use of estrogen only MHT (yes/no)	combined	4218/ 9164		1.00	9.8E-01
rs10941679	Duration of combined estrogen/progestagen MHT among current users (years)	never	1879/ 4486	1.16 (1.06-1.26)		
rs10941679	Duration of combined estrogen/progestagen MHT among current users (years)	>0-<5	175/ 373	1.30 (0.97-1.74)		
rs10941679	Duration of combined estrogen/progestagen MHT among current users (years)	5-<10	283/ 411	1.09 (0.85-1.41)		
rs10941679	Duration of combined estrogen/progestagen MHT among current users (years)	>=10	368/ 523	0.99 (0.79-1.24)		
rs10941679	Duration of combined estrogen/progestagen MHT among current users (years/5)	combined	3962/ 8878		0.95	1.7E-01
rs10941679	Duration of estrogen only MHT among current users (years)	never	1979/ 4568	1.15 (1.06-1.25)		
rs10941679	Duration of estrogen only MHT among current users (years)	>0-<5	95/ 298	1.09 (0.75-1.58)		
rs10941679	Duration of estrogen only MHT among current users (years)	5-<10	131/ 286	1.12 (0.79-1.58)		
rs10941679	Duration of estrogen only MHT among current users (years)	>=10	193/ 432	1.17 (0.87-1.57)		
rs10941679	Duration of estrogen only MHT among current users (years/5)	combined	4177/ 9047		1.00	1.0E+00
rs10941679	Mean lifetime intake of alcohol (g/day)	0	1499/ 3017	1.16 (1.05-1.28)		
rs10941679	Mean lifetime intake of alcohol (g/day)	>0-<10	2232/ 4933	1.11 (1.02-1.20)		
rs10941679	Mean lifetime intake of alcohol (g/day)	>=10-<20	467/ 1128	1.13 (0.95-1.35)		
rs10941679	Mean lifetime intake of alcohol (g/day)	>=20	377/ 801	1.15 (0.95-1.40)		
rs10941679	Mean lifetime intake of alcohol (10g/day)	combined	4575/ 9879		1.01	8.0E-01
rs10941679	Smoking (ever)	no	5628/10276	1.13 (1.07-1.20)		
rs10941679	Smoking (ever)	yes	4319/ 8592	1.12 (1.05-1.19)		
rs10941679	Smoking (ever/never)	combined	9947/18868		0.99	7.5E-01
rs10941679	Smoking (pack-years)	0	5039/ 9154	1.11 (1.05-1.18)		
rs10941679	Smoking (pack-years)	0-<10	1662/ 3682	1.18 (1.07-1.30)		
rs10941679	Smoking (pack-years)	10-<20	773/ 1652	1.01 (0.87-1.17)		
rs10941679	Smoking (pack-years)	>=20	1071/ 2162	1.14 (1.01-1.30)		
rs10941679	Smoking (pack-years/10)	combined	8545/16650		1.00	8.8E-01
rs10941679	Physical activity during year before reference date (h/week)	0	517/ 1068	1.37 (1.13-1.67)		
rs10941679	Physical activity during year before reference date (h/week)	0-<3.5	1341/ 2758	1.12 (1.00-1.24)		
rs10941679	Physical activity during year before reference date (h/week)	3.5-<7	970/ 2183	1.11 (0.98-1.25)		
rs10941679	Physical activity during year before reference date (h/week)	>=7	1359/ 3684	1.09 (0.98-1.20)		
rs10941679	Physical activity during year before reference date (square root of h/week)	combined	4187/ 9693		0.98	3.2E-01
rs10995190	Age at menarche (years)	<=11	1357/ 2927	0.92 (0.80-1.05)		
rs10995190	Age at menarche (years)	12-13	5020/10817	0.86 (0.80-0.92)		
rs10995190	Age at menarche (years)	>=14	4706/10884	0.90 (0.84-0.97)		
rs10995190	Age at menarche (years/2)	combined	11083/24628		1.00	9.6E-01
rs10995190	Parous	no	2119/ 3814	0.91 (0.82-1.02)		
rs10995190	Parous	yes	11317/24215	0.88 (0.84-0.92)		
rs10995190	Parous (yes/no)	combined	13436/28029		0.96	5.5E-01
rs10995190	Number of births (among parous)	1	2747/ 5150	0.89 (0.81-0.98)		
rs10995190	Number of births (among parous)	2	4905/11034	0.83 (0.78-0.90)		
rs10995190	Number of births (among parous)	3	2268/ 4831	0.93 (0.84-1.03)		

SNP	Variable	Stratum	N (cases/ controls)	OR (95% CI)[1]	OR int[2]	P int[3]
rs10995190	Number of births (among parous)	>=4	1211/2327	0.96 (0.83-1.11)		
rs10995190	Number of births (among parous)	combined	11131/23342		1.02	3.2E-01
rs10995190	Age at first birth (among parous, years)	<20	1043/2483	0.89 (0.77-1.03)		
rs10995190	Age at first birth (among parous, years)	20-24	3984/9312	0.93 (0.87-1.01)		
rs10995190	Age at first birth (among parous, years)	25-29	3059/6920	0.87 (0.80-0.95)		
rs10995190	Age at first birth (among parous, years)	>=30	1566/3005	0.80 (0.70-0.91)		
rs10995190	Age at first birth (among parous, years/5)	combined	9652/21720		0.95	7.4E-02
rs10995190	Ever breastfed (among parous, yes/no)	no	1405/2302	0.84 (0.74-0.96)		
rs10995190	Ever breastfed (among parous, yes/no)	yes	4606/9423	0.90 (0.84-0.96)		
rs10995190	Ever breastfed (among parous, yes/no)	combined	6011/11725		1.07	4.0E-01
rs10995190	Usual adult BMI, age<54	<25	1672/2728	0.91 (0.81-1.03)		
rs10995190	Usual adult BMI, age<54	25-<30	534/ 881	0.90 (0.73-1.12)		
rs10995190	Usual adult BMI, age<54	>=30	236/ 417	0.96 (0.71-1.29)		
rs10995190	Usual adult BMI (BMI/5), age<54	combined	2442/4026		0.96	4.8E-01
rs10995190	Usual adult BMI, age>=54	<25	2900/5945	0.89 (0.82-0.98)		
rs10995190	Usual adult BMI, age>=54	25-<30	1258/2487	0.83 (0.72-0.95)		
rs10995190	Usual adult BMI, age>=54	>=30	478/ 811	0.79 (0.62-1.01)		
rs10995190	Usual adult BMI (BMI/5), age>=54	combined	4636/9243		0.99	7.8E-01
rs10995190	Usual adult height (cm)	<160	2462/4897	0.80 (0.72-0.88)		
rs10995190	Usual adult height (cm)	160-<165	3119/5900	0.87 (0.79-0.95)		
rs10995190	Usual adult height (cm)	165-<170	2691/4855	0.88 (0.80-0.96)		
rs10995190	Usual adult height (cm)	>=170	1969/3207	0.95 (0.85-1.07)		
rs10995190	Usual adult height (cm/5)	combined	10241/18859		1.04	3.3E-02
rs10995190	Ever use of oral contraceptives	no	4086/7379	0.82 (0.76-0.88)		
rs10995190	Ever use of oral contraceptives	yes	4749/10198	0.92 (0.86-0.99)		
rs10995190	Ever use of oral contraceptives (yes/no)	combined	8835/17577		1.13	2.5E-02
rs10995190	Duration of oral contraceptive use (years)	0	4086/7379	0.82 (0.76-0.88)		
rs10995190	Duration of oral contraceptive use (years)	>0-<5	1576/3383	0.96 (0.85-1.08)		
rs10995190	Duration of oral contraceptive use (years)	5-<10	1132/2482	0.89 (0.77-1.03)		
rs10995190	Duration of oral contraceptive use (years)	>=10	1937/4051	0.90 (0.81-1.01)		
rs10995190	Duration of oral contraceptive use (years/5)	combined	8731/17295		1.02	2.4E-01
rs10995190	Current use of combined estrogen/ progestagen MHT	never	1775/4070	0.81 (0.72-0.91)		
rs10995190	Current use of combined estrogen/ progestagen MHT	EPCurrent	833/1189	0.76 (0.64-0.91)		
rs10995190	Current use of combined estrogen/ progestagen MHT (yes/no)	combined	3832/8113		0.89	2.6E-01
rs10995190	Current use of estrogen only MHT	never	1875/4140	0.81 (0.73-0.91)		
rs10995190	Current use of estrogen only MHT	ECurrent	427/ 918	0.81 (0.64-1.03)		
rs10995190	Current use of estrogen only MHT (yes/no)	combined	4011/8275		0.94	6.5E-01
rs10995190	Duration of combined estrogen/progestagen MHT among current users (years)	never	1775/4070	0.81 (0.73-0.91)		
rs10995190	Duration of combined estrogen/progestagen MHT among current users (years)	>0-<5	167/ 314	0.85 (0.59-1.22)		
rs10995190	Duration of combined estrogen/progestagen MHT among current users (years)	5-<10	265/ 353	0.68 (0.49-0.94)		
rs10995190	Duration of combined estrogen/progestagen MHT among current users (years)	>=10	345/ 464	0.78 (0.59-1.04)		
rs10995190	Duration of combined estrogen/progestagen MHT among current users (years/5)	combined	3755/8018		0.95	2.8E-01
rs10995190	Duration of estrogen only MHT among current users (years)	never	1875/4140	0.81 (0.73-0.91)		
rs10995190	Duration of estrogen only MHT among current users (years)	>0-<5	95/ 280	0.83 (0.52-1.32)		
rs10995190	Duration of estrogen only MHT among current users (years)	5-<10	126/ 255	0.81 (0.53-1.25)		
rs10995190	Duration of estrogen only MHT among current users (years)	>=10	191/ 360	0.81 (0.57-1.17)		
rs10995190	Duration of estrogen only MHT among current users (years/5)	combined	3970/8200		0.97	5.5E-01

SNP	Variable	Stratum	N (cases/ controls)	OR (95% CI)[1]	OR int[2]	P int[3]
rs10995190	Mean lifetime intake of alcohol (g/day)	0	1339/ 2798	0.73 (0.64-0.83)		
rs10995190	Mean lifetime intake of alcohol (g/day)	>0-<10	2060/ 4659	0.90 (0.81-1.00)		
rs10995190	Mean lifetime intake of alcohol (g/day)	>=10-<20	418/ 1059	0.79 (0.62-1.00)		
rs10995190	Mean lifetime intake of alcohol (g/day)	>=20	341/ 740	0.96 (0.73-1.24)		
rs10995190	Mean lifetime intake of alcohol (10g/day)	combined	4158/ 9256		1.06	8.1E-02
rs10995190	Smoking (ever)	no	5547/ 9654	0.88 (0.82-0.94)		
rs10995190	Smoking (ever)	yes	4136/ 7795	0.86 (0.79-0.92)		
rs10995190	Smoking (ever/never)	combined	9683/17449		0.97	5.8E-01
rs10995190	Smoking (pack-years)	0	4962/ 8475	0.89 (0.83-0.95)		
rs10995190	Smoking (pack-years)	0-<10	1524/ 3374	0.84 (0.75-0.95)		
rs10995190	Smoking (pack-years)	10-<20	724/ 1472	0.97 (0.81-1.15)		
rs10995190	Smoking (pack-years)	>=20	1013/ 1854	0.91 (0.78-1.07)		
rs10995190	Smoking (pack-years/10)	combined	8223/15175		1.01	5.6E-01
rs10995190	Physical activity during year before reference date (h/week)	0	515/ 679	0.97 (0.77-1.23)		
rs10995190	Physical activity during year before reference date (h/week)	0-<3.5	1335/ 2417	0.88 (0.77-1.00)		
rs10995190	Physical activity during year before reference date (h/week)	3.5-<7	952/ 1969	0.90 (0.77-1.05)		
rs10995190	Physical activity during year before reference date (h/week)	>=7	1266/ 3342	0.85 (0.74-0.97)		
rs10995190	Physical activity during year before reference date (square root of h/week)	combined	4068/ 8407		0.97	3.9E-01
rs11249433	Age at menarche (years)	<=11	2153/ 3510	1.12 (1.03-1.21)		
rs11249433	Age at menarche (years)	12-13	6464/11480	1.18 (1.13-1.23)		
rs11249433	Age at menarche (years)	>=14	5487/11182	1.08 (1.03-1.13)		
rs11249433	Age at menarche (years/2)	combined	14104/26172		0.96	5.8E-02
rs11249433	Parous	no	2543/ 3796	0.97 (0.90-1.04)		
rs11249433	Parous	yes	14443/25432	1.16 (1.13-1.20)		
rs11249433	Parous (yes/no)	combined	16986/29228		1.20	1.6E-05
rs11249433	Number of births (among parous)	1	3146/ 4880	1.16 (1.08-1.24)		
rs11249433	Number of births (among parous)	2	6414/11511	1.18 (1.13-1.23)		
rs11249433	Number of births (among parous)	3	2993/ 5187	1.15 (1.08-1.24)		
rs11249433	Number of births (among parous)	>=4	1472/ 2605	1.11 (1.01-1.23)		
rs11249433	Number of births (among parous)	combined	14025/24183		0.99	3.4E-01
rs11249433	Age at first birth (among parous, years)	<20	1439/ 2412	1.17 (1.06-1.29)		
rs11249433	Age at first birth (among parous, years)	20-24	5040/ 9784	1.19 (1.13-1.25)		
rs11249433	Age at first birth (among parous, years)	25-29	3786/ 7415	1.13 (1.07-1.20)		
rs11249433	Age at first birth (among parous, years)	>=30	1929/ 3266	1.15 (1.05-1.25)		
rs11249433	Age at first birth (among parous, years/5)	combined	12194/22877		0.99	4.5E-01
rs11249433	Ever breastfed (among parous, yes/no)	no	1326/ 2254	1.19 (1.07-1.32)		
rs11249433	Ever breastfed (among parous, yes/no)	yes	4482/ 7952	1.13 (1.07-1.20)		
rs11249433	Ever breastfed (among parous, yes/no)	combined	5808/10206		0.95	4.1E-01
rs11249433	Usual adult BMI, age<54	<25	1738/ 2863	1.12 (1.03-1.23)		
rs11249433	Usual adult BMI, age<54	25-<30	558/ 993	1.09 (0.93-1.27)		
rs11249433	Usual adult BMI, age<54	>=30	248/ 501	1.03 (0.82-1.30)		
rs11249433	Usual adult BMI (BMI/5), age<54	combined	2544/ 4357		0.98	6.3E-01
rs11249433	Usual adult BMI, age>=54	<25	2509/ 4064	1.08 (1.00-1.16)		
rs11249433	Usual adult BMI, age>=54	25-<30	1161/ 2098	1.19 (1.07-1.33)		
rs11249433	Usual adult BMI, age>=54	>=30	477/ 840	1.25 (1.04-1.50)		
rs11249433	Usual adult BMI (BMI/5), age>=54	combined	4147/ 7002		1.03	4.0E-01
rs11249433	Usual adult height (cm)	<160	2368/ 4519	1.11 (1.03-1.19)		
rs11249433	Usual adult height (cm)	160-<165	2935/ 5243	1.17 (1.09-1.25)		
rs11249433	Usual adult height (cm)	165-<170	2473/ 4167	1.10 (1.02-1.19)		

SNP	Variable	Stratum	N (cases/ controls)	OR (95% CI)[1]	OR int[2]	P int[3]
rs11249433	Usual adult height (cm)	>=170	1794/ 2739	1.04 (0.95-1.13)		
rs11249433	Usual adult height (cm/5)	combined	9570/16668		0.98	2.4E-01
rs11249433	Ever use of oral contraceptives	no	3926/ 6821	1.14 (1.08-1.21)		
rs11249433	Ever use of oral contraceptives	yes	4416/ 8286	1.08 (1.02-1.14)		
rs11249433	Ever use of oral contraceptives (yes/no)	combined	8342/15107		0.95	2.0E-01
rs11249433	Duration of oral contraceptive use (years)	0	3926/ 6821	1.14 (1.08-1.21)		
rs11249433	Duration of oral contraceptive use (years)	>0-<5	1510/ 2821	1.05 (0.96-1.16)		
rs11249433	Duration of oral contraceptive use (years)	5-<10	1056/ 2053	1.06 (0.95-1.19)		
rs11249433	Duration of oral contraceptive use (years)	>=10	1744/ 3145	1.11 (1.02-1.22)		
rs11249433	Duration of oral contraceptive use (years/5)	combined	8236/14840		1.00	8.0E-01
rs11249433	Current use of combined estrogen/ progestagen MHT	never	1627/ 3247	1.05 (0.96-1.15)		
rs11249433	Current use of combined estrogen/ progestagen MHT	EPCurrent	638/ 769	1.19 (1.02-1.39)		
rs11249433	Current use of combined estrogen/ progestagen MHT (yes/no)	combined	3305/ 5801		1.11	2.4E-01
rs11249433	Current use of estrogen only MHT	never	1730/ 3329	1.05 (0.96-1.14)		
rs11249433	Current use of estrogen only MHT	ECurrent	374/ 648	1.11 (0.92-1.34)		
rs11249433	Current use of estrogen only MHT (yes/no)	combined	3490/ 5988		1.02	8.1E-01
rs11249433	Duration of combined estrogen/progestagen MHT among current users (years)	never	1627/ 3247	1.05 (0.96-1.15)		
rs11249433	Duration of combined estrogen/progestagen MHT among current users (years)	>0-<5	134/ 245	1.30 (0.95-1.77)		
rs11249433	Duration of combined estrogen/progestagen MHT among current users (years)	5-<10	206/ 230	1.19 (0.89-1.59)		
rs11249433	Duration of combined estrogen/progestagen MHT among current users (years)	>=10	242/ 243	1.15 (0.88-1.49)		
rs11249433	Duration of combined estrogen/progestagen MHT among current users (years/5)	combined	3228/ 5717		1.03	4.6E-01
rs11249433	Duration of estrogen only MHT among current users (years)	never	1730/ 3329	1.04 (0.96-1.14)		
rs11249433	Duration of estrogen only MHT among current users (years)	>0-<5	83/ 180	1.14 (0.78-1.67)		
rs11249433	Duration of estrogen only MHT among current users (years)	5-<10	115/ 175	0.75 (0.53-1.06)		
rs11249433	Duration of estrogen only MHT among current users (years)	>=10	161/ 259	1.45 (1.07-1.96)		
rs11249433	Duration of estrogen only MHT among current users (years/5)	combined	3449/ 5875		1.05	2.7E-01
rs11249433	Mean lifetime intake of alcohol (g/day)	0	1350/ 2523	1.11 (1.01-1.22)		
rs11249433	Mean lifetime intake of alcohol (g/day)	>0-<10	1779/ 2858	1.11 (1.02-1.21)		
rs11249433	Mean lifetime intake of alcohol (g/day)	>=10-<20	374/ 590	1.23 (1.02-1.49)		
rs11249433	Mean lifetime intake of alcohol (g/day)	>=20	287/ 393	1.11 (0.89-1.39)		
rs11249433	Mean lifetime intake of alcohol (10g/day)	combined	3790/ 6364		1.01	6.3E-01
rs11249433	Smoking (ever)	no	5108/ 8382	1.12 (1.06-1.18)		
rs11249433	Smoking (ever)	yes	3887/ 6896	1.09 (1.03-1.16)		
rs11249433	Smoking (ever/never)	combined	8995/15278		0.98	6.2E-01
rs11249433	Smoking (pack-years)	0	4518/ 7256	1.11 (1.05-1.18)		
rs11249433	Smoking (pack-years)	0-<10	1465/ 2884	1.09 (0.99-1.20)		
rs11249433	Smoking (pack-years)	10-<20	685/ 1300	1.11 (0.96-1.28)		
rs11249433	Smoking (pack-years)	>=20	954/ 1633	1.06 (0.94-1.20)		
rs11249433	Smoking (pack-years/10)	combined	7622/13073		0.99	7.6E-01
rs11249433	Physical activity during year before reference date (h/week)	0	502/ 1004	1.05 (0.88-1.24)		
rs11249433	Physical activity during year before reference date (h/week)	0-<3.5	1247/ 2387	1.12 (1.01-1.24)		
rs11249433	Physical activity during year before reference date (h/week)	3.5-<7	808/ 1463	1.05 (0.93-1.20)		
rs11249433	Physical activity during year before reference date (h/week)	>=7	890/ 1341	1.10 (0.97-1.25)		
rs11249433	Physical activity during year before reference date (square root of h/week)	combined	3447/ 6195		1.01	6.0E-01
rs12662670	Age at menarche (years)	<=11	1358/ 1719	1.06 (0.88-1.28)		
rs12662670	Age at menarche (years)	12-13	3670/ 4831	1.10 (0.98-1.24)		

SNP	Variable	Stratum	N (cases/ controls)	OR (95% CI)[1]	OR int[2]	P int[3]
rs12662670	Age at menarche (years)	>=14	2890/ 4336	1.10 (0.97-1.25)		
rs12662670	Age at menarche (years/2)	combined	7918/10886		1.04	3.9E-01
rs12662670	Parous	no	1529/ 1880	1.14 (0.94-1.38)		
rs12662670	Parous	yes	8845/11781	1.11 (1.03-1.20)		
rs12662670	Parous (yes/no)	combined	10374/13661		0.97	7.9E-01
rs12662670	Number of births (among parous)	1	1801/ 2151	1.07 (0.91-1.26)		
rs12662670	Number of births (among parous)	2	3966/ 5068	1.21 (1.09-1.35)		
rs12662670	Number of births (among parous)	3	1931/ 2461	1.03 (0.87-1.20)		
rs12662670	Number of births (among parous)	>=4	1025/ 1296	0.99 (0.80-1.22)		
rs12662670	Number of births (among parous)	combined	8723/10976		0.95	1.0E-01
rs12662670	Age at first birth (among parous, years)	<20	912/ 948	1.04 (0.82-1.31)		
rs12662670	Age at first birth (among parous, years)	20-24	2870/ 4156	1.23 (1.09-1.40)		
rs12662670	Age at first birth (among parous, years)	25-29	2021/ 3080	0.99 (0.85-1.15)		
rs12662670	Age at first birth (among parous, years)	>=30	1011/ 1294	1.07 (0.86-1.33)		
rs12662670	Age at first birth (among parous, years/5)	combined	6814/ 9478		0.96	4.2E-01
rs12662670	Ever breastfed (among parous, yes/no)	no	516/ 598	1.16 (0.85-1.57)		
rs12662670	Ever breastfed (among parous, yes/no)	yes	2033/ 3418	1.12 (0.96-1.31)		
rs12662670	Ever breastfed (among parous, yes/no)	combined	2549/ 4016		0.97	8.6E-01
rs12662670	Usual adult BMI, age<54	<25	375/ 539	1.16 (0.83-1.63)		
rs12662670	Usual adult BMI, age<54	25-<30	183/ 252	1.44 (0.81-2.55)		
rs12662670	Usual adult BMI, age<54	>=30	86/ 156	1.36 (0.59-3.15)		
rs12662670	Usual adult BMI (BMI/5), age<54	combined	644/ 947		1.00	9.8E-01
rs12662670	Usual adult BMI, age>=54	<25	1463/ 2269	1.10 (0.91-1.33)		
rs12662670	Usual adult BMI, age>=54	25-<30	675/ 1153	1.15 (0.88-1.50)		
rs12662670	Usual adult BMI, age>=54	>=30	275/ 452	1.74 (1.12-2.69)		
rs12662670	Usual adult BMI (BMI/5), age>=54	combined	2413/ 3874		1.20	4.1E-02
rs12662670	Usual adult height (cm)	<160	1109/ 1999	1.16 (0.94-1.41)		
rs12662670	Usual adult height (cm)	160-<165	1477/ 2530	1.20 (1.01-1.44)		
rs12662670	Usual adult height (cm)	165-<170	1397/ 2244	1.23 (1.02-1.47)		
rs12662670	Usual adult height (cm)	>=170	1062/ 1525	0.85 (0.68-1.06)		
rs12662670	Usual adult height (cm/5)	combined	5045/ 8298		0.91	2.0E-02
rs12662670	Ever use of oral contraceptives	no	2030/ 2907	1.21 (1.04-1.41)		
rs12662670	Ever use of oral contraceptives	yes	2384/ 4747	1.06 (0.93-1.21)		
rs12662670	Ever use of oral contraceptives (yes/no)	combined	4414/ 7654		0.88	2.1E-01
rs12662670	Duration of oral contraceptive use (years)	0	2030/ 2907	1.21 (1.04-1.42)		
rs12662670	Duration of oral contraceptive use (years)	>0-<5	836/ 1658	1.08 (0.85-1.36)		
rs12662670	Duration of oral contraceptive use (years)	5-<10	562/ 1138	1.00 (0.76-1.30)		
rs12662670	Duration of oral contraceptive use (years)	>=10	916/ 1778	1.09 (0.88-1.36)		
rs12662670	Duration of oral contraceptive use (years/5)	combined	4344/ 7481		0.96	2.6E-01
rs12662670	Current use of combined estrogen/ progestagen MHT	never	797/ 1476	1.41 (1.12-1.77)		
rs12662670	Current use of combined estrogen/ progestagen MHT	EPCurrent	457/ 509	0.82 (0.58-1.15)		
rs12662670	Current use of combined estrogen/ progestagen MHT (yes/no)	combined	1931/ 3069		0.65	2.5E-02
rs12662670	Current use of estrogen only MHT	never	891/ 1514	1.40 (1.12-1.75)		
rs12662670	Current use of estrogen only MHT	ECurrent	310/ 455	0.94 (0.63-1.40)		
rs12662670	Current use of estrogen only MHT (yes/no)	combined	2100/ 3171		0.77	2.4E-01
rs12662670	Duration of combined estrogen/progestagen MHT among current users (years)	never	797/ 1476	1.41 (1.12-1.77)		
rs12662670	Duration of combined estrogen/progestagen MHT among current users (years)	>0-<5	94/ 153	0.68 (0.32-1.48)		
rs12662670	Duration of combined estrogen/progestagen MHT among current users (years)	5-<10	150/ 165	0.60 (0.32-1.12)		

SNP	Variable	Stratum	N (cases/ controls)	OR (95% CI)[1]	OR int[2]	P int[3]
rs12662670	Duration of combined estrogen/progestagen MHT among current users (years)	>=10	208/ 187	0.99 (0.59-1.65)		
rs12662670	Duration of combined estrogen/progestagen MHT among current users (years/5)	combined	1923/ 3064		0.88	1.5E-01
rs12662670	Duration of estrogen only MHT among current users (years)	never	891/ 1514	1.40 (1.12-1.75)		
rs12662670	Duration of estrogen only MHT among current users (years)	>0-<5	68/ 143	0.86 (0.40-1.84)		
rs12662670	Duration of estrogen only MHT among current users (years)	5-<10	107/ 138	0.94 (0.47-1.86)		
rs12662670	Duration of estrogen only MHT among current users (years)	>=10	131/ 172	1.04 (0.54-2.01)		
rs12662670	Duration of estrogen only MHT among current users (years/5)	combined	2090/ 3158		0.97	7.6E-01
rs12662670	Mean lifetime intake of alcohol (g/day)	0	165/ 268	1.39 (0.85-2.28)		
rs12662670	Mean lifetime intake of alcohol (g/day)	>0-<10	728/ 1057	1.01 (0.78-1.30)		
rs12662670	Mean lifetime intake of alcohol (g/day)	>=10-<20	163/ 236	1.83 (1.08-3.12)		
rs12662670	Mean lifetime intake of alcohol (g/day)	>=20	150/ 198	1.49 (0.84-2.64)		
rs12662670	Mean lifetime intake of alcohol (10g/day)	combined	1206/ 1759		1.00	9.8E-01
rs12662670	Smoking (ever)	no	2735/ 4219	1.16 (1.02-1.33)		
rs12662670	Smoking (ever)	yes	1965/ 2996	1.07 (0.91-1.26)		
rs12662670	Smoking (ever/never)	combined	4700/ 7215		0.92	4.3E-01
rs12662670	Smoking (pack-years)	0	2162/ 3096	1.17 (1.00-1.36)		
rs12662670	Smoking (pack-years)	0-<10	531/ 1035	1.03 (0.76-1.42)		
rs12662670	Smoking (pack-years)	10-<20	288/ 440	1.57 (1.05-2.35)		
rs12662670	Smoking (pack-years)	>=20	455/ 640	0.81 (0.58-1.13)		
rs12662670	Smoking (pack-years/10)	combined	3436/ 5211		0.97	4.4E-01
rs12662670	Physical activity during year before reference date (h/week)	0	24/ 32	1.41 (0.31-6.31)		
rs12662670	Physical activity during year before reference date (h/week)	0-<3.5	795/ 1320	1.22 (0.96-1.54)		
rs12662670	Physical activity during year before reference date (h/week)	3.5-<7	569/ 991	1.33 (1.01-1.73)		
rs12662670	Physical activity during year before reference date (h/week)	>=7	815/ 1148	1.06 (0.83-1.36)		
rs12662670	Physical activity during year before reference date (square root of h/week)	combined	2203/ 3491		0.97	5.9E-01
rs1292011	Age at menarche (years)	<=11	1547/ 1784	0.87 (0.79-0.96)		
rs1292011	Age at menarche (years)	12-13	4322/ 4745	0.95 (0.89-1.01)		
rs1292011	Age at menarche (years)	>=14	3444/ 3975	0.95 (0.88-1.02)		
rs1292011	Age at menarche (years/2)	combined	9313/10504		1.01	7.0E-01
rs1292011	Parous	no	1718/ 1737	0.91 (0.82-1.00)		
rs1292011	Parous	yes	10137/10569	0.93 (0.89-0.97)		
rs1292011	Parous (yes/no)	combined	11855/12306		1.03	6.6E-01
rs1292011	Number of births (among parous)	1	2043/ 1987	0.97 (0.88-1.07)		
rs1292011	Number of births (among parous)	2	4657/ 4688	0.94 (0.88-1.00)		
rs1292011	Number of births (among parous)	3	2240/ 2428	0.89 (0.81-0.97)		
rs1292011	Number of births (among parous)	>=4	1131/ 1437	0.92 (0.82-1.04)		
rs1292011	Number of births (among parous)	combined	10071/10540		0.99	5.9E-01
rs1292011	Age at first birth (among parous, years)	<20	1066/ 868	0.92 (0.80-1.05)		
rs1292011	Age at first birth (among parous, years)	20-24	3465/ 3984	0.92 (0.86-0.99)		
rs1292011	Age at first birth (among parous, years)	25-29	2445/ 2977	0.97 (0.90-1.06)		
rs1292011	Age at first birth (among parous, years)	>=30	1201/ 1219	0.87 (0.77-0.99)		
rs1292011	Age at first birth (among parous, years/5)	combined	8177/ 9048		1.00	9.4E-01
rs1292011	Ever breastfed (among parous, yes/no)	no	719/ 847	0.96 (0.84-1.11)		
rs1292011	Ever breastfed (among parous, yes/no)	yes	2133/ 2851	0.92 (0.85-1.00)		
rs1292011	Ever breastfed (among parous, yes/no)	combined	2852/ 3698		0.96	6.0E-01
rs1292011	Usual adult BMI, age<54	<25	889/ 884	0.94 (0.82-1.08)		
rs1292011	Usual adult BMI, age<54	25-<30	255/ 290	1.15 (0.89-1.47)		
rs1292011	Usual adult BMI, age<54	>=30	106/ 157	0.82 (0.57-1.17)		

SNP	Variable	Stratum	N (cases/ controls)	OR (95% CI)[1]	OR int[2]	P int[3]
rs1292011	Usual adult BMI (BMI/5), age<54	combined	1250/ 1331		1.02	7.4E-01
rs1292011	Usual adult BMI, age>=54	<25	1393/ 1702	0.89 (0.81-0.99)		
rs1292011	Usual adult BMI, age>=54	25-<30	526/ 627	0.89 (0.75-1.06)		
rs1292011	Usual adult BMI, age>=54	>=30	182/ 215	1.03 (0.78-1.37)		
rs1292011	Usual adult BMI (BMI/5), age>=54	combined	2101/ 2544		0.99	8.5E-01
rs1292011	Usual adult height (cm)	<160	1225/ 1850	0.88 (0.80-0.98)		
rs1292011	Usual adult height (cm)	160-<165	1557/ 2101	0.94 (0.85-1.03)		
rs1292011	Usual adult height (cm)	165-<170	1267/ 1756	1.05 (0.94-1.16)		
rs1292011	Usual adult height (cm)	>=170	956/ 1208	0.93 (0.82-1.06)		
rs1292011	Usual adult height (cm/5)	combined	5005/ 6915		1.03	1.4E-01
rs1292011	Ever use of oral contraceptives	no	1827/ 2240	0.88 (0.80-0.96)		
rs1292011	Ever use of oral contraceptives	yes	2652/ 4089	0.99 (0.92-1.06)		
rs1292011	Ever use of oral contraceptives (yes/no)	combined	4479/ 6329		1.13	4.0E-02
rs1292011	Duration of oral contraceptive use (years)	0	1827/ 2240	0.88 (0.80-0.96)		
rs1292011	Duration of oral contraceptive use (years)	>0-<5	844/ 1335	1.01 (0.89-1.15)		
rs1292011	Duration of oral contraceptive use (years)	5-<10	648/ 1043	0.98 (0.85-1.13)		
rs1292011	Duration of oral contraceptive use (years)	>=10	1101/ 1595	0.95 (0.85-1.06)		
rs1292011	Duration of oral contraceptive use (years/5)	combined	4420/ 6213		1.02	3.2E-01
rs1292011	Current use of combined estrogen/ progestagen MHT	never	783/ 1079	0.93 (0.81-1.07)		
rs1292011	Current use of combined estrogen/ progestagen MHT	EPCurrent	395/ 381	1.00 (0.82-1.22)		
rs1292011	Current use of combined estrogen/ progestagen MHT (yes/no)	combined	1749/ 2267		1.12	3.3E-01
rs1292011	Current use of estrogen only MHT	never	783/ 1079	0.93 (0.81-1.07)		
rs1292011	Current use of estrogen only MHT	ECurrent	169/ 221	0.78 (0.58-1.05)		
rs1292011	Current use of estrogen only MHT (yes/no)	combined	1751/ 2270		0.83	2.5E-01
rs1292011	Duration of combined estrogen/progestagen MHT among current users (years)	never	783/ 1079	0.93 (0.81-1.07)		
rs1292011	Duration of combined estrogen/progestagen MHT among current users (years)	>0-<5	75/ 71	0.97 (0.60-1.59)		
rs1292011	Duration of combined estrogen/progestagen MHT among current users (years)	5-<10	90/ 106	1.14 (0.77-1.68)		
rs1292011	Duration of combined estrogen/progestagen MHT among current users (years)	>=10	180/ 157	1.04 (0.77-1.39)		
rs1292011	Duration of combined estrogen/progestagen MHT among current users (years/5)	combined	1683/ 2192		1.02	6.3E-01
rs1292011	Duration of estrogen only MHT among current users (years)	never	783/ 1079	0.93 (0.81-1.07)		
rs1292011	Duration of estrogen only MHT among current users (years)	>0-<5	34/ 46	0.73 (0.37-1.45)		
rs1292011	Duration of estrogen only MHT among current users (years)	5-<10	43/ 63	0.61 (0.33-1.11)		
rs1292011	Duration of estrogen only MHT among current users (years)	>=10	80/ 96	0.94 (0.62-1.43)		
rs1292011	Duration of estrogen only MHT among current users (years/5)	combined	1722/ 2223		0.97	6.0E-01
rs1292011	Mean lifetime intake of alcohol (g/day)	0	792/ 1055	0.90 (0.79-1.03)		
rs1292011	Mean lifetime intake of alcohol (g/day)	>0-<10	1328/ 1848	0.93 (0.84-1.03)		
rs1292011	Mean lifetime intake of alcohol (g/day)	>=10-<20	263/ 392	0.75 (0.60-0.95)		
rs1292011	Mean lifetime intake of alcohol (g/day)	>=20	211/ 297	1.25 (0.96-1.61)		
rs1292011	Mean lifetime intake of alcohol (10g/day)	combined	2594/ 3592		1.08	1.8E-02
rs1292011	Smoking (ever)	no	2758/ 3889	0.90 (0.84-0.97)		
rs1292011	Smoking (ever)	yes	2294/ 2777	1.02 (0.94-1.10)		
rs1292011	Smoking (ever/never)	combined	5052/ 6666		1.13	2.7E-02
rs1292011	Smoking (pack-years)	0	2590/ 3558	0.88 (0.82-0.95)		
rs1292011	Smoking (pack-years)	0-<10	908/ 1269	0.99 (0.87-1.12)		
rs1292011	Smoking (pack-years)	10-<20	409/ 547	1.09 (0.90-1.31)		
rs1292011	Smoking (pack-years)	>=20	580/ 662	1.04 (0.89-1.23)		
rs1292011	Smoking (pack-years/10)	combined	4487/ 6036		1.03	2.1E-01

SNP	Variable	Stratum	N (cases/ controls)	OR (95% CI)[1]	OR int[2]	P int[3]
rs1292011	Physical activity during year before reference date (h/week)	0	482/ 593	0.79 (0.67-0.94)		
rs1292011	Physical activity during year before reference date (h/week)	0-<3.5	306/ 469	1.01 (0.82-1.24)		
rs1292011	Physical activity during year before reference date (h/week)	3.5-<7	294/ 467	1.04 (0.85-1.28)		
rs1292011	Physical activity during year before reference date (h/week)	>=7	845/ 1182	0.87 (0.77-0.99)		
rs1292011	Physical activity during year before reference date (square root of h/week)	combined	1927/ 2711		1.01	8.5E-01
rs13281615	Age at menarche (years)	<=11	1964/ 3094	1.09 (1.00-1.18)		
rs13281615	Age at menarche (years)	12-13	5860/ 9457	1.18 (1.12-1.24)		
rs13281615	Age at menarche (years)	>=14	4917/ 9023	1.12 (1.06-1.17)		
rs13281615	Age at menarche (years/2)	combined	12741/21574		1.00	9.4E-01
rs13281615	Parous	no	2362/ 3254	1.12 (1.03-1.21)		
rs13281615	Parous	yes	13186/21325	1.15 (1.11-1.18)		
rs13281615	Parous (yes/no)	combined	15548/24579		1.02	5.9E-01
rs13281615	Number of births (among parous)	1	2915/ 4083	1.12 (1.05-1.21)		
rs13281615	Number of births (among parous)	2	5926/ 9535	1.16 (1.10-1.21)		
rs13281615	Number of births (among parous)	3	2751/ 4480	1.09 (1.01-1.17)		
rs13281615	Number of births (among parous)	>=4	1402/ 2390	1.29 (1.17-1.42)		
rs13281615	Number of births (among parous)	combined	12994/20488		1.02	2.1E-01
rs13281615	Age at first birth (among parous, years)	<20	1350/ 2024	1.19 (1.07-1.32)		
rs13281615	Age at first birth (among parous, years)	20-24	4635/ 8350	1.15 (1.09-1.21)		
rs13281615	Age at first birth (among parous, years)	25-29	3383/ 6004	1.11 (1.04-1.18)		
rs13281615	Age at first birth (among parous, years)	>=30	1688/ 2504	1.12 (1.02-1.23)		
rs13281615	Age at first birth (among parous, years/5)	combined	11056/18882		0.98	3.1E-01
rs13281615	Ever breastfed (among parous, yes/no)	no	1153/ 2134	1.01 (0.91-1.13)		
rs13281615	Ever breastfed (among parous, yes/no)	yes	3945/ 7613	1.18 (1.11-1.25)		
rs13281615	Ever breastfed (among parous, yes/no)	combined	5098/ 9747		1.16	1.5E-02
rs13281615	Usual adult BMI, age<54	<25	1543/ 2779	1.12 (1.03-1.23)		
rs13281615	Usual adult BMI, age<54	25-<30	432/ 970	1.25 (1.06-1.48)		
rs13281615	Usual adult BMI, age<54	>=30	183/ 465	1.36 (1.05-1.76)		
rs13281615	Usual adult BMI (BMI/5), age<54	combined	2158/ 4214		1.08	1.1E-01
rs13281615	Usual adult BMI, age>=54	<25	2272/ 3866	0.99 (0.92-1.07)		
rs13281615	Usual adult BMI, age>=54	25-<30	1009/ 1968	1.32 (1.18-1.48)		
rs13281615	Usual adult BMI, age>=54	>=30	399/ 781	1.19 (0.99-1.44)		
rs13281615	Usual adult BMI (BMI/5), age>=54	combined	3680/ 6615		1.11	6.2E-03
rs13281615	Usual adult height (cm)	<160	2219/ 4440	1.12 (1.04-1.21)		
rs13281615	Usual adult height (cm)	160-<165	2729/ 5068	1.16 (1.08-1.24)		
rs13281615	Usual adult height (cm)	165-<170	2274/ 3990	1.13 (1.04-1.22)		
rs13281615	Usual adult height (cm)	>=170	1670/ 2623	1.12 (1.02-1.23)		
rs13281615	Usual adult height (cm/5)	combined	8892/16121		1.00	9.2E-01
rs13281615	Ever use of oral contraceptives	no	3697/ 6696	1.08 (1.02-1.15)		
rs13281615	Ever use of oral contraceptives	yes	3956/ 7978	1.15 (1.09-1.22)		
rs13281615	Ever use of oral contraceptives (yes/no)	combined	7653/14674		1.06	1.5E-01
rs13281615	Duration of oral contraceptive use (years)	0	3697/ 6696	1.08 (1.02-1.15)		
rs13281615	Duration of oral contraceptive use (years)	>0-<5	1353/ 2738	1.11 (1.01-1.23)		
rs13281615	Duration of oral contraceptive use (years)	5-<10	936/ 1992	1.19 (1.06-1.34)		
rs13281615	Duration of oral contraceptive use (years)	>=10	1557/ 2991	1.16 (1.06-1.27)		
rs13281615	Duration of oral contraceptive use (years/5)	combined	7543/14417		1.02	1.2E-01
rs13281615	Current use of combined estrogen/ progestagen MHT	never	1487/ 3116	1.06 (0.97-1.16)		
rs13281615	Current use of combined estrogen/ progestagen MHT	EPCurrent	587/ 729	1.24 (1.06-1.46)		
rs13281615	Current use of combined estrogen/ progestagen MHT (yes/no)	combined	2963/ 5486		1.17	8.7E-02

SNP	Variable	Stratum	N (cases/ controls)	OR (95% CI)[1]	OR int[2]	P int[3]
rs13281615	Current use of estrogen only MHT	never	1590/ 3187	1.06 (0.97-1.16)		
rs13281615	Current use of estrogen only MHT	ECurrent	333/ 601	1.19 (0.98-1.46)		
rs13281615	Current use of estrogen only MHT (yes/no)	combined	3144/ 5640		1.09	4.1E-01
rs13281615	Duration of combined estrogen/progestagen MHT among current users (years)	never	1487/ 3116	1.06 (0.97-1.16)		
rs13281615	Duration of combined estrogen/progestagen MHT among current users (years)	>0-<5	127/ 238	1.16 (0.83-1.61)		
rs13281615	Duration of combined estrogen/progestagen MHT among current users (years)	5-<10	192/ 212	1.71 (1.26-2.34)		
rs13281615	Duration of combined estrogen/progestagen MHT among current users (years)	>=10	213/ 229	0.96 (0.74-1.25)		
rs13281615	Duration of combined estrogen/progestagen MHT among current users (years/5)	combined	2891/ 5404		1.02	5.9E-01
rs13281615	Duration of estrogen only MHT among current users (years)	never	1590/ 3187	1.06 (0.97-1.16)		
rs13281615	Duration of estrogen only MHT among current users (years)	>0-<5	67/ 173	1.25 (0.82-1.92)		
rs13281615	Duration of estrogen only MHT among current users (years)	5-<10	111/ 157	1.01 (0.70-1.46)		
rs13281615	Duration of estrogen only MHT among current users (years)	>=10	140/ 238	1.15 (0.85-1.56)		
rs13281615	Duration of estrogen only MHT among current users (years/5)	combined	3103/ 5535		1.01	8.7E-01
rs13281615	Mean lifetime intake of alcohol (g/day)	0	1358/ 2524	1.17 (1.06-1.29)		
rs13281615	Mean lifetime intake of alcohol (g/day)	>0-<10	1730/ 2810	1.08 (0.99-1.17)		
rs13281615	Mean lifetime intake of alcohol (g/day)	>=10-<20	360/ 580	1.24 (1.03-1.50)		
rs13281615	Mean lifetime intake of alcohol (g/day)	>=20	276/ 378	0.86 (0.69-1.07)		
rs13281615	Mean lifetime intake of alcohol (10g/day)	combined	3724/ 6292		0.97	3.4E-01
rs13281615	Smoking (ever)	no	4720/ 8020	1.11 (1.05-1.17)		
rs13281615	Smoking (ever)	yes	3588/ 6612	1.15 (1.08-1.22)		
rs13281615	Smoking (ever/never)	combined	8308/14632		1.04	4.0E-01
rs13281615	Smoking (pack-years)	0	4150/ 6895	1.10 (1.03-1.16)		
rs13281615	Smoking (pack-years)	0-<10	1357/ 2779	1.12 (1.02-1.23)		
rs13281615	Smoking (pack-years)	10-<20	603/ 1224	1.18 (1.02-1.36)		
rs13281615	Smoking (pack-years)	>=20	845/ 1547	1.06 (0.93-1.21)		
rs13281615	Smoking (pack-years/10)	combined	6955/12445		1.00	8.1E-01
rs13281615	Physical activity during year before reference date (h/week)	0	498/ 999	1.15 (0.97-1.35)		
rs13281615	Physical activity during year before reference date (h/week)	0-<3.5	1048/ 2151	1.17 (1.05-1.31)		
rs13281615	Physical activity during year before reference date (h/week)	3.5-<7	620/ 1321	1.06 (0.92-1.22)		
rs13281615	Physical activity during year before reference date (h/week)	>=7	825/ 1276	1.05 (0.92-1.19)		
rs13281615	Physical activity during year before reference date (square root of h/week)	combined	2991/ 5747		0.98	4.2E-01
rs13387042	Age at menarche (years)	<=11	2164/ 3720	0.90 (0.83-0.97)		
rs13387042	Age at menarche (years)	12-13	6506/12415	0.88 (0.84-0.92)		
rs13387042	Age at menarche (years)	>=14	5752/12786	0.87 (0.83-0.91)		
rs13387042	Age at menarche (years/2)	combined	14422/28921		0.99	6.0E-01
rs13387042	Parous	no	2633/ 4330	0.82 (0.77-0.89)		
rs13387042	Parous	yes	14691/28506	0.88 (0.86-0.91)		
rs13387042	Parous (yes/no)	combined	17324/32836		1.07	9.0E-02
rs13387042	Number of births (among parous)	1	3371/ 5813	0.88 (0.83-0.94)		
rs13387042	Number of births (among parous)	2	6580/13131	0.89 (0.85-0.93)		
rs13387042	Number of births (among parous)	3	3035/ 5779	0.89 (0.83-0.95)		
rs13387042	Number of births (among parous)	>=4	1507/ 2908	0.87 (0.79-0.95)		
rs13387042	Number of births (among parous)	combined	14493/27631		1.00	8.2E-01
rs13387042	Age at first birth (among parous, years)	<20	1487/ 2863	0.88 (0.80-0.97)		
rs13387042	Age at first birth (among parous, years)	20-24	5239/11201	0.84 (0.80-0.89)		
rs13387042	Age at first birth (among parous, years)	25-29	3853/ 8361	0.91 (0.86-0.97)		

SNP	Variable	Stratum	N (cases/ controls)	OR (95% CI)[1]	OR int[2]	P int[3]
rs13387042	Age at first birth (among parous, years)	>=30	1979/ 3600	0.92 (0.84-0.99)		
rs13387042	Age at first birth (among parous, years/5)	combined	12558/26025		1.02	1.7E-01
rs13387042	Ever breastfed (among parous, yes/no)	no	1461/ 2884	0.82 (0.74-0.90)		
rs13387042	Ever breastfed (among parous, yes/no)	yes	4849/10331	0.88 (0.84-0.93)		
rs13387042	Ever breastfed (among parous, yes/no)	combined	6310/13215		1.08	1.4E-01
rs13387042	Usual adult BMI, age<54	<25	1678/ 3046	0.85 (0.78-0.93)		
rs13387042	Usual adult BMI, age<54	25-<30	541/ 1028	0.83 (0.71-0.97)		
rs13387042	Usual adult BMI, age<54	>=30	235/ 508	0.87 (0.69-1.09)		
rs13387042	Usual adult BMI (BMI/5), age<54	combined	2454/ 4582		1.01	8.5E-01
rs13387042	Usual adult BMI, age>=54	<25	3046/ 6492	0.87 (0.82-0.93)		
rs13387042	Usual adult BMI, age>=54	25-<30	1292/ 2766	0.82 (0.74-0.91)		
rs13387042	Usual adult BMI, age>=54	>=30	487/ 954	0.91 (0.76-1.08)		
rs13387042	Usual adult BMI (BMI/5), age>=54	combined	4825/10212		1.02	4.8E-01
rs13387042	Usual adult height (cm)	<160	2550/ 5394	0.86 (0.80-0.92)		
rs13387042	Usual adult height (cm)	160-<165	3221/ 6464	0.85 (0.80-0.91)		
rs13387042	Usual adult height (cm)	165-<170	2743/ 5332	0.85 (0.79-0.91)		
rs13387042	Usual adult height (cm)	>=170	1993/ 3532	0.93 (0.85-1.01)		
rs13387042	Usual adult height (cm/5)	combined	10507/20722		1.02	1.4E-01
rs13387042	Ever use of oral contraceptives	no	4237/ 8529	0.84 (0.79-0.88)		
rs13387042	Ever use of oral contraceptives	yes	4792/10511	0.90 (0.86-0.95)		
rs13387042	Ever use of oral contraceptives (yes/no)	combined	9029/19040		1.08	3.7E-02
rs13387042	Duration of oral contraceptive use (years)	0	4237/ 8529	0.84 (0.79-0.88)		
rs13387042	Duration of oral contraceptive use (years)	>0-<5	1618/ 3488	0.87 (0.80-0.95)		
rs13387042	Duration of oral contraceptive use (years)	5-<10	1138/ 2562	0.87 (0.78-0.97)		
rs13387042	Duration of oral contraceptive use (years)	>=10	1925/ 4155	0.95 (0.88-1.03)		
rs13387042	Duration of oral contraceptive use (years/5)	combined	8918/18734		1.03	3.6E-02
rs13387042	Current use of combined estrogen/ progestagen MHT	never	1863/ 4533	0.87 (0.80-0.94)		
rs13387042	Current use of combined estrogen/ progestagen MHT	EPCurrent	881/ 1373	0.76 (0.67-0.86)		
rs13387042	Current use of combined estrogen/ progestagen MHT (yes/no)	combined	4017/ 9043		0.85	1.8E-02
rs13387042	Current use of estrogen only MHT	never	1965/ 4614	0.87 (0.81-0.94)		
rs13387042	Current use of estrogen only MHT	ECurrent	433/ 1064	0.84 (0.71-0.99)		
rs13387042	Current use of estrogen only MHT (yes/no)	combined	4198/ 9229		0.96	6.4E-01
rs13387042	Duration of combined estrogen/progestagen MHT among current users (years)	never	1863/ 4533	0.86 (0.80-0.94)		
rs13387042	Duration of combined estrogen/progestagen MHT among current users (years)	>0-<5	176/ 373	0.67 (0.52-0.88)		
rs13387042	Duration of combined estrogen/progestagen MHT among current users (years)	5-<10	281/ 415	0.71 (0.56-0.89)		
rs13387042	Duration of combined estrogen/progestagen MHT among current users (years)	>=10	368/ 527	0.81 (0.67-0.99)		
rs13387042	Duration of combined estrogen/progestagen MHT among current users (years/5)	combined	3940/ 8948		0.96	2.2E-01
rs13387042	Duration of estrogen only MHT among current users (years)	never	1965/ 4614	0.87 (0.81-0.94)		
rs13387042	Duration of estrogen only MHT among current users (years)	>0-<5	97/ 299	0.67 (0.48-0.95)		
rs13387042	Duration of estrogen only MHT among current users (years)	5-<10	129/ 288	0.92 (0.68-1.24)		
rs13387042	Duration of estrogen only MHT among current users (years)	>=10	192/ 438	0.86 (0.67-1.10)		
rs13387042	Duration of estrogen only MHT among current users (years/5)	combined	4157/ 9112		0.98	5.5E-01
rs13387042	Mean lifetime intake of alcohol (g/day)	0	1486/ 3007	0.81 (0.74-0.89)		
rs13387042	Mean lifetime intake of alcohol (g/day)	>0-<10	2229/ 4942	0.92 (0.86-0.99)		
rs13387042	Mean lifetime intake of alcohol (g/day)	>=10-<20	466/ 1134	0.92 (0.79-1.07)		
rs13387042	Mean lifetime intake of alcohol (g/day)	>=20	376/ 801	0.84 (0.71-1.00)		
rs13387042	Mean lifetime intake of alcohol (10g/day)	combined	4557/ 9884		1.00	8.3E-01

SNP	Variable	Stratum	N (cases/ controls)	OR (95% CI)[1]	OR int[2]	P int[3]
rs13387042	Smoking (ever)	no	5660/10467	0.86 (0.82-0.90)		
rs13387042	Smoking (ever)	yes	4248/ 8786	0.89 (0.84-0.94)		
rs13387042	Smoking (ever/never)	combined	9908/19253		1.03	3.8E-01
rs13387042	Smoking (pack-years)	0	5067/ 9289	0.86 (0.82-0.90)		
rs13387042	Smoking (pack-years)	0-<10	1602/ 3731	0.92 (0.85-1.01)		
rs13387042	Smoking (pack-years)	10-<20	755/ 1682	0.87 (0.77-0.99)		
rs13387042	Smoking (pack-years)	>=20	1045/ 2211	0.87 (0.78-0.97)		
rs13387042	Smoking (pack-years/10)	combined	8469/16913		0.99	6.0E-01
rs13387042	Physical activity during year before reference date (h/week)	0	517/ 1071	0.96 (0.82-1.12)		
rs13387042	Physical activity during year before reference date (h/week)	0-<3.5	1332/ 2827	0.87 (0.79-0.96)		
rs13387042	Physical activity during year before reference date (h/week)	3.5-<7	967/ 2211	0.85 (0.76-0.95)		
rs13387042	Physical activity during year before reference date (h/week)	>=7	1359/ 3685	0.88 (0.81-0.97)		
rs13387042	Physical activity during year before reference date (square root of h/week)	combined	4175/ 9794		0.98	3.8E-01
rs17468277[7]	Age at menarche (years)	<=11	2129/ 3868	0.92 (0.82-1.04)		
rs17468277	Age at menarche (years)	12-13	6434/12749	0.99 (0.93-1.06)		
rs17468277	Age at menarche (years)	>=14	5589/12811	0.95 (0.89-1.02)		
rs17468277	Age at menarche (years/2)	combined	14152/29428		1.01	8.3E-01
rs17468277	Parous	no	2614/ 4395	1.03 (0.92-1.15)		
rs17468277	Parous	yes	14442/28798	0.97 (0.92-1.01)		
rs17468277	Parous (yes/no)	combined	17056/33193		0.94	3.0E-01
rs17468277	Number of births (among parous)	1	3266/ 5804	1.00 (0.91-1.11)		
rs17468277	Number of births (among parous)	2	6448/13175	0.97 (0.90-1.03)		
rs17468277	Number of births (among parous)	3	3021/ 5942	0.99 (0.90-1.09)		
rs17468277	Number of births (among parous)	>=4	1510/ 3035	0.87 (0.76-1.00)		
rs17468277	Number of births (among parous)	combined	14245/27956		0.97	1.3E-01
rs17468277	Age at first birth (among parous, years)	<20	1445/ 2792	0.89 (0.78-1.03)		
rs17468277	Age at first birth (among parous, years)	20-24	5150/11439	0.98 (0.91-1.05)		
rs17468277	Age at first birth (among parous, years)	25-29	3787/ 8457	0.92 (0.85-1.01)		
rs17468277	Age at first birth (among parous, years)	>=30	1922/ 3653	1.05 (0.93-1.19)		
rs17468277	Age at first birth (among parous, years/5)	combined	12304/26341		1.02	4.5E-01
rs17468277	Ever breastfed (among parous, yes/no)	no	1414/ 2759	1.07 (0.92-1.24)		
rs17468277	Ever breastfed (among parous, yes/no)	yes	4826/10127	0.92 (0.85-0.99)		
rs17468277	Ever breastfed (among parous, yes/no)	combined	6240/12886		0.86	6.7E-02
rs17468277	Usual adult BMI, age<54	<25	1625/ 2777	0.90 (0.79-1.04)		
rs17468277	Usual adult BMI, age<54	25-<30	513/ 925	1.02 (0.80-1.30)		
rs17468277	Usual adult BMI, age<54	>=30	229/ 478	0.84 (0.58-1.21)		
rs17468277	Usual adult BMI (BMI/5), age<54	combined	2367/ 4180		1.00	9.6E-01
rs17468277	Usual adult BMI, age>=54	<25	3058/ 6505	0.99 (0.91-1.09)		
rs17468277	Usual adult BMI, age>=54	25-<30	1297/ 2767	1.01 (0.87-1.17)		
rs17468277	Usual adult BMI, age>=54	>=30	496/ 960	0.96 (0.75-1.24)		
rs17468277	Usual adult BMI (BMI/5), age>=54	combined	4851/10232		1.05	3.2E-01
rs17468277	Usual adult height (cm)	<160	2543/ 5296	1.00 (0.90-1.11)		
rs17468277	Usual adult height (cm)	160-<165	3159/ 6255	0.94 (0.86-1.03)		
rs17468277	Usual adult height (cm)	165-<170	2666/ 5140	0.95 (0.86-1.06)		
rs17468277	Usual adult height (cm)	>=170	1989/ 3433	0.98 (0.87-1.11)		
rs17468277	Usual adult height (cm/5)	combined	10357/20124		0.99	7.1E-01
rs17468277	Ever use of oral contraceptives	no	4306/ 8361	0.94 (0.86-1.02)		
rs17468277	Ever use of oral contraceptives	yes	4843/10344	0.97 (0.90-1.05)		
rs17468277	Ever use of oral contraceptives (yes/no)	combined	9149/18705		1.04	4.8E-01

SNP	Variable	Stratum	N (cases/ controls)	OR (95% CI)[1]	OR int[2]	P int[3]
rs17468277	Duration of oral contraceptive use (years)	0	4306/ 8361	0.94 (0.86-1.02)		
rs17468277	Duration of oral contraceptive use (years)	>0-<5	1645/ 3493	1.02 (0.90-1.16)		
rs17468277	Duration of oral contraceptive use (years)	5-<10	1155/ 2546	0.88 (0.75-1.03)		
rs17468277	Duration of oral contraceptive use (years)	>=10	1934/ 4015	1.00 (0.89-1.13)		
rs17468277	Duration of oral contraceptive use (years/5)	combined	9040/18415		1.01	7.5E-01
rs17468277	Current use of combined estrogen/ progestagen MHT	never	1864/ 4542	1.00 (0.89-1.12)		
rs17468277	Current use of combined estrogen/ progestagen MHT	EPCurrent	884/ 1373	1.04 (0.88-1.25)		
rs17468277	Current use of combined estrogen/ progestagen MHT (yes/no)	combined	4023/ 9060		1.06	5.6E-01
rs17468277	Current use of estrogen only MHT	never	1966/ 4629	0.98 (0.87-1.10)		
rs17468277	Current use of estrogen only MHT	ECurrent	437/ 1063	0.88 (0.69-1.11)		
rs17468277	Current use of estrogen only MHT (yes/no)	combined	4206/ 9253		0.87	2.6E-01
rs17468277	Duration of combined estrogen/progestagen MHT among current users (years)	never	1864/ 4542	1.00 (0.89-1.12)		
rs17468277	Duration of combined estrogen/progestagen MHT among current users (years)	>0-<5	178/ 376	1.16 (0.80-1.70)		
rs17468277	Duration of combined estrogen/progestagen MHT among current users (years)	5-<10	283/ 411	1.05 (0.76-1.45)		
rs17468277	Duration of combined estrogen/progestagen MHT among current users (years)	>=10	367/ 528	1.05 (0.80-1.37)		
rs17468277	Duration of combined estrogen/progestagen MHT among current users (years/5)	combined	3946/ 8965		1.01	7.9E-01
rs17468277	Duration of estrogen only MHT among current users (years)	never	1966/ 4629	0.98 (0.87-1.10)		
rs17468277	Duration of estrogen only MHT among current users (years)	>0-<5	97/ 301	0.84 (0.52-1.37)		
rs17468277	Duration of estrogen only MHT among current users (years)	5-<10	131/ 286	0.83 (0.52-1.33)		
rs17468277	Duration of estrogen only MHT among current users (years)	>=10	194/ 437	0.89 (0.62-1.27)		
rs17468277	Duration of estrogen only MHT among current users (years/5)	combined	4165/ 9136		0.96	4.1E-01
rs17468277	Mean lifetime intake of alcohol (g/day)	0	1441/ 2928	0.91 (0.79-1.04)		
rs17468277	Mean lifetime intake of alcohol (g/day)	>0-<10	2096/ 4586	0.97 (0.87-1.09)		
rs17468277	Mean lifetime intake of alcohol (g/day)	>=10-<20	428/ 1033	0.90 (0.70-1.15)		
rs17468277	Mean lifetime intake of alcohol (g/day)	>=20	345/ 758	1.48 (1.14-1.91)		
rs17468277	Mean lifetime intake of alcohol (10g/day)	combined	4310/ 9305		1.06	6.1E-02
rs17468277	Smoking (ever)	no	5515/10101	0.94 (0.88-1.02)		
rs17468277	Smoking (ever)	yes	4179/ 8408	0.98 (0.90-1.06)		
rs17468277	Smoking (ever/never)	combined	9694/18509		1.04	5.3E-01
rs17468277	Smoking (pack-years)	0	4918/ 8913	0.95 (0.88-1.03)		
rs17468277	Smoking (pack-years)	0-<10	1614/ 3572	0.93 (0.82-1.06)		
rs17468277	Smoking (pack-years)	10-<20	731/ 1583	0.91 (0.75-1.10)		
rs17468277	Smoking (pack-years)	>=20	1025/ 2097	0.97 (0.83-1.15)		
rs17468277	Smoking (pack-years/10)	combined	8288/16165		0.99	6.4E-01
rs17468277	Physical activity during year before reference date (h/week)	0	517/ 1069	1.03 (0.80-1.32)		
rs17468277	Physical activity during year before reference date (h/week)	0-<3.5	1333/ 2830	1.01 (0.88-1.16)		
rs17468277	Physical activity during year before reference date (h/week)	3.5-<7	970/ 2219	0.98 (0.84-1.15)		
rs17468277	Physical activity during year before reference date (h/week)	>=7	1359/ 3684	1.08 (0.95-1.23)		
rs17468277	Physical activity during year before reference date (square root of h/week)	combined	4179/ 9802		0.99	6.4E-01
rs1982073	Age at menarche (years)	<=11	1406/ 2588	1.10 (1.00-1.22)		
rs1982073	Age at menarche (years)	12-13	4114/ 8764	1.06 (1.00-1.12)		
rs1982073	Age at menarche (years)	>=14	3457/ 8570	1.00 (0.94-1.06)		
rs1982073	Age at menarche (years/2)	combined	8977/19922		0.97	1.6E-01
rs1982073	Parous	no	1453/ 2852	0.97 (0.88-1.06)		
rs1982073	Parous	yes	8290/18339	1.04 (1.00-1.09)		
rs1982073	Parous (yes/no)	combined	9743/21191		1.08	1.4E-01

SNP	Variable	Stratum	N (cases/ controls)	OR (95% CI)[1]	OR int[2]	P int[3]
rs1982073	Number of births (among parous)	1	1708/ 3460	1.10 (1.01-1.20)		
rs1982073	Number of births (among parous)	2	3880/ 8602	1.02 (0.97-1.09)		
rs1982073	Number of births (among parous)	3	1723/ 3855	1.04 (0.96-1.14)		
rs1982073	Number of births (among parous)	>=4	817/ 1841	1.03 (0.91-1.16)		
rs1982073	Number of births (among parous)	combined	8128/17758		0.97	1.7E-01
rs1982073	Age at first birth (among parous, years)	<20	1022/ 1736	0.95 (0.85-1.07)		
rs1982073	Age at first birth (among parous, years)	20-24	3354/ 7541	1.09 (1.02-1.16)		
rs1982073	Age at first birth (among parous, years)	25-29	2386/ 5891	1.06 (0.98-1.14)		
rs1982073	Age at first birth (among parous, years)	>=30	1246/ 2528	0.99 (0.89-1.10)		
rs1982073	Age at first birth (among parous, years/5)	combined	8008/17696		0.99	5.5E-01
rs1982073	Ever breastfed (among parous, yes/no)	no	345/ 727	1.24 (1.04-1.49)		
rs1982073	Ever breastfed (among parous, yes/no)	yes	2142/ 4875	1.06 (0.98-1.14)		
rs1982073	Ever breastfed (among parous, yes/no)	combined	2487/ 5602		0.85	1.1E-01
rs1982073	Usual adult BMI, age<54	<25	926/ 1953	1.08 (0.97-1.21)		
rs1982073	Usual adult BMI, age<54	25-<30	246/ 600	1.21 (0.98-1.50)		
rs1982073	Usual adult BMI, age<54	>=30	101/ 271	0.88 (0.63-1.25)		
rs1982073	Usual adult BMI (BMI/5), age<54	combined	1273/ 2824		0.99	8.3E-01
rs1982073	Usual adult BMI, age>=54	<25	830/ 1824	1.07 (0.94-1.21)		
rs1982073	Usual adult BMI, age>=54	25-<30	423/ 1042	0.99 (0.84-1.17)		
rs1982073	Usual adult BMI, age>=54	>=30	164/ 357	1.04 (0.78-1.39)		
rs1982073	Usual adult BMI (BMI/5), age>=54	combined	1417/ 3223		0.98	7.5E-01
rs1982073	Usual adult height (cm)	<160	1186/ 2386	1.02 (0.92-1.13)		
rs1982073	Usual adult height (cm)	160-<165	1507/ 2855	1.08 (0.99-1.19)		
rs1982073	Usual adult height (cm)	165-<170	1178/ 2058	1.02 (0.91-1.13)		
rs1982073	Usual adult height (cm)	>=170	910/ 1311	1.06 (0.94-1.21)		
rs1982073	Usual adult height (cm/5)	combined	4781/ 8610		1.00	9.1E-01
rs1982073	Ever use of oral contraceptives	no	2238/ 4141	1.02 (0.95-1.10)		
rs1982073	Ever use of oral contraceptives	yes	1868/ 3466	1.04 (0.96-1.14)		
rs1982073	Ever use of oral contraceptives (yes/no)	combined	4106/ 7607		1.02	7.4E-01
rs1982073	Duration of oral contraceptive use (years)	0	2238/ 4141	1.02 (0.95-1.10)		
rs1982073	Duration of oral contraceptive use (years)	>0-<5	703/ 1237	1.10 (0.96-1.26)		
rs1982073	Duration of oral contraceptive use (years)	5-<10	470/ 889	0.98 (0.83-1.15)		
rs1982073	Duration of oral contraceptive use (years)	>=10	622/ 1189	0.99 (0.86-1.15)		
rs1982073	Duration of oral contraceptive use (years/5)	combined	4033/ 7456		0.99	7.7E-01
rs1982073	Current use of combined estrogen/ progestagen MHT	never	770/ 1765	1.10 (0.97-1.24)		
rs1982073	Current use of combined estrogen/ progestagen MHT	EPCurrent	175/ 224	0.92 (0.71-1.21)		
rs1982073	Current use of combined estrogen/ progestagen MHT (yes/no)	combined	1211/ 2542		0.84	2.5E-01
rs1982073	Current use of estrogen only MHT	never	870/ 1856	1.08 (0.96-1.22)		
rs1982073	Current use of estrogen only MHT	ECurrent	144/ 234	0.87 (0.65-1.16)		
rs1982073	Current use of estrogen only MHT (yes/no)	combined	1388/ 2743		0.80	1.5E-01
rs1982073	Duration of combined estrogen/progestagen MHT among current users (years)	never	770/ 1765	1.10 (0.97-1.24)		
rs1982073	Duration of combined estrogen/progestagen MHT among current users (years)	>0-<5	56/ 112	0.90 (0.59-1.37)		
rs1982073	Duration of combined estrogen/progestagen MHT among current users (years)	5-<10	84/ 73	0.93 (0.61-1.41)		
rs1982073	Duration of combined estrogen/progestagen MHT among current users (years)	>=10	34/ 39	1.02 (0.52-2.02)		
rs1982073	Duration of combined estrogen/progestagen MHT among current users (years/5)	combined	1210/ 2542		0.98	8.4E-01
rs1982073	Duration of estrogen only MHT among current users (years)	never	870/ 1856	1.08 (0.96-1.22)		
rs1982073	Duration of estrogen only MHT among current users (years)	>0-<5	36/ 105	0.76 (0.46-1.27)		

SNP	Variable	Stratum	N (cases/ controls)	OR (95% CI)[1]	OR int[2]	P int[3]
rs1982073	Duration of estrogen only MHT among current users (years)	5-<10	64/ 69	0.80 (0.49-1.30)		
rs1982073	Duration of estrogen only MHT among current users (years)	>=10	43/ 60	1.33 (0.71-2.47)		
rs1982073	Duration of estrogen only MHT among current users (years/5)	combined	1380/ 2728		0.99	9.5E-01
rs1982073	Mean lifetime intake of alcohol (g/day)	0	865/ 1879	1.00 (0.89-1.12)		
rs1982073	Mean lifetime intake of alcohol (g/day)	>0-<10	568/ 1324	1.22 (1.05-1.40)		
rs1982073	Mean lifetime intake of alcohol (g/day)	>=10-<20	141/ 272	1.34 (0.99-1.81)		
rs1982073	Mean lifetime intake of alcohol (g/day)	>=20	87/ 158	0.77 (0.53-1.13)		
rs1982073	Mean lifetime intake of alcohol (10g/day)	combined	1661/ 3633		1.04	3.5E-01
rs1982073	Smoking (ever)	no	2780/ 4677	1.04 (0.97-1.12)		
rs1982073	Smoking (ever)	yes	2021/ 3781	1.05 (0.97-1.14)		
rs1982073	Smoking (ever/never)	combined	4801/ 8458		1.01	8.8E-01
rs1982073	Smoking (pack-years)	0	2365/ 3827	1.04 (0.96-1.12)		
rs1982073	Smoking (pack-years)	0-<10	781/ 1562	1.00 (0.88-1.13)		
rs1982073	Smoking (pack-years)	10-<20	308/ 671	1.12 (0.92-1.37)		
rs1982073	Smoking (pack-years)	>=20	382/ 713	1.12 (0.94-1.34)		
rs1982073	Smoking (pack-years/10)	combined	3836/ 6773		1.01	6.1E-01
rs1982073	Physical activity during year before reference date (h/week)	0	./ .			
rs1982073	Physical activity during year before reference date (h/week)	0-<3.5	476/ 918	1.00 (0.85-1.17)		
rs1982073	Physical activity during year before reference date (h/week)	3.5-<7	223/ 521	1.11 (0.88-1.40)		
rs1982073	Physical activity during year before reference date (h/week)	>=7	./ .			
rs1982073	Physical activity during year before reference date (square root of h/week)	combined	699/ 1439		0.90	5.9E-01
rs2046210	Age at menarche (years)	<=11	2071/ 3411	1.04 (0.96-1.14)		
rs2046210	Age at menarche (years)	12-13	6086/10928	1.07 (1.02-1.13)		
rs2046210	Age at menarche (years)	>=14	5045/10540	1.08 (1.02-1.14)		
rs2046210	Age at menarche (years/2)	combined	13202/24879		1.01	6.3E-01
rs2046210	Parous	no	2430/ 3622	1.12 (1.03-1.22)		
rs2046210	Parous	yes	13636/24261	1.05 (1.02-1.09)		
rs2046210	Parous (yes/no)	combined	16066/27883		0.94	1.8E-01
rs2046210	Number of births (among parous)	1	2887/ 4597	1.08 (1.01-1.17)		
rs2046210	Number of births (among parous)	2	6080/11054	1.06 (1.00-1.11)		
rs2046210	Number of births (among parous)	3	2863/ 4916	1.04 (0.96-1.11)		
rs2046210	Number of births (among parous)	>=4	1410/ 2487	1.01 (0.91-1.12)		
rs2046210	Number of births (among parous)	combined	13240/23054		0.98	2.1E-01
rs2046210	Age at first birth (among parous, years)	<20	1388/ 2343	0.99 (0.89-1.10)		
rs2046210	Age at first birth (among parous, years)	20-24	4842/ 9507	1.12 (1.06-1.18)		
rs2046210	Age at first birth (among parous, years)	25-29	3603/ 7182	1.01 (0.95-1.08)		
rs2046210	Age at first birth (among parous, years)	>=30	1833/ 3184	1.02 (0.93-1.11)		
rs2046210	Age at first birth (among parous, years/5)	combined	11666/22216		0.98	3.0E-01
rs2046210	Ever breastfed (among parous, yes/no)	no	1325/ 2313	1.04 (0.94-1.16)		
rs2046210	Ever breastfed (among parous, yes/no)	yes	4460/ 7961	1.08 (1.02-1.14)		
rs2046210	Ever breastfed (among parous, yes/no)	combined	5785/10274		1.03	6.1E-01
rs2046210	Usual adult BMI, age<54	<25	1732/ 2887	1.09 (1.00-1.20)		
rs2046210	Usual adult BMI, age<54	25-<30	548/ 1000	1.19 (1.00-1.40)		
rs2046210	Usual adult BMI, age<54	>=30	249/ 501	0.99 (0.78-1.26)		
rs2046210	Usual adult BMI (BMI/5), age<54	combined	2529/ 4388		0.97	5.1E-01
rs2046210	Usual adult BMI, age>=54	<25	2507/ 4084	1.05 (0.97-1.13)		
rs2046210	Usual adult BMI, age>=54	25-<30	1157/ 2118	1.06 (0.94-1.19)		
rs2046210	Usual adult BMI, age>=54	>=30	473/ 854	1.22 (1.01-1.46)		
rs2046210	Usual adult BMI (BMI/5), age>=54	combined	4137/ 7056		1.02	6.6E-01

SNP	Variable	Stratum	N (cases/ controls)	OR (95% CI)[1]	OR int[2]	P int[3]
rs2046210	Usual adult height (cm)	<160	2168/ 4210	1.06 (0.98-1.15)		
rs2046210	Usual adult height (cm)	160-<165	2636/ 4896	1.07 (0.99-1.15)		
rs2046210	Usual adult height (cm)	165-<170	2239/ 3814	1.08 (1.00-1.17)		
rs2046210	Usual adult height (cm)	>=170	1631/ 2523	1.02 (0.92-1.12)		
rs2046210	Usual adult height (cm/5)	combined	8674/15443		0.99	6.6E-01
rs2046210	Ever use of oral contraceptives	no	3788/ 6631	1.07 (1.00-1.14)		
rs2046210	Ever use of oral contraceptives	yes	4250/ 8053	1.05 (0.99-1.12)		
rs2046210	Ever use of oral contraceptives (yes/no)	combined	8038/14684		0.99	8.0E-01
rs2046210	Duration of oral contraceptive use (years)	0	3788/ 6631	1.07 (1.00-1.14)		
rs2046210	Duration of oral contraceptive use (years)	>0-<5	1457/ 2781	1.05 (0.95-1.16)		
rs2046210	Duration of oral contraceptive use (years)	5-<10	1028/ 2012	1.19 (1.06-1.34)		
rs2046210	Duration of oral contraceptive use (years)	>=10	1666/ 3005	0.99 (0.91-1.09)		
rs2046210	Duration of oral contraceptive use (years/5)	combined	7939/14429		0.99	3.8E-01
rs2046210	Current use of combined estrogen/ progestagen MHT	never	1621/ 3278	1.12 (1.02-1.23)		
rs2046210	Current use of combined estrogen/ progestagen MHT	EPCurrent	640/ 783	1.01 (0.86-1.19)		
rs2046210	Current use of combined estrogen/ progestagen MHT (yes/no)	combined	3301/ 5875		0.93	4.1E-01
rs2046210	Current use of estrogen only MHT	never	1723/ 3360	1.11 (1.02-1.22)		
rs2046210	Current use of estrogen only MHT	ECurrent	375/ 673	0.92 (0.75-1.12)		
rs2046210	Current use of estrogen only MHT (yes/no)	combined	3486/ 6060		0.84	9.9E-02
rs2046210	Duration of combined estrogen/progestagen MHT among current users (years)	never	1621/ 3278	1.12 (1.02-1.23)		
rs2046210	Duration of combined estrogen/progestagen MHT among current users (years)	>0-<5	135/ 253	1.02 (0.73-1.42)		
rs2046210	Duration of combined estrogen/progestagen MHT among current users (years)	5-<10	206/ 232	0.85 (0.63-1.15)		
rs2046210	Duration of combined estrogen/progestagen MHT among current users (years)	>=10	243/ 247	1.09 (0.83-1.43)		
rs2046210	Duration of combined estrogen/progestagen MHT among current users (years/5)	combined	3224/ 5791		0.99	7.9E-01
rs2046210	Duration of estrogen only MHT among current users (years)	never	1723/ 3360	1.11 (1.02-1.22)		
rs2046210	Duration of estrogen only MHT among current users (years)	>0-<5	83/ 185	1.04 (0.68-1.59)		
rs2046210	Duration of estrogen only MHT among current users (years)	5-<10	116/ 183	0.95 (0.67-1.36)		
rs2046210	Duration of estrogen only MHT among current users (years)	>=10	161/ 272	0.88 (0.64-1.21)		
rs2046210	Duration of estrogen only MHT among current users (years/5)	combined	3445/ 5947		0.93	1.3E-01
rs2046210	Mean lifetime intake of alcohol (g/day)	0	1332/ 2503	1.10 (1.00-1.22)		
rs2046210	Mean lifetime intake of alcohol (g/day)	>0-<10	1781/ 2857	1.03 (0.94-1.13)		
rs2046210	Mean lifetime intake of alcohol (g/day)	>=10-<20	374/ 593	1.38 (1.14-1.67)		
rs2046210	Mean lifetime intake of alcohol (g/day)	>=20	288/ 389	1.15 (0.92-1.43)		
rs2046210	Mean lifetime intake of alcohol (10g/day)	combined	3775/ 6342		1.04	1.5E-01
rs2046210	Smoking (ever)	no	4453/ 7648	1.06 (1.00-1.12)		
rs2046210	Smoking (ever)	yes	3646/ 6416	1.10 (1.03-1.17)		
rs2046210	Smoking (ever/never)	combined	8099/14064		1.04	4.0E-01
rs2046210	Smoking (pack-years)	0	4041/ 6856	1.04 (0.97-1.10)		
rs2046210	Smoking (pack-years)	0-<10	1439/ 2747	1.12 (1.01-1.24)		
rs2046210	Smoking (pack-years)	10-<20	676/ 1240	1.17 (1.01-1.36)		
rs2046210	Smoking (pack-years)	>=20	935/ 1587	1.07 (0.94-1.21)		
rs2046210	Smoking (pack-years/10)	combined	7091/12430		1.02	3.6E-01
rs2046210	Physical activity during year before reference date (h/week)	0	502/ 1051	1.29 (1.09-1.54)		
rs2046210	Physical activity during year before reference date (h/week)	0-<3.5	1254/ 2439	1.06 (0.95-1.17)		
rs2046210	Physical activity during year before reference date (h/week)	3.5-<7	809/ 1492	1.02 (0.90-1.17)		
rs2046210	Physical activity during year before reference date (h/week)	>=7	890/ 1359	1.00 (0.88-1.14)		

SNP	Variable	Stratum	N (cases/ controls)	OR (95% CI)[1]	OR int[2]	P int[3]
rs2046210	Physical activity during year before reference date (square root of h/week)	combined	3455/ 6341		0.94	2.8E-02
rs2823093	Age at menarche (years)	<=11	1521/ 1810	0.88 (0.78-0.98)		
rs2823093	Age at menarche (years)	12-13	4392/ 5221	0.96 (0.90-1.03)		
rs2823093	Age at menarche (years)	>=14	3807/ 4696	0.90 (0.84-0.97)		
rs2823093	Age at menarche (years/2)	combined	9720/11727		1.00	9.9E-01
rs2823093	Parous	no	1745/ 1972	0.90 (0.81-1.01)		
rs2823093	Parous	yes	10615/12491	0.95 (0.90-0.99)		
rs2823093	Parous (yes/no)	combined	12360/14463		1.05	4.4E-01
rs2823093	Number of births (among parous)	1	2219/ 2264	0.91 (0.82-1.00)		
rs2823093	Number of births (among parous)	2	4845/ 5182	1.01 (0.95-1.08)		
rs2823093	Number of births (among parous)	3	2279/ 2700	0.88 (0.80-0.96)		
rs2823093	Number of births (among parous)	>=4	1116/ 1559	0.93 (0.82-1.05)		
rs2823093	Number of births (among parous)	combined	10459/11705		1.01	6.7E-01
rs2823093	Age at first birth (among parous, years)	<20	1090/ 965	0.88 (0.76-1.01)		
rs2823093	Age at first birth (among parous, years)	20-24	3596/ 4435	0.95 (0.88-1.03)		
rs2823093	Age at first birth (among parous, years)	25-29	2567/ 3382	0.96 (0.88-1.05)		
rs2823093	Age at first birth (among parous, years)	>=30	1314/ 1434	0.95 (0.84-1.08)		
rs2823093	Age at first birth (among parous, years/5)	combined	8567/10216		1.02	4.8E-01
rs2823093	Ever breastfed (among parous, yes/no)	no	706/ 899	0.95 (0.81-1.12)		
rs2823093	Ever breastfed (among parous, yes/no)	yes	2511/ 4441	0.92 (0.84-0.99)		
rs2823093	Ever breastfed (among parous, yes/no)	combined	3217/ 5340		0.96	6.7E-01
rs2823093	Usual adult BMI, age<54	<25	907/ 1108	0.98 (0.84-1.13)		
rs2823093	Usual adult BMI, age<54	25-<30	271/ 391	0.97 (0.74-1.26)		
rs2823093	Usual adult BMI, age<54	>=30	110/ 220	1.02 (0.69-1.49)		
rs2823093	Usual adult BMI (BMI/5), age<54	combined	1288/ 1719		0.98	7.8E-01
rs2823093	Usual adult BMI, age>=54	<25	1627/ 2552	0.92 (0.83-1.02)		
rs2823093	Usual adult BMI, age>=54	25-<30	688/ 1252	0.93 (0.80-1.08)		
rs2823093	Usual adult BMI, age>=54	>=30	248/ 481	0.84 (0.65-1.09)		
rs2823093	Usual adult BMI (BMI/5), age>=54	combined	2563/ 4285		1.00	9.3E-01
rs2823093	Usual adult height (cm)	<160	1264/ 2338	0.93 (0.83-1.04)		
rs2823093	Usual adult height (cm)	160-<165	1727/ 2812	0.92 (0.83-1.01)		
rs2823093	Usual adult height (cm)	165-<170	1484/ 2359	0.94 (0.84-1.05)		
rs2823093	Usual adult height (cm)	>=170	1100/ 1585	0.84 (0.74-0.96)		
rs2823093	Usual adult height (cm/5)	combined	5575/ 9094		0.97	1.2E-01
rs2823093	Ever use of oral contraceptives	no	2408/ 3401	0.94 (0.87-1.03)		
rs2823093	Ever use of oral contraceptives	yes	3109/ 5435	0.88 (0.82-0.94)		
rs2823093	Ever use of oral contraceptives (yes/no)	combined	5517/ 8836		0.93	2.1E-01
rs2823093	Duration of oral contraceptive use (years)	0	2408/ 3401	0.94 (0.87-1.03)		
rs2823093	Duration of oral contraceptive use (years)	>0-<5	993/ 1770	0.94 (0.83-1.06)		
rs2823093	Duration of oral contraceptive use (years)	5-<10	754/ 1343	0.90 (0.77-1.04)		
rs2823093	Duration of oral contraceptive use (years)	>=10	1272/ 2128	0.82 (0.73-0.92)		
rs2823093	Duration of oral contraceptive use (years/5)	combined	5427/ 8642		0.96	8.5E-02
rs2823093	Current use of combined estrogen/ progestagen MHT	never	1187/ 1890	0.85 (0.75-0.96)		
rs2823093	Current use of combined estrogen/ progestagen MHT	EPCurrent	519/ 553	1.00 (0.83-1.20)		
rs2823093	Current use of combined estrogen/ progestagen MHT (yes/no)	combined	2531/ 3709		1.09	4.1E-01
rs2823093	Current use of estrogen only MHT	never	1187/ 1890	0.85 (0.75-0.96)		
rs2823093	Current use of estrogen only MHT	ECurrent	317/ 454	1.18 (0.95-1.48)		
rs2823093	Current use of estrogen only MHT (yes/no)	combined	2543/ 3735		1.32	2.1E-02

SNP	Variable	Stratum	N (cases/ controls)	OR (95% CI)[1]	OR int[2]	P int[3]
rs2823093	Duration of combined estrogen/progestagen MHT among current users (years)	never	1187/ 1890	0.85 (0.75-0.96)		
rs2823093	Duration of combined estrogen/progestagen MHT among current users (years)	>0-<5	104/ 156	1.20 (0.82-1.75)		
rs2823093	Duration of combined estrogen/progestagen MHT among current users (years)	5-<10	150/ 162	1.00 (0.70-1.44)		
rs2823093	Duration of combined estrogen/progestagen MHT among current users (years)	>=10	214/ 186	1.03 (0.77-1.40)		
rs2823093	Duration of combined estrogen/progestagen MHT among current users (years/5)	combined	2462/ 3631		1.03	4.8E-01
rs2823093	Duration of estrogen only MHT among current users (years)	never	1187/ 1890	0.85 (0.75-0.96)		
rs2823093	Duration of estrogen only MHT among current users (years)	>0-<5	74/ 143	1.24 (0.83-1.86)		
rs2823093	Duration of estrogen only MHT among current users (years)	5-<10	101/ 129	1.22 (0.79-1.87)		
rs2823093	Duration of estrogen only MHT among current users (years)	>=10	127/ 165	1.08 (0.76-1.54)		
rs2823093	Duration of estrogen only MHT among current users (years/5)	combined	2510/ 3681		1.06	3.2E-01
rs2823093	Mean lifetime intake of alcohol (g/day)	0	779/ 1044	0.85 (0.73-0.99)		
rs2823093	Mean lifetime intake of alcohol (g/day)	>0-<10	1303/ 1799	0.95 (0.85-1.07)		
rs2823093	Mean lifetime intake of alcohol (g/day)	>=10-<20	258/ 381	0.91 (0.69-1.19)		
rs2823093	Mean lifetime intake of alcohol (g/day)	>=20	202/ 285	1.03 (0.77-1.39)		
rs2823093	Mean lifetime intake of alcohol (10g/day)	combined	2542/ 3509		1.02	5.0E-01
rs2823093	Smoking (ever)	no	3178/ 5150	0.91 (0.85-0.98)		
rs2823093	Smoking (ever)	yes	2442/ 3706	0.89 (0.82-0.97)		
rs2823093	Smoking (ever/never)	combined	5620/ 8856		0.98	7.1E-01
rs2823093	Smoking (pack-years)	0	2612/ 4042	0.92 (0.84-0.99)		
rs2823093	Smoking (pack-years)	0-<10	858/ 1424	0.96 (0.83-1.11)		
rs2823093	Smoking (pack-years)	10-<20	403/ 620	0.93 (0.76-1.14)		
rs2823093	Smoking (pack-years)	>=20	517/ 773	0.84 (0.70-1.02)		
rs2823093	Smoking (pack-years/10)	combined	4390/ 6859		1.00	9.2E-01
rs2823093	Physical activity during year before reference date (h/week)	0	477/ 587	0.81 (0.66-0.98)		
rs2823093	Physical activity during year before reference date (h/week)	0-<3.5	952/ 1578	0.86 (0.75-0.98)		
rs2823093	Physical activity during year before reference date (h/week)	3.5-<7	619/ 1061	1.02 (0.87-1.19)		
rs2823093	Physical activity during year before reference date (h/week)	>=7	832/ 1139	0.97 (0.84-1.12)		
rs2823093	Physical activity during year before reference date (square root of h/week)	combined	2880/ 4365		1.04	1.9E-01
rs2981582	Age at menarche (years)	<=11	2195/ 3999	1.24 (1.14-1.34)		
rs2981582	Age at menarche (years)	12-13	6560/12586	1.32 (1.26-1.38)		
rs2981582	Age at menarche (years)	>=14	5684/11554	1.28 (1.22-1.34)		
rs2981582	Age at menarche (years/2)	combined	14439/28139		1.03	1.9E-01
rs2981582	Parous	no	2654/ 4453	1.36 (1.26-1.46)		
rs2981582	Parous	yes	14776/27332	1.28 (1.24-1.31)		
rs2981582	Parous (yes/no)	combined	17430/31785		0.94	1.4E-01
rs2981582	Number of births (among parous)	1	3358/ 5461	1.28 (1.20-1.37)		
rs2981582	Number of births (among parous)	2	6584/12159	1.25 (1.20-1.31)		
rs2981582	Number of births (among parous)	3	3086/ 5717	1.32 (1.23-1.41)		
rs2981582	Number of births (among parous)	>=4	1555/ 3108	1.28 (1.16-1.40)		
rs2981582	Number of births (among parous)	combined	14583/26445		1.00	8.8E-01
rs2981582	Age at first birth (among parous, years)	<20	1498/ 2638	1.22 (1.11-1.35)		
rs2981582	Age at first birth (among parous, years)	20-24	5266/10922	1.27 (1.21-1.33)		
rs2981582	Age at first birth (among parous, years)	25-29	3838/ 7909	1.31 (1.23-1.39)		
rs2981582	Age at first birth (among parous, years)	>=30	1983/ 3356	1.32 (1.21-1.43)		
rs2981582	Age at first birth (among parous, years/5)	combined	12585/24825		1.02	2.6E-01
rs2981582	Ever breastfed (among parous, yes/no)	no	1445/ 2872	1.22 (1.10-1.34)		

SNP	Variable	Stratum	N (cases/ controls)	OR (95% CI)[1]	OR int[2]	P int[3]
rs2981582	Ever breastfed (among parous, yes/no)	yes	4764/10162	1.29 (1.22-1.36)		
rs2981582	Ever breastfed (among parous, yes/no)	combined	6209/13034		1.06	3.1E-01
rs2981582	Usual adult BMI, age<54	<25	1678/ 3042	1.32 (1.21-1.45)		
rs2981582	Usual adult BMI, age<54	25-<30	530/ 1039	1.40 (1.19-1.64)		
rs2981582	Usual adult BMI, age<54	>=30	232/ 506	1.24 (0.97-1.58)		
rs2981582	Usual adult BMI (BMI/5), age<54	combined	2440/ 4587		1.01	7.7E-01
rs2981582	Usual adult BMI, age>=54	<25	2978/ 6309	1.29 (1.21-1.38)		
rs2981582	Usual adult BMI, age>=54	25-<30	1263/ 2739	1.29 (1.16-1.42)		
rs2981582	Usual adult BMI, age>=54	>=30	476/ 950	1.27 (1.06-1.51)		
rs2981582	Usual adult BMI (BMI/5), age>=54	combined	4717/ 9998		1.01	7.4E-01
rs2981582	Usual adult height (cm)	<160	2605/ 5459	1.33 (1.24-1.42)		
rs2981582	Usual adult height (cm)	160-<165	3240/ 6538	1.23 (1.15-1.31)		
rs2981582	Usual adult height (cm)	165-<170	2765/ 5400	1.30 (1.21-1.39)		
rs2981582	Usual adult height (cm)	>=170	2032/ 3618	1.35 (1.24-1.47)		
rs2981582	Usual adult height (cm/5)	combined	10642/21015		1.01	5.9E-01
rs2981582	Ever use of oral contraceptives	no	4301/ 8278	1.30 (1.23-1.37)		
rs2981582	Ever use of oral contraceptives	yes	4905/11154	1.29 (1.22-1.36)		
rs2981582	Ever use of oral contraceptives (yes/no)	combined	9206/19432		0.99	8.7E-01
rs2981582	Duration of oral contraceptive use (years)	0	4301/ 8278	1.30 (1.23-1.37)		
rs2981582	Duration of oral contraceptive use (years)	>0-<5	1675/ 3815	1.31 (1.21-1.43)		
rs2981582	Duration of oral contraceptive use (years)	5-<10	1155/ 2739	1.27 (1.15-1.41)		
rs2981582	Duration of oral contraceptive use (years)	>=10	1959/ 4299	1.29 (1.19-1.40)		
rs2981582	Duration of oral contraceptive use (years/5)	combined	9090/19131		0.99	6.7E-01
rs2981582	Current use of combined estrogen/ progestagen MHT	never	1845/ 4470	1.30 (1.20-1.41)		
rs2981582	Current use of combined estrogen/ progestagen MHT	EPCurrent	846/ 1320	1.26 (1.10-1.43)		
rs2981582	Current use of combined estrogen/ progestagen MHT (yes/no)	combined	3933/ 8831		0.96	6.1E-01
rs2981582	Current use of estrogen only MHT	never	1931/ 4558	1.31 (1.21-1.42)		
rs2981582	Current use of estrogen only MHT	ECurrent	426/ 1039	1.40 (1.18-1.66)		
rs2981582	Current use of estrogen only MHT (yes/no)	combined	4086/ 9021		1.09	3.6E-01
rs2981582	Duration of combined estrogen/progestagen MHT among current users (years)	never	1845/ 4470	1.30 (1.20-1.41)		
rs2981582	Duration of combined estrogen/progestagen MHT among current users (years)	>0-<5	170/ 369	1.26 (0.95-1.66)		
rs2981582	Duration of combined estrogen/progestagen MHT among current users (years)	5-<10	272/ 397	1.21 (0.96-1.52)		
rs2981582	Duration of combined estrogen/progestagen MHT among current users (years)	>=10	348/ 496	1.28 (1.05-1.56)		
rs2981582	Duration of combined estrogen/progestagen MHT among current users (years/5)	combined	3856/ 8736		0.99	8.0E-01
rs2981582	Duration of estrogen only MHT among current users (years)	never	1931/ 4558	1.31 (1.21-1.42)		
rs2981582	Duration of estrogen only MHT among current users (years)	>0-<5	95/ 295	1.75 (1.21-2.51)		
rs2981582	Duration of estrogen only MHT among current users (years)	5-<10	126/ 282	1.17 (0.86-1.59)		
rs2981582	Duration of estrogen only MHT among current users (years)	>=10	190/ 423	1.42 (1.11-1.83)		
rs2981582	Duration of estrogen only MHT among current users (years/5)	combined	4047/ 8905		1.02	6.0E-01
rs2981582	Mean lifetime intake of alcohol (g/day)	0	1486/ 2986	1.34 (1.22-1.47)		
rs2981582	Mean lifetime intake of alcohol (g/day)	>0-<10	2172/ 4796	1.31 (1.22-1.42)		
rs2981582	Mean lifetime intake of alcohol (g/day)	>=10-<20	452/ 1086	1.06 (0.90-1.25)		
rs2981582	Mean lifetime intake of alcohol (g/day)	>=20	359/ 766	1.28 (1.07-1.54)		
rs2981582	Mean lifetime intake of alcohol (10g/day)	combined	4469/ 9634		0.96	4.5E-02
rs2981582	Smoking (ever)	no	5732/10701	1.29 (1.23-1.35)		
rs2981582	Smoking (ever)	yes	4266/ 8723	1.28 (1.22-1.36)		
rs2981582	Smoking (ever/never)	combined	9998/19424		1.00	9.1E-01

SNP	Variable	Stratum	N (cases/ controls)	OR (95% CI)[1]	OR int[2]	P int[3]
rs2981582	Smoking (pack-years)	0	5140/ 9508	1.30 (1.24-1.37)		
rs2981582	Smoking (pack-years)	0-<10	1588/ 3747	1.25 (1.14-1.36)		
rs2981582	Smoking (pack-years)	10-<20	745/ 1650	1.30 (1.14-1.49)		
rs2981582	Smoking (pack-years)	>=20	1041/ 2170	1.33 (1.19-1.49)		
rs2981582	Smoking (pack-years/10)	combined	8514/17075		1.00	9.9E-01
rs2981582	Physical activity during year before reference date (h/week)	0	515/ 1069	1.33 (1.13-1.58)		
rs2981582	Physical activity during year before reference date (h/week)	0-<3.5	1346/ 2820	1.30 (1.18-1.44)		
rs2981582	Physical activity during year before reference date (h/week)	3.5-<7	960/ 2175	1.29 (1.15-1.44)		
rs2981582	Physical activity during year before reference date (h/week)	>=7	1267/ 3491	1.27 (1.16-1.40)		
rs2981582	Physical activity during year before reference date (square root of h/week)	combined	4088/ 9555		1.01	7.6E-01
rs3803662	Age at menarche (years)	<=11	2051/ 3391	1.28 (1.17-1.40)		
rs3803662	Age at menarche (years)	12-13	6034/10489	1.29 (1.22-1.36)		
rs3803662	Age at menarche (years)	>=14	5057/ 9962	1.22 (1.15-1.29)		
rs3803662	Age at menarche (years/2)	combined	13142/23842		0.97	2.3E-01
rs3803662	Parous	no	2341/ 3545	1.38 (1.26-1.50)		
rs3803662	Parous	yes	13113/23652	1.25 (1.21-1.30)		
rs3803662	Parous (yes/no)	combined	15454/27197		0.91	4.9E-02
rs3803662	Number of births (among parous)	1	2841/ 4601	1.29 (1.20-1.39)		
rs3803662	Number of births (among parous)	2	5874/10467	1.26 (1.20-1.33)		
rs3803662	Number of births (among parous)	3	2799/ 5009	1.29 (1.20-1.39)		
rs3803662	Number of births (among parous)	>=4	1423/ 2729	1.09 (0.98-1.21)		
rs3803662	Number of births (among parous)	combined	12937/22806		0.97	6.6E-02
rs3803662	Age at first birth (among parous, years)	<20	1387/ 2278	1.19 (1.07-1.32)		
rs3803662	Age at first birth (among parous, years)	20-24	4800/ 9459	1.26 (1.20-1.34)		
rs3803662	Age at first birth (among parous, years)	25-29	3476/ 6675	1.26 (1.18-1.34)		
rs3803662	Age at first birth (among parous, years)	>=30	1758/ 2771	1.16 (1.05-1.28)		
rs3803662	Age at first birth (among parous, years/5)	combined	11421/21183		0.99	5.1E-01
rs3803662	Ever breastfed (among parous, yes/no)	no	1256/ 2484	1.20 (1.07-1.34)		
rs3803662	Ever breastfed (among parous, yes/no)	yes	4165/ 8636	1.20 (1.13-1.27)		
rs3803662	Ever breastfed (among parous, yes/no)	combined	5421/11120		1.00	9.9E-01
rs3803662	Usual adult BMI, age<54	<25	1599/ 2793	1.24 (1.13-1.37)		
rs3803662	Usual adult BMI, age<54	25-<30	501/ 955	0.98 (0.83-1.17)		
rs3803662	Usual adult BMI, age<54	>=30	212/ 467	1.23 (0.95-1.60)		
rs3803662	Usual adult BMI (BMI/5), age<54	combined	2312/ 4215		0.98	5.9E-01
rs3803662	Usual adult BMI, age>=54	<25	2367/ 4873	1.24 (1.15-1.35)		
rs3803662	Usual adult BMI, age>=54	25-<30	1081/ 2338	1.21 (1.08-1.37)		
rs3803662	Usual adult BMI, age>=54	>=30	431/ 834	1.17 (0.96-1.43)		
rs3803662	Usual adult BMI (BMI/5), age>=54	combined	3879/ 8045		0.98	5.6E-01
rs3803662	Usual adult height (cm)	<160	2379/ 4876	1.27 (1.17-1.37)		
rs3803662	Usual adult height (cm)	160-<165	2880/ 5547	1.21 (1.12-1.30)		
rs3803662	Usual adult height (cm)	165-<170	2357/ 4423	1.22 (1.12-1.32)		
rs3803662	Usual adult height (cm)	>=170	1709/ 2844	1.32 (1.19-1.45)		
rs3803662	Usual adult height (cm/5)	combined	9325/17690		1.01	7.3E-01
rs3803662	Ever use of oral contraceptives	no	3904/ 7377	1.17 (1.10-1.25)		
rs3803662	Ever use of oral contraceptives	yes	4178/ 8827	1.25 (1.17-1.32)		
rs3803662	Ever use of oral contraceptives (yes/no)	combined	8082/16204		1.06	1.7E-01
rs3803662	Duration of oral contraceptive use (years)	0	3904/ 7377	1.17 (1.10-1.25)		
rs3803662	Duration of oral contraceptive use (years)	>0-<5	1464/ 3027	1.21 (1.09-1.34)		
rs3803662	Duration of oral contraceptive use (years)	5-<10	998/ 2174	1.31 (1.16-1.49)		

SNP	Variable	Stratum	N (cases/ controls)	OR (95% CI)[1]	OR int[2]	P int[3]
rs3803662	Duration of oral contraceptive use (years)	>=10	1604/ 3353	1.26 (1.14-1.39)		
rs3803662	Duration of oral contraceptive use (years/5)	combined	7970/15931		1.03	1.1E-01
rs3803662	Current use of combined estrogen/ progestagen MHT	never	1560/ 3756	1.20 (1.09-1.32)		
rs3803662	Current use of combined estrogen/ progestagen MHT	EPCurrent	578/ 924	1.27 (1.08-1.49)		
rs3803662	Current use of combined estrogen/ progestagen MHT (yes/no)	combined	3071/ 6921		1.05	5.7E-01
rs3803662	Current use of estrogen only MHT	never	1654/ 3829	1.19 (1.09-1.31)		
rs3803662	Current use of estrogen only MHT	ECurrent	342/ 798	1.38 (1.13-1.70)		
rs3803662	Current use of estrogen only MHT (yes/no)	combined	3242/ 7082		1.16	1.8E-01
rs3803662	Duration of combined estrogen/progestagen MHT among current users (years)	never	1560/ 3756	1.20 (1.09-1.32)		
rs3803662	Duration of combined estrogen/progestagen MHT among current users (years)	>0-<5	133/ 293	1.37 (0.99-1.89)		
rs3803662	Duration of combined estrogen/progestagen MHT among current users (years)	5-<10	189/ 281	1.16 (0.87-1.54)		
rs3803662	Duration of combined estrogen/progestagen MHT among current users (years)	>=10	205/ 299	1.36 (1.03-1.80)		
rs3803662	Duration of combined estrogen/progestagen MHT among current users (years/5)	combined	3003/ 6836		1.04	3.9E-01
rs3803662	Duration of estrogen only MHT among current users (years)	never	1654/ 3829	1.19 (1.09-1.31)		
rs3803662	Duration of estrogen only MHT among current users (years)	>0-<5	81/ 235	1.68 (1.13-2.49)		
rs3803662	Duration of estrogen only MHT among current users (years)	5-<10	102/ 214	1.08 (0.73-1.60)		
rs3803662	Duration of estrogen only MHT among current users (years)	>=10	145/ 314	1.29 (0.92-1.79)		
rs3803662	Duration of estrogen only MHT among current users (years/5)	combined	3202/ 6976		1.00	9.6E-01
rs3803662	Mean lifetime intake of alcohol (g/day)	0	1405/ 2784	1.14 (1.03-1.26)		
rs3803662	Mean lifetime intake of alcohol (g/day)	>0-<10	1749/ 3707	1.28 (1.17-1.39)		
rs3803662	Mean lifetime intake of alcohol (g/day)	>=10-<20	354/ 773	1.15 (0.94-1.41)		
rs3803662	Mean lifetime intake of alcohol (g/day)	>=20	267/ 544	0.98 (0.77-1.24)		
rs3803662	Mean lifetime intake of alcohol (10g/day)	combined	3775/ 7808		0.98	4.1E-01
rs3803662	Smoking (ever)	no	5008/ 8933	1.24 (1.17-1.31)		
rs3803662	Smoking (ever)	yes	3660/ 7215	1.22 (1.14-1.30)		
rs3803662	Smoking (ever/never)	combined	8668/16148		0.98	6.8E-01
rs3803662	Smoking (pack-years)	0	4411/ 7745	1.24 (1.16-1.31)		
rs3803662	Smoking (pack-years)	0-<10	1404/ 3076	1.16 (1.04-1.28)		
rs3803662	Smoking (pack-years)	10-<20	615/ 1332	1.19 (1.01-1.39)		
rs3803662	Smoking (pack-years)	>=20	853/ 1706	1.31 (1.14-1.50)		
rs3803662	Smoking (pack-years/10)	combined	7283/13859		1.02	4.2E-01
rs3803662	Physical activity during year before reference date (h/week)	0	514/ 1055	1.14 (0.95-1.38)		
rs3803662	Physical activity during year before reference date (h/week)	0-<3.5	1139/ 2477	1.26 (1.12-1.42)		
rs3803662	Physical activity during year before reference date (h/week)	3.5-<7	691/ 1704	1.03 (0.90-1.19)		
rs3803662	Physical activity during year before reference date (h/week)	>=7	748/ 2104	1.20 (1.05-1.36)		
rs3803662	Physical activity during year before reference date (square root of h/week)	combined	3092/ 7340		0.99	7.1E-01
rs3817198	Age at menarche (years)	<=11	2046/ 3338	1.08 (0.98-1.18)		
rs3817198	Age at menarche (years)	12-13	6022/10153	1.11 (1.05-1.17)		
rs3817198	Age at menarche (years)	>=14	5034/ 9624	1.08 (1.02-1.14)		
rs3817198	Age at menarche (years/2)	combined	13102/23115		1.01	6.6E-01
rs3817198	Parous	no	2387/ 3435	1.09 (1.01-1.19)		
rs3817198	Parous	yes	13489/23015	1.08 (1.05-1.12)		
rs3817198	Parous (yes/no)	combined	15876/26450		0.99	8.1E-01
rs3817198	Number of births (among parous)	1	2970/ 4464	1.02 (0.95-1.10)		
rs3817198	Number of births (among parous)	2	6044/10234	1.05 (1.00-1.11)		
rs3817198	Number of births (among parous)	3	2871/ 4821	1.15 (1.07-1.24)		

SNP	Variable	Stratum	N (cases/ controls)	OR (95% CI)[1]	OR int[2]	P int[3]
rs3817198	Number of births (among parous)	>=4	1453/ 2632	1.26 (1.13-1.40)		
rs3817198	Number of births (among parous)	combined	13338/22151		1.07	5.6E-05
rs3817198	Age at first birth (among parous, years)	<20	1384/ 2202	1.08 (0.97-1.21)		
rs3817198	Age at first birth (among parous, years)	20-24	4806/ 9203	1.09 (1.03-1.15)		
rs3817198	Age at first birth (among parous, years)	25-29	3458/ 6484	1.10 (1.03-1.17)		
rs3817198	Age at first birth (among parous, years)	>=30	1752/ 2691	1.09 (0.98-1.20)		
rs3817198	Age at first birth (among parous, years/5)	combined	11400/20580		1.00	8.7E-01
rs3817198	Ever breastfed (among parous, yes/no)	no	1253/ 2499	1.03 (0.92-1.15)		
rs3817198	Ever breastfed (among parous, yes/no)	yes	4132/ 8692	1.09 (1.03-1.16)		
rs3817198	Ever breastfed (among parous, yes/no)	combined	5385/11191		1.06	3.7E-01
rs3817198	Usual adult BMI, age<54	<25	1578/ 2813	1.09 (0.99-1.20)		
rs3817198	Usual adult BMI, age<54	25-<30	484/ 965	1.15 (0.96-1.38)		
rs3817198	Usual adult BMI, age<54	>=30	205/ 466	1.00 (0.76-1.30)		
rs3817198	Usual adult BMI (BMI/5), age<54	combined	2267/ 4244		0.98	6.3E-01
rs3817198	Usual adult BMI, age>=54	<25	2365/ 4886	1.04 (0.96-1.12)		
rs3817198	Usual adult BMI, age>=54	25-<30	1087/ 2354	1.08 (0.96-1.21)		
rs3817198	Usual adult BMI, age>=54	>=30	432/ 841	1.16 (0.95-1.41)		
rs3817198	Usual adult BMI (BMI/5), age>=54	combined	3884/ 8081		1.04	3.6E-01
rs3817198	Usual adult height (cm)	<160	2377/ 4746	1.10 (1.01-1.19)		
rs3817198	Usual adult height (cm)	160-<165	2849/ 5363	1.10 (1.02-1.18)		
rs3817198	Usual adult height (cm)	165-<170	2323/ 4235	1.08 (0.99-1.17)		
rs3817198	Usual adult height (cm)	>=170	1678/ 2686	1.01 (0.91-1.11)		
rs3817198	Usual adult height (cm/5)	combined	9227/17030		0.98	2.0E-01
rs3817198	Ever use of oral contraceptives	no	3896/ 7403	1.06 (1.00-1.13)		
rs3817198	Ever use of oral contraceptives	yes	4125/ 8856	1.08 (1.02-1.14)		
rs3817198	Ever use of oral contraceptives (yes/no)	combined	8021/16259		1.01	7.5E-01
rs3817198	Duration of oral contraceptive use (years)	0	3896/ 7403	1.06 (1.00-1.13)		
rs3817198	Duration of oral contraceptive use (years)	>0-<5	1446/ 3047	1.11 (1.01-1.23)		
rs3817198	Duration of oral contraceptive use (years)	5-<10	983/ 2176	0.98 (0.87-1.11)		
rs3817198	Duration of oral contraceptive use (years)	>=10	1586/ 3361	1.11 (1.01-1.22)		
rs3817198	Duration of oral contraceptive use (years/5)	combined	7911/15987		1.00	8.5E-01
rs3817198	Current use of combined estrogen/ progestagen MHT	never	1562/ 3759	1.04 (0.94-1.13)		
rs3817198	Current use of combined estrogen/ progestagen MHT	EPCurrent	578/ 928	1.07 (0.91-1.27)		
rs3817198	Current use of combined estrogen/ progestagen MHT (yes/no)	combined	3075/ 6942		1.05	6.0E-01
rs3817198	Current use of estrogen only MHT	never	1665/ 3836	1.04 (0.95-1.14)		
rs3817198	Current use of estrogen only MHT	ECurrent	340/ 808	0.95 (0.77-1.16)		
rs3817198	Current use of estrogen only MHT (yes/no)	combined	3253/ 7105		0.91	3.8E-01
rs3817198	Duration of combined estrogen/progestagen MHT among current users (years)	never	1562/ 3759	1.03 (0.94-1.13)		
rs3817198	Duration of combined estrogen/progestagen MHT among current users (years)	>0-<5	132/ 295	0.94 (0.67-1.32)		
rs3817198	Duration of combined estrogen/progestagen MHT among current users (years)	5-<10	190/ 282	0.94 (0.71-1.25)		
rs3817198	Duration of combined estrogen/progestagen MHT among current users (years)	>=10	205/ 300	1.35 (1.01-1.79)		
rs3817198	Duration of combined estrogen/progestagen MHT among current users (years/5)	combined	3007/ 6857		1.06	1.8E-01
rs3817198	Duration of estrogen only MHT among current users (years)	never	1665/ 3836	1.04 (0.95-1.14)		
rs3817198	Duration of estrogen only MHT among current users (years)	>0-<5	81/ 236	0.81 (0.54-1.23)		
rs3817198	Duration of estrogen only MHT among current users (years)	5-<10	102/ 216	1.14 (0.78-1.66)		
rs3817198	Duration of estrogen only MHT among current users (years)	>=10	143/ 320	1.01 (0.73-1.41)		
rs3817198	Duration of estrogen only MHT among current users (years/5)	combined	3213/ 6998		1.02	7.4E-01

SNP	Variable	Stratum	N (cases/ controls)	OR (95% CI)[1]	OR int[2]	P int[3]
rs3817198	Mean lifetime intake of alcohol (g/day)	0	1402/ 2796	1.11 (1.00-1.23)		
rs3817198	Mean lifetime intake of alcohol (g/day)	>0-<10	1748/ 3712	1.06 (0.97-1.15)		
rs3817198	Mean lifetime intake of alcohol (g/day)	>=10-<20	348/ 777	1.41 (1.16-1.71)		
rs3817198	Mean lifetime intake of alcohol (g/day)	>=20	267/ 545	0.82 (0.66-1.02)		
rs3817198	Mean lifetime intake of alcohol (10g/day)	combined	3765/ 7830		0.98	4.5E-01
rs3817198	Smoking (ever)	no	4935/ 8570	1.07 (1.01-1.13)		
rs3817198	Smoking (ever)	yes	3639/ 6964	1.10 (1.03-1.17)		
rs3817198	Smoking (ever/never)	combined	8574/15534		1.03	5.6E-01
rs3817198	Smoking (pack-years)	0	4341/ 7378	1.08 (1.01-1.15)		
rs3817198	Smoking (pack-years)	0-<10	1390/ 2975	1.12 (1.01-1.24)		
rs3817198	Smoking (pack-years)	10-<20	617/ 1283	1.10 (0.94-1.29)		
rs3817198	Smoking (pack-years)	>=20	848/ 1663	1.10 (0.96-1.27)		
rs3817198	Smoking (pack-years/10)	combined	7196/13299		1.01	7.4E-01
rs3817198	Physical activity during year before reference date (h/week)	0	513/ 1061	1.28 (1.08-1.53)		
rs3817198	Physical activity during year before reference date (h/week)	0-<3.5	1142/ 2492	1.03 (0.92-1.15)		
rs3817198	Physical activity during year before reference date (h/week)	3.5-<7	692/ 1709	1.06 (0.93-1.22)		
rs3817198	Physical activity during year before reference date (h/week)	>=7	748/ 2106	1.01 (0.89-1.14)		
rs3817198	Physical activity during year before reference date (square root of h/week)	combined	3095/ 7368		0.95	7.9E-02
rs4973768	Age at menarche (years)	<=11	2160/ 3680	1.13 (1.05-1.23)		
rs4973768	Age at menarche (years)	12-13	6495/12211	1.07 (1.03-1.12)		
rs4973768	Age at menarche (years)	>=14	5613/12465	1.14 (1.09-1.19)		
rs4973768	Age at menarche (years/2)	combined	14268/28356		1.01	7.6E-01
rs4973768	Parous	no	2620/ 4238	1.11 (1.03-1.19)		
rs4973768	Parous	yes	14502/27677	1.11 (1.08-1.14)		
rs4973768	Parous (yes/no)	combined	17122/31915		1.00	9.9E-01
rs4973768	Number of births (among parous)	1	3284/ 5633	1.06 (0.99-1.13)		
rs4973768	Number of births (among parous)	2	6539/12765	1.12 (1.07-1.17)		
rs4973768	Number of births (among parous)	3	3011/ 5653	1.14 (1.07-1.22)		
rs4973768	Number of births (among parous)	>=4	1481/ 2815	1.15 (1.04-1.26)		
rs4973768	Number of births (among parous)	combined	14315/26866		1.03	7.5E-02
rs4973768	Age at first birth (among parous, years)	<20	1507/ 2811	1.22 (1.12-1.34)		
rs4973768	Age at first birth (among parous, years)	20-24	5267/11142	1.11 (1.06-1.16)		
rs4973768	Age at first birth (among parous, years)	25-29	3882/ 8223	1.10 (1.04-1.16)		
rs4973768	Age at first birth (among parous, years)	>=30	2006/ 3538	1.07 (0.99-1.16)		
rs4973768	Age at first birth (among parous, years/5)	combined	12662/25714		0.97	1.4E-01
rs4973768	Ever breastfed (among parous, yes/no)	no	1444/ 2865	1.10 (1.00-1.21)		
rs4973768	Ever breastfed (among parous, yes/no)	yes	4851/10281	1.15 (1.09-1.21)		
rs4973768	Ever breastfed (among parous, yes/no)	combined	6295/13146		1.04	4.2E-01
rs4973768	Usual adult BMI, age<54	<25	1736/ 3090	1.12 (1.03-1.23)		
rs4973768	Usual adult BMI, age<54	25-<30	553/ 1042	1.06 (0.91-1.24)		
rs4973768	Usual adult BMI, age<54	>=30	247/ 519	1.24 (0.99-1.56)		
rs4973768	Usual adult BMI (BMI/5), age<54	combined	2536/ 4651		1.01	7.1E-01
rs4973768	Usual adult BMI, age>=54	<25	3017/ 6445	1.10 (1.03-1.17)		
rs4973768	Usual adult BMI, age>=54	25-<30	1265/ 2743	1.18 (1.07-1.30)		
rs4973768	Usual adult BMI, age>=54	>=30	483/ 956	1.20 (1.02-1.41)		
rs4973768	Usual adult BMI (BMI/5), age>=54	combined	4765/10144		1.01	7.1E-01
rs4973768	Usual adult height (cm)	<160	2512/ 5110	1.22 (1.14-1.31)		
rs4973768	Usual adult height (cm)	160-<165	3143/ 6190	1.12 (1.05-1.19)		
rs4973768	Usual adult height (cm)	165-<170	2696/ 5106	1.05 (0.98-1.13)		

SNP	Variable	Stratum	N (cases/ controls)	OR (95% CI)[1]	OR int[2]	P int[3]
rs4973768	Usual adult height (cm)	>=170	1996/ 3415	1.14 (1.05-1.23)		
rs4973768	Usual adult height (cm/5)	combined	10347/19821		0.97	2.3E-02
rs4973768	Ever use of oral contraceptives	no	4140/ 7878	1.12 (1.06-1.18)		
rs4973768	Ever use of oral contraceptives	yes	4810/10403	1.14 (1.08-1.20)		
rs4973768	Ever use of oral contraceptives (yes/no)	combined	8950/18281		1.02	6.4E-01
rs4973768	Duration of oral contraceptive use (years)	0	4140/ 7878	1.12 (1.06-1.18)		
rs4973768	Duration of oral contraceptive use (years)	>0-<5	1619/ 3464	1.14 (1.04-1.24)		
rs4973768	Duration of oral contraceptive use (years)	5-<10	1151/ 2582	1.17 (1.06-1.30)		
rs4973768	Duration of oral contraceptive use (years)	>=10	1930/ 4087	1.10 (1.02-1.19)		
rs4973768	Duration of oral contraceptive use (years/5)	combined	8840/18011		1.00	8.2E-01
rs4973768	Current use of combined estrogen/ progestagen MHT	never	1843/ 4500	1.09 (1.01-1.18)		
rs4973768	Current use of combined estrogen/ progestagen MHT	EPCurrent	880/ 1368	1.22 (1.08-1.38)		
rs4973768	Current use of combined estrogen/ progestagen MHT (yes/no)	combined	3993/ 9007		1.11	1.5E-01
rs4973768	Current use of estrogen only MHT	never	1945/ 4577	1.08 (1.00-1.17)		
rs4973768	Current use of estrogen only MHT	ECurrent	434/ 1063	1.06 (0.90-1.25)		
rs4973768	Current use of estrogen only MHT (yes/no)	combined	4175/ 9189		0.93	4.3E-01
rs4973768	Duration of combined estrogen/progestagen MHT among current users (years)	never	1843/ 4500	1.09 (1.01-1.18)		
rs4973768	Duration of combined estrogen/progestagen MHT among current users (years)	>0-<5	175/ 374	1.07 (0.82-1.40)		
rs4973768	Duration of combined estrogen/progestagen MHT among current users (years)	5-<10	281/ 412	1.16 (0.93-1.45)		
rs4973768	Duration of combined estrogen/progestagen MHT among current users (years)	>=10	368/ 524	1.33 (1.10-1.60)		
rs4973768	Duration of combined estrogen/progestagen MHT among current users (years/5)	combined	3916/ 8912		1.05	1.3E-01
rs4973768	Duration of estrogen only MHT among current users (years)	never	1945/ 4577	1.08 (1.00-1.17)		
rs4973768	Duration of estrogen only MHT among current users (years)	>0-<5	96/ 303	1.44 (1.03-2.03)		
rs4973768	Duration of estrogen only MHT among current users (years)	5-<10	131/ 288	1.10 (0.82-1.49)		
rs4973768	Duration of estrogen only MHT among current users (years)	>=10	192/ 433	0.89 (0.70-1.14)		
rs4973768	Duration of estrogen only MHT among current users (years/5)	combined	4134/ 9072		0.94	1.0E-01
rs4973768	Mean lifetime intake of alcohol (g/day)	0	1453/ 2940	1.15 (1.05-1.26)		
rs4973768	Mean lifetime intake of alcohol (g/day)	>0-<10	2206/ 4912	1.13 (1.05-1.21)		
rs4973768	Mean lifetime intake of alcohol (g/day)	>=10-<20	457/ 1127	1.14 (0.97-1.33)		
rs4973768	Mean lifetime intake of alcohol (g/day)	>=20	374/ 787	1.08 (0.91-1.29)		
rs4973768	Mean lifetime intake of alcohol (10g/day)	combined	4490/ 9766		1.01	4.7E-01
rs4973768	Smoking (ever)	no	5530/ 9880	1.07 (1.02-1.12)		
rs4973768	Smoking (ever)	yes	4167/ 8383	1.18 (1.11-1.24)		
rs4973768	Smoking (ever/never)	combined	9697/18263		1.10	1.1E-02
rs4973768	Smoking (pack-years)	0	5113/ 9028	1.07 (1.02-1.13)		
rs4973768	Smoking (pack-years)	0-<10	1627/ 3647	1.12 (1.03-1.23)		
rs4973768	Smoking (pack-years)	10-<20	763/ 1620	1.14 (1.00-1.29)		
rs4973768	Smoking (pack-years)	>=20	1039/ 2137	1.24 (1.11-1.38)		
rs4973768	Smoking (pack-years/10)	combined	8542/16432		1.03	4.3E-02
rs4973768	Physical activity during year before reference date (h/week)	0	517/ 1072	1.13 (0.96-1.33)		
rs4973768	Physical activity during year before reference date (h/week)	0-<3.5	1337/ 2807	1.21 (1.10-1.33)		
rs4973768	Physical activity during year before reference date (h/week)	3.5-<7	960/ 2211	1.07 (0.96-1.20)		
rs4973768	Physical activity during year before reference date (h/week)	>=7	1357/ 3683	1.15 (1.05-1.26)		
rs4973768	Physical activity during year before reference date (square root of h/week)	combined	4171/ 9773		1.00	8.9E-01
rs614367	Age at menarche (years)	<=11	1275/ 2358	1.19 (1.04-1.36)		
rs614367	Age at menarche (years)	12-13	4494/ 8055	1.33 (1.24-1.43)		

SNP	Variable	Stratum	N (cases/ controls)	OR (95% CI)[1]	OR int[2]	P int[3]
rs614367	Age at menarche (years)	>=14	4016/ 7651	1.26 (1.17-1.35)		
rs614367	Age at menarche (years/2)	combined	9785/18064		1.01	7.4E-01
rs614367	Parous	no	1978/ 3050	1.45 (1.29-1.62)		
rs614367	Parous	yes	10329/18334	1.24 (1.18-1.30)		
rs614367	Parous (yes/no)	combined	12307/21384		0.86	1.5E-02
rs614367	Number of births (among parous)	1	2606/ 3980	1.27 (1.15-1.40)		
rs614367	Number of births (among parous)	2	4317/ 7768	1.28 (1.19-1.38)		
rs614367	Number of births (among parous)	3	2072/ 3706	1.21 (1.09-1.36)		
rs614367	Number of births (among parous)	>=4	1173/ 2015	1.16 (1.00-1.34)		
rs614367	Number of births (among parous)	combined	10168/17469		0.97	2.3E-01
rs614367	Age at first birth (among parous, years)	<20	882/ 1857	1.34 (1.15-1.56)		
rs614367	Age at first birth (among parous, years)	20-24	3422/ 6999	1.27 (1.18-1.38)		
rs614367	Age at first birth (among parous, years)	25-29	2612/ 4913	1.29 (1.17-1.42)		
rs614367	Age at first birth (among parous, years)	>=30	1353/ 2093	1.18 (1.03-1.35)		
rs614367	Age at first birth (among parous, years/5)	combined	8269/15862		0.96	1.6E-01
rs614367	Ever breastfed (among parous, yes/no)	no	1406/ 2304	1.34 (1.18-1.52)		
rs614367	Ever breastfed (among parous, yes/no)	yes	4604/ 9427	1.29 (1.21-1.38)		
rs614367	Ever breastfed (among parous, yes/no)	combined	6010/11731		0.97	6.3E-01
rs614367	Usual adult BMI, age<54	<25	1670/ 2720	1.30 (1.16-1.45)		
rs614367	Usual adult BMI, age<54	25-<30	533/ 880	1.38 (1.13-1.68)		
rs614367	Usual adult BMI, age<54	>=30	236/ 418	1.10 (0.81-1.50)		
rs614367	Usual adult BMI (BMI/5), age<54	combined	2439/ 4018		1.01	8.5E-01
rs614367	Usual adult BMI, age>=54	<25	2900/ 5948	1.39 (1.27-1.51)		
rs614367	Usual adult BMI, age>=54	25-<30	1261/ 2494	1.24 (1.09-1.41)		
rs614367	Usual adult BMI, age>=54	>=30	479/ 811	1.28 (1.03-1.61)		
rs614367	Usual adult BMI (BMI/5), age>=54	combined	4640/ 9253		0.94	1.1E-01
rs614367	Usual adult height (cm)	<160	2464/ 4895	1.32 (1.20-1.46)		
rs614367	Usual adult height (cm)	160-<165	3119/ 5892	1.23 (1.13-1.34)		
rs614367	Usual adult height (cm)	165-<170	2691/ 4851	1.27 (1.16-1.40)		
rs614367	Usual adult height (cm)	>=170	1966/ 3204	1.30 (1.17-1.45)		
rs614367	Usual adult height (cm/5)	combined	10240/18842		1.00	9.8E-01
rs614367	Ever use of oral contraceptives	no	4085/ 7377	1.36 (1.27-1.46)		
rs614367	Ever use of oral contraceptives	yes	4746/10179	1.24 (1.16-1.33)		
rs614367	Ever use of oral contraceptives (yes/no)	combined	8831/17556		0.91	5.8E-02
rs614367	Duration of oral contraceptive use (years)	0	4085/ 7377	1.36 (1.27-1.46)		
rs614367	Duration of oral contraceptive use (years)	>0-<5	1577/ 3377	1.21 (1.08-1.36)		
rs614367	Duration of oral contraceptive use (years)	5-<10	1135/ 2481	1.22 (1.06-1.40)		
rs614367	Duration of oral contraceptive use (years)	>=10	1929/ 4044	1.28 (1.15-1.42)		
rs614367	Duration of oral contraceptive use (years/5)	combined	8726/17279		0.98	2.9E-01
rs614367	Current use of combined estrogen/ progestagen MHT	never	1775/ 4073	1.31 (1.18-1.45)		
rs614367	Current use of combined estrogen/ progestagen MHT	EPCurrent	832/ 1189	1.45 (1.22-1.71)		
rs614367	Current use of combined estrogen/ progestagen MHT (yes/no)	combined	3831/ 8117		1.10	3.3E-01
rs614367	Current use of estrogen only MHT	never	1876/ 4143	1.33 (1.21-1.48)		
rs614367	Current use of estrogen only MHT	ECurrent	426/ 916	1.46 (1.18-1.81)		
rs614367	Current use of estrogen only MHT (yes/no)	combined	4011/ 8280		1.09	4.4E-01
rs614367	Duration of combined estrogen/progestagen MHT among current users (years)	never	1775/ 4073	1.31 (1.18-1.45)		
rs614367	Duration of combined estrogen/progestagen MHT among current users (years)	>0-<5	166/ 314	1.71 (1.18-2.46)		
rs614367	Duration of combined estrogen/progestagen MHT among current users (years)	5-<10	265/ 353	1.38 (1.03-1.85)		

SNP	Variable	Stratum	N (cases/ controls)	OR (95% CI)[1]	OR int[2]	P int[3]
rs614367	Duration of combined estrogen/progestagen MHT among current users (years)	>=10	345/ 464	1.48 (1.14-1.93)		
rs614367	Duration of combined estrogen/progestagen MHT among current users (years/5)	combined	3754/ 8022		1.07	1.2E-01
rs614367	Duration of estrogen only MHT among current users (years)	never	1876/ 4143	1.33 (1.21-1.48)		
rs614367	Duration of estrogen only MHT among current users (years)	>0-<5	95/ 280	1.69 (1.12-2.55)		
rs614367	Duration of estrogen only MHT among current users (years)	5-<10	126/ 255	1.16 (0.76-1.78)		
rs614367	Duration of estrogen only MHT among current users (years)	>=10	190/ 358	1.55 (1.12-2.13)		
rs614367	Duration of estrogen only MHT among current users (years/5)	combined	3970/ 8205		1.03	5.7E-01
rs614367	Mean lifetime intake of alcohol (g/day)	0	1338/ 2800	1.38 (1.23-1.54)		
rs614367	Mean lifetime intake of alcohol (g/day)	>0-<10	2063/ 4659	1.31 (1.19-1.45)		
rs614367	Mean lifetime intake of alcohol (g/day)	>=10-<20	418/ 1060	1.47 (1.18-1.83)		
rs614367	Mean lifetime intake of alcohol (g/day)	>=20	339/ 742	1.37 (1.07-1.75)		
rs614367	Mean lifetime intake of alcohol (10g/day)	combined	4158/ 9261		1.01	7.6E-01
rs614367	Smoking (ever)	no	5545/ 9641	1.31 (1.23-1.40)		
rs614367	Smoking (ever)	yes	4137/ 7791	1.25 (1.17-1.35)		
rs614367	Smoking (ever/never)	combined	9682/17432		0.95	3.5E-01
rs614367	Smoking (pack-years)	0	4960/ 8465	1.32 (1.23-1.41)		
rs614367	Smoking (pack-years)	0-<10	1524/ 3368	1.29 (1.15-1.45)		
rs614367	Smoking (pack-years)	10-<20	722/ 1473	1.14 (0.96-1.36)		
rs614367	Smoking (pack-years)	>=20	1016/ 1853	1.33 (1.15-1.55)		
rs614367	Smoking (pack-years/10)	combined	8222/15159		1.00	9.9E-01
rs614367	Physical activity during year before reference date (h/week)	0	515/ 680	1.11 (0.88-1.40)		
rs614367	Physical activity during year before reference date (h/week)	0-<3.5	1336/ 2410	1.33 (1.17-1.52)		
rs614367	Physical activity during year before reference date (h/week)	3.5-<7	952/ 1969	1.33 (1.14-1.54)		
rs614367	Physical activity during year before reference date (h/week)	>=7	1265/ 3344	1.37 (1.21-1.55)		
rs614367	Physical activity during year before reference date (square root of h/week)	combined	4068/ 8403		1.02	5.3E-01
rs6504950	Age at menarche (years)	<=11	2184/ 3700	0.92 (0.84-1.01)		
rs6504950	Age at menarche (years)	12-13	6596/12333	0.93 (0.88-0.97)		
rs6504950	Age at menarche (years)	>=14	5785/12711	0.94 (0.89-0.99)		
rs6504950	Age at menarche (years/2)	combined	14565/28744		1.01	5.8E-01
rs6504950	Parous	no	2652/ 4200	0.91 (0.83-0.98)		
rs6504950	Parous	yes	14834/27832	0.92 (0.89-0.95)		
rs6504950	Parous (yes/no)	combined	17486/32032		1.01	8.0E-01
rs6504950	Number of births (among parous)	1	3383/ 5733	0.92 (0.85-0.98)		
rs6504950	Number of births (among parous)	2	6643/12902	0.91 (0.87-0.96)		
rs6504950	Number of births (among parous)	3	3078/ 5719	0.94 (0.88-1.02)		
rs6504950	Number of births (among parous)	>=4	1531/ 2866	0.94 (0.85-1.04)		
rs6504950	Number of births (among parous)	combined	14635/27220		1.02	2.9E-01
rs6504950	Age at first birth (among parous, years)	<20	1507/ 2802	0.96 (0.87-1.06)		
rs6504950	Age at first birth (among parous, years)	20-24	5291/11124	0.95 (0.90-1.00)		
rs6504950	Age at first birth (among parous, years)	25-29	3883/ 8202	0.88 (0.83-0.94)		
rs6504950	Age at first birth (among parous, years)	>=30	2006/ 3535	0.92 (0.84-1.01)		
rs6504950	Age at first birth (among parous, years/5)	combined	12687/25663		0.97	1.7E-01
rs6504950	Ever breastfed (among parous, yes/no)	no	1462/ 2839	0.96 (0.86-1.07)		
rs6504950	Ever breastfed (among parous, yes/no)	yes	4845/10042	0.90 (0.85-0.95)		
rs6504950	Ever breastfed (among parous, yes/no)	combined	6307/12881		0.94	2.9E-01
rs6504950	Usual adult BMI, age<54	<25	1677/ 2970	0.97 (0.88-1.07)		
rs6504950	Usual adult BMI, age<54	25-<30	542/ 1001	0.94 (0.79-1.13)		
rs6504950	Usual adult BMI, age<54	>=30	235/ 493	1.07 (0.83-1.39)		

SNP	Variable	Stratum	N (cases/ controls)	OR (95% CI)[1]	OR int[2]	P int[3]
rs6504950	Usual adult BMI (BMI/5), age<54	combined	2454/ 4464		1.02	6.8E-01
rs6504950	Usual adult BMI, age>=54	<25	3049/ 6375	0.89 (0.83-0.96)		
rs6504950	Usual adult BMI, age>=54	25-<30	1291/ 2689	0.86 (0.77-0.96)		
rs6504950	Usual adult BMI, age>=54	>=30	489/ 907	0.91 (0.75-1.10)		
rs6504950	Usual adult BMI (BMI/5), age>=54	combined	4829/ 9971		0.99	7.5E-01
rs6504950	Usual adult height (cm)	<160	2576/ 5173	0.93 (0.86-1.01)		
rs6504950	Usual adult height (cm)	160-<165	3260/ 6257	0.90 (0.84-0.96)		
rs6504950	Usual adult height (cm)	165-<170	2762/ 5136	0.93 (0.86-1.00)		
rs6504950	Usual adult height (cm)	>=170	2037/ 3404	0.96 (0.88-1.05)		
rs6504950	Usual adult height (cm/5)	combined	10635/19970		1.00	9.8E-01
rs6504950	Ever use of oral contraceptives	no	4271/ 8070	0.93 (0.88-0.99)		
rs6504950	Ever use of oral contraceptives	yes	4889/10355	0.91 (0.86-0.96)		
rs6504950	Ever use of oral contraceptives (yes/no)	combined	9160/18425		0.97	5.1E-01
rs6504950	Duration of oral contraceptive use (years)	0	4271/ 8070	0.93 (0.88-0.99)		
rs6504950	Duration of oral contraceptive use (years)	>0-<5	1652/ 3444	0.98 (0.89-1.08)		
rs6504950	Duration of oral contraceptive use (years)	5-<10	1160/ 2530	0.86 (0.77-0.97)		
rs6504950	Duration of oral contraceptive use (years)	>=10	1962/ 4103	0.87 (0.80-0.96)		
rs6504950	Duration of oral contraceptive use (years/5)	combined	9045/18147		0.97	4.0E-02
rs6504950	Current use of combined estrogen/ progestagen MHT	never	1859/ 4514	0.88 (0.81-0.96)		
rs6504950	Current use of combined estrogen/ progestagen MHT	EPCurrent	883/ 1373	0.81 (0.70-0.93)		
rs6504950	Current use of combined estrogen/ progestagen MHT (yes/no)	combined	4017/ 9028		0.92	2.8E-01
rs6504950	Current use of estrogen only MHT	never	1961/ 4590	0.90 (0.82-0.98)		
rs6504950	Current use of estrogen only MHT	ECurrent	438/ 1067	0.99 (0.83-1.18)		
rs6504950	Current use of estrogen only MHT (yes/no)	combined	4199/ 9209		1.15	1.6E-01
rs6504950	Duration of combined estrogen/progestagen MHT among current users (years)	never	1859/ 4514	0.88 (0.81-0.96)		
rs6504950	Duration of combined estrogen/progestagen MHT among current users (years)	>0-<5	178/ 376	0.66 (0.49-0.89)		
rs6504950	Duration of combined estrogen/progestagen MHT among current users (years)	5-<10	281/ 415	0.81 (0.63-1.05)		
rs6504950	Duration of combined estrogen/progestagen MHT among current users (years)	>=10	368/ 525	0.84 (0.67-1.04)		
rs6504950	Duration of combined estrogen/progestagen MHT among current users (years/5)	combined	3940/ 8934		0.98	5.5E-01
rs6504950	Duration of estrogen only MHT among current users (years)	never	1961/ 4590	0.90 (0.82-0.98)		
rs6504950	Duration of estrogen only MHT among current users (years)	>0-<5	97/ 303	1.01 (0.71-1.44)		
rs6504950	Duration of estrogen only MHT among current users (years)	5-<10	131/ 288	0.90 (0.64-1.27)		
rs6504950	Duration of estrogen only MHT among current users (years)	>=10	195/ 437	1.00 (0.76-1.32)		
rs6504950	Duration of estrogen only MHT among current users (years/5)	combined	4158/ 9092		1.06	1.8E-01
rs6504950	Mean lifetime intake of alcohol (g/day)	0	1463/ 2945	0.92 (0.83-1.02)		
rs6504950	Mean lifetime intake of alcohol (g/day)	>0-<10	2218/ 4928	0.88 (0.81-0.96)		
rs6504950	Mean lifetime intake of alcohol (g/day)	>=10-<20	462/ 1129	0.80 (0.67-0.96)		
rs6504950	Mean lifetime intake of alcohol (g/day)	>=20	375/ 794	1.00 (0.82-1.22)		
rs6504950	Mean lifetime intake of alcohol (10g/day)	combined	4518/ 9796		1.03	2.1E-01
rs6504950	Smoking (ever)	no	5713/10012	0.93 (0.88-0.98)		
rs6504950	Smoking (ever)	yes	4266/ 8375	0.90 (0.85-0.96)		
rs6504950	Smoking (ever/never)	combined	9979/18387		0.98	5.6E-01
rs6504950	Smoking (pack-years)	0	5117/ 8830	0.92 (0.87-0.98)		
rs6504950	Smoking (pack-years)	0-<10	1618/ 3548	0.92 (0.84-1.02)		
rs6504950	Smoking (pack-years)	10-<20	756/ 1599	0.94 (0.82-1.09)		
rs6504950	Smoking (pack-years)	>=20	1048/ 2095	0.86 (0.75-0.97)		
rs6504950	Smoking (pack-years/10)	combined	8539/16072		1.00	8.0E-01

SNP	Variable	Stratum	N (cases/ controls)	OR (95% CI)[1]	OR int[2]	P int[3]
rs6504950	Physical activity during year before reference date (h/week)	0	517/ 1064	0.98 (0.82-1.18)		
rs6504950	Physical activity during year before reference date (h/week)	0-<3.5	1349/ 2818	0.93 (0.84-1.04)		
rs6504950	Physical activity during year before reference date (h/week)	3.5-<7	973/ 2223	0.85 (0.75-0.96)		
rs6504950	Physical activity during year before reference date (h/week)	>=7	1359/ 3681	0.88 (0.79-0.97)		
rs6504950	Physical activity during year before reference date (square root of h/week)	combined	4198/ 9786		0.98	3.9E-01
rs704010	Age at menarche (years)	<=11	1351/ 2918	1.09 (0.99-1.21)		
rs704010	Age at menarche (years)	12-13	5014/10806	1.04 (0.98-1.09)		
rs704010	Age at menarche (years)	>=14	4697/10866	1.04 (0.98-1.09)		
rs704010	Age at menarche (years/2)	combined	11062/24590		1.00	8.4E-01
rs704010	Parous	no	2184/ 3814	1.09 (1.00-1.18)		
rs704010	Parous	yes	11735/24165	1.05 (1.01-1.08)		
rs704010	Parous (yes/no)	combined	13919/27979		0.96	4.3E-01
rs704010	Number of births (among parous)	1	2881/ 5140	1.03 (0.96-1.11)		
rs704010	Number of births (among parous)	2	5071/11018	1.05 (0.99-1.10)		
rs704010	Number of births (among parous)	3	2340/ 4815	1.07 (0.99-1.16)		
rs704010	Number of births (among parous)	>=4	1243/ 2319	1.05 (0.95-1.17)		
rs704010	Number of births (among parous)	combined	11535/23292		1.00	8.4E-01
rs704010	Age at first birth (among parous, years)	<20	1040/ 2477	1.09 (0.98-1.21)		
rs704010	Age at first birth (among parous, years)	20-24	3974/ 9293	1.03 (0.97-1.09)		
rs704010	Age at first birth (among parous, years)	25-29	3058/ 6910	1.03 (0.97-1.10)		
rs704010	Age at first birth (among parous, years)	>=30	1562/ 2997	1.00 (0.91-1.10)		
rs704010	Age at first birth (among parous, years/5)	combined	9634/21677		0.99	7.1E-01
rs704010	Ever breastfed (among parous, yes/no)	no	1400/ 2296	1.12 (1.01-1.24)		
rs704010	Ever breastfed (among parous, yes/no)	yes	4592/ 9410	1.02 (0.97-1.08)		
rs704010	Ever breastfed (among parous, yes/no)	combined	5992/11706		0.92	1.3E-01
rs704010	Usual adult BMI, age<54	<25	1666/ 2719	1.08 (0.98-1.18)		
rs704010	Usual adult BMI, age<54	25-<30	531/ 879	1.04 (0.88-1.22)		
rs704010	Usual adult BMI, age<54	>=30	234/ 413	1.12 (0.88-1.42)		
rs704010	Usual adult BMI (BMI/5), age<54	combined	2431/ 4011		1.02	6.1E-01
rs704010	Usual adult BMI, age>=54	<25	2897/ 5940	1.03 (0.96-1.10)		
rs704010	Usual adult BMI, age>=54	25-<30	1255/ 2486	1.06 (0.95-1.17)		
rs704010	Usual adult BMI, age>=54	>=30	473/ 806	1.00 (0.84-1.20)		
rs704010	Usual adult BMI (BMI/5), age>=54	combined	4625/ 9232		0.99	8.8E-01
rs704010	Usual adult height (cm)	<160	2459/ 4892	1.04 (0.97-1.12)		
rs704010	Usual adult height (cm)	160-<165	3116/ 5891	1.04 (0.97-1.11)		
rs704010	Usual adult height (cm)	165-<170	2678/ 4848	1.02 (0.95-1.09)		
rs704010	Usual adult height (cm)	>=170	1963/ 3199	1.12 (1.03-1.22)		
rs704010	Usual adult height (cm/5)	combined	10216/18830		1.01	4.0E-01
rs704010	Ever use of oral contraceptives	no	4079/ 7376	1.06 (1.00-1.13)		
rs704010	Ever use of oral contraceptives	yes	4741/10185	1.04 (0.99-1.10)		
rs704010	Ever use of oral contraceptives (yes/no)	combined	8820/17561		0.98	6.4E-01
rs704010	Duration of oral contraceptive use (years)	0	4079/ 7376	1.06 (1.00-1.13)		
rs704010	Duration of oral contraceptive use (years)	>0-<5	1575/ 3383	1.01 (0.92-1.10)		
rs704010	Duration of oral contraceptive use (years)	5-<10	1131/ 2477	1.06 (0.96-1.18)		
rs704010	Duration of oral contraceptive use (years)	>=10	1930/ 4043	1.07 (0.99-1.16)		
rs704010	Duration of oral contraceptive use (years/5)	combined	8715/17279		1.00	8.9E-01
rs704010	Current use of combined estrogen/ progestagen MHT	never	1772/ 4067	1.10 (1.01-1.19)		
rs704010	Current use of combined estrogen/ progestagen MHT	EPCurrent	831/ 1187	1.01 (0.88-1.16)		
rs704010	Current use of combined estrogen/ progestagen MHT (yes/no)	combined	3828/ 8109		0.96	6.1E-01

SNP	Variable	Stratum	N (cases/ controls)	OR (95% CI)[1]	OR int[2]	P int[3]
rs704010	Current use of estrogen only MHT	never	1873/ 4138	1.10 (1.01-1.19)		
rs704010	Current use of estrogen only MHT	ECurrent	426/ 916	1.18 (1.00-1.40)		
rs704010	Current use of estrogen only MHT (yes/no)	combined	4008/ 8272		1.15	1.2E-01
rs704010	Duration of combined estrogen/progestagen MHT among current users (years)	never	1772/ 4067	1.10 (1.01-1.19)		
rs704010	Duration of combined estrogen/progestagen MHT among current users (years)	>0-<5	167/ 313	0.98 (0.74-1.30)		
rs704010	Duration of combined estrogen/progestagen MHT among current users (years)	5-<10	264/ 353	0.94 (0.74-1.20)		
rs704010	Duration of combined estrogen/progestagen MHT among current users (years)	>=10	344/ 463	1.07 (0.86-1.32)		
rs704010	Duration of combined estrogen/progestagen MHT among current users (years/5)	combined	3751/ 8014		1.01	7.2E-01
rs704010	Duration of estrogen only MHT among current users (years)	never	1873/ 4138	1.10 (1.01-1.19)		
rs704010	Duration of estrogen only MHT among current users (years)	>0-<5	95/ 279	1.35 (0.97-1.89)		
rs704010	Duration of estrogen only MHT among current users (years)	5-<10	125/ 255	1.15 (0.85-1.57)		
rs704010	Duration of estrogen only MHT among current users (years)	>=10	191/ 359	1.10 (0.85-1.43)		
rs704010	Duration of estrogen only MHT among current users (years/5)	combined	3967/ 8197		1.02	6.1E-01
rs704010	Mean lifetime intake of alcohol (g/day)	0	1334/ 2802	1.09 (0.99-1.20)		
rs704010	Mean lifetime intake of alcohol (g/day)	>0-<10	2062/ 4653	1.05 (0.97-1.13)		
rs704010	Mean lifetime intake of alcohol (g/day)	>=10-<20	419/ 1060	1.06 (0.90-1.26)		
rs704010	Mean lifetime intake of alcohol (g/day)	>=20	340/ 741	1.02 (0.84-1.24)		
rs704010	Mean lifetime intake of alcohol (10g/day)	combined	4155/ 9256		0.99	5.9E-01
rs704010	Smoking (ever)	no	5534/ 9642	1.09 (1.04-1.15)		
rs704010	Smoking (ever)	yes	4126/ 7785	1.02 (0.96-1.08)		
rs704010	Smoking (ever/never)	combined	9660/17427		0.93	7.6E-02
rs704010	Smoking (pack-years)	0	4950/ 8468	1.09 (1.03-1.15)		
rs704010	Smoking (pack-years)	0-<10	1516/ 3371	1.00 (0.91-1.10)		
rs704010	Smoking (pack-years)	10-<20	724/ 1474	0.95 (0.83-1.09)		
rs704010	Smoking (pack-years)	>=20	1012/ 1846	1.11 (0.98-1.24)		
rs704010	Smoking (pack-years/10)	combined	8202/15159		1.00	8.2E-01
rs704010	Physical activity during year before reference date (h/week)	0	515/ 678	0.97 (0.82-1.15)		
rs704010	Physical activity during year before reference date (h/week)	0-<3.5	1335/ 2413	1.09 (0.98-1.20)		
rs704010	Physical activity during year before reference date (h/week)	3.5-<7	952/ 1966	0.97 (0.87-1.09)		
rs704010	Physical activity during year before reference date (h/week)	>=7	1265/ 3342	1.10 (1.00-1.21)		
rs704010	Physical activity during year before reference date (square root of h/week)	combined	4067/ 8399		1.01	5.5E-01
rs865686	Age at menarche (years)	<=11	2041/ 3572	0.85 (0.78-0.93)		
rs865686	Age at menarche (years)	12-13	6123/11574	0.86 (0.82-0.91)		
rs865686	Age at menarche (years)	>=14	5207/11081	0.90 (0.86-0.95)		
rs865686	Age at menarche (years/2)	combined	13371/26227		1.01	5.2E-01
rs865686	Parous	no	2474/ 4121	0.86 (0.79-0.93)		
rs865686	Parous	yes	13895/25672	0.89 (0.86-0.92)		
rs865686	Parous (yes/no)	combined	16369/29793		1.04	4.1E-01
rs865686	Number of births (among parous)	1	2922/ 5006	0.90 (0.84-0.97)		
rs865686	Number of births (among parous)	2	6269/11760	0.87 (0.83-0.91)[1]		
rs865686	Number of births (among parous)	3	2999/ 5381	0.92 (0.86-0.99)		
rs865686	Number of births (among parous)	>=4	1520/ 2657	0.88 (0.80-0.97)		
rs865686	Number of births (among parous)	combined	13710/24804		1.01	7.1E-01
rs865686	Age at first birth (among parous, years)	<20	1415/ 2406	1.00 (0.91-1.11)		
rs865686	Age at first birth (among parous, years)	20-24	4811/ 9712	0.87 (0.83-0.92)		
rs865686	Age at first birth (among parous, years)	25-29	3641/ 7727	0.86 (0.81-0.92)		

SNP	Variable	Stratum	N (cases/ controls)	OR (95% CI)[1]	OR int[2]	P int[3]
rs865686	Age at first birth (among parous, years)	>=30	1824/ 3325	0.86 (0.79-0.95)		
rs865686	Age at first birth (among parous, years/5)	combined	11691/23170		0.96	5.8E-02
rs865686	Ever breastfed (among parous, yes/no)	no	1189/ 1895	0.84 (0.75-0.94)		
rs865686	Ever breastfed (among parous, yes/no)	yes	3829/ 7795	0.90 (0.85-0.96)		
rs865686	Ever breastfed (among parous, yes/no)	combined	5018/ 9690		1.08	2.5E-01
rs865686	Usual adult BMI, age<54	<25	1240/ 1921	0.85 (0.76-0.95)		
rs865686	Usual adult BMI, age<54	25-<30	462/ 728	0.81 (0.68-0.97)		
rs865686	Usual adult BMI, age<54	>=30	228/ 400	0.89 (0.69-1.15)		
rs865686	Usual adult BMI (BMI/5), age<54	combined	1930/ 3049		1.01	8.3E-01
rs865686	Usual adult BMI, age>=54	<25	2403/ 4973	0.90 (0.83-0.97)		
rs865686	Usual adult BMI, age>=54	25-<30	1107/ 2174	0.87 (0.78-0.97)		
rs865686	Usual adult BMI, age>=54	>=30	457/ 766	0.95 (0.79-1.15)		
rs865686	Usual adult BMI (BMI/5), age>=54	combined	3967/ 7913		1.04	2.9E-01
rs865686	Usual adult height (cm)	<160	2126/ 4115	0.85 (0.79-0.92)		
rs865686	Usual adult height (cm)	160-<165	2655/ 5003	0.91 (0.84-0.97)		
rs865686	Usual adult height (cm)	165-<170	2464/ 4409	0.90 (0.83-0.97)		
rs865686	Usual adult height (cm)	>=170	1817/ 2983	0.84 (0.77-0.93)		
rs865686	Usual adult height (cm/5)	combined	9062/16510		1.00	7.8E-01
rs865686	Ever use of oral contraceptives	no	3137/ 5440	0.86 (0.81-0.92)		
rs865686	Ever use of oral contraceptives	yes	4542/ 9780	0.87 (0.82-0.92)		
rs865686	Ever use of oral contraceptives (yes/no)	combined	7679/15220		1.01	8.4E-01
rs865686	Duration of oral contraceptive use (years)	0	3137/ 5440	0.86 (0.81-0.92)		
rs865686	Duration of oral contraceptive use (years)	>0-<5	1475/ 3184	0.91 (0.83-1.00)		
rs865686	Duration of oral contraceptive use (years)	5-<10	1097/ 2409	0.84 (0.75-0.94)		
rs865686	Duration of oral contraceptive use (years)	>=10	1872/ 3926	0.86 (0.79-0.94)		
rs865686	Duration of oral contraceptive use (years/5)	combined	7581/14959		1.00	7.6E-01
rs865686	Current use of combined estrogen/ progestagen MHT	never	1332/ 3082	0.89 (0.81-0.98)		
rs865686	Current use of combined estrogen/ progestagen MHT	EPCurrent	758/ 1112	0.92 (0.80-1.05)		
rs865686	Current use of combined estrogen/ progestagen MHT (yes/no)	combined	3196/ 6820		1.03	6.8E-01
rs865686	Current use of estrogen only MHT	never	1433/ 3152	0.91 (0.83-1.00)		
rs865686	Current use of estrogen only MHT	ECurrent	402/ 875	1.02 (0.85-1.22)		
rs865686	Current use of estrogen only MHT (yes/no)	combined	3377/ 6985		1.15	1.5E-01
rs865686	Duration of combined estrogen/progestagen MHT among current users (years)	never	1332/ 3082	0.89 (0.81-0.98)		
rs865686	Duration of combined estrogen/progestagen MHT among current users (years)	>0-<5	138/ 277	0.88 (0.65-1.19)		
rs865686	Duration of combined estrogen/progestagen MHT among current users (years)	5-<10	229/ 330	0.93 (0.73-1.19)		
rs865686	Duration of combined estrogen/progestagen MHT among current users (years)	>=10	337/ 450	0.96 (0.78-1.18)		
rs865686	Duration of combined estrogen/progestagen MHT among current users (years/5)	combined	3121/ 6728		1.04	2.5E-01
rs865686	Duration of estrogen only MHT among current users (years)	never	1433/ 3152	0.91 (0.83-1.00)		
rs865686	Duration of estrogen only MHT among current users (years)	>0-<5	89/ 263	1.05 (0.73-1.50)		
rs865686	Duration of estrogen only MHT among current users (years)	5-<10	123/ 240	1.18 (0.85-1.62)		
rs865686	Duration of estrogen only MHT among current users (years)	>=10	176/ 351	0.93 (0.71-1.23)		
rs865686	Duration of estrogen only MHT among current users (years/5)	combined	3338/ 6914		1.03	4.5E-01
rs865686	Mean lifetime intake of alcohol (g/day)	0	671/ 1380	0.98 (0.86-1.12)		
rs865686	Mean lifetime intake of alcohol (g/day)	>0-<10	1705/ 3999	0.85 (0.78-0.93)		
rs865686	Mean lifetime intake of alcohol (g/day)	>=10-<20	370/ 964	0.98 (0.82-1.17)		
rs865686	Mean lifetime intake of alcohol (g/day)	>=20	317/ 693	0.79 (0.65-0.96)		
rs865686	Mean lifetime intake of alcohol (10g/day)	combined	3063/ 7036		0.96	9.5E-02

SNP	Variable	Stratum	N (cases/ controls)	OR (95% CI)[1]	OR int[2]	P int[3]
rs865686	Smoking (ever)	no	5034/ 8586	0.86 (0.81-0.91)		
rs865686	Smoking (ever)	yes	3473/ 6552	0.92 (0.86-0.98)		
rs865686	Smoking (ever/never)	combined	8507/15138		1.07	1.3E-01
rs865686	Smoking (pack-years)	0	4448/ 7418	0.86 (0.82-0.92)		
rs865686	Smoking (pack-years)	0-<10	1143/ 2671	0.91 (0.82-1.01)		
rs865686	Smoking (pack-years)	10-<20	603/ 1199	0.90 (0.77-1.05)		
rs865686	Smoking (pack-years)	>=20	858/ 1600	0.89 (0.78-1.01)		
rs865686	Smoking (pack-years/10)	combined	7052/12888		1.01	6.9E-01
rs865686	Physical activity during year before reference date (h/week)	0	510/ 671	0.84 (0.71-1.00)		
rs865686	Physical activity during year before reference date (h/week)	0-<3.5	1308/ 2375	0.85 (0.77-0.94)		
rs865686	Physical activity during year before reference date (h/week)	3.5-<7	910/ 1942	0.89 (0.79-1.00)		
rs865686	Physical activity during year before reference date (h/week)	>=7	1234/ 3241	0.89 (0.81-0.98)		
rs865686	Physical activity during year before reference date (square root of h/week)	combined	3962/ 8229		1.01	6.4E-01
rs889312	Age at menarche (years)	<=11	2052/ 3407	1.16 (1.06-1.27)		
rs889312	Age at menarche (years)	12-13	6044/10500	1.11 (1.06-1.17)		
rs889312	Age at menarche (years)	>=14	5074/ 9970	1.12 (1.06-1.18)		
rs889312	Age at menarche (years/2)	combined	13170/23877		0.98	4.8E-01
rs889312	Parous	no	2402/ 3512	1.10 (1.01-1.20)		
rs889312	Parous	yes	13526/23500	1.13 (1.10-1.17)		
rs889312	Parous (yes/no)	combined	15928/27012		1.03	4.7E-01
rs889312	Number of births (among parous)	1	2986/ 4631	1.08 (1.00-1.17)		
rs889312	Number of births (among parous)	2	6071/10499	1.18 (1.12-1.24)		
rs889312	Number of births (among parous)	3	2883/ 4998	1.10 (1.02-1.19)		
rs889312	Number of births (among parous)	>=4	1454/ 2723	1.10 (0.99-1.22)		
rs889312	Number of births (among parous)	combined	13394/22851		1.00	1.0E+00
rs889312	Age at first birth (among parous, years)	<20	1387/ 2280	1.16 (1.04-1.29)		
rs889312	Age at first birth (among parous, years)	20-24	4815/ 9480	1.15 (1.09-1.22)		
rs889312	Age at first birth (among parous, years)	25-29	3482/ 6690	1.13 (1.05-1.20)		
rs889312	Age at first birth (among parous, years)	>=30	1769/ 2783	1.07 (0.97-1.18)		
rs889312	Age at first birth (among parous, years/5)	combined	11453/21233		0.98	3.5E-01
rs889312	Ever breastfed (among parous, yes/no)	no	1258/ 2473	1.13 (1.01-1.26)		
rs889312	Ever breastfed (among parous, yes/no)	yes	4139/ 8517	1.14 (1.07-1.21)		
rs889312	Ever breastfed (among parous, yes/no)	combined	5397/10990		1.01	8.8E-01
rs889312	Usual adult BMI, age<54	<25	1576/ 2771	1.16 (1.04-1.28)		
rs889312	Usual adult BMI, age<54	25-<30	482/ 941	1.15 (0.96-1.39)		
rs889312	Usual adult BMI, age<54	>=30	205/ 454	1.09 (0.83-1.44)		
rs889312	Usual adult BMI (BMI/5), age<54	combined	2263/ 4166		0.99	8.7E-01
rs889312	Usual adult BMI, age>=54	<25	2379/ 4812	1.13 (1.04-1.22)		
rs889312	Usual adult BMI, age>=54	25-<30	1085/ 2300	1.12 (1.00-1.27)		
rs889312	Usual adult BMI, age>=54	>=30	434/ 810	1.10 (0.90-1.35)		
rs889312	Usual adult BMI (BMI/5), age>=54	combined	3898/ 7922		0.98	6.1E-01
rs889312	Usual adult height (cm)	<160	2379/ 4818	1.14 (1.05-1.24)		
rs889312	Usual adult height (cm)	160-<165	2866/ 5487	1.10 (1.02-1.18)		
rs889312	Usual adult height (cm)	165-<170	2342/ 4378	1.16 (1.07-1.26)		
rs889312	Usual adult height (cm)	>=170	1689/ 2803	1.05 (0.95-1.16)		
rs889312	Usual adult height (cm/5)	combined	9276/17486		0.98	3.0E-01
rs889312	Ever use of oral contraceptives	no	3921/ 7370	1.12 (1.05-1.20)		
rs889312	Ever use of oral contraceptives	yes	4111/ 8656	1.13 (1.06-1.20)		
rs889312	Ever use of oral contraceptives (yes/no)	combined	8032/16026		1.00	9.3E-01

SNP	Variable	Stratum	N (cases/ controls)	OR (95% CI)[1]	OR int[2]	P int[3]
rs889312	Duration of oral contraceptive use (years)	0	3921/ 7370	1.12 (1.05-1.20)		
rs889312	Duration of oral contraceptive use (years)	>0-<5	1447/ 2994	1.16 (1.05-1.29)		
rs889312	Duration of oral contraceptive use (years)	5-<10	974/ 2124	1.09 (0.96-1.23)		
rs889312	Duration of oral contraceptive use (years)	>=10	1580/ 3271	1.12 (1.01-1.23)		
rs889312	Duration of oral contraceptive use (years/5)	combined	7922/15759		1.00	7.8E-01
rs889312	Current use of combined estrogen/ progestagen MHT	never	1573/ 3776	1.12 (1.02-1.23)		
rs889312	Current use of combined estrogen/ progestagen MHT	EPCurrent	581/ 923	1.19 (1.01-1.42)		
rs889312	Current use of combined estrogen/ progestagen MHT (yes/no)	combined	3090/ 6950		1.06	5.3E-01
rs889312	Current use of estrogen only MHT	never	1678/ 3853	1.12 (1.02-1.22)		
rs889312	Current use of estrogen only MHT	ECurrent	342/ 802	1.16 (0.95-1.43)		
rs889312	Current use of estrogen only MHT (yes/no)	combined	3274/ 7117		1.03	8.0E-01
rs889312	Duration of combined estrogen/progestagen MHT among current users (years)	never	1573/ 3776	1.12 (1.02-1.23)		
rs889312	Duration of combined estrogen/progestagen MHT among current users (years)	>0-<5	134/ 292	0.97 (0.69-1.38)		
rs889312	Duration of combined estrogen/progestagen MHT among current users (years)	5-<10	190/ 280	1.30 (0.96-1.75)		
rs889312	Duration of combined estrogen/progestagen MHT among current users (years)	>=10	206/ 300	1.12 (0.83-1.50)		
rs889312	Duration of combined estrogen/progestagen MHT among current users (years/5)	combined	3022/ 6865		1.02	6.2E-01
rs889312	Duration of estrogen only MHT among current users (years)	never	1678/ 3853	1.12 (1.02-1.22)		
rs889312	Duration of estrogen only MHT among current users (years)	>0-<5	81/ 235	1.12 (0.76-1.66)		
rs889312	Duration of estrogen only MHT among current users (years)	5-<10	102/ 213	1.51 (1.01-2.24)		
rs889312	Duration of estrogen only MHT among current users (years)	>=10	145/ 318	1.14 (0.81-1.61)		
rs889312	Duration of estrogen only MHT among current users (years/5)	combined	3234/ 7010		1.02	6.3E-01
rs889312	Mean lifetime intake of alcohol (g/day)	0	1422/ 2807	1.19 (1.07-1.31)		
rs889312	Mean lifetime intake of alcohol (g/day)	>0-<10	1760/ 3719	1.07 (0.98-1.17)		
rs889312	Mean lifetime intake of alcohol (g/day)	>=10-<20	351/ 775	1.26 (1.04-1.53)		
rs889312	Mean lifetime intake of alcohol (g/day)	>=20	269/ 544	1.16 (0.93-1.46)		
rs889312	Mean lifetime intake of alcohol (10g/day)	combined	3802/ 7845		1.00	9.5E-01
rs889312	Smoking (ever)	no	4984/ 8828	1.14 (1.07-1.20)		
rs889312	Smoking (ever)	yes	3647/ 7115	1.09 (1.02-1.17)		
rs889312	Smoking (ever/never)	combined	8631/15943		0.96	3.9E-01
rs889312	Smoking (pack-years)	0	4386/ 7637	1.12 (1.06-1.19)		
rs889312	Smoking (pack-years)	0-<10	1397/ 3024	1.14 (1.03-1.26)		
rs889312	Smoking (pack-years)	10-<20	615/ 1308	1.21 (1.03-1.42)		
rs889312	Smoking (pack-years)	>=20	849/ 1692	1.06 (0.92-1.22)		
rs889312	Smoking (pack-years/10)	combined	7247/13661		0.99	5.6E-01
rs889312	Physical activity during year before reference date (h/week)	0	514/ 1059	1.28 (1.07-1.53)		
rs889312	Physical activity during year before reference date (h/week)	0-<3.5	1144/ 2480	1.09 (0.97-1.22)		
rs889312	Physical activity during year before reference date (h/week)	3.5-<7	693/ 1708	1.15 (1.00-1.33)		
rs889312	Physical activity during year before reference date (h/week)	>=7	748/ 2104	1.08 (0.95-1.23)		
rs889312	Physical activity during year before reference date (square root of h/week)	combined	3099/ 7351		0.96	1.6E-01
rs999737[8]	Age at menarche (years)	<=11	2140/ 3486	0.93 (0.84-1.02)		
rs999737	Age at menarche (years)	12-13	6401/11398	0.97 (0.92-1.03)		
rs999737	Age at menarche (years)	>=14	5423/11067	0.91 (0.86-0.96)		
rs999737	Age at menarche (years/2)	combined	13964/25951		0.98	4.4E-01
rs999737	Parous	no	2513/ 3775	0.87 (0.80-0.96)		
rs999737	Parous	yes	14302/25161	0.94 (0.91-0.98)		
rs999737	Parous (yes/no)	combined	16815/28936		1.08	1.3E-01

SNP	Variable	Stratum	N (cases/ controls)	OR (95% CI)[1]	OR int[2]	P int[3]
rs999737	Number of births (among parous)	1	3098/ 4849	0.92 (0.85-1.00)		
rs999737	Number of births (among parous)	2	6358/11402	0.96 (0.91-1.01)		
rs999737	Number of births (among parous)	3	2965/ 5135	0.92 (0.85-0.99)		
rs999737	Number of births (among parous)	>=4	1468/ 2573	0.95 (0.85-1.07)		
rs999737	Number of births (among parous)	combined	13889/23959		1.00	8.9E-01
rs999737	Age at first birth (among parous, years)	<20	1437/ 2385	0.95 (0.85-1.07)		
rs999737	Age at first birth (among parous, years)	20-24	4987/ 9682	0.94 (0.88-0.99)		
rs999737	Age at first birth (among parous, years)	25-29	3746/ 7344	0.95 (0.88-1.02)		
rs999737	Age at first birth (among parous, years)	>=30	1893/ 3243	0.95 (0.86-1.06)		
rs999737	Age at first birth (among parous, years/5)	combined	12063/22654		1.00	9.4E-01
rs999737	Ever breastfed (among parous, yes/no)	no	1317/ 2253	0.99 (0.88-1.12)		
rs999737	Ever breastfed (among parous, yes/no)	yes	4378/ 7767	0.89 (0.84-0.95)		
rs999737	Ever breastfed (among parous, yes/no)	combined	5695/10020		0.90	1.4E-01
rs999737	Usual adult BMI, age<54	<25	1739/ 2862	0.98 (0.88-1.09)		
rs999737	Usual adult BMI, age<54	25-<30	552/ 989	0.85 (0.70-1.02)		
rs999737	Usual adult BMI, age<54	>=30	247/ 497	0.79 (0.60-1.05)		
rs999737	Usual adult BMI (BMI/5), age<54	combined	2538/ 4348		0.87	5.1E-03
rs999737	Usual adult BMI, age>=54	<25	2451/ 3954	0.88 (0.81-0.97)		
rs999737	Usual adult BMI, age>=54	25-<30	1099/ 1988	0.97 (0.85-1.10)		
rs999737	Usual adult BMI, age>=54	>=30	462/ 822	0.91 (0.74-1.12)		
rs999737	Usual adult BMI (BMI/5), age>=54	combined	4012/ 6764		1.02	6.1E-01
rs999737	Usual adult height (cm)	<160	2323/ 4444	0.91 (0.83-1.00)		
rs999737	Usual adult height (cm)	160-<165	2892/ 5155	0.91 (0.84-0.99)		
rs999737	Usual adult height (cm)	165-<170	2426/ 4091	0.89 (0.81-0.97)		
rs999737	Usual adult height (cm)	>=170	1773/ 2706	1.00 (0.90-1.11)		
rs999737	Usual adult height (cm/5)	combined	9414/16396		1.03	1.3E-01
rs999737	Ever use of oral contraceptives	no	3828/ 6663	0.93 (0.87-1.00)		
rs999737	Ever use of oral contraceptives	yes	4366/ 8157	0.92 (0.86-0.98)		
rs999737	Ever use of oral contraceptives (yes/no)	combined	8194/14820		0.99	8.0E-01
rs999737	Duration of oral contraceptive use (years)	0	3828/ 6663	0.93 (0.87-1.00)		
rs999737	Duration of oral contraceptive use (years)	>0-<5	1478/ 2778	0.93 (0.83-1.04)		
rs999737	Duration of oral contraceptive use (years)	5-<10	1049/ 2016	0.94 (0.83-1.08)		
rs999737	Duration of oral contraceptive use (years)	>=10	1739/ 3112	0.91 (0.81-1.01)		
rs999737	Duration of oral contraceptive use (years/5)	combined	8094/14569		1.00	8.8E-01
rs999737	Current use of combined estrogen/ progestagen MHT	never	1542/ 3115	0.93 (0.84-1.03)		
rs999737	Current use of combined estrogen/ progestagen MHT	EPCurrent	625/ 755	0.83 (0.68-1.00)		
rs999737	Current use of combined estrogen/ progestagen MHT (yes/no)	combined	3167/ 5600		0.89	3.0E-01
rs999737	Current use of estrogen only MHT	never	1645/ 3196	0.94 (0.85-1.04)		
rs999737	Current use of estrogen only MHT	ECurrent	356/ 640	0.93 (0.74-1.17)		
rs999737	Current use of estrogen only MHT (yes/no)	combined	3350/ 5779		1.01	9.2E-01
rs999737	Duration of combined estrogen/progestagen MHT among current users (years)	never	1542/ 3115	0.93 (0.83-1.03)		
rs999737	Duration of combined estrogen/progestagen MHT among current users (years)	>0-<5	125/ 234	1.00 (0.67-1.50)		
rs999737	Duration of combined estrogen/progestagen MHT among current users (years)	5-<10	203/ 229	0.76 (0.55-1.07)		
rs999737	Duration of combined estrogen/progestagen MHT among current users (years)	>=10	241/ 241	0.70 (0.51-0.98)		
rs999737	Duration of combined estrogen/progestagen MHT among current users (years/5)	combined	3090/ 5516		0.93	1.8E-01
rs999737	Duration of estrogen only MHT among current users (years)	never	1645/ 3196	0.94 (0.84-1.04)		
rs999737	Duration of estrogen only MHT among current users (years)	>0-<5	74/ 175	0.82 (0.49-1.36)		

SNP	Variable	Stratum	N (cases/ controls)	OR (95% CI)[1]	OR int[2]	P int[3]
rs999737	Duration of estrogen only MHT among current users (years)	5-<10	112/ 173	0.72 (0.46-1.13)		
rs999737	Duration of estrogen only MHT among current users (years)	>=10	156/ 258	1.23 (0.87-1.74)		
rs999737	Duration of estrogen only MHT among current users (years/5)	combined	3310/ 5667		1.07	1.8E-01
rs999737	Mean lifetime intake of alcohol (g/day)	0	1358/ 2533	0.92 (0.82-1.03)		
rs999737	Mean lifetime intake of alcohol (g/day)	>0-<10	1781/ 2864	0.91 (0.82-1.00)		
rs999737	Mean lifetime intake of alcohol (g/day)	>=10-<20	375/ 590	0.73 (0.59-0.92)		
rs999737	Mean lifetime intake of alcohol (g/day)	>=20	288/ 394	1.20 (0.93-1.55)		
rs999737	Mean lifetime intake of alcohol (10g/day)	combined	3802/ 6381		1.03	2.9E-01
rs999737	Smoking (ever)	no	5007/ 8263	0.92 (0.86-0.98)		
rs999737	Smoking (ever)	yes	3838/ 6774	0.92 (0.85-0.99)		
rs999737	Smoking (ever/never)	combined	8845/15037		1.00	1.0E+00
rs999737	Smoking (pack-years)	0	4522/ 7263	0.93 (0.87-1.00)		
rs999737	Smoking (pack-years)	0-<10	1466/ 2881	0.91 (0.82-1.02)		
rs999737	Smoking (pack-years)	10-<20	691/ 1292	0.96 (0.82-1.13)		
rs999737	Smoking (pack-years)	>=20	951/ 1629	0.85 (0.73-0.98)		
rs999737	Smoking (pack-years/10)	combined	7630/13065		1.00	8.7E-01
rs999737	Physical activity during year before reference date (h/week)	0	502/ 1004	0.99 (0.81-1.19)		
rs999737	Physical activity during year before reference date (h/week)	0-<3.5	1142/ 2238	0.95 (0.84-1.08)		
rs999737	Physical activity during year before reference date (h/week)	3.5-<7	759/ 1392	0.86 (0.74-1.01)		
rs999737	Physical activity during year before reference date (h/week)	>=7	890/ 1342	0.95 (0.83-1.10)		
rs999737	Physical activity during year before reference date (square root of h/week)	combined	3293/ 5976		0.98	4.9E-01

[1] Per-allele SNP effect in each stratum of environmental risk factors using case-control analysis adjusted for study and reference age
[2] OR for GxE interaction from case-control analysis stratified by study and adjusted for reference age
[3] p value for case-control analysis stratified by study and adjusted for reference age
[4] model used never use of MHT (menopausal hormone therapy) as the reference category and adjusted for former use of MHT and current use of other MHT type, as appropriate
[5] mean lifetime alcohol intake derived from duration and amount of alcohol intake in g/day at different age periods
[6] or the highly correlated SNP rs1975930 (r^2= 1 in HapMap CEU)
[7] or the highly correlated SNP rs1045485 (r^2= 1 in HapMap CEU)
[8] or the highly correlated SNP rs10483813 (r^2= 1 in HapMap CEU)
