# Supplementary material for: Evidence of Gene–Environment Interactions between Common Breast Cancer Susceptibility Loci and Established Environmental Risk Factors
Source: PLoS Genet. 2013 Mar 27;9(3):e1003284. doi: 10.1371/journal.pgen.1003284 (PMC3609648; doi:10.1371/journal.pgen.1003284)
Supplement: Table S6 — Per-allele odds ratios (OR) and 95% confidence intervals (CI) for SNPs by environmental risk factors of breast cancer, estrogen receptor negative. (PDF) [file pgen.1003284.s006.pdf]

Table S6. Per-allele odds ratios (OR) and 95% confidence intervals (CI) for SNPs by environmental risk factors of breast cancer, estrogen receptor negative

SNP	Variable	Stratum	N (cases/ controls)	OR (95% CI)[1]	OR int[2]	P int[3]
rs1011970	Age at menarche (years)	<=11	437/ 2920	1.02 (0.84-1.24)		
rs1011970	Age at menarche (years)	12-13	1560/10797	1.12 (1.01-1.25)		
rs1011970	Age at menarche (years)	>=14	1394/10850	1.17 (1.05-1.30)		
rs1011970	Age at menarche (years/2)	combined	3391/24567		1.06	1.6E-01
rs1011970	Parous	no	617/ 3808	1.19 (1.01-1.40)		
rs1011970	Parous	yes	3418/24161	1.12 (1.05-1.21)		
rs1011970	Parous (yes/no)	combined	4035/27969		0.94	5.1E-01
rs1011970	Number of births (among parous)	1	879/ 5136	1.14 (0.99-1.31)		
rs1011970	Number of births (among parous)	2	1503/11012	1.08 (0.97-1.20)		
rs1011970	Number of births (among parous)	3	630/ 4819	1.27 (1.09-1.49)		
rs1011970	Number of births (among parous)	>=4	332/ 2321	1.00 (0.80-1.26)		
rs1011970	Number of births (among parous)	combined	3344/23288		1.01	8.2E-01
rs1011970	Age at first birth (among parous, years)	<20	362/ 2480	1.26 (1.03-1.53)		
rs1011970	Age at first birth (among parous, years)	20-24	1244/ 9285	1.05 (0.94-1.19)		
rs1011970	Age at first birth (among parous, years)	25-29	908/ 6903	1.20 (1.05-1.38)		
rs1011970	Age at first birth (among parous, years)	>=30	437/ 2996	1.00 (0.81-1.22)		
rs1011970	Age at first birth (among parous, years/5)	combined	2951/21664		0.94	1.6E-01
rs1011970	Ever breastfed (among parous, yes/no)	no	504/ 2303	1.10 (0.91-1.33)		
rs1011970	Ever breastfed (among parous, yes/no)	yes	1611/ 9425	1.14 (1.03-1.26)		
rs1011970	Ever breastfed (among parous, yes/no)	combined	2115/11728		1.04	7.5E-01
rs1011970	Usual adult BMI, age<54	<25	845/ 2725	1.11 (0.96-1.29)		
rs1011970	Usual adult BMI, age<54	25-<30	261/ 880	0.98 (0.75-1.29)		
rs1011970	Usual adult BMI, age<54	>=30	114/ 417	1.02 (0.67-1.53)		
rs1011970	Usual adult BMI (BMI/5), age<54	combined	1220/ 4022		0.93	3.0E-01
rs1011970	Usual adult BMI, age>=54	<25	731/ 5944	1.22 (1.06-1.41)		
rs1011970	Usual adult BMI, age>=54	25-<30	341/ 2490	1.06 (0.85-1.31)		
rs1011970	Usual adult BMI, age>=54	>=30	114/ 811	1.14 (0.81-1.62)		
rs1011970	Usual adult BMI (BMI/5), age>=54	combined	1186/ 9245		0.93	3.2E-01
rs1011970	Usual adult height (cm)	<160	811/ 4896	0.97 (0.84-1.12)		
rs1011970	Usual adult height (cm)	160-<165	987/ 5901	1.14 (1.00-1.30)		
rs1011970	Usual adult height (cm)	165-<170	875/ 4857	1.28 (1.12-1.47)		
rs1011970	Usual adult height (cm)	>=170	611/ 3206	1.12 (0.94-1.32)		
rs1011970	Usual adult height (cm/5)	combined	3284/18860		1.06	4.8E-02
rs1011970	Ever use of oral contraceptives	no	1154/ 7376	1.08 (0.96-1.22)		
rs1011970	Ever use of oral contraceptives	yes	1587/10197	1.17 (1.06-1.29)		
rs1011970	Ever use of oral contraceptives (yes/no)	combined	2741/17573		1.09	3.1E-01
rs1011970	Duration of oral contraceptive use (years)	0	1154/ 7376	1.08 (0.96-1.22)		
rs1011970	Duration of oral contraceptive use (years)	>0-<5	487/ 3384	1.19 (1.00-1.42)		
rs1011970	Duration of oral contraceptive use (years)	5-<10	405/ 2484	1.07 (0.87-1.31)		
rs1011970	Duration of oral contraceptive use (years)	>=10	666/ 4047	1.26 (1.08-1.47)		
rs1011970	Duration of oral contraceptive use (years/5)	combined	2712/17291		1.05	9.4E-02
rs1011970	Current use of combined estrogen/ progestagen MHT	never	547/ 4067	1.08 (0.91-1.28)		
rs1011970	Current use of combined estrogen/ progestagen MHT	EPCurrent	139/ 1186	1.41 (1.03-1.92)		
rs1011970	Current use of combined estrogen/ progestagen MHT (yes/no)	combined	990/ 8108		1.27	1.6E-01
rs1011970	Current use of estrogen only MHT	never	571/ 4138	1.10 (0.93-1.30)		
rs1011970	Current use of estrogen only MHT	ECurrent	85/ 916	0.88 (0.56-1.38)		
rs1011970	Current use of estrogen only MHT (yes/no)	combined	1033/ 8272		0.76	2.5E-01

SNP	Variable	Stratum	N (cases/ controls)	OR (95% CI)[1]	OR int[2]	P int[3]
rs1011970	Duration of combined estrogen/progestagen MHT among current users (years)	never	547/ 4067	1.08 (0.90-1.28)		
rs1011970	Duration of combined estrogen/progestagen MHT among current users (years)	>0-<5	36/ 314	0.95 (0.48-1.91)		
rs1011970	Duration of combined estrogen/progestagen MHT among current users (years)	5-<10	47/ 352	1.20 (0.67-2.14)		
rs1011970	Duration of combined estrogen/progestagen MHT among current users (years)	>=10	48/ 462	1.79 (1.09-2.94)		
rs1011970	Duration of combined estrogen/progestagen MHT among current users (years/5)	combined	981/ 8013		1.11	1.8E-01
rs1011970	Duration of estrogen only MHT among current users (years)	never	571/ 4138	1.10 (0.93-1.30)		
rs1011970	Duration of estrogen only MHT among current users (years)	>0-<5	27/ 279	0.58 (0.22-1.48)		
rs1011970	Duration of estrogen only MHT among current users (years)	5-<10	30/ 254	1.13 (0.51-2.47)		
rs1011970	Duration of estrogen only MHT among current users (years)	>=10	27/ 360	1.10 (0.54-2.25)		
rs1011970	Duration of estrogen only MHT among current users (years/5)	combined	1029/ 8197		0.97	7.4E-01
rs1011970	Mean lifetime intake of alcohol (g/day)	0	553/ 2803	1.07 (0.89-1.29)		
rs1011970	Mean lifetime intake of alcohol (g/day)	>0-<10	640/ 4654	1.19 (1.02-1.39)		
rs1011970	Mean lifetime intake of alcohol (g/day)	>=10-<20	114/ 1059	1.16 (0.81-1.67)		
rs1011970	Mean lifetime intake of alcohol (g/day)	>=20	100/ 741	1.24 (0.85-1.80)		
rs1011970	Mean lifetime intake of alcohol (10g/day)	combined	1407/ 9257		0.95	2.9E-01
rs1011970	Smoking (ever)	no	1704/ 9654	1.12 (1.01-1.24)		
rs1011970	Smoking (ever)	yes	1442/ 7798	1.12 (1.00-1.25)		
rs1011970	Smoking (ever/never)	combined	3146/17452		1.00	9.7E-01
rs1011970	Smoking (pack-years)	0	1579/ 8476	1.14 (1.03-1.27)		
rs1011970	Smoking (pack-years)	0-<10	598/ 3379	1.12 (0.94-1.32)		
rs1011970	Smoking (pack-years)	10-<20	304/ 1477	1.12 (0.89-1.40)		
rs1011970	Smoking (pack-years)	>=20	299/ 1849	1.10 (0.87-1.39)		
rs1011970	Smoking (pack-years/10)	combined	2780/15181		0.99	7.4E-01
rs1011970	Physical activity during year before reference date (h/week)	0	112/ 680	1.50 (1.04-2.17)		
rs1011970	Physical activity during year before reference date (h/week)	0-<3.5	330/ 2413	1.02 (0.82-1.28)		
rs1011970	Physical activity during year before reference date (h/week)	3.5-<7	239/ 1962	1.28 (1.01-1.61)		
rs1011970	Physical activity during year before reference date (h/week)	>=7	322/ 3343	1.29 (1.05-1.58)		
rs1011970	Physical activity during year before reference date (square root of h/week)	combined	1003/ 8398		1.02	7.2E-01
rs10771399[6]	Age at menarche (years)	<=11	468/ 2111	0.79 (0.62-1.01)		
rs10771399	Age at menarche (years)	12-13	1297/ 5902	0.91 (0.78-1.05)		
rs10771399	Age at menarche (years)	>=14	996/ 5310	0.79 (0.67-0.95)		
rs10771399	Age at menarche (years/2)	combined	2761/13323		0.96	5.4E-01
rs10771399	Parous	no	482/ 2187	0.69 (0.53-0.88)		
rs10771399	Parous	yes	2888/13944	0.84 (0.76-0.93)		
rs10771399	Parous (yes/no)	combined	3370/16131		1.23	1.4E-01
rs10771399	Number of births (among parous)	1	611/ 2515	0.99 (0.80-1.23)		
rs10771399	Number of births (among parous)	2	1296/ 5872	0.81 (0.69-0.94)		
rs10771399	Number of births (among parous)	3	615/ 3033	0.70 (0.56-0.88)		
rs10771399	Number of births (among parous)	>=4	315/ 1720	1.01 (0.77-1.32)		
rs10771399	Number of births (among parous)	combined	2837/13140		0.96	4.6E-01
rs10771399	Age at first birth (among parous, years)	<20	349/ 1162	1.06 (0.80-1.40)		
rs10771399	Age at first birth (among parous, years)	20-24	1004/ 5115	0.76 (0.64-0.90)		
rs10771399	Age at first birth (among parous, years)	25-29	723/ 3796	0.88 (0.72-1.07)		
rs10771399	Age at first birth (among parous, years)	>=30	352/ 1567	0.98 (0.74-1.30)		
rs10771399	Age at first birth (among parous, years/5)	combined	2428/11640		1.06	3.8E-01
rs10771399	Ever breastfed (among parous, yes/no)	no	275/ 1045	0.65 (0.46-0.91)		

SNP	Variable	Stratum	N (cases/ controls)	OR (95% CI)[1]	OR int[2]	P int[3]
rs10771399	Ever breastfed (among parous, yes/no)	yes	862/ 4980	0.85 (0.71-1.01)		
rs10771399	Ever breastfed (among parous, yes/no)	combined	1137/ 6025		1.31	1.7E-01
rs10771399	Usual adult BMI, age<54	<25	412/ 1232	0.70 (0.53-0.92)		
rs10771399	Usual adult BMI, age<54	25-<30	134/ 468	1.03 (0.63-1.67)		
rs10771399	Usual adult BMI, age<54	>=30	69/ 275	0.47 (0.23-0.96)		
rs10771399	Usual adult BMI (BMI/5), age<54	combined	615/ 1975		1.03	7.8E-01
rs10771399	Usual adult BMI, age>=54	<25	400/ 2851	0.76 (0.59-0.98)		
rs10771399	Usual adult BMI, age>=54	25-<30	202/ 1409	0.93 (0.66-1.30)		
rs10771399	Usual adult BMI, age>=54	>=30	72/ 558	0.88 (0.49-1.57)		
rs10771399	Usual adult BMI (BMI/5), age>=54	combined	674/ 4818		1.02	8.6E-01
rs10771399	Usual adult height (cm)	<160	448/ 2834	0.83 (0.65-1.06)		
rs10771399	Usual adult height (cm)	160-<165	497/ 3310	0.72 (0.57-0.91)		
rs10771399	Usual adult height (cm)	165-<170	487/ 2748	1.00 (0.80-1.24)		
rs10771399	Usual adult height (cm)	>=170	369/ 1816	0.76 (0.57-1.01)		
rs10771399	Usual adult height (cm/5)	combined	1801/10708		1.00	9.5E-01
rs10771399	Ever use of oral contraceptives	no	506/ 3904	0.86 (0.69-1.08)		
rs10771399	Ever use of oral contraceptives	yes	1065/ 6211	0.84 (0.72-0.98)		
rs10771399	Ever use of oral contraceptives (yes/no)	combined	1571/10115		0.98	8.6E-01
rs10771399	Duration of oral contraceptive use (years)	0	506/ 3904	0.86 (0.69-1.08)		
rs10771399	Duration of oral contraceptive use (years)	>0-<5	317/ 2097	0.83 (0.63-1.10)		
rs10771399	Duration of oral contraceptive use (years)	5-<10	286/ 1531	0.85 (0.63-1.14)		
rs10771399	Duration of oral contraceptive use (years)	>=10	439/ 2384	0.80 (0.63-1.02)		
rs10771399	Duration of oral contraceptive use (years/5)	combined	1548/ 9916		0.96	4.3E-01
rs10771399	Current use of combined estrogen/ progestagen MHT	never	249/ 1972	0.75 (0.56-1.02)		
rs10771399	Current use of combined estrogen/ progestagen MHT	EPCurrent	93/ 603	1.01 (0.63-1.63)		
rs10771399	Current use of combined estrogen/ progestagen MHT (yes/no)	combined	537/ 3929		1.22	4.6E-01
rs10771399	Current use of estrogen only MHT	never	272/ 2010	0.76 (0.57-1.03)		
rs10771399	Current use of estrogen only MHT	ECurrent	63/ 488	0.95 (0.52-1.72)		
rs10771399	Current use of estrogen only MHT (yes/no)	combined	578/ 4031		1.11	7.5E-01
rs10771399	Duration of combined estrogen/progestagen MHT among current users (years)	never	249/ 1972	0.75 (0.56-1.02)		
rs10771399	Duration of combined estrogen/progestagen MHT among current users (years)	>0-<5	19/ 168	1.20 (0.38-3.75)		
rs10771399	Duration of combined estrogen/progestagen MHT among current users (years)	5-<10	34/ 176	0.67 (0.29-1.51)		
rs10771399	Duration of combined estrogen/progestagen MHT among current users (years)	>=10	32/ 209	1.26 (0.58-2.75)		
rs10771399	Duration of combined estrogen/progestagen MHT among current users (years/5)	combined	528/ 3851		1.10	4.5E-01
rs10771399	Duration of estrogen only MHT among current users (years)	never	272/ 2010	0.76 (0.57-1.03)		
rs10771399	Duration of estrogen only MHT among current users (years)	>0-<5	18/ 150	0.65 (0.19-2.16)		
rs10771399	Duration of estrogen only MHT among current users (years)	5-<10	22/ 141	0.90 (0.33-2.41)		
rs10771399	Duration of estrogen only MHT among current users (years)	>=10	23/ 181	1.46 (0.54-3.92)		
rs10771399	Duration of estrogen only MHT among current users (years/5)	combined	576/ 3974		1.15	3.5E-01
rs10771399	Mean lifetime intake of alcohol (g/day)	0	124/ 1072	0.75 (0.47-1.20)		
rs10771399	Mean lifetime intake of alcohol (g/day)	>0-<10	314/ 1926	0.81 (0.61-1.07)		
rs10771399	Mean lifetime intake of alcohol (g/day)	>=10-<20	52/ 417	0.37 (0.14-0.93)		
rs10771399	Mean lifetime intake of alcohol (g/day)	>=20	51/ 313	0.46 (0.22-1.00)		
rs10771399	Mean lifetime intake of alcohol (10g/day)	combined	541/ 3728		0.78	5.1E-02
rs10771399	Smoking (ever)	no	935/ 5606	0.75 (0.63-0.90)		
rs10771399	Smoking (ever)	yes	777/ 4071	0.87 (0.73-1.05)		
rs10771399	Smoking (ever/never)	combined	1712/ 9677		1.16	2.5E-01

SNP	Variable	Stratum	N (cases/ controls)	OR (95% CI)[1]	OR int[2]	P int[3]
rs10771399	Smoking (pack-years)	0	810/ 4483	0.70 (0.57-0.85)		
rs10771399	Smoking (pack-years)	0-<10	281/ 1589	0.88 (0.65-1.19)		
rs10771399	Smoking (pack-years)	10-<20	142/ 689	1.05 (0.69-1.59)		
rs10771399	Smoking (pack-years)	>=20	173/ 891	0.59 (0.39-0.90)		
rs10771399	Smoking (pack-years/10)	combined	1406/ 7652		0.99	8.3E-01
rs10771399	Physical activity during year before reference date (h/week)	0	101/ 595	0.66 (0.37-1.17)		
rs10771399	Physical activity during year before reference date (h/week)	0-<3.5	203/ 1607	0.93 (0.67-1.29)		
rs10771399	Physical activity during year before reference date (h/week)	3.5-<7	168/ 1113	1.05 (0.75-1.47)		
rs10771399	Physical activity during year before reference date (h/week)	>=7	258/ 1290	0.67 (0.48-0.94)		
rs10771399	Physical activity during year before reference date (square root of h/week)	combined	730/ 4605		0.99	8.6E-01
rs10941679	Age at menarche (years)	<=11	676/ 3760	1.08 (0.94-1.23)		
rs10941679	Age at menarche (years)	12-13	2061/12457	1.03 (0.96-1.12)		
rs10941679	Age at menarche (years)	>=14	1723/12787	1.00 (0.92-1.09)		
rs10941679	Age at menarche (years/2)	combined	4460/29004		0.95	1.7E-01
rs10941679	Parous	no	741/ 4299	0.95 (0.83-1.09)		
rs10941679	Parous	yes	4287/28290	1.04 (0.98-1.09)		
rs10941679	Parous (yes/no)	combined	5028/32589		1.09	2.6E-01
rs10941679	Number of births (among parous)	1	1025/ 5763	1.03 (0.93-1.16)		
rs10941679	Number of births (among parous)	2	1957/13017	1.04 (0.96-1.12)		
rs10941679	Number of births (among parous)	3	819/ 5783	1.03 (0.91-1.16)		
rs10941679	Number of births (among parous)	>=4	417/ 2883	1.03 (0.87-1.23)		
rs10941679	Number of births (among parous)	combined	4218/27446		1.01	8.4E-01
rs10941679	Age at first birth (among parous, years)	<20	529/ 2834	1.07 (0.92-1.25)		
rs10941679	Age at first birth (among parous, years)	20-24	1651/11195	1.03 (0.95-1.13)		
rs10941679	Age at first birth (among parous, years)	25-29	1160/ 8275	1.08 (0.98-1.20)		
rs10941679	Age at first birth (among parous, years)	>=30	536/ 3551	0.92 (0.79-1.08)		
rs10941679	Age at first birth (among parous, years/5)	combined	3876/25855		0.97	2.7E-01
rs10941679	Ever breastfed (among parous, yes/no)	no	522/ 2886	1.04 (0.89-1.22)		
rs10941679	Ever breastfed (among parous, yes/no)	yes	1747/10347	1.03 (0.95-1.13)		
rs10941679	Ever breastfed (among parous, yes/no)	combined	2269/13233		0.99	9.6E-01
rs10941679	Usual adult BMI, age<54	<25	899/ 3124	1.08 (0.95-1.22)		
rs10941679	Usual adult BMI, age<54	25-<30	277/ 1055	0.86 (0.68-1.08)		
rs10941679	Usual adult BMI, age<54	>=30	115/ 521	1.16 (0.83-1.63)		
rs10941679	Usual adult BMI (BMI/5), age<54	combined	1291/ 4700		1.03	5.7E-01
rs10941679	Usual adult BMI, age>=54	<25	785/ 6461	1.05 (0.93-1.18)		
rs10941679	Usual adult BMI, age>=54	25-<30	355/ 2735	0.88 (0.73-1.07)		
rs10941679	Usual adult BMI, age>=54	>=30	114/ 942	1.31 (0.95-1.80)		
rs10941679	Usual adult BMI (BMI/5), age>=54	combined	1254/10138		1.06	3.3E-01
rs10941679	Usual adult height (cm)	<160	858/ 5328	0.97 (0.86-1.10)		
rs10941679	Usual adult height (cm)	160-<165	1039/ 6388	0.99 (0.88-1.10)		
rs10941679	Usual adult height (cm)	165-<170	907/ 5263	1.18 (1.05-1.33)		
rs10941679	Usual adult height (cm)	>=170	640/ 3480	1.04 (0.90-1.20)		
rs10941679	Usual adult height (cm/5)	combined	3444/20459		1.03	1.9E-01
rs10941679	Ever use of oral contraceptives	no	1265/ 8189	0.98 (0.89-1.08)		
rs10941679	Ever use of oral contraceptives	yes	1680/10716	1.07 (0.98-1.16)		
rs10941679	Ever use of oral contraceptives (yes/no)	combined	2945/18905		1.09	1.9E-01
rs10941679	Duration of oral contraceptive use (years)	0	1265/ 8189	0.98 (0.89-1.08)		
rs10941679	Duration of oral contraceptive use (years)	>0-<5	517/ 3540	1.26 (1.08-1.46)		
rs10941679	Duration of oral contraceptive use (years)	5-<10	409/ 2644	0.97 (0.81-1.16)		

SNP	Variable	Stratum	N (cases/ controls)	OR (95% CI)[1]	OR int[2]	P int[3]
rs10941679	Duration of oral contraceptive use (years)	>=10	722/ 4247	0.99 (0.87-1.13)		
rs10941679	Duration of oral contraceptive use (years/5)	combined	2913/18620		1.00	9.8E-01
rs10941679	Current use of combined estrogen/ progestagen MHT	never	582/ 4486	0.92 (0.79-1.06)		
rs10941679	Current use of combined estrogen/ progestagen MHT	EPCurrent	151/ 1365	1.21 (0.92-1.58)		
rs10941679	Current use of combined estrogen/ progestagen MHT (yes/no)	combined	1051/ 8973		1.23	1.6E-01
rs10941679	Current use of estrogen only MHT	never	606/ 4568	0.92 (0.80-1.06)		
rs10941679	Current use of estrogen only MHT	ECurrent	87/ 1055	1.01 (0.70-1.46)		
rs10941679	Current use of estrogen only MHT (yes/no)	combined	1094/ 9164		1.00	9.9E-01
rs10941679	Duration of combined estrogen/progestagen MHT among current users (years)	never	582/ 4486	0.92 (0.79-1.06)		
rs10941679	Duration of combined estrogen/progestagen MHT among current users (years)	>0-<5	39/ 373	0.72 (0.39-1.31)		
rs10941679	Duration of combined estrogen/progestagen MHT among current users (years)	5-<10	52/ 411	1.76 (1.13-2.74)		
rs10941679	Duration of combined estrogen/progestagen MHT among current users (years)	>=10	52/ 523	1.12 (0.71-1.79)		
rs10941679	Duration of combined estrogen/progestagen MHT among current users (years/5)	combined	1041/ 8878		1.10	1.5E-01
rs10941679	Duration of estrogen only MHT among current users (years)	never	606/ 4568	0.92 (0.80-1.06)		
rs10941679	Duration of estrogen only MHT among current users (years)	>0-<5	27/ 298	0.74 (0.37-1.46)		
rs10941679	Duration of estrogen only MHT among current users (years)	5-<10	29/ 286	1.06 (0.56-2.02)		
rs10941679	Duration of estrogen only MHT among current users (years)	>=10	30/ 432	1.18 (0.63-2.18)		
rs10941679	Duration of estrogen only MHT among current users (years/5)	combined	1089/ 9047		1.06	5.1E-01
rs10941679	Mean lifetime intake of alcohol (g/day)	0	628/ 3017	1.06 (0.92-1.22)		
rs10941679	Mean lifetime intake of alcohol (g/day)	>0-<10	689/ 4933	1.00 (0.88-1.15)		
rs10941679	Mean lifetime intake of alcohol (g/day)	>=10-<20	132/ 1128	0.94 (0.70-1.28)		
rs10941679	Mean lifetime intake of alcohol (g/day)	>=20	106/ 801	1.03 (0.74-1.42)		
rs10941679	Mean lifetime intake of alcohol (10g/day)	combined	1555/ 9879		0.99	8.6E-01
rs10941679	Smoking (ever)	no	1745/10276	1.04 (0.96-1.14)		
rs10941679	Smoking (ever)	yes	1508/ 8592	1.05 (0.96-1.15)		
rs10941679	Smoking (ever/never)	combined	3253/18868		1.01	9.2E-01
rs10941679	Smoking (pack-years)	0	1622/ 9154	1.05 (0.96-1.15)		
rs10941679	Smoking (pack-years)	0-<10	639/ 3682	1.09 (0.95-1.26)		
rs10941679	Smoking (pack-years)	10-<20	313/ 1652	1.04 (0.85-1.28)		
rs10941679	Smoking (pack-years)	>=20	329/ 2162	0.93 (0.76-1.13)		
rs10941679	Smoking (pack-years/10)	combined	2903/16650		0.95	1.0E-01
rs10941679	Physical activity during year before reference date (h/week)	0	112/ 1068	1.06 (0.75-1.50)		
rs10941679	Physical activity during year before reference date (h/week)	0-<3.5	331/ 2758	0.90 (0.75-1.09)		
rs10941679	Physical activity during year before reference date (h/week)	3.5-<7	241/ 2183	1.05 (0.85-1.31)		
rs10941679	Physical activity during year before reference date (h/week)	>=7	345/ 3684	1.05 (0.88-1.26)		
rs10941679	Physical activity during year before reference date (square root of h/week)	combined	1029/ 9693		1.05	2.3E-01
rs10995190	Age at menarche (years)	<=11	435/ 2927	1.09 (0.89-1.33)		
rs10995190	Age at menarche (years)	12-13	1563/10817	0.91 (0.81-1.01)		
rs10995190	Age at menarche (years)	>=14	1395/10884	0.85 (0.76-0.95)		
rs10995190	Age at menarche (years/2)	combined	3393/24628		0.92	8.5E-02
rs10995190	Parous	no	606/ 3814	0.93 (0.78-1.11)		
rs10995190	Parous	yes	3333/24215	0.91 (0.85-0.98)		
rs10995190	Parous (yes/no)	combined	3939/28029		0.98	8.6E-01
rs10995190	Number of births (among parous)	1	859/ 5150	0.89 (0.77-1.03)		
rs10995190	Number of births (among parous)	2	1466/11034	0.90 (0.81-1.01)		
rs10995190	Number of births (among parous)	3	617/ 4831	0.95 (0.80-1.13)		

SNP	Variable	Stratum	N (cases/ controls)	OR (95% CI)[1]	OR int[2]	P int[3]
rs10995190	Number of births (among parous)	>=4	320/ 2327	1.00 (0.79-1.27)		
rs10995190	Number of births (among parous)	combined	3262/23342		1.06	1.2E-01
rs10995190	Age at first birth (among parous, years)	<20	361/ 2483	0.76 (0.60-0.96)		
rs10995190	Age at first birth (among parous, years)	20-24	1244/ 9312	0.98 (0.87-1.10)		
rs10995190	Age at first birth (among parous, years)	25-29	908/ 6920	1.00 (0.87-1.15)		
rs10995190	Age at first birth (among parous, years)	>=30	440/ 3005	0.86 (0.70-1.05)		
rs10995190	Age at first birth (among parous, years/5)	combined	2953/21720		1.01	8.2E-01
rs10995190	Ever breastfed (among parous, yes/no)	no	502/ 2302	0.88 (0.72-1.07)		
rs10995190	Ever breastfed (among parous, yes/no)	yes	1611/ 9423	0.94 (0.84-1.04)		
rs10995190	Ever breastfed (among parous, yes/no)	combined	2113/11725		1.07	5.7E-01
rs10995190	Usual adult BMI, age<54	<25	841/ 2728	0.82 (0.70-0.96)		
rs10995190	Usual adult BMI, age<54	25-<30	261/ 881	0.97 (0.74-1.27)		
rs10995190	Usual adult BMI, age<54	>=30	114/ 417	1.01 (0.68-1.48)		
rs10995190	Usual adult BMI (BMI/5), age<54	combined	1216/ 4026		1.06	3.7E-01
rs10995190	Usual adult BMI, age>=54	<25	732/ 5945	0.97 (0.83-1.12)		
rs10995190	Usual adult BMI, age>=54	25-<30	340/ 2487	0.87 (0.69-1.09)		
rs10995190	Usual adult BMI, age>=54	>=30	114/ 811	0.84 (0.55-1.27)		
rs10995190	Usual adult BMI (BMI/5), age>=54	combined	1186/ 9243		0.99	9.4E-01
rs10995190	Usual adult height (cm)	<160	813/ 4897	0.98 (0.84-1.13)		
rs10995190	Usual adult height (cm)	160-<165	987/ 5900	0.98 (0.86-1.12)		
rs10995190	Usual adult height (cm)	165-<170	873/ 4855	0.85 (0.73-0.98)		
rs10995190	Usual adult height (cm)	>=170	610/ 3207	0.95 (0.79-1.13)		
rs10995190	Usual adult height (cm/5)	combined	3283/18859		0.99	6.3E-01
rs10995190	Ever use of oral contraceptives	no	1152/ 7379	0.84 (0.74-0.95)		
rs10995190	Ever use of oral contraceptives	yes	1588/10198	0.99 (0.89-1.10)		
rs10995190	Ever use of oral contraceptives (yes/no)	combined	2740/17577		1.17	5.3E-02
rs10995190	Duration of oral contraceptive use (years)	0	1152/ 7379	0.84 (0.74-0.95)		
rs10995190	Duration of oral contraceptive use (years)	>0-<5	487/ 3383	1.00 (0.83-1.21)		
rs10995190	Duration of oral contraceptive use (years)	5-<10	404/ 2482	0.99 (0.80-1.23)		
rs10995190	Duration of oral contraceptive use (years)	>=10	668/ 4051	0.98 (0.83-1.15)		
rs10995190	Duration of oral contraceptive use (years/5)	combined	2711/17295		1.04	2.0E-01
rs10995190	Current use of combined estrogen/ progestagen MHT	never	545/ 4070	0.95 (0.80-1.13)		
rs10995190	Current use of combined estrogen/ progestagen MHT	EPCurrent	141/ 1189	0.73 (0.51-1.04)		
rs10995190	Current use of combined estrogen/ progestagen MHT (yes/no)	combined	990/ 8113		0.79	2.2E-01
rs10995190	Current use of estrogen only MHT	never	569/ 4140	0.94 (0.79-1.11)		
rs10995190	Current use of estrogen only MHT	ECurrent	85/ 918	0.83 (0.53-1.31)		
rs10995190	Current use of estrogen only MHT (yes/no)	combined	1033/ 8275		0.91	6.9E-01
rs10995190	Duration of combined estrogen/progestagen MHT among current users (years)	never	545/ 4070	0.95 (0.80-1.13)		
rs10995190	Duration of combined estrogen/progestagen MHT among current users (years)	>0-<5	36/ 314	0.95 (0.49-1.85)		
rs10995190	Duration of combined estrogen/progestagen MHT among current users (years)	5-<10	48/ 353	0.67 (0.35-1.25)		
rs10995190	Duration of combined estrogen/progestagen MHT among current users (years)	>=10	49/ 464	0.78 (0.43-1.42)		
rs10995190	Duration of combined estrogen/progestagen MHT among current users (years/5)	combined	981/ 8018		0.94	4.6E-01
rs10995190	Duration of estrogen only MHT among current users (years)	never	569/ 4140	0.94 (0.79-1.11)		
rs10995190	Duration of estrogen only MHT among current users (years)	>0-<5	27/ 280	0.65 (0.28-1.52)		
rs10995190	Duration of estrogen only MHT among current users (years)	5-<10	30/ 255	1.19 (0.61-2.32)		
rs10995190	Duration of estrogen only MHT among current users (years)	>=10	27/ 360	0.64 (0.26-1.56)		
rs10995190	Duration of estrogen only MHT among current users (years/5)	combined	1029/ 8200		0.95	6.6E-01

SNP	Variable	Stratum	N (cases/ controls)	OR (95% CI)[1]	OR int[2]	P int[3]
rs10995190	Mean lifetime intake of alcohol (g/day)	0	551/ 2798	0.86 (0.72-1.02)		
rs10995190	Mean lifetime intake of alcohol (g/day)	>0-<10	638/ 4659	0.89 (0.76-1.05)		
rs10995190	Mean lifetime intake of alcohol (g/day)	>=10-<20	114/ 1059	1.09 (0.75-1.59)		
rs10995190	Mean lifetime intake of alcohol (g/day)	>=20	102/ 740	1.01 (0.67-1.53)		
rs10995190	Mean lifetime intake of alcohol (10g/day)	combined	1405/ 9256		1.06	1.5E-01
rs10995190	Smoking (ever)	no	1704/ 9654	0.92 (0.83-1.02)		
rs10995190	Smoking (ever)	yes	1441/ 7795	0.90 (0.81-1.01)		
rs10995190	Smoking (ever/never)	combined	3145/17449		0.98	8.0E-01
rs10995190	Smoking (pack-years)	0	1579/ 8475	0.93 (0.84-1.04)		
rs10995190	Smoking (pack-years)	0-<10	597/ 3374	0.91 (0.77-1.08)		
rs10995190	Smoking (pack-years)	10-<20	302/ 1472	0.89 (0.69-1.14)		
rs10995190	Smoking (pack-years)	>=20	300/ 1854	0.95 (0.74-1.21)		
rs10995190	Smoking (pack-years/10)	combined	2778/15175		1.01	8.4E-01
rs10995190	Physical activity during year before reference date (h/week)	0	112/ 679	0.95 (0.63-1.43)		
rs10995190	Physical activity during year before reference date (h/week)	0-<3.5	331/ 2417	0.80 (0.63-1.02)		
rs10995190	Physical activity during year before reference date (h/week)	3.5-<7	239/ 1969	0.84 (0.64-1.11)		
rs10995190	Physical activity during year before reference date (h/week)	>=7	323/ 3342	1.08 (0.87-1.34)		
rs10995190	Physical activity during year before reference date (square root of h/week)	combined	1005/ 8407		1.10	6.9E-02
rs11249433	Age at menarche (years)	<=11	663/ 3510	0.99 (0.87-1.12)		
rs11249433	Age at menarche (years)	12-13	1993/11480	1.08 (1.01-1.17)		
rs11249433	Age at menarche (years)	>=14	1614/11182	0.98 (0.91-1.06)		
rs11249433	Age at menarche (years/2)	combined	4270/26172		0.97	3.1E-01
rs11249433	Parous	no	720/ 3796	0.96 (0.85-1.08)		
rs11249433	Parous	yes	4203/25432	1.04 (0.99-1.10)		
rs11249433	Parous (yes/no)	combined	4923/29228		1.09	1.9E-01
rs11249433	Number of births (among parous)	1	987/ 4880	1.04 (0.94-1.15)		
rs11249433	Number of births (among parous)	2	1887/11511	1.00 (0.93-1.07)		
rs11249433	Number of births (among parous)	3	795/ 5187	1.14 (1.02-1.27)		
rs11249433	Number of births (among parous)	>=4	400/ 2605	1.00 (0.86-1.17)		
rs11249433	Number of births (among parous)	combined	4069/24183		1.04	1.3E-01
rs11249433	Age at first birth (among parous, years)	<20	498/ 2412	1.06 (0.91-1.23)		
rs11249433	Age at first birth (among parous, years)	20-24	1554/ 9784	1.06 (0.98-1.15)		
rs11249433	Age at first birth (among parous, years)	25-29	1101/ 7415	1.01 (0.92-1.11)		
rs11249433	Age at first birth (among parous, years)	>=30	540/ 3266	1.06 (0.92-1.21)		
rs11249433	Age at first birth (among parous, years/5)	combined	3693/22877		0.99	8.2E-01
rs11249433	Ever breastfed (among parous, yes/no)	no	486/ 2254	1.05 (0.90-1.21)		
rs11249433	Ever breastfed (among parous, yes/no)	yes	1625/ 7952	1.07 (0.98-1.15)		
rs11249433	Ever breastfed (among parous, yes/no)	combined	2111/10206		1.02	8.3E-01
rs11249433	Usual adult BMI, age<54	<25	885/ 2863	1.06 (0.95-1.18)		
rs11249433	Usual adult BMI, age<54	25-<30	274/ 993	0.96 (0.78-1.18)		
rs11249433	Usual adult BMI, age<54	>=30	116/ 501	0.85 (0.62-1.15)		
rs11249433	Usual adult BMI (BMI/5), age<54	combined	1275/ 4357		0.95	3.8E-01
rs11249433	Usual adult BMI, age>=54	<25	654/ 4064	0.99 (0.88-1.12)		
rs11249433	Usual adult BMI, age>=54	25-<30	321/ 2098	1.20 (1.01-1.43)		
rs11249433	Usual adult BMI, age>=54	>=30	111/ 840	0.86 (0.64-1.17)		
rs11249433	Usual adult BMI (BMI/5), age>=54	combined	1086/ 7002		0.99	9.1E-01
rs11249433	Usual adult height (cm)	<160	818/ 4519	1.02 (0.92-1.15)		
rs11249433	Usual adult height (cm)	160-<165	944/ 5243	1.01 (0.91-1.12)		
rs11249433	Usual adult height (cm)	165-<170	830/ 4167	1.06 (0.95-1.19)		

SNP	Variable	Stratum	N (cases/ controls)	OR (95% CI)[1]	OR int[2]	P int[3]
rs11249433	Usual adult height (cm)	>=170	584/ 2739	1.06 (0.93-1.21)		
rs11249433	Usual adult height (cm/5)	combined	3176/16668		1.02	5.1E-01
rs11249433	Ever use of oral contraceptives	no	1151/ 6821	1.04 (0.94-1.13)		
rs11249433	Ever use of oral contraceptives	yes	1528/ 8286	1.00 (0.92-1.09)		
rs11249433	Ever use of oral contraceptives (yes/no)	combined	2679/15107		0.97	6.0E-01
rs11249433	Duration of oral contraceptive use (years)	0	1151/ 6821	1.04 (0.94-1.14)		
rs11249433	Duration of oral contraceptive use (years)	>0-<5	472/ 2821	1.02 (0.88-1.17)		
rs11249433	Duration of oral contraceptive use (years)	5-<10	383/ 2053	0.92 (0.78-1.08)		
rs11249433	Duration of oral contraceptive use (years)	>=10	644/ 3145	1.05 (0.92-1.19)		
rs11249433	Duration of oral contraceptive use (years/5)	combined	2650/14840		1.00	9.9E-01
rs11249433	Current use of combined estrogen/ progestagen MHT	never	509/ 3247	1.06 (0.92-1.22)		
rs11249433	Current use of combined estrogen/ progestagen MHT	EPCurrent	118/ 769	1.21 (0.91-1.60)		
rs11249433	Current use of combined estrogen/ progestagen MHT (yes/no)	combined	881/ 5801		1.20	2.5E-01
rs11249433	Current use of estrogen only MHT	never	533/ 3329	1.05 (0.92-1.21)		
rs11249433	Current use of estrogen only MHT	ECurrent	75/ 648	1.10 (0.79-1.53)		
rs11249433	Current use of estrogen only MHT (yes/no)	combined	926/ 5988		1.06	7.3E-01
rs11249433	Duration of combined estrogen/progestagen MHT among current users (years)	never	509/ 3247	1.06 (0.92-1.21)		
rs11249433	Duration of combined estrogen/progestagen MHT among current users (years)	>0-<5	32/ 245	1.23 (0.73-2.08)		
rs11249433	Duration of combined estrogen/progestagen MHT among current users (years)	5-<10	44/ 230	1.13 (0.69-1.85)		
rs11249433	Duration of combined estrogen/progestagen MHT among current users (years)	>=10	34/ 243	1.39 (0.83-2.33)		
rs11249433	Duration of combined estrogen/progestagen MHT among current users (years/5)	combined	871/ 5717		1.08	3.1E-01
rs11249433	Duration of estrogen only MHT among current users (years)	never	533/ 3329	1.05 (0.92-1.20)		
rs11249433	Duration of estrogen only MHT among current users (years)	>0-<5	24/ 180	1.51 (0.82-2.78)		
rs11249433	Duration of estrogen only MHT among current users (years)	5-<10	22/ 175	0.93 (0.51-1.69)		
rs11249433	Duration of estrogen only MHT among current users (years)	>=10	28/ 259	1.06 (0.62-1.82)		
rs11249433	Duration of estrogen only MHT among current users (years/5)	combined	921/ 5875		1.00	1.0E+00
rs11249433	Mean lifetime intake of alcohol (g/day)	0	575/ 2523	1.04 (0.91-1.18)		
rs11249433	Mean lifetime intake of alcohol (g/day)	>0-<10	583/ 2858	1.08 (0.95-1.23)		
rs11249433	Mean lifetime intake of alcohol (g/day)	>=10-<20	115/ 590	1.09 (0.82-1.47)		
rs11249433	Mean lifetime intake of alcohol (g/day)	>=20	94/ 393	1.27 (0.92-1.76)		
rs11249433	Mean lifetime intake of alcohol (10g/day)	combined	1367/ 6364		1.04	2.9E-01
rs11249433	Smoking (ever)	no	1625/ 8382	1.05 (0.97-1.13)		
rs11249433	Smoking (ever)	yes	1406/ 6896	1.03 (0.95-1.13)		
rs11249433	Smoking (ever/never)	combined	3031/15278		0.99	8.5E-01
rs11249433	Smoking (pack-years)	0	1504/ 7256	1.06 (0.97-1.15)		
rs11249433	Smoking (pack-years)	0-<10	592/ 2884	1.04 (0.91-1.19)		
rs11249433	Smoking (pack-years)	10-<20	292/ 1300	0.95 (0.78-1.14)		
rs11249433	Smoking (pack-years)	>=20	302/ 1633	1.01 (0.84-1.22)		
rs11249433	Smoking (pack-years/10)	combined	2690/13073		0.99	6.7E-01
rs11249433	Physical activity during year before reference date (h/week)	0	107/ 1004	1.05 (0.78-1.42)		
rs11249433	Physical activity during year before reference date (h/week)	0-<3.5	309/ 2387	0.97 (0.82-1.16)		
rs11249433	Physical activity during year before reference date (h/week)	3.5-<7	187/ 1463	1.11 (0.89-1.38)		
rs11249433	Physical activity during year before reference date (h/week)	>=7	254/ 1341	0.92 (0.76-1.12)		
rs11249433	Physical activity during year before reference date (square root of h/week)	combined	857/ 6195		0.97	5.5E-01
rs12662670	Age at menarche (years)	<=11	376/ 1719	1.16 (0.87-1.55)		
rs12662670	Age at menarche (years)	12-13	979/ 4831	1.13 (0.94-1.35)		

SNP	Variable	Stratum	N (cases/ controls)	OR (95% CI)[1]	OR int[2]	P int[3]
rs12662670	Age at menarche (years)	>=14	737/ 4336	1.31 (1.07-1.60)		
rs12662670	Age at menarche (years/2)	combined	2092/10886		1.08	3.1E-01
rs12662670	Parous	no	370/ 1880	1.64 (1.24-2.19)		
rs12662670	Parous	yes	2271/11781	1.16 (1.03-1.30)		
rs12662670	Parous (yes/no)	combined	2641/13661		0.71	2.7E-02
rs12662670	Number of births (among parous)	1	489/ 2151	1.15 (0.90-1.48)		
rs12662670	Number of births (among parous)	2	990/ 5068	1.21 (1.02-1.44)		
rs12662670	Number of births (among parous)	3	490/ 2461	1.07 (0.83-1.38)		
rs12662670	Number of births (among parous)	>=4	257/ 1296	1.09 (0.78-1.53)		
rs12662670	Number of births (among parous)	combined	2226/10976		0.97	5.3E-01
rs12662670	Age at first birth (among parous, years)	<20	288/ 948	1.13 (0.80-1.58)		
rs12662670	Age at first birth (among parous, years)	20-24	777/ 4156	1.18 (0.96-1.45)		
rs12662670	Age at first birth (among parous, years)	25-29	514/ 3080	0.85 (0.65-1.11)		
rs12662670	Age at first birth (among parous, years)	>=30	238/ 1294	1.48 (1.05-2.08)		
rs12662670	Age at first birth (among parous, years/5)	combined	1817/ 9478		1.01	9.0E-01
rs12662670	Ever breastfed (among parous, yes/no)	no	199/ 598	1.31 (0.87-1.96)		
rs12662670	Ever breastfed (among parous, yes/no)	yes	601/ 3418	1.13 (0.90-1.43)		
rs12662670	Ever breastfed (among parous, yes/no)	combined	800/ 4016		0.86	5.4E-01
rs12662670	Usual adult BMI, age<54	<25	195/ 539	1.13 (0.74-1.74)		
rs12662670	Usual adult BMI, age<54	25-<30	67/ 252	1.73 (0.80-3.74)		
rs12662670	Usual adult BMI, age<54	>=30	36/ 156	0.49 (0.09-2.57)		
rs12662670	Usual adult BMI (BMI/5), age<54	combined	298/ 947		0.91	6.9E-01
rs12662670	Usual adult BMI, age>=54	<25	355/ 2269	1.43 (1.07-1.90)		
rs12662670	Usual adult BMI, age>=54	25-<30	181/ 1153	1.07 (0.70-1.63)		
rs12662670	Usual adult BMI, age>=54	>=30	66/ 452	1.37 (0.65-2.88)		
rs12662670	Usual adult BMI (BMI/5), age>=54	combined	602/ 3874		0.90	4.6E-01
rs12662670	Usual adult height (cm)	<160	329/ 1999	1.33 (0.99-1.79)		
rs12662670	Usual adult height (cm)	160-<165	395/ 2530	1.20 (0.90-1.61)		
rs12662670	Usual adult height (cm)	165-<170	368/ 2244	1.14 (0.85-1.54)		
rs12662670	Usual adult height (cm)	>=170	279/ 1525	1.39 (1.02-1.91)		
rs12662670	Usual adult height (cm/5)	combined	1371/ 8298		0.98	7.3E-01
rs12662670	Ever use of oral contraceptives	no	430/ 2907	1.18 (0.90-1.55)		
rs12662670	Ever use of oral contraceptives	yes	708/ 4747	1.26 (1.03-1.55)		
rs12662670	Ever use of oral contraceptives (yes/no)	combined	1138/ 7654		1.07	7.0E-01
rs12662670	Duration of oral contraceptive use (years)	0	430/ 2907	1.18 (0.91-1.55)		
rs12662670	Duration of oral contraceptive use (years)	>0-<5	228/ 1658	1.21 (0.84-1.75)		
rs12662670	Duration of oral contraceptive use (years)	5-<10	165/ 1138	1.17 (0.77-1.79)		
rs12662670	Duration of oral contraceptive use (years)	>=10	294/ 1778	1.38 (1.01-1.87)		
rs12662670	Duration of oral contraceptive use (years/5)	combined	1117/ 7481		1.01	8.4E-01
rs12662670	Current use of combined estrogen/ progestagen MHT	never	211/ 1476	1.61 (1.12-2.29)		
rs12662670	Current use of combined estrogen/ progestagen MHT	EPCurrent	84/ 509	0.98 (0.54-1.78)		
rs12662670	Current use of combined estrogen/ progestagen MHT (yes/no)	combined	468/ 3069		0.71	3.2E-01
rs12662670	Current use of estrogen only MHT	never	234/ 1514	1.55 (1.09-2.20)		
rs12662670	Current use of estrogen only MHT	ECurrent	58/ 455	1.04 (0.51-2.13)		
rs12662670	Current use of estrogen only MHT (yes/no)	combined	509/ 3171		0.77	5.0E-01
rs12662670	Duration of combined estrogen/progestagen MHT among current users (years)	never	211/ 1476	1.61 (1.12-2.29)		
rs12662670	Duration of combined estrogen/progestagen MHT among current users (years)	>0-<5	18/ 153	1.04 (0.30-3.66)		
rs12662670	Duration of combined estrogen/progestagen MHT among current users (years)	5-<10	33/ 165	0.79 (0.28-2.17)		

SNP	Variable	Stratum	N (cases/ controls)	OR (95% CI)[1]	OR int[2]	P int[3]
rs12662670	Duration of combined estrogen/progestagen MHT among current users (years)	>=10	32/ 187	1.14 (0.45-2.92)		
rs12662670	Duration of combined estrogen/progestagen MHT among current users (years/5)	combined	466/ 3064		0.87	4.0E-01
rs12662670	Duration of estrogen only MHT among current users (years)	never	234/ 1514	1.55 (1.09-2.20)		
rs12662670	Duration of estrogen only MHT among current users (years)	>0-<5	17/ 143	1.39 (0.49-3.92)		
rs12662670	Duration of estrogen only MHT among current users (years)	5-<10	21/ 138	0.98 (0.28-3.46)		
rs12662670	Duration of estrogen only MHT among current users (years)	>=10	20/ 172	0.67 (0.15-3.11)		
rs12662670	Duration of estrogen only MHT among current users (years/5)	combined	507/ 3158		0.84	4.0E-01
rs12662670	Mean lifetime intake of alcohol (g/day)	0	67/ 268	2.13 (1.18-3.85)		
rs12662670	Mean lifetime intake of alcohol (g/day)	>0-<10	210/ 1057	0.79 (0.51-1.21)		
rs12662670	Mean lifetime intake of alcohol (g/day)	>=10-<20	36/ 236	2.34 (1.03-5.32)		
rs12662670	Mean lifetime intake of alcohol (g/day)	>=20	39/ 198	1.42 (0.59-3.43)		
rs12662670	Mean lifetime intake of alcohol (10g/day)	combined	352/ 1759		1.07	4.1E-01
rs12662670	Smoking (ever)	no	701/ 4219	1.15 (0.93-1.43)		
rs12662670	Smoking (ever)	yes	577/ 2996	1.45 (1.15-1.83)		
rs12662670	Smoking (ever/never)	combined	1278/ 7215		1.26	1.5E-01
rs12662670	Smoking (pack-years)	0	576/ 3096	1.24 (0.98-1.57)		
rs12662670	Smoking (pack-years)	0-<10	178/ 1035	1.20 (0.76-1.89)		
rs12662670	Smoking (pack-years)	10-<20	97/ 440	2.04 (1.19-3.49)		
rs12662670	Smoking (pack-years)	>=20	124/ 640	1.54 (0.98-2.43)		
rs12662670	Smoking (pack-years/10)	combined	975/ 5211		1.09	1.7E-01
rs12662670	Physical activity during year before reference date (h/week)	0	11/ 32	5.65 (1.15-27.62)		
rs12662670	Physical activity during year before reference date (h/week)	0-<3.5	174/ 1320	1.11 (0.72-1.71)		
rs12662670	Physical activity during year before reference date (h/week)	3.5-<7	156/ 991	1.45 (0.95-2.20)		
rs12662670	Physical activity during year before reference date (h/week)	>=7	243/ 1148	1.20 (0.84-1.72)		
rs12662670	Physical activity during year before reference date (square root of h/week)	combined	584/ 3491		0.88	1.9E-01
rs1292011	Age at menarche (years)	<=11	409/ 1784	0.94 (0.80-1.10)		
rs1292011	Age at menarche (years)	12-13	1112/ 4745	0.94 (0.85-1.04)		
rs1292011	Age at menarche (years)	>=14	792/ 3975	1.14 (1.01-1.29)		
rs1292011	Age at menarche (years/2)	combined	2313/10504		1.06	2.3E-01
rs1292011	Parous	no	415/ 1737	1.08 (0.91-1.28)		
rs1292011	Parous	yes	2438/10569	0.98 (0.92-1.05)		
rs1292011	Parous (yes/no)	combined	2853/12306		0.91	3.3E-01
rs1292011	Number of births (among parous)	1	493/ 1987	0.96 (0.82-1.12)		
rs1292011	Number of births (among parous)	2	1117/ 4688	0.98 (0.89-1.09)		
rs1292011	Number of births (among parous)	3	543/ 2428	1.00 (0.87-1.15)		
rs1292011	Number of births (among parous)	>=4	275/ 1437	1.00 (0.82-1.21)		
rs1292011	Number of births (among parous)	combined	2428/10540		1.03	3.3E-01
rs1292011	Age at first birth (among parous, years)	<20	307/ 868	1.18 (0.95-1.45)		
rs1292011	Age at first birth (among parous, years)	20-24	830/ 3984	0.98 (0.87-1.11)		
rs1292011	Age at first birth (among parous, years)	25-29	596/ 2977	0.91 (0.79-1.04)		
rs1292011	Age at first birth (among parous, years)	>=30	290/ 1219	0.90 (0.73-1.10)		
rs1292011	Age at first birth (among parous, years/5)	combined	2023/ 9048		0.93	1.2E-01
rs1292011	Ever breastfed (among parous, yes/no)	no	221/ 847	0.83 (0.66-1.04)		
rs1292011	Ever breastfed (among parous, yes/no)	yes	583/ 2851	1.02 (0.89-1.16)		
rs1292011	Ever breastfed (among parous, yes/no)	combined	804/ 3698		1.23	1.2E-01
rs1292011	Usual adult BMI, age<54	<25	325/ 884	1.06 (0.88-1.29)		
rs1292011	Usual adult BMI, age<54	25-<30	107/ 290	1.03 (0.72-1.46)		
rs1292011	Usual adult BMI, age<54	>=30	43/ 157	0.75 (0.46-1.23)		

SNP	Variable	Stratum	N (cases/ controls)	OR (95% CI)[1]	OR int[2]	P int[3]
rs1292011	Usual adult BMI (BMI/5), age<54	combined	475/ 1331		0.92	3.4E-01
rs1292011	Usual adult BMI, age>=54	<25	288/ 1702	1.06 (0.88-1.27)		
rs1292011	Usual adult BMI, age>=54	25-<30	112/ 627	0.75 (0.55-1.03)		
rs1292011	Usual adult BMI, age>=54	>=30	37/ 215	0.72 (0.43-1.19)		
rs1292011	Usual adult BMI (BMI/5), age>=54	combined	437/ 2544		0.86	1.0E-01
rs1292011	Usual adult height (cm)	<160	316/ 1850	1.07 (0.90-1.28)		
rs1292011	Usual adult height (cm)	160-<165	347/ 2101	0.95 (0.81-1.13)		
rs1292011	Usual adult height (cm)	165-<170	364/ 1756	0.95 (0.80-1.13)		
rs1292011	Usual adult height (cm)	>=170	261/ 1208	0.99 (0.81-1.22)		
rs1292011	Usual adult height (cm/5)	combined	1288/ 6915		0.98	6.7E-01
rs1292011	Ever use of oral contraceptives	no	281/ 2240	0.82 (0.68-0.99)		
rs1292011	Ever use of oral contraceptives	yes	800/ 4089	1.08 (0.96-1.20)		
rs1292011	Ever use of oral contraceptives (yes/no)	combined	1081/ 6329		1.31	1.5E-02
rs1292011	Duration of oral contraceptive use (years)	0	281/ 2240	0.82 (0.68-0.99)		
rs1292011	Duration of oral contraceptive use (years)	>0-<5	219/ 1335	1.00 (0.81-1.23)		
rs1292011	Duration of oral contraceptive use (years)	5-<10	229/ 1043	1.03 (0.84-1.27)		
rs1292011	Duration of oral contraceptive use (years)	>=10	343/ 1595	1.12 (0.95-1.33)		
rs1292011	Duration of oral contraceptive use (years/5)	combined	1072/ 6213		1.05	1.4E-01
rs1292011	Current use of combined estrogen/ progestagen MHT	never	154/ 1079	0.88 (0.68-1.14)		
rs1292011	Current use of combined estrogen/ progestagen MHT	EPCurrent	72/ 381	1.16 (0.82-1.65)		
rs1292011	Current use of combined estrogen/ progestagen MHT (yes/no)	combined	344/ 2267		1.20	3.7E-01
rs1292011	Current use of estrogen only MHT	never	154/ 1079	0.88 (0.68-1.14)		
rs1292011	Current use of estrogen only MHT	ECurrent	36/ 221	0.87 (0.52-1.46)		
rs1292011	Current use of estrogen only MHT (yes/no)	combined	344/ 2270		0.85	5.6E-01
rs1292011	Duration of combined estrogen/progestagen MHT among current users (years)	never	154/ 1079	0.88 (0.68-1.14)		
rs1292011	Duration of combined estrogen/progestagen MHT among current users (years)	>0-<5	13/ 71	0.65 (0.26-1.64)		
rs1292011	Duration of combined estrogen/progestagen MHT among current users (years)	5-<10	22/ 106	1.94 (1.02-3.69)		
rs1292011	Duration of combined estrogen/progestagen MHT among current users (years)	>=10	30/ 157	1.13 (0.65-1.97)		
rs1292011	Duration of combined estrogen/progestagen MHT among current users (years/5)	combined	337/ 2192		1.04	6.4E-01
rs1292011	Duration of estrogen only MHT among current users (years)	never	154/ 1079	0.88 (0.68-1.14)		
rs1292011	Duration of estrogen only MHT among current users (years)	>0-<5	13/ 46	1.30 (0.51-3.32)		
rs1292011	Duration of estrogen only MHT among current users (years)	5-<10	9/ 63	0.67 (0.23-1.97)		
rs1292011	Duration of estrogen only MHT among current users (years)	>=10	14/ 96	0.85 (0.39-1.87)		
rs1292011	Duration of estrogen only MHT among current users (years/5)	combined	344/ 2223		0.94	6.1E-01
rs1292011	Mean lifetime intake of alcohol (g/day)	0	118/ 1055	0.94 (0.70-1.25)		
rs1292011	Mean lifetime intake of alcohol (g/day)	>0-<10	301/ 1848	1.01 (0.85-1.20)		
rs1292011	Mean lifetime intake of alcohol (g/day)	>=10-<20	52/ 392	1.19 (0.79-1.78)		
rs1292011	Mean lifetime intake of alcohol (g/day)	>=20	50/ 297	0.85 (0.54-1.32)		
rs1292011	Mean lifetime intake of alcohol (10g/day)	combined	521/ 3592		1.10	8.5E-02
rs1292011	Smoking (ever)	no	706/ 3889	1.01 (0.90-1.14)		
rs1292011	Smoking (ever)	yes	595/ 2777	0.98 (0.86-1.12)		
rs1292011	Smoking (ever/never)	combined	1301/ 6666		0.98	7.9E-01
rs1292011	Smoking (pack-years)	0	656/ 3558	0.99 (0.87-1.12)		
rs1292011	Smoking (pack-years)	0-<10	241/ 1269	0.93 (0.75-1.15)		
rs1292011	Smoking (pack-years)	10-<20	121/ 547	0.95 (0.71-1.27)		
rs1292011	Smoking (pack-years)	>=20	139/ 662	1.09 (0.83-1.42)		
rs1292011	Smoking (pack-years/10)	combined	1157/ 6036		1.01	8.5E-01

SNP	Variable	Stratum	N (cases/ controls)	OR (95% CI)[1]	OR int[2]	P int[3]
rs1292011	Physical activity during year before reference date (h/week)	0	100/ 593	0.93 (0.69-1.26)		
rs1292011	Physical activity during year before reference date (h/week)	0-<3.5	58/ 469	1.20 (0.80-1.79)		
rs1292011	Physical activity during year before reference date (h/week)	3.5-<7	74/ 467	1.12 (0.80-1.58)		
rs1292011	Physical activity during year before reference date (h/week)	>=7	245/ 1182	0.89 (0.73-1.09)		
rs1292011	Physical activity during year before reference date (square root of h/week)	combined	477/ 2711		0.96	3.5E-01
rs13281615	Age at menarche (years)	<=11	604/ 3094	1.09 (0.96-1.24)		
rs13281615	Age at menarche (years)	12-13	1823/ 9457	1.13 (1.04-1.21)		
rs13281615	Age at menarche (years)	>=14	1494/ 9023	1.02 (0.94-1.10)		
rs13281615	Age at menarche (years/2)	combined	3921/21574		0.98	4.6E-01
rs13281615	Parous	no	676/ 3254	1.18 (1.04-1.34)		
rs13281615	Parous	yes	3877/21325	1.06 (1.01-1.12)		
rs13281615	Parous (yes/no)	combined	4553/24579		0.90	1.2E-01
rs13281615	Number of births (among parous)	1	912/ 4083	1.11 (1.00-1.23)		
rs13281615	Number of births (among parous)	2	1757/ 9535	1.05 (0.98-1.13)		
rs13281615	Number of births (among parous)	3	746/ 4480	1.04 (0.92-1.16)		
rs13281615	Number of births (among parous)	>=4	391/ 2390	1.09 (0.93-1.28)		
rs13281615	Number of births (among parous)	combined	3806/20488		0.99	7.0E-01
rs13281615	Age at first birth (among parous, years)	<20	460/ 2024	1.09 (0.93-1.26)		
rs13281615	Age at first birth (among parous, years)	20-24	1455/ 8350	1.07 (0.99-1.16)		
rs13281615	Age at first birth (among parous, years)	25-29	1012/ 6004	1.04 (0.94-1.15)		
rs13281615	Age at first birth (among parous, years)	>=30	459/ 2504	1.08 (0.94-1.25)		
rs13281615	Age at first birth (among parous, years/5)	combined	3386/18882		0.98	5.9E-01
rs13281615	Ever breastfed (among parous, yes/no)	no	420/ 2134	1.05 (0.90-1.23)		
rs13281615	Ever breastfed (among parous, yes/no)	yes	1470/ 7613	1.08 (1.00-1.18)		
rs13281615	Ever breastfed (among parous, yes/no)	combined	1890/ 9747		1.03	7.3E-01
rs13281615	Usual adult BMI, age<54	<25	779/ 2779	1.07 (0.95-1.20)		
rs13281615	Usual adult BMI, age<54	25-<30	243/ 970	1.01 (0.82-1.26)		
rs13281615	Usual adult BMI, age<54	>=30	91/ 465	1.73 (1.24-2.42)		
rs13281615	Usual adult BMI (BMI/5), age<54	combined	1113/ 4214		1.08	1.8E-01
rs13281615	Usual adult BMI, age>=54	<25	590/ 3866	1.02 (0.90-1.16)		
rs13281615	Usual adult BMI, age>=54	25-<30	293/ 1968	1.13 (0.94-1.36)		
rs13281615	Usual adult BMI, age>=54	>=30	95/ 781	1.51 (1.11-2.06)		
rs13281615	Usual adult BMI (BMI/5), age>=54	combined	978/ 6615		1.12	5.3E-02
rs13281615	Usual adult height (cm)	<160	777/ 4440	1.04 (0.93-1.16)		
rs13281615	Usual adult height (cm)	160-<165	891/ 5068	1.14 (1.03-1.27)		
rs13281615	Usual adult height (cm)	165-<170	772/ 3990	1.04 (0.92-1.16)		
rs13281615	Usual adult height (cm)	>=170	537/ 2623	1.19 (1.03-1.37)		
rs13281615	Usual adult height (cm/5)	combined	2977/16121		1.01	6.6E-01
rs13281615	Ever use of oral contraceptives	no	1144/ 6696	1.06 (0.97-1.16)		
rs13281615	Ever use of oral contraceptives	yes	1337/ 7978	1.09 (1.00-1.19)		
rs13281615	Ever use of oral contraceptives (yes/no)	combined	2481/14674		1.03	6.6E-01
rs13281615	Duration of oral contraceptive use (years)	0	1144/ 6696	1.06 (0.97-1.16)		
rs13281615	Duration of oral contraceptive use (years)	>0-<5	412/ 2738	0.94 (0.80-1.09)		
rs13281615	Duration of oral contraceptive use (years)	5-<10	337/ 1992	1.29 (1.09-1.53)		
rs13281615	Duration of oral contraceptive use (years)	>=10	558/ 2991	1.11 (0.97-1.26)		
rs13281615	Duration of oral contraceptive use (years/5)	combined	2451/14417		1.01	5.9E-01
rs13281615	Current use of combined estrogen/ progestagen MHT	never	474/ 3116	1.13 (0.98-1.30)		
rs13281615	Current use of combined estrogen/ progestagen MHT	EPCurrent	104/ 729	1.01 (0.74-1.36)		
rs13281615	Current use of combined estrogen/ progestagen MHT (yes/no)	combined	803/ 5486		0.95	7.5E-01

SNP	Variable	Stratum	N (cases/ controls)	OR (95% CI)[1]	OR int[2]	P int[3]
rs13281615	Current use of estrogen only MHT	never	498/ 3187	1.12 (0.98-1.29)		
rs13281615	Current use of estrogen only MHT	ECurrent	69/ 601	0.93 (0.64-1.35)		
rs13281615	Current use of estrogen only MHT (yes/no)	combined	847/ 5640		0.86	4.3E-01
rs13281615	Duration of combined estrogen/progestagen MHT among current users (years)	never	474/ 3116	1.13 (0.99-1.30)		
rs13281615	Duration of combined estrogen/progestagen MHT among current users (years)	>0-<5	29/ 238	1.08 (0.61-1.91)		
rs13281615	Duration of combined estrogen/progestagen MHT among current users (years)	5-<10	38/ 212	1.03 (0.59-1.81)		
rs13281615	Duration of combined estrogen/progestagen MHT among current users (years)	>=10	30/ 229	0.91 (0.54-1.55)		
rs13281615	Duration of combined estrogen/progestagen MHT among current users (years/5)	combined	794/ 5404		0.97	7.0E-01
rs13281615	Duration of estrogen only MHT among current users (years)	never	498/ 3187	1.12 (0.98-1.29)		
rs13281615	Duration of estrogen only MHT among current users (years)	>0-<5	24/ 173	0.83 (0.42-1.63)		
rs13281615	Duration of estrogen only MHT among current users (years)	5-<10	21/ 157	0.95 (0.49-1.84)		
rs13281615	Duration of estrogen only MHT among current users (years)	>=10	23/ 238	0.99 (0.54-1.83)		
rs13281615	Duration of estrogen only MHT among current users (years/5)	combined	843/ 5535		0.95	5.5E-01
rs13281615	Mean lifetime intake of alcohol (g/day)	0	583/ 2524	1.16 (1.01-1.32)		
rs13281615	Mean lifetime intake of alcohol (g/day)	>0-<10	572/ 2810	1.07 (0.94-1.22)		
rs13281615	Mean lifetime intake of alcohol (g/day)	>=10-<20	109/ 580	0.95 (0.71-1.28)		
rs13281615	Mean lifetime intake of alcohol (g/day)	>=20	94/ 378	0.99 (0.72-1.35)		
rs13281615	Mean lifetime intake of alcohol (10g/day)	combined	1358/ 6292		0.96	2.8E-01
rs13281615	Smoking (ever)	no	1513/ 8020	1.11 (1.02-1.20)		
rs13281615	Smoking (ever)	yes	1286/ 6612	1.08 (0.99-1.18)		
rs13281615	Smoking (ever/never)	combined	2799/14632		0.97	6.3E-01
rs13281615	Smoking (pack-years)	0	1390/ 6895	1.14 (1.04-1.24)		
rs13281615	Smoking (pack-years)	0-<10	555/ 2779	1.10 (0.96-1.26)		
rs13281615	Smoking (pack-years)	10-<20	268/ 1224	1.04 (0.86-1.26)		
rs13281615	Smoking (pack-years)	>=20	255/ 1547	1.05 (0.86-1.28)		
rs13281615	Smoking (pack-years/10)	combined	2468/12445		0.98	4.7E-01
rs13281615	Physical activity during year before reference date (h/week)	0	105/ 999	0.99 (0.74-1.32)		
rs13281615	Physical activity during year before reference date (h/week)	0-<3.5	246/ 2151	1.11 (0.91-1.34)		
rs13281615	Physical activity during year before reference date (h/week)	3.5-<7	142/ 1321	0.98 (0.76-1.26)		
rs13281615	Physical activity during year before reference date (h/week)	>=7	229/ 1276	0.88 (0.72-1.08)		
rs13281615	Physical activity during year before reference date (square root of h/week)	combined	722/ 5747		0.95	2.6E-01
rs13387042	Age at menarche (years)	<=11	656/ 3720	0.96 (0.85-1.08)		
rs13387042	Age at menarche (years)	12-13	2000/12415	0.98 (0.92-1.05)		
rs13387042	Age at menarche (years)	>=14	1685/12786	0.91 (0.85-0.98)		
rs13387042	Age at menarche (years/2)	combined	4341/28921		0.98	5.4E-01
rs13387042	Parous	no	730/ 4330	1.04 (0.93-1.17)		
rs13387042	Parous	yes	4270/28506	0.92 (0.88-0.97)		
rs13387042	Parous (yes/no)	combined	5000/32836		0.88	5.6E-02
rs13387042	Number of births (among parous)	1	1023/ 5813	0.91 (0.83-1.00)		
rs13387042	Number of births (among parous)	2	1946/13131	0.92 (0.86-0.98)		
rs13387042	Number of births (among parous)	3	806/ 5779	0.90 (0.81-1.00)		
rs13387042	Number of births (among parous)	>=4	423/ 2908	1.02 (0.88-1.19)		
rs13387042	Number of births (among parous)	combined	4198/27631		1.03	2.1E-01
rs13387042	Age at first birth (among parous, years)	<20	517/ 2863	0.95 (0.83-1.09)		
rs13387042	Age at first birth (among parous, years)	20-24	1618/11201	0.87 (0.81-0.94)		
rs13387042	Age at first birth (among parous, years)	25-29	1115/ 8361	0.97 (0.88-1.06)		

SNP	Variable	Stratum	N (cases/ controls)	OR (95% CI)[1]	OR int[2]	P int[3]
rs13387042	Age at first birth (among parous, years)	>=30	527/ 3600	0.99 (0.86-1.13)		
rs13387042	Age at first birth (among parous, years/5)	combined	3777/26025		1.03	2.8E-01
rs13387042	Ever breastfed (among parous, yes/no)	no	509/ 2884	0.87 (0.75-1.00)		
rs13387042	Ever breastfed (among parous, yes/no)	yes	1689/10331	0.90 (0.83-0.97)		
rs13387042	Ever breastfed (among parous, yes/no)	combined	2198/13215		1.03	6.7E-01
rs13387042	Usual adult BMI, age<54	<25	844/ 3046	0.85 (0.76-0.95)		
rs13387042	Usual adult BMI, age<54	25-<30	266/ 1028	0.89 (0.73-1.08)		
rs13387042	Usual adult BMI, age<54	>=30	105/ 508	1.03 (0.75-1.40)		
rs13387042	Usual adult BMI (BMI/5), age<54	combined	1215/ 4582		1.10	7.6E-02
rs13387042	Usual adult BMI, age>=54	<25	775/ 6492	0.94 (0.84-1.05)		
rs13387042	Usual adult BMI, age>=54	25-<30	354/ 2766	0.95 (0.80-1.11)		
rs13387042	Usual adult BMI, age>=54	>=30	114/ 954	0.97 (0.73-1.29)		
rs13387042	Usual adult BMI (BMI/5), age>=54	combined	1243/10212		1.05	3.2E-01
rs13387042	Usual adult height (cm)	<160	857/ 5394	0.91 (0.82-1.01)		
rs13387042	Usual adult height (cm)	160-<165	1022/ 6464	1.02 (0.92-1.12)		
rs13387042	Usual adult height (cm)	165-<170	892/ 5332	0.84 (0.76-0.93)		
rs13387042	Usual adult height (cm)	>=170	616/ 3532	1.02 (0.90-1.16)		
rs13387042	Usual adult height (cm/5)	combined	3387/20722		1.02	3.1E-01
rs13387042	Ever use of oral contraceptives	no	1237/ 8529	0.91 (0.84-1.00)		
rs13387042	Ever use of oral contraceptives	yes	1593/10511	0.97 (0.90-1.04)		
rs13387042	Ever use of oral contraceptives (yes/no)	combined	2830/19040		1.06	3.2E-01
rs13387042	Duration of oral contraceptive use (years)	0	1237/ 8529	0.91 (0.84-1.00)		
rs13387042	Duration of oral contraceptive use (years)	>0-<5	493/ 3488	0.90 (0.78-1.03)		
rs13387042	Duration of oral contraceptive use (years)	5-<10	384/ 2562	0.92 (0.79-1.08)		
rs13387042	Duration of oral contraceptive use (years)	>=10	684/ 4155	1.06 (0.94-1.19)		
rs13387042	Duration of oral contraceptive use (years/5)	combined	2798/18734		1.04	9.1E-02
rs13387042	Current use of combined estrogen/ progestagen MHT	never	583/ 4533	0.94 (0.83-1.07)		
rs13387042	Current use of combined estrogen/ progestagen MHT	EPCurrent	149/ 1373	0.84 (0.66-1.07)		
rs13387042	Current use of combined estrogen/ progestagen MHT (yes/no)	combined	1044/ 9043		0.88	3.3E-01
rs13387042	Current use of estrogen only MHT	never	607/ 4614	0.94 (0.83-1.06)		
rs13387042	Current use of estrogen only MHT	ECurrent	84/ 1064	0.94 (0.69-1.29)		
rs13387042	Current use of estrogen only MHT (yes/no)	combined	1088/ 9229		1.01	9.7E-01
rs13387042	Duration of combined estrogen/progestagen MHT among current users (years)	never	583/ 4533	0.94 (0.83-1.07)		
rs13387042	Duration of combined estrogen/progestagen MHT among current users (years)	>0-<5	38/ 373	0.72 (0.44-1.17)		
rs13387042	Duration of combined estrogen/progestagen MHT among current users (years)	5-<10	51/ 415	0.82 (0.53-1.27)		
rs13387042	Duration of combined estrogen/progestagen MHT among current users (years)	>=10	52/ 527	1.09 (0.73-1.62)		
rs13387042	Duration of combined estrogen/progestagen MHT among current users (years/5)	combined	1034/ 8948		1.02	7.0E-01
rs13387042	Duration of estrogen only MHT among current users (years)	never	607/ 4614	0.94 (0.83-1.06)		
rs13387042	Duration of estrogen only MHT among current users (years)	>0-<5	25/ 299	0.86 (0.48-1.55)		
rs13387042	Duration of estrogen only MHT among current users (years)	5-<10	28/ 288	1.10 (0.63-1.92)		
rs13387042	Duration of estrogen only MHT among current users (years)	>=10	30/ 438	0.90 (0.53-1.51)		
rs13387042	Duration of estrogen only MHT among current users (years/5)	combined	1083/ 9112		1.00	9.9E-01
rs13387042	Mean lifetime intake of alcohol (g/day)	0	624/ 3007	0.87 (0.76-0.98)		
rs13387042	Mean lifetime intake of alcohol (g/day)	>0-<10	690/ 4942	0.98 (0.88-1.10)		
rs13387042	Mean lifetime intake of alcohol (g/day)	>=10-<20	131/ 1134	1.10 (0.85-1.43)		
rs13387042	Mean lifetime intake of alcohol (g/day)	>=20	107/ 801	0.88 (0.66-1.18)		
rs13387042	Mean lifetime intake of alcohol (10g/day)	combined	1552/ 9884		1.05	1.9E-01

SNP	Variable	Stratum	N (cases/ controls)	OR (95% CI)[1]	OR int[2]	P int[3]
rs13387042	Smoking (ever)	no	1739/10467	0.92 (0.86-1.00)		
rs13387042	Smoking (ever)	yes	1473/ 8786	0.95 (0.88-1.03)		
rs13387042	Smoking (ever/never)	combined	3212/19253		1.03	6.3E-01
rs13387042	Smoking (pack-years)	0	1611/ 9289	0.91 (0.84-0.98)		
rs13387042	Smoking (pack-years)	0-<10	620/ 3731	0.90 (0.79-1.02)		
rs13387042	Smoking (pack-years)	10-<20	308/ 1682	0.99 (0.83-1.18)		
rs13387042	Smoking (pack-years)	>=20	320/ 2211	1.02 (0.86-1.21)		
rs13387042	Smoking (pack-years/10)	combined	2859/16913		1.04	1.6E-01
rs13387042	Physical activity during year before reference date (h/week)	0	112/ 1071	1.07 (0.81-1.42)		
rs13387042	Physical activity during year before reference date (h/week)	0-<3.5	330/ 2827	0.94 (0.80-1.11)		
rs13387042	Physical activity during year before reference date (h/week)	3.5-<7	242/ 2211	0.88 (0.73-1.06)		
rs13387042	Physical activity during year before reference date (h/week)	>=7	345/ 3685	0.97 (0.83-1.14)		
rs13387042	Physical activity during year before reference date (square root of h/week)	combined	1029/ 9794		1.00	9.2E-01
rs17468277[7]	Age at menarche (years)	<=11	645/ 3868	1.02 (0.85-1.23)		
rs17468277	Age at menarche (years)	12-13	1930/12749	0.86 (0.77-0.97)		
rs17468277	Age at menarche (years)	>=14	1630/12811	0.89 (0.79-1.00)		
rs17468277	Age at menarche (years/2)	combined	4205/29428		0.95	3.0E-01
rs17468277	Parous	no	702/ 4395	1.00 (0.84-1.20)		
rs17468277	Parous	yes	4170/28798	0.88 (0.82-0.95)		
rs17468277	Parous (yes/no)	combined	4872/33193		0.87	1.7E-01
rs17468277	Number of births (among parous)	1	988/ 5804	0.86 (0.73-1.00)		
rs17468277	Number of births (among parous)	2	1883/13175	0.89 (0.80-0.99)		
rs17468277	Number of births (among parous)	3	813/ 5942	0.95 (0.81-1.12)		
rs17468277	Number of births (among parous)	>=4	414/ 3035	0.79 (0.62-1.00)		
rs17468277	Number of births (among parous)	combined	4098/27956		0.99	8.7E-01
rs17468277	Age at first birth (among parous, years)	<20	489/ 2792	0.82 (0.66-1.02)		
rs17468277	Age at first birth (among parous, years)	20-24	1573/11439	0.88 (0.78-0.99)		
rs17468277	Age at first birth (among parous, years)	25-29	1096/ 8457	0.84 (0.73-0.97)		
rs17468277	Age at first birth (among parous, years)	>=30	518/ 3653	1.03 (0.83-1.27)		
rs17468277	Age at first birth (among parous, years/5)	combined	3676/26341		1.02	6.5E-01
rs17468277	Ever breastfed (among parous, yes/no)	no	489/ 2759	0.76 (0.60-0.96)		
rs17468277	Ever breastfed (among parous, yes/no)	yes	1677/10127	0.89 (0.79-1.00)		
rs17468277	Ever breastfed (among parous, yes/no)	combined	2166/12886		1.17	2.4E-01
rs17468277	Usual adult BMI, age<54	<25	802/ 2777	0.85 (0.71-1.01)		
rs17468277	Usual adult BMI, age<54	25-<30	249/ 925	0.76 (0.55-1.06)		
rs17468277	Usual adult BMI, age<54	>=30	102/ 478	1.00 (0.62-1.60)		
rs17468277	Usual adult BMI (BMI/5), age<54	combined	1153/ 4180		1.07	4.3E-01
rs17468277	Usual adult BMI, age>=54	<25	780/ 6505	0.93 (0.79-1.09)		
rs17468277	Usual adult BMI, age>=54	25-<30	357/ 2767	0.79 (0.61-1.01)		
rs17468277	Usual adult BMI, age>=54	>=30	117/ 960	0.89 (0.58-1.37)		
rs17468277	Usual adult BMI (BMI/5), age>=54	combined	1254/10232		0.96	6.3E-01
rs17468277	Usual adult height (cm)	<160	831/ 5296	0.95 (0.81-1.12)		
rs17468277	Usual adult height (cm)	160-<165	1001/ 6255	0.84 (0.72-0.98)		
rs17468277	Usual adult height (cm)	165-<170	866/ 5140	0.89 (0.76-1.04)		
rs17468277	Usual adult height (cm)	>=170	603/ 3433	0.89 (0.73-1.08)		
rs17468277	Usual adult height (cm/5)	combined	3301/20124		0.99	6.7E-01
rs17468277	Ever use of oral contraceptives	no	1257/ 8361	0.86 (0.75-0.98)		
rs17468277	Ever use of oral contraceptives	yes	1569/10344	0.90 (0.80-1.01)		
rs17468277	Ever use of oral contraceptives (yes/no)	combined	2826/18705		1.05	6.2E-01

SNP	Variable	Stratum	N (cases/ controls)	OR (95% CI)[1]	OR int[2]	P int[3]
rs17468277	Duration of oral contraceptive use (years)	0	1257/ 8361	0.86 (0.75-0.98)		
rs17468277	Duration of oral contraceptive use (years)	>0-<5	493/ 3493	0.96 (0.78-1.17)		
rs17468277	Duration of oral contraceptive use (years)	5-<10	389/ 2546	0.85 (0.67-1.09)		
rs17468277	Duration of oral contraceptive use (years)	>=10	656/ 4015	0.88 (0.73-1.06)		
rs17468277	Duration of oral contraceptive use (years/5)	combined	2795/18415		0.98	6.0E-01
rs17468277	Current use of combined estrogen/ progestagen MHT	never	579/ 4542	0.87 (0.72-1.06)		
rs17468277	Current use of combined estrogen/ progestagen MHT	EPCurrent	150/ 1373	0.87 (0.60-1.25)		
rs17468277	Current use of combined estrogen/ progestagen MHT (yes/no)	combined	1049/ 9060		0.92	6.9E-01
rs17468277	Current use of estrogen only MHT	never	603/ 4629	0.89 (0.74-1.08)		
rs17468277	Current use of estrogen only MHT	ECurrent	88/ 1063	0.79 (0.49-1.28)		
rs17468277	Current use of estrogen only MHT (yes/no)	combined	1092/ 9253		0.84	5.0E-01
rs17468277	Duration of combined estrogen/progestagen MHT among current users (years)	never	579/ 4542	0.87 (0.72-1.06)		
rs17468277	Duration of combined estrogen/progestagen MHT among current users (years)	>0-<5	38/ 376	1.88 (1.02-3.48)		
rs17468277	Duration of combined estrogen/progestagen MHT among current users (years)	5-<10	52/ 411	0.42 (0.18-0.97)		
rs17468277	Duration of combined estrogen/progestagen MHT among current users (years)	>=10	52/ 528	0.83 (0.45-1.54)		
rs17468277	Duration of combined estrogen/progestagen MHT among current users (years/5)	combined	1039/ 8965		0.93	4.3E-01
rs17468277	Duration of estrogen only MHT among current users (years)	never	603/ 4629	0.89 (0.74-1.08)		
rs17468277	Duration of estrogen only MHT among current users (years)	>0-<5	27/ 301	0.84 (0.36-1.92)		
rs17468277	Duration of estrogen only MHT among current users (years)	5-<10	30/ 286	0.89 (0.38-2.06)		
rs17468277	Duration of estrogen only MHT among current users (years)	>=10	30/ 437	0.73 (0.32-1.65)		
rs17468277	Duration of estrogen only MHT among current users (years/5)	combined	1088/ 9136		0.88	3.0E-01
rs17468277	Mean lifetime intake of alcohol (g/day)	0	597/ 2928	0.91 (0.75-1.09)		
rs17468277	Mean lifetime intake of alcohol (g/day)	>0-<10	609/ 4586	0.84 (0.69-1.01)		
rs17468277	Mean lifetime intake of alcohol (g/day)	>=10-<20	109/ 1033	0.98 (0.64-1.51)		
rs17468277	Mean lifetime intake of alcohol (g/day)	>=20	83/ 758	1.22 (0.77-1.94)		
rs17468277	Mean lifetime intake of alcohol (10g/day)	combined	1398/ 9305		1.05	3.5E-01
rs17468277	Smoking (ever)	no	1679/10101	0.92 (0.82-1.03)		
rs17468277	Smoking (ever)	yes	1428/ 8408	0.84 (0.74-0.96)		
rs17468277	Smoking (ever/never)	combined	3107/18509		0.91	3.0E-01
rs17468277	Smoking (pack-years)	0	1549/ 8913	0.93 (0.82-1.05)		
rs17468277	Smoking (pack-years)	0-<10	604/ 3572	0.79 (0.65-0.97)		
rs17468277	Smoking (pack-years)	10-<20	290/ 1583	0.84 (0.64-1.10)		
rs17468277	Smoking (pack-years)	>=20	311/ 2097	0.82 (0.63-1.08)		
rs17468277	Smoking (pack-years/10)	combined	2754/16165		0.96	3.7E-01
rs17468277	Physical activity during year before reference date (h/week)	0	112/ 1069	0.82 (0.51-1.33)		
rs17468277	Physical activity during year before reference date (h/week)	0-<3.5	327/ 2830	0.92 (0.72-1.18)		
rs17468277	Physical activity during year before reference date (h/week)	3.5-<7	245/ 2219	0.93 (0.71-1.24)		
rs17468277	Physical activity during year before reference date (h/week)	>=7	345/ 3684	0.83 (0.65-1.07)		
rs17468277	Physical activity during year before reference date (square root of h/week)	combined	1029/ 9802		1.04	5.3E-01
rs1982073	Age at menarche (years)	<=11	445/ 2588	1.08 (0.93-1.26)		
rs1982073	Age at menarche (years)	12-13	1297/ 8764	1.13 (1.04-1.24)		
rs1982073	Age at menarche (years)	>=14	1056/ 8570	0.96 (0.87-1.05)		
rs1982073	Age at menarche (years/2)	combined	2798/19922		0.92	3.1E-02
rs1982073	Parous	no	419/ 2852	0.87 (0.75-1.02)		
rs1982073	Parous	yes	2582/18339	1.10 (1.03-1.17)		
rs1982073	Parous (yes/no)	combined	3001/21191		1.26	8.0E-03

SNP	Variable	Stratum	N (cases/ controls)	OR (95% CI)[1]	OR int[2]	P int[3]
rs1982073	Number of births (among parous)	1	596/ 3460	1.06 (0.93-1.21)		
rs1982073	Number of births (among parous)	2	1186/ 8602	1.12 (1.02-1.22)		
rs1982073	Number of births (among parous)	3	497/ 3855	1.12 (0.98-1.29)		
rs1982073	Number of births (among parous)	>=4	237/ 1841	0.98 (0.80-1.19)		
rs1982073	Number of births (among parous)	combined	2516/17758		0.98	6.0E-01
rs1982073	Age at first birth (among parous, years)	<20	348/ 1736	1.13 (0.95-1.34)		
rs1982073	Age at first birth (among parous, years)	20-24	1064/ 7541	1.09 (0.99-1.21)		
rs1982073	Age at first birth (among parous, years)	25-29	716/ 5891	1.09 (0.97-1.22)		
rs1982073	Age at first birth (among parous, years)	>=30	348/ 2528	0.98 (0.82-1.15)		
rs1982073	Age at first birth (among parous, years/5)	combined	2476/17696		0.94	8.3E-02
rs1982073	Ever breastfed (among parous, yes/no)	no	177/ 727	1.05 (0.83-1.33)		
rs1982073	Ever breastfed (among parous, yes/no)	yes	931/ 4875	1.07 (0.96-1.18)		
rs1982073	Ever breastfed (among parous, yes/no)	combined	1108/ 5602		1.02	9.1E-01
rs1982073	Usual adult BMI, age<54	<25	519/ 1953	1.05 (0.91-1.21)		
rs1982073	Usual adult BMI, age<54	25-<30	154/ 600	1.42 (1.10-1.84)		
rs1982073	Usual adult BMI, age<54	>=30	64/ 271	0.75 (0.49-1.13)		
rs1982073	Usual adult BMI (BMI/5), age<54	combined	737/ 2824		0.96	5.6E-01
rs1982073	Usual adult BMI, age>=54	<25	260/ 1824	1.08 (0.89-1.32)		
rs1982073	Usual adult BMI, age>=54	25-<30	150/ 1042	1.10 (0.86-1.41)		
rs1982073	Usual adult BMI, age>=54	>=30	39/ 357	0.93 (0.56-1.55)		
rs1982073	Usual adult BMI (BMI/5), age>=54	combined	449/ 3223		0.98	8.3E-01
rs1982073	Usual adult height (cm)	<160	459/ 2386	0.97 (0.84-1.13)		
rs1982073	Usual adult height (cm)	160-<165	546/ 2855	1.02 (0.89-1.16)		
rs1982073	Usual adult height (cm)	165-<170	451/ 2058	1.14 (0.98-1.32)		
rs1982073	Usual adult height (cm)	>=170	319/ 1311	1.03 (0.86-1.23)		
rs1982073	Usual adult height (cm/5)	combined	1775/ 8610		1.03	3.4E-01
rs1982073	Ever use of oral contraceptives	no	774/ 4141	1.04 (0.93-1.16)		
rs1982073	Ever use of oral contraceptives	yes	733/ 3466	1.02 (0.91-1.15)		
rs1982073	Ever use of oral contraceptives (yes/no)	combined	1507/ 7607		0.98	8.2E-01
rs1982073	Duration of oral contraceptive use (years)	0	774/ 4141	1.04 (0.93-1.16)		
rs1982073	Duration of oral contraceptive use (years)	>0-<5	229/ 1237	1.08 (0.88-1.33)		
rs1982073	Duration of oral contraceptive use (years)	5-<10	203/ 889	1.22 (0.98-1.52)		
rs1982073	Duration of oral contraceptive use (years)	>=10	279/ 1189	0.86 (0.71-1.04)		
rs1982073	Duration of oral contraceptive use (years/5)	combined	1485/ 7456		0.97	3.5E-01
rs1982073	Current use of combined estrogen/ progestagen MHT	never	279/ 1765	1.12 (0.93-1.34)		
rs1982073	Current use of combined estrogen/ progestagen MHT	EPCurrent	34/ 224	0.99 (0.60-1.63)		
rs1982073	Current use of combined estrogen/ progestagen MHT (yes/no)	combined	391/ 2542		0.90	7.0E-01
rs1982073	Current use of estrogen only MHT	never	301/ 1856	1.07 (0.90-1.28)		
rs1982073	Current use of estrogen only MHT	ECurrent	27/ 234	1.26 (0.73-2.19)		
rs1982073	Current use of estrogen only MHT (yes/no)	combined	433/ 2743		1.20	5.3E-01
rs1982073	Duration of combined estrogen/progestagen MHT among current users (years)	never	279/ 1765	1.12 (0.93-1.34)		
rs1982073	Duration of combined estrogen/progestagen MHT among current users (years)	>0-<5	14/ 112	0.55 (0.24-1.26)		
rs1982073	Duration of combined estrogen/progestagen MHT among current users (years)	5-<10	19/ 73	1.54 (0.75-3.15)		
rs1982073	Duration of combined estrogen/progestagen MHT among current users (years)	>=10	1/ 39	1.63 (0.07-36.64)		
rs1982073	Duration of combined estrogen/progestagen MHT among current users (years/5)	combined	391/ 2542		1.13	5.3E-01
rs1982073	Duration of estrogen only MHT among current users (years)	never	301/ 1856	1.07 (0.90-1.28)		
rs1982073	Duration of estrogen only MHT among current users (years)	>0-<5	11/ 105	0.84 (0.36-1.95)		

SNP	Variable	Stratum	N (cases/ controls)	OR (95% CI)[1]	OR int[2]	P int[3]
rs1982073	Duration of estrogen only MHT among current users (years)	5-<10	12/ 69	2.04 (0.84-4.95)		
rs1982073	Duration of estrogen only MHT among current users (years)	>=10	4/ 60	0.86 (0.14-5.08)		
rs1982073	Duration of estrogen only MHT among current users (years/5)	combined	431/ 2728		1.15	4.2E-01
rs1982073	Mean lifetime intake of alcohol (g/day)	0	418/ 1879	0.95 (0.81-1.11)		
rs1982073	Mean lifetime intake of alcohol (g/day)	>0-<10	267/ 1324	1.09 (0.90-1.32)		
rs1982073	Mean lifetime intake of alcohol (g/day)	>=10-<20	64/ 272	1.10 (0.74-1.64)		
rs1982073	Mean lifetime intake of alcohol (g/day)	>=20	48/ 158	0.82 (0.51-1.31)		
rs1982073	Mean lifetime intake of alcohol (10g/day)	combined	797/ 3633		1.05	2.9E-01
rs1982073	Smoking (ever)	no	957/ 4677	1.03 (0.93-1.14)		
rs1982073	Smoking (ever)	yes	818/ 3781	1.04 (0.94-1.17)		
rs1982073	Smoking (ever/never)	combined	1775/ 8458		1.02	8.3E-01
rs1982073	Smoking (pack-years)	0	879/ 3827	1.01 (0.90-1.12)		
rs1982073	Smoking (pack-years)	0-<10	352/ 1562	1.09 (0.92-1.29)		
rs1982073	Smoking (pack-years)	10-<20	175/ 671	0.91 (0.71-1.17)		
rs1982073	Smoking (pack-years)	>=20	141/ 713	1.14 (0.88-1.48)		
rs1982073	Smoking (pack-years/10)	combined	1547/ 6773		1.01	7.4E-01
rs1982073	Physical activity during year before reference date (h/week)	0	./ .			
rs1982073	Physical activity during year before reference date (h/week)	0-<3.5	99/ 918	1.10 (0.82-1.47)		
rs1982073	Physical activity during year before reference date (h/week)	3.5-<7	60/ 521	1.23 (0.83-1.83)		
rs1982073	Physical activity during year before reference date (h/week)	>=7	./ .			
rs1982073	Physical activity during year before reference date (square root of h/week)	combined	159/ 1439		1.12	7.6E-01
rs2046210	Age at menarche (years)	<=11	642/ 3411	1.16 (1.02-1.32)		
rs2046210	Age at menarche (years)	12-13	1840/10928	1.17 (1.09-1.27)		
rs2046210	Age at menarche (years)	>=14	1448/10540	1.22 (1.12-1.32)		
rs2046210	Age at menarche (years/2)	combined	3930/24879		1.00	9.2E-01
rs2046210	Parous	no	677/ 3622	1.18 (1.04-1.34)		
rs2046210	Parous	yes	3897/24261	1.20 (1.14-1.27)		
rs2046210	Parous (yes/no)	combined	4574/27883		1.02	8.0E-01
rs2046210	Number of births (among parous)	1	886/ 4597	1.20 (1.07-1.34)		
rs2046210	Number of births (among parous)	2	1767/11054	1.19 (1.10-1.28)		
rs2046210	Number of births (among parous)	3	741/ 4916	1.17 (1.04-1.31)		
rs2046210	Number of births (among parous)	>=4	375/ 2487	1.39 (1.18-1.63)		
rs2046210	Number of births (among parous)	combined	3769/23054		1.03	3.2E-01
rs2046210	Age at first birth (among parous, years)	<20	485/ 2343	1.32 (1.14-1.53)		
rs2046210	Age at first birth (among parous, years)	20-24	1469/ 9507	1.19 (1.10-1.30)		
rs2046210	Age at first birth (among parous, years)	25-29	1019/ 7182	1.06 (0.96-1.18)		
rs2046210	Age at first birth (among parous, years)	>=30	496/ 3184	1.28 (1.11-1.47)		
rs2046210	Age at first birth (among parous, years/5)	combined	3469/22216		0.99	7.1E-01
rs2046210	Ever breastfed (among parous, yes/no)	no	478/ 2313	1.26 (1.09-1.46)		
rs2046210	Ever breastfed (among parous, yes/no)	yes	1618/ 7961	1.19 (1.09-1.29)		
rs2046210	Ever breastfed (among parous, yes/no)	combined	2096/10274		0.94	4.9E-01
rs2046210	Usual adult BMI, age<54	<25	870/ 2887	1.29 (1.15-1.44)		
rs2046210	Usual adult BMI, age<54	25-<30	273/ 1000	1.31 (1.06-1.61)		
rs2046210	Usual adult BMI, age<54	>=30	115/ 501	0.94 (0.69-1.30)		
rs2046210	Usual adult BMI (BMI/5), age<54	combined	1258/ 4388		0.93	1.6E-01
rs2046210	Usual adult BMI, age>=54	<25	653/ 4084	1.10 (0.97-1.24)		
rs2046210	Usual adult BMI, age>=54	25-<30	322/ 2118	1.22 (1.02-1.47)		
rs2046210	Usual adult BMI, age>=54	>=30	112/ 854	1.04 (0.76-1.42)		
rs2046210	Usual adult BMI (BMI/5), age>=54	combined	1087/ 7056		0.97	6.5E-01

SNP	Variable	Stratum	N (cases/ controls)	OR (95% CI)[1]	OR int[2]	P int[3]
rs2046210	Usual adult height (cm)	<160	742/ 4210	1.15 (1.02-1.30)		
rs2046210	Usual adult height (cm)	160-<165	847/ 4896	1.15 (1.03-1.29)		
rs2046210	Usual adult height (cm)	165-<170	728/ 3814	1.23 (1.09-1.39)		
rs2046210	Usual adult height (cm)	>=170	516/ 2523	1.27 (1.10-1.46)		
rs2046210	Usual adult height (cm/5)	combined	2833/15443		1.04	1.2E-01
rs2046210	Ever use of oral contraceptives	no	1113/ 6631	1.10 (1.00-1.22)		
rs2046210	Ever use of oral contraceptives	yes	1469/ 8053	1.25 (1.15-1.36)		
rs2046210	Ever use of oral contraceptives (yes/no)	combined	2582/14684		1.13	5.5E-02
rs2046210	Duration of oral contraceptive use (years)	0	1113/ 6631	1.10 (1.00-1.22)		
rs2046210	Duration of oral contraceptive use (years)	>0-<5	461/ 2781	1.24 (1.07-1.45)		
rs2046210	Duration of oral contraceptive use (years)	5-<10	371/ 2012	1.27 (1.07-1.51)		
rs2046210	Duration of oral contraceptive use (years)	>=10	612/ 3005	1.25 (1.10-1.42)		
rs2046210	Duration of oral contraceptive use (years/5)	combined	2557/14429		1.03	2.5E-01
rs2046210	Current use of combined estrogen/ progestagen MHT	never	509/ 3278	1.17 (1.02-1.35)		
rs2046210	Current use of combined estrogen/ progestagen MHT	EPCurrent	118/ 783	1.15 (0.86-1.54)		
rs2046210	Current use of combined estrogen/ progestagen MHT (yes/no)	combined	883/ 5875		1.02	8.9E-01
rs2046210	Current use of estrogen only MHT	never	533/ 3360	1.18 (1.02-1.35)		
rs2046210	Current use of estrogen only MHT	ECurrent	75/ 673	1.09 (0.76-1.55)		
rs2046210	Current use of estrogen only MHT (yes/no)	combined	927/ 6060		0.95	8.0E-01
rs2046210	Duration of combined estrogen/progestagen MHT among current users (years)	never	509/ 3278	1.17 (1.02-1.35)		
rs2046210	Duration of combined estrogen/progestagen MHT among current users (years)	>0-<5	32/ 253	1.84 (1.08-3.12)		
rs2046210	Duration of combined estrogen/progestagen MHT among current users (years)	5-<10	44/ 232	0.97 (0.59-1.60)		
rs2046210	Duration of combined estrogen/progestagen MHT among current users (years)	>=10	34/ 247	0.80 (0.44-1.43)		
rs2046210	Duration of combined estrogen/progestagen MHT among current users (years/5)	combined	873/ 5791		0.94	4.4E-01
rs2046210	Duration of estrogen only MHT among current users (years)	never	533/ 3360	1.17 (1.02-1.35)		
rs2046210	Duration of estrogen only MHT among current users (years)	>0-<5	24/ 185	0.97 (0.50-1.88)		
rs2046210	Duration of estrogen only MHT among current users (years)	5-<10	22/ 183	0.95 (0.50-1.84)		
rs2046210	Duration of estrogen only MHT among current users (years)	>=10	28/ 272	1.47 (0.83-2.59)		
rs2046210	Duration of estrogen only MHT among current users (years/5)	combined	922/ 5947		1.04	6.7E-01
rs2046210	Mean lifetime intake of alcohol (g/day)	0	565/ 2503	1.19 (1.04-1.36)		
rs2046210	Mean lifetime intake of alcohol (g/day)	>0-<10	581/ 2857	1.15 (1.01-1.31)		
rs2046210	Mean lifetime intake of alcohol (g/day)	>=10-<20	115/ 593	1.53 (1.14-2.06)		
rs2046210	Mean lifetime intake of alcohol (g/day)	>=20	93/ 389	1.28 (0.92-1.77)		
rs2046210	Mean lifetime intake of alcohol (10g/day)	combined	1354/ 6342		1.01	6.7E-01
rs2046210	Smoking (ever)	no	1392/ 7648	1.22 (1.12-1.33)		
rs2046210	Smoking (ever)	yes	1298/ 6416	1.20 (1.10-1.32)		
rs2046210	Smoking (ever/never)	combined	2690/14064		0.98	8.1E-01
rs2046210	Smoking (pack-years)	0	1322/ 6856	1.22 (1.12-1.34)		
rs2046210	Smoking (pack-years)	0-<10	567/ 2747	1.11 (0.96-1.28)		
rs2046210	Smoking (pack-years)	10-<20	277/ 1240	1.26 (1.04-1.54)		
rs2046210	Smoking (pack-years)	>=20	296/ 1587	1.29 (1.07-1.56)		
rs2046210	Smoking (pack-years/10)	combined	2462/12430		1.03	3.8E-01
rs2046210	Physical activity during year before reference date (h/week)	0	107/ 1051	1.62 (1.20-2.18)		
rs2046210	Physical activity during year before reference date (h/week)	0-<3.5	310/ 2439	1.20 (1.00-1.43)		
rs2046210	Physical activity during year before reference date (h/week)	3.5-<7	188/ 1492	1.02 (0.82-1.28)		
rs2046210	Physical activity during year before reference date (h/week)	>=7	254/ 1359	1.03 (0.84-1.26)		

SNP	Variable	Stratum	N (cases/ controls)	OR (95% CI)[1]	OR int[2]	P int[3]
rs2046210	Physical activity during year before reference date (square root of h/week)	combined	859/ 6341		0.90	1.6E-02
rs2823093	Age at menarche (years)	<=11	398/ 1810	1.07 (0.89-1.28)		
rs2823093	Age at menarche (years)	12-13	1080/ 5221	0.98 (0.87-1.10)		
rs2823093	Age at menarche (years)	>=14	845/ 4696	0.93 (0.82-1.06)		
rs2823093	Age at menarche (years/2)	combined	2323/11727		0.96	4.3E-01
rs2823093	Parous	no	380/ 1972	0.95 (0.79-1.14)		
rs2823093	Parous	yes	2509/12491	1.01 (0.94-1.09)		
rs2823093	Parous (yes/no)	combined	2889/14463		1.07	5.3E-01
rs2823093	Number of births (among parous)	1	536/ 2264	0.97 (0.82-1.14)		
rs2823093	Number of births (among parous)	2	1128/ 5182	1.08 (0.96-1.20)		
rs2823093	Number of births (among parous)	3	528/ 2700	1.00 (0.85-1.17)		
rs2823093	Number of births (among parous)	>=4	267/ 1559	0.85 (0.68-1.06)		
rs2823093	Number of births (among parous)	combined	2459/11705		0.99	6.8E-01
rs2823093	Age at first birth (among parous, years)	<20	306/ 965	1.00 (0.80-1.24)		
rs2823093	Age at first birth (among parous, years)	20-24	850/ 4435	0.95 (0.83-1.08)		
rs2823093	Age at first birth (among parous, years)	25-29	599/ 3382	1.03 (0.88-1.20)		
rs2823093	Age at first birth (among parous, years)	>=30	302/ 1434	0.96 (0.77-1.20)		
rs2823093	Age at first birth (among parous, years/5)	combined	2057/10216		1.03	4.9E-01
rs2823093	Ever breastfed (among parous, yes/no)	no	193/ 899	0.98 (0.76-1.26)		
rs2823093	Ever breastfed (among parous, yes/no)	yes	661/ 4441	0.96 (0.84-1.10)		
rs2823093	Ever breastfed (among parous, yes/no)	combined	854/ 5340		0.98	8.9E-01
rs2823093	Usual adult BMI, age<54	<25	320/ 1108	0.76 (0.61-0.96)		
rs2823093	Usual adult BMI, age<54	25-<30	97/ 391	1.10 (0.75-1.61)		
rs2823093	Usual adult BMI, age<54	>=30	48/ 220	1.28 (0.78-2.11)		
rs2823093	Usual adult BMI (BMI/5), age<54	combined	465/ 1719		1.20	6.3E-02
rs2823093	Usual adult BMI, age>=54	<25	321/ 2552	1.00 (0.83-1.20)		
rs2823093	Usual adult BMI, age>=54	25-<30	142/ 1252	0.97 (0.74-1.28)		
rs2823093	Usual adult BMI, age>=54	>=30	47/ 481	1.37 (0.88-2.13)		
rs2823093	Usual adult BMI (BMI/5), age>=54	combined	510/ 4285		1.07	4.1E-01
rs2823093	Usual adult height (cm)	<160	311/ 2338	0.94 (0.77-1.15)		
rs2823093	Usual adult height (cm)	160-<165	376/ 2812	0.87 (0.72-1.04)		
rs2823093	Usual adult height (cm)	165-<170	383/ 2359	1.13 (0.95-1.35)		
rs2823093	Usual adult height (cm)	>=170	298/ 1585	1.10 (0.90-1.34)		
rs2823093	Usual adult height (cm/5)	combined	1368/ 9094		1.07	5.6E-02
rs2823093	Ever use of oral contraceptives	no	391/ 3401	1.18 (1.00-1.39)		
rs2823093	Ever use of oral contraceptives	yes	957/ 5435	0.92 (0.82-1.03)		
rs2823093	Ever use of oral contraceptives (yes/no)	combined	1348/ 8836		0.78	1.5E-02
rs2823093	Duration of oral contraceptive use (years)	0	391/ 3401	1.18 (1.00-1.39)		
rs2823093	Duration of oral contraceptive use (years)	>0-<5	270/ 1770	0.95 (0.77-1.17)		
rs2823093	Duration of oral contraceptive use (years)	5-<10	262/ 1343	0.95 (0.76-1.18)		
rs2823093	Duration of oral contraceptive use (years)	>=10	404/ 2128	0.90 (0.75-1.07)		
rs2823093	Duration of oral contraceptive use (years/5)	combined	1327/ 8642		0.95	9.2E-02
rs2823093	Current use of combined estrogen/ progestagen MHT	never	239/ 1890	1.01 (0.81-1.25)		
rs2823093	Current use of combined estrogen/ progestagen MHT	EPCurrent	90/ 553	1.06 (0.74-1.50)		
rs2823093	Current use of combined estrogen/ progestagen MHT (yes/no)	combined	506/ 3709		1.07	7.2E-01
rs2823093	Current use of estrogen only MHT	never	239/ 1890	1.01 (0.81-1.25)		
rs2823093	Current use of estrogen only MHT	ECurrent	57/ 454	1.29 (0.86-1.92)		
rs2823093	Current use of estrogen only MHT (yes/no)	combined	507/ 3735		1.35	1.8E-01

SNP	Variable	Stratum	N (cases/ controls)	OR (95% CI)[1]	OR int[2]	P int[3]
rs2823093	Duration of combined estrogen/progestagen MHT among current users (years)	never	239/ 1890	1.01 (0.81-1.25)		
rs2823093	Duration of combined estrogen/progestagen MHT among current users (years)	>0-<5	18/ 156	2.02 (0.94-4.34)		
rs2823093	Duration of combined estrogen/progestagen MHT among current users (years)	5-<10	33/ 162	1.01 (0.54-1.87)		
rs2823093	Duration of combined estrogen/progestagen MHT among current users (years)	>=10	31/ 186	0.94 (0.52-1.70)		
rs2823093	Duration of combined estrogen/progestagen MHT among current users (years/5)	combined	497/ 3631		0.98	8.1E-01
rs2823093	Duration of estrogen only MHT among current users (years)	never	239/ 1890	1.01 (0.81-1.25)		
rs2823093	Duration of estrogen only MHT among current users (years)	>0-<5	18/ 143	1.54 (0.78-3.03)		
rs2823093	Duration of estrogen only MHT among current users (years)	5-<10	21/ 129	1.21 (0.58-2.51)		
rs2823093	Duration of estrogen only MHT among current users (years)	>=10	18/ 165	1.15 (0.56-2.34)		
rs2823093	Duration of estrogen only MHT among current users (years/5)	combined	507/ 3681		1.07	4.9E-01
rs2823093	Mean lifetime intake of alcohol (g/day)	0	116/ 1044	0.97 (0.71-1.33)		
rs2823093	Mean lifetime intake of alcohol (g/day)	>0-<10	294/ 1799	0.90 (0.74-1.11)		
rs2823093	Mean lifetime intake of alcohol (g/day)	>=10-<20	50/ 381	1.03 (0.64-1.69)		
rs2823093	Mean lifetime intake of alcohol (g/day)	>=20	49/ 285	1.33 (0.84-2.10)		
rs2823093	Mean lifetime intake of alcohol (10g/day)	combined	509/ 3509		0.99	8.8E-01
rs2823093	Smoking (ever)	no	756/ 5150	1.06 (0.93-1.20)		
rs2823093	Smoking (ever)	yes	622/ 3706	0.92 (0.80-1.06)		
rs2823093	Smoking (ever/never)	combined	1378/ 8856		0.87	1.4E-01
rs2823093	Smoking (pack-years)	0	630/ 4042	1.03 (0.89-1.18)		
rs2823093	Smoking (pack-years)	0-<10	216/ 1424	0.87 (0.68-1.12)		
rs2823093	Smoking (pack-years)	10-<20	109/ 620	0.96 (0.69-1.35)		
rs2823093	Smoking (pack-years)	>=20	130/ 773	0.99 (0.72-1.34)		
rs2823093	Smoking (pack-years/10)	combined	1085/ 6859		0.98	7.1E-01
rs2823093	Physical activity during year before reference date (h/week)	0	99/ 587	0.86 (0.61-1.21)		
rs2823093	Physical activity during year before reference date (h/week)	0-<3.5	201/ 1578	0.79 (0.61-1.01)		
rs2823093	Physical activity during year before reference date (h/week)	3.5-<7	161/ 1061	1.04 (0.80-1.35)		
rs2823093	Physical activity during year before reference date (h/week)	>=7	238/ 1139	1.08 (0.87-1.36)		
rs2823093	Physical activity during year before reference date (square root of h/week)	combined	699/ 4365		1.08	1.2E-01
rs2981582	Age at menarche (years)	<=11	666/ 3999	0.99 (0.88-1.12)		
rs2981582	Age at menarche (years)	12-13	2017/12586	1.07 (1.00-1.15)		
rs2981582	Age at menarche (years)	>=14	1692/11554	1.04 (0.96-1.12)		
rs2981582	Age at menarche (years/2)	combined	4375/28139		1.02	5.7E-01
rs2981582	Parous	no	737/ 4453	1.05 (0.93-1.18)		
rs2981582	Parous	yes	4310/27332	1.04 (0.99-1.10)		
rs2981582	Parous (yes/no)	combined	5047/31785		1.00	9.7E-01
rs2981582	Number of births (among parous)	1	1035/ 5461	1.11 (1.00-1.22)		
rs2981582	Number of births (among parous)	2	1938/12159	1.02 (0.95-1.10)		
rs2981582	Number of births (among parous)	3	836/ 5717	1.04 (0.93-1.16)		
rs2981582	Number of births (among parous)	>=4	430/ 3108	0.94 (0.80-1.10)		
rs2981582	Number of births (among parous)	combined	4239/26445		0.96	6.6E-02
rs2981582	Age at first birth (among parous, years)	<20	523/ 2638	1.04 (0.90-1.20)		
rs2981582	Age at first birth (among parous, years)	20-24	1629/10922	1.03 (0.96-1.12)		
rs2981582	Age at first birth (among parous, years)	25-29	1140/ 7909	1.01 (0.91-1.10)		
rs2981582	Age at first birth (among parous, years)	>=30	526/ 3356	1.15 (1.00-1.32)		
rs2981582	Age at first birth (among parous, years/5)	combined	3818/24825		1.02	4.2E-01
rs2981582	Ever breastfed (among parous, yes/no)	no	513/ 2872	1.09 (0.95-1.26)		

SNP	Variable	Stratum	N (cases/ controls)	OR (95% CI)[1]	OR int[2]	P int[3]
rs2981582	Ever breastfed (among parous, yes/no)	yes	1690/10162	1.02 (0.94-1.10)		
rs2981582	Ever breastfed (among parous, yes/no)	combined	2203/13034		0.93	4.0E-01
rs2981582	Usual adult BMI, age<54	<25	847/ 3042	1.06 (0.95-1.19)		
rs2981582	Usual adult BMI, age<54	25-<30	262/ 1039	0.99 (0.81-1.22)		
rs2981582	Usual adult BMI, age<54	>=30	104/ 506	0.97 (0.71-1.35)		
rs2981582	Usual adult BMI (BMI/5), age<54	combined	1213/ 4587		0.95	3.8E-01
rs2981582	Usual adult BMI, age>=54	<25	771/ 6309	1.11 (0.99-1.23)		
rs2981582	Usual adult BMI, age>=54	25-<30	351/ 2739	1.04 (0.88-1.22)		
rs2981582	Usual adult BMI, age>=54	>=30	115/ 950	0.83 (0.61-1.12)		
rs2981582	Usual adult BMI (BMI/5), age>=54	combined	1237/ 9998		0.97	5.3E-01
rs2981582	Usual adult height (cm)	<160	858/ 5459	1.01 (0.91-1.12)		
rs2981582	Usual adult height (cm)	160-<165	1046/ 6538	1.01 (0.92-1.12)		
rs2981582	Usual adult height (cm)	165-<170	907/ 5400	1.04 (0.94-1.16)		
rs2981582	Usual adult height (cm)	>=170	628/ 3618	1.14 (1.00-1.30)		
rs2981582	Usual adult height (cm/5)	combined	3439/21015		1.04	9.9E-02
rs2981582	Ever use of oral contraceptives	no	1250/ 8278	1.05 (0.96-1.15)		
rs2981582	Ever use of oral contraceptives	yes	1625/11154	1.04 (0.96-1.12)		
rs2981582	Ever use of oral contraceptives (yes/no)	combined	2875/19432		0.99	8.5E-01
rs2981582	Duration of oral contraceptive use (years)	0	1250/ 8278	1.05 (0.96-1.15)		
rs2981582	Duration of oral contraceptive use (years)	>0-<5	507/ 3815	0.97 (0.85-1.12)		
rs2981582	Duration of oral contraceptive use (years)	5-<10	398/ 2739	1.15 (0.98-1.34)		
rs2981582	Duration of oral contraceptive use (years)	>=10	686/ 4299	1.05 (0.93-1.18)		
rs2981582	Duration of oral contraceptive use (years/5)	combined	2841/19131		1.01	7.5E-01
rs2981582	Current use of combined estrogen/ progestagen MHT	never	579/ 4470	1.05 (0.93-1.20)		
rs2981582	Current use of combined estrogen/ progestagen MHT	EPCurrent	148/ 1320	1.16 (0.91-1.49)		
rs2981582	Current use of combined estrogen/ progestagen MHT (yes/no)	combined	1040/ 8831		1.12	4.1E-01
rs2981582	Current use of estrogen only MHT	never	602/ 4558	1.05 (0.93-1.19)		
rs2981582	Current use of estrogen only MHT	ECurrent	86/ 1039	1.09 (0.78-1.52)		
rs2981582	Current use of estrogen only MHT (yes/no)	combined	1080/ 9021		1.04	8.4E-01
rs2981582	Duration of combined estrogen/progestagen MHT among current users (years)	never	579/ 4470	1.05 (0.93-1.20)		
rs2981582	Duration of combined estrogen/progestagen MHT among current users (years)	>0-<5	39/ 369	1.05 (0.63-1.75)		
rs2981582	Duration of combined estrogen/progestagen MHT among current users (years)	5-<10	51/ 397	1.15 (0.75-1.77)		
rs2981582	Duration of combined estrogen/progestagen MHT among current users (years)	>=10	50/ 496	1.40 (0.93-2.12)		
rs2981582	Duration of combined estrogen/progestagen MHT among current users (years/5)	combined	1030/ 8736		1.08	2.3E-01
rs2981582	Duration of estrogen only MHT among current users (years)	never	602/ 4558	1.05 (0.93-1.19)		
rs2981582	Duration of estrogen only MHT among current users (years)	>0-<5	27/ 295	1.15 (0.61-2.15)		
rs2981582	Duration of estrogen only MHT among current users (years)	5-<10	30/ 282	1.13 (0.64-1.99)		
rs2981582	Duration of estrogen only MHT among current users (years)	>=10	28/ 423	0.95 (0.54-1.67)		
rs2981582	Duration of estrogen only MHT among current users (years/5)	combined	1075/ 8905		0.98	8.0E-01
rs2981582	Mean lifetime intake of alcohol (g/day)	0	625/ 2986	0.99 (0.87-1.12)		
rs2981582	Mean lifetime intake of alcohol (g/day)	>0-<10	684/ 4796	1.08 (0.96-1.22)		
rs2981582	Mean lifetime intake of alcohol (g/day)	>=10-<20	128/ 1086	0.87 (0.66-1.14)		
rs2981582	Mean lifetime intake of alcohol (g/day)	>=20	107/ 766	1.26 (0.94-1.68)		
rs2981582	Mean lifetime intake of alcohol (10g/day)	combined	1544/ 9634		1.04	2.0E-01
rs2981582	Smoking (ever)	no	1766/10701	1.05 (0.97-1.13)		
rs2981582	Smoking (ever)	yes	1492/ 8723	1.02 (0.94-1.10)		
rs2981582	Smoking (ever/never)	combined	3258/19424		0.97	5.8E-01

SNP	Variable	Stratum	N (cases/ controls)	OR (95% CI)[1]	OR int[2]	P int[3]
rs2981582	Smoking (pack-years)	0	1638/ 9508	1.06 (0.98-1.14)		
rs2981582	Smoking (pack-years)	0-<10	618/ 3747	0.99 (0.87-1.13)		
rs2981582	Smoking (pack-years)	10-<20	315/ 1650	1.06 (0.88-1.27)		
rs2981582	Smoking (pack-years)	>=20	317/ 2170	1.02 (0.85-1.22)		
rs2981582	Smoking (pack-years/10)	combined	2888/17075		1.02	5.7E-01
rs2981582	Physical activity during year before reference date (h/week)	0	112/ 1069	0.90 (0.67-1.22)		
rs2981582	Physical activity during year before reference date (h/week)	0-<3.5	337/ 2820	1.03 (0.87-1.21)		
rs2981582	Physical activity during year before reference date (h/week)	3.5-<7	244/ 2175	1.09 (0.90-1.32)		
rs2981582	Physical activity during year before reference date (h/week)	>=7	327/ 3491	1.04 (0.88-1.23)		
rs2981582	Physical activity during year before reference date (square root of h/week)	combined	1020/ 9555		1.02	5.2E-01
rs3803662	Age at menarche (years)	<=11	617/ 3391	1.14 (1.00-1.31)		
rs3803662	Age at menarche (years)	12-13	1887/10489	1.19 (1.09-1.29)		
rs3803662	Age at menarche (years)	>=14	1539/ 9962	1.21 (1.11-1.32)		
rs3803662	Age at menarche (years/2)	combined	4043/23842		1.04	2.6E-01
rs3803662	Parous	no	670/ 3545	1.40 (1.22-1.60)		
rs3803662	Parous	yes	3924/23652	1.15 (1.09-1.21)		
rs3803662	Parous (yes/no)	combined	4594/27197		0.82	8.0E-03
rs3803662	Number of births (among parous)	1	911/ 4601	1.12 (1.00-1.26)		
rs3803662	Number of births (among parous)	2	1776/10467	1.12 (1.04-1.22)		
rs3803662	Number of births (among parous)	3	767/ 5009	1.21 (1.07-1.36)		
rs3803662	Number of births (among parous)	>=4	401/ 2729	1.26 (1.06-1.48)		
rs3803662	Number of births (among parous)	combined	3855/22806		1.02	3.7E-01
rs3803662	Age at first birth (among parous, years)	<20	488/ 2278	0.95 (0.81-1.12)		
rs3803662	Age at first birth (among parous, years)	20-24	1511/ 9459	1.23 (1.12-1.34)		
rs3803662	Age at first birth (among parous, years)	25-29	1042/ 6675	1.11 (1.00-1.23)		
rs3803662	Age at first birth (among parous, years)	>=30	478/ 2771	1.19 (1.02-1.39)		
rs3803662	Age at first birth (among parous, years/5)	combined	3519/21183		1.02	5.1E-01
rs3803662	Ever breastfed (among parous, yes/no)	no	459/ 2484	1.17 (1.00-1.38)		
rs3803662	Ever breastfed (among parous, yes/no)	yes	1551/ 8636	1.19 (1.09-1.30)		
rs3803662	Ever breastfed (among parous, yes/no)	combined	2010/11120		1.01	8.8E-01
rs3803662	Usual adult BMI, age<54	<25	803/ 2793	1.21 (1.07-1.38)		
rs3803662	Usual adult BMI, age<54	25-<30	255/ 955	1.15 (0.92-1.44)		
rs3803662	Usual adult BMI, age<54	>=30	99/ 467	1.14 (0.80-1.62)		
rs3803662	Usual adult BMI (BMI/5), age<54	combined	1157/ 4215		0.99	8.2E-01
rs3803662	Usual adult BMI, age>=54	<25	650/ 4873	1.26 (1.11-1.43)		
rs3803662	Usual adult BMI, age>=54	25-<30	309/ 2338	1.03 (0.85-1.25)		
rs3803662	Usual adult BMI, age>=54	>=30	106/ 834	1.21 (0.87-1.66)		
rs3803662	Usual adult BMI (BMI/5), age>=54	combined	1065/ 8045		0.94	2.8E-01
rs3803662	Usual adult height (cm)	<160	792/ 4876	1.24 (1.10-1.39)		
rs3803662	Usual adult height (cm)	160-<165	960/ 5547	1.20 (1.08-1.34)		
rs3803662	Usual adult height (cm)	165-<170	804/ 4423	1.16 (1.03-1.31)		
rs3803662	Usual adult height (cm)	>=170	556/ 2844	1.27 (1.09-1.47)		
rs3803662	Usual adult height (cm/5)	combined	3112/17690		1.01	8.0E-01
rs3803662	Ever use of oral contraceptives	no	1182/ 7377	1.19 (1.08-1.31)		
rs3803662	Ever use of oral contraceptives	yes	1435/ 8827	1.19 (1.09-1.30)		
rs3803662	Ever use of oral contraceptives (yes/no)	combined	2617/16204		1.00	9.9E-01
rs3803662	Duration of oral contraceptive use (years)	0	1182/ 7377	1.19 (1.08-1.31)		
rs3803662	Duration of oral contraceptive use (years)	>0-<5	455/ 3027	1.21 (1.04-1.42)		
rs3803662	Duration of oral contraceptive use (years)	5-<10	360/ 2174	1.10 (0.92-1.32)		

SNP	Variable	Stratum	N (cases/ controls)	OR (95% CI)[1]	OR int[2]	P int[3]
rs3803662	Duration of oral contraceptive use (years)	>=10	587/ 3353	1.20 (1.04-1.38)		
rs3803662	Duration of oral contraceptive use (years/5)	combined	2584/15931		1.01	6.9E-01
rs3803662	Current use of combined estrogen/ progestagen MHT	never	508/ 3756	1.26 (1.09-1.46)		
rs3803662	Current use of combined estrogen/ progestagen MHT	EPCurrent	121/ 924	1.02 (0.76-1.37)		
rs3803662	Current use of combined estrogen/ progestagen MHT (yes/no)	combined	867/ 6921		0.85	3.1E-01
rs3803662	Current use of estrogen only MHT	never	532/ 3829	1.30 (1.13-1.50)		
rs3803662	Current use of estrogen only MHT	ECurrent	72/ 798	1.17 (0.79-1.74)		
rs3803662	Current use of estrogen only MHT (yes/no)	combined	910/ 7082		0.99	9.6E-01
rs3803662	Duration of combined estrogen/progestagen MHT among current users (years)	never	508/ 3756	1.26 (1.09-1.46)		
rs3803662	Duration of combined estrogen/progestagen MHT among current users (years)	>0-<5	33/ 293	0.70 (0.38-1.27)		
rs3803662	Duration of combined estrogen/progestagen MHT among current users (years)	5-<10	45/ 281	1.23 (0.77-1.96)		
rs3803662	Duration of combined estrogen/progestagen MHT among current users (years)	>=10	35/ 299	1.04 (0.59-1.82)		
rs3803662	Duration of combined estrogen/progestagen MHT among current users (years/5)	combined	857/ 6836		0.94	4.5E-01
rs3803662	Duration of estrogen only MHT among current users (years)	never	532/ 3829	1.30 (1.13-1.50)		
rs3803662	Duration of estrogen only MHT among current users (years)	>0-<5	24/ 235	1.39 (0.70-2.77)		
rs3803662	Duration of estrogen only MHT among current users (years)	5-<10	26/ 214	1.18 (0.62-2.25)		
rs3803662	Duration of estrogen only MHT among current users (years)	>=10	21/ 314	0.69 (0.31-1.54)		
rs3803662	Duration of estrogen only MHT among current users (years/5)	combined	906/ 6976		0.91	3.6E-01
rs3803662	Mean lifetime intake of alcohol (g/day)	0	603/ 2784	1.17 (1.02-1.35)		
rs3803662	Mean lifetime intake of alcohol (g/day)	>0-<10	596/ 3707	1.27 (1.11-1.45)		
rs3803662	Mean lifetime intake of alcohol (g/day)	>=10-<20	115/ 773	0.98 (0.71-1.34)		
rs3803662	Mean lifetime intake of alcohol (g/day)	>=20	95/ 544	1.26 (0.90-1.76)		
rs3803662	Mean lifetime intake of alcohol (10g/day)	combined	1409/ 7808		0.99	7.8E-01
rs3803662	Smoking (ever)	no	1584/ 8933	1.27 (1.16-1.38)		
rs3803662	Smoking (ever)	yes	1341/ 7215	1.16 (1.06-1.28)		
rs3803662	Smoking (ever/never)	combined	2925/16148		0.92	1.9E-01
rs3803662	Smoking (pack-years)	0	1454/ 7745	1.28 (1.17-1.40)		
rs3803662	Smoking (pack-years)	0-<10	575/ 3076	1.21 (1.05-1.40)		
rs3803662	Smoking (pack-years)	10-<20	280/ 1332	1.22 (0.99-1.50)		
rs3803662	Smoking (pack-years)	>=20	279/ 1706	1.02 (0.83-1.26)		
rs3803662	Smoking (pack-years/10)	combined	2588/13859		0.96	2.1E-01
rs3803662	Physical activity during year before reference date (h/week)	0	111/ 1055	1.48 (1.07-2.03)		
rs3803662	Physical activity during year before reference date (h/week)	0-<3.5	268/ 2477	1.43 (1.18-1.74)		
rs3803662	Physical activity during year before reference date (h/week)	3.5-<7	182/ 1704	1.03 (0.82-1.31)		
rs3803662	Physical activity during year before reference date (h/week)	>=7	227/ 2104	1.12 (0.91-1.39)		
rs3803662	Physical activity during year before reference date (square root of h/week)	combined	788/ 7340		0.95	2.6E-01
rs3817198	Age at menarche (years)	<=11	618/ 3338	1.04 (0.91-1.19)		
rs3817198	Age at menarche (years)	12-13	1886/10153	1.14 (1.06-1.24)		
rs3817198	Age at menarche (years)	>=14	1537/ 9624	1.00 (0.92-1.09)		
rs3817198	Age at menarche (years/2)	combined	4041/23115		0.96	2.5E-01
rs3817198	Parous	no	680/ 3435	1.09 (0.96-1.24)		
rs3817198	Parous	yes	3981/23015	1.07 (1.01-1.13)		
rs3817198	Parous (yes/no)	combined	4661/26450		0.98	8.1E-01
rs3817198	Number of births (among parous)	1	936/ 4464	0.98 (0.87-1.10)		
rs3817198	Number of births (among parous)	2	1800/10234	1.05 (0.97-1.14)		
rs3817198	Number of births (among parous)	3	780/ 4821	1.13 (1.00-1.27)		

SNP	Variable	Stratum	N (cases/ controls)	OR (95% CI)[1]	OR int[2]	P int[3]
rs3817198	Number of births (among parous)	>=4	416/ 2632	1.26 (1.06-1.49)		
rs3817198	Number of births (among parous)	combined	3932/22151		1.07	5.7E-03
rs3817198	Age at first birth (among parous, years)	<20	488/ 2202	1.15 (0.98-1.35)		
rs3817198	Age at first birth (among parous, years)	20-24	1510/ 9203	1.06 (0.97-1.16)		
rs3817198	Age at first birth (among parous, years)	25-29	1037/ 6484	1.02 (0.91-1.13)		
rs3817198	Age at first birth (among parous, years)	>=30	476/ 2691	1.07 (0.91-1.25)		
rs3817198	Age at first birth (among parous, years/5)	combined	3511/20580		0.96	2.6E-01
rs3817198	Ever breastfed (among parous, yes/no)	no	459/ 2499	1.07 (0.91-1.26)		
rs3817198	Ever breastfed (among parous, yes/no)	yes	1535/ 8692	1.10 (1.01-1.19)		
rs3817198	Ever breastfed (among parous, yes/no)	combined	1994/11191		1.02	8.4E-01
rs3817198	Usual adult BMI, age<54	<25	794/ 2813	1.05 (0.93-1.19)		
rs3817198	Usual adult BMI, age<54	25-<30	252/ 965	1.09 (0.88-1.37)		
rs3817198	Usual adult BMI, age<54	>=30	92/ 466	0.99 (0.69-1.43)		
rs3817198	Usual adult BMI (BMI/5), age<54	combined	1138/ 4244		0.99	9.2E-01
rs3817198	Usual adult BMI, age>=54	<25	652/ 4886	1.09 (0.96-1.24)		
rs3817198	Usual adult BMI, age>=54	25-<30	307/ 2354	1.12 (0.93-1.34)		
rs3817198	Usual adult BMI, age>=54	>=30	107/ 841	1.19 (0.86-1.63)		
rs3817198	Usual adult BMI (BMI/5), age>=54	combined	1066/ 8081		1.01	9.2E-01
rs3817198	Usual adult height (cm)	<160	785/ 4746	1.15 (1.01-1.29)		
rs3817198	Usual adult height (cm)	160-<165	954/ 5363	0.97 (0.87-1.09)		
rs3817198	Usual adult height (cm)	165-<170	795/ 4235	1.12 (0.99-1.26)		
rs3817198	Usual adult height (cm)	>=170	550/ 2686	1.04 (0.90-1.21)		
rs3817198	Usual adult height (cm/5)	combined	3084/17030		0.98	4.9E-01
rs3817198	Ever use of oral contraceptives	no	1186/ 7403	1.01 (0.91-1.11)		
rs3817198	Ever use of oral contraceptives	yes	1414/ 8856	1.09 (1.00-1.19)		
rs3817198	Ever use of oral contraceptives (yes/no)	combined	2600/16259		1.08	2.4E-01
rs3817198	Duration of oral contraceptive use (years)	0	1186/ 7403	1.01 (0.91-1.11)		
rs3817198	Duration of oral contraceptive use (years)	>0-<5	452/ 3047	1.25 (1.07-1.45)		
rs3817198	Duration of oral contraceptive use (years)	5-<10	352/ 2176	0.98 (0.82-1.18)		
rs3817198	Duration of oral contraceptive use (years)	>=10	578/ 3361	1.00 (0.87-1.14)		
rs3817198	Duration of oral contraceptive use (years/5)	combined	2568/15987		0.98	4.5E-01
rs3817198	Current use of combined estrogen/ progestagen MHT	never	508/ 3759	1.07 (0.93-1.24)		
rs3817198	Current use of combined estrogen/ progestagen MHT	EPCurrent	121/ 928	0.91 (0.68-1.23)		
rs3817198	Current use of combined estrogen/ progestagen MHT (yes/no)	combined	868/ 6942		0.85	3.3E-01
rs3817198	Current use of estrogen only MHT	never	532/ 3836	1.09 (0.94-1.25)		
rs3817198	Current use of estrogen only MHT	ECurrent	72/ 808	1.31 (0.91-1.89)		
rs3817198	Current use of estrogen only MHT (yes/no)	combined	912/ 7105		1.26	2.3E-01
rs3817198	Duration of combined estrogen/progestagen MHT among current users (years)	never	508/ 3759	1.07 (0.93-1.24)		
rs3817198	Duration of combined estrogen/progestagen MHT among current users (years)	>0-<5	33/ 295	1.00 (0.56-1.79)		
rs3817198	Duration of combined estrogen/progestagen MHT among current users (years)	5-<10	45/ 282	1.08 (0.69-1.72)		
rs3817198	Duration of combined estrogen/progestagen MHT among current users (years)	>=10	35/ 300	0.82 (0.46-1.48)		
rs3817198	Duration of combined estrogen/progestagen MHT among current users (years/5)	combined	858/ 6857		0.97	7.1E-01
rs3817198	Duration of estrogen only MHT among current users (years)	never	532/ 3836	1.09 (0.94-1.25)		
rs3817198	Duration of estrogen only MHT among current users (years)	>0-<5	24/ 236	1.55 (0.84-2.84)		
rs3817198	Duration of estrogen only MHT among current users (years)	5-<10	26/ 216	1.40 (0.75-2.60)		
rs3817198	Duration of estrogen only MHT among current users (years)	>=10	21/ 320	1.13 (0.56-2.29)		
rs3817198	Duration of estrogen only MHT among current users (years/5)	combined	908/ 6998		1.12	2.6E-01

SNP	Variable	Stratum	N (cases/ controls)	OR (95% CI)[1]	OR int[2]	P int[3]
rs3817198	Mean lifetime intake of alcohol (g/day)	0	607/ 2796	1.02 (0.89-1.17)		
rs3817198	Mean lifetime intake of alcohol (g/day)	>0-<10	597/ 3712	1.12 (0.98-1.27)		
rs3817198	Mean lifetime intake of alcohol (g/day)	>=10-<20	116/ 777	1.03 (0.75-1.40)		
rs3817198	Mean lifetime intake of alcohol (g/day)	>=20	94/ 545	0.93 (0.67-1.29)		
rs3817198	Mean lifetime intake of alcohol (10g/day)	combined	1414/ 7830		1.01	8.4E-01
rs3817198	Smoking (ever)	no	1561/ 8570	1.07 (0.98-1.17)		
rs3817198	Smoking (ever)	yes	1334/ 6964	1.08 (0.98-1.18)		
rs3817198	Smoking (ever/never)	combined	2895/15534		1.00	9.9E-01
rs3817198	Smoking (pack-years)	0	1431/ 7378	1.08 (0.99-1.19)		
rs3817198	Smoking (pack-years)	0-<10	571/ 2975	1.11 (0.96-1.28)		
rs3817198	Smoking (pack-years)	10-<20	280/ 1283	1.00 (0.80-1.23)		
rs3817198	Smoking (pack-years)	>=20	274/ 1663	1.24 (1.01-1.52)		
rs3817198	Smoking (pack-years/10)	combined	2556/13299		1.03	3.1E-01
rs3817198	Physical activity during year before reference date (h/week)	0	111/ 1061	1.01 (0.75-1.38)		
rs3817198	Physical activity during year before reference date (h/week)	0-<3.5	269/ 2492	0.96 (0.79-1.17)		
rs3817198	Physical activity during year before reference date (h/week)	3.5-<7	182/ 1709	0.95 (0.75-1.21)		
rs3817198	Physical activity during year before reference date (h/week)	>=7	227/ 2106	1.10 (0.89-1.35)		
rs3817198	Physical activity during year before reference date (square root of h/week)	combined	789/ 7368		1.03	5.3E-01
rs4973768	Age at menarche (years)	<=11	661/ 3680	1.02 (0.90-1.15)		
rs4973768	Age at menarche (years)	12-13	1985/12211	1.02 (0.95-1.10)		
rs4973768	Age at menarche (years)	>=14	1656/12465	1.02 (0.95-1.10)		
rs4973768	Age at menarche (years/2)	combined	4302/28356		0.97	3.6E-01
rs4973768	Parous	no	723/ 4238	1.07 (0.95-1.20)		
rs4973768	Parous	yes	4229/27677	1.03 (0.99-1.09)		
rs4973768	Parous (yes/no)	combined	4952/31915		0.97	6.1E-01
rs4973768	Number of births (among parous)	1	1018/ 5633	1.05 (0.95-1.16)		
rs4973768	Number of births (among parous)	2	1928/12765	1.03 (0.96-1.10)		
rs4973768	Number of births (among parous)	3	808/ 5653	1.06 (0.95-1.18)		
rs4973768	Number of births (among parous)	>=4	404/ 2815	0.98 (0.84-1.14)		
rs4973768	Number of births (among parous)	combined	4158/26866		0.98	3.0E-01
rs4973768	Age at first birth (among parous, years)	<20	512/ 2811	1.01 (0.88-1.16)		
rs4973768	Age at first birth (among parous, years)	20-24	1623/11142	0.99 (0.91-1.07)		
rs4973768	Age at first birth (among parous, years)	25-29	1135/ 8223	1.09 (0.99-1.19)		
rs4973768	Age at first birth (among parous, years)	>=30	545/ 3538	1.17 (1.02-1.34)		
rs4973768	Age at first birth (among parous, years/5)	combined	3815/25714		1.07	2.0E-02
rs4973768	Ever breastfed (among parous, yes/no)	no	499/ 2865	0.98 (0.86-1.13)		
rs4973768	Ever breastfed (among parous, yes/no)	yes	1700/10281	1.05 (0.98-1.13)		
rs4973768	Ever breastfed (among parous, yes/no)	combined	2199/13146		1.07	4.1E-01
rs4973768	Usual adult BMI, age<54	<25	877/ 3090	1.03 (0.92-1.15)		
rs4973768	Usual adult BMI, age<54	25-<30	277/ 1042	0.98 (0.80-1.19)		
rs4973768	Usual adult BMI, age<54	>=30	113/ 519	1.19 (0.88-1.61)		
rs4973768	Usual adult BMI (BMI/5), age<54	combined	1267/ 4651		1.02	7.0E-01
rs4973768	Usual adult BMI, age>=54	<25	757/ 6445	0.97 (0.87-1.08)		
rs4973768	Usual adult BMI, age>=54	25-<30	340/ 2743	1.01 (0.86-1.18)		
rs4973768	Usual adult BMI, age>=54	>=30	109/ 956	1.02 (0.77-1.34)		
rs4973768	Usual adult BMI (BMI/5), age>=54	combined	1206/10144		1.00	9.3E-01
rs4973768	Usual adult height (cm)	<160	821/ 5110	1.10 (0.99-1.23)		
rs4973768	Usual adult height (cm)	160-<165	1023/ 6190	0.93 (0.84-1.03)		
rs4973768	Usual adult height (cm)	165-<170	875/ 5106	1.08 (0.97-1.20)		

SNP	Variable	Stratum	N (cases/ controls)	OR (95% CI)[1]	OR int[2]	P int[3]
rs4973768	Usual adult height (cm)	>=170	631/ 3415	1.07 (0.95-1.21)		
rs4973768	Usual adult height (cm/5)	combined	3350/19821		1.00	8.8E-01
rs4973768	Ever use of oral contraceptives	no	1208/ 7878	0.97 (0.89-1.06)		
rs4973768	Ever use of oral contraceptives	yes	1610/10403	1.08 (1.00-1.16)		
rs4973768	Ever use of oral contraceptives (yes/no)	combined	2818/18281		1.11	7.9E-02
rs4973768	Duration of oral contraceptive use (years)	0	1208/ 7878	0.97 (0.89-1.06)		
rs4973768	Duration of oral contraceptive use (years)	>0-<5	500/ 3464	0.99 (0.86-1.13)		
rs4973768	Duration of oral contraceptive use (years)	5-<10	396/ 2582	1.14 (0.97-1.33)		
rs4973768	Duration of oral contraceptive use (years)	>=10	685/ 4087	1.10 (0.98-1.24)		
rs4973768	Duration of oral contraceptive use (years/5)	combined	2789/18011		1.04	9.0E-02
rs4973768	Current use of combined estrogen/ progestagen MHT	never	566/ 4500	0.92 (0.81-1.04)		
rs4973768	Current use of combined estrogen/ progestagen MHT	EPCurrent	150/ 1368	0.96 (0.75-1.22)		
rs4973768	Current use of combined estrogen/ progestagen MHT (yes/no)	combined	1029/ 9007		1.01	9.4E-01
rs4973768	Current use of estrogen only MHT	never	590/ 4577	0.93 (0.82-1.05)		
rs4973768	Current use of estrogen only MHT	ECurrent	86/ 1063	1.06 (0.78-1.44)		
rs4973768	Current use of estrogen only MHT (yes/no)	combined	1074/ 9189		1.12	5.0E-01
rs4973768	Duration of combined estrogen/progestagen MHT among current users (years)	never	566/ 4500	0.92 (0.81-1.04)		
rs4973768	Duration of combined estrogen/progestagen MHT among current users (years)	>0-<5	38/ 374	0.96 (0.58-1.58)		
rs4973768	Duration of combined estrogen/progestagen MHT among current users (years)	5-<10	52/ 412	1.10 (0.73-1.64)		
rs4973768	Duration of combined estrogen/progestagen MHT among current users (years)	>=10	52/ 524	0.80 (0.53-1.21)		
rs4973768	Duration of combined estrogen/progestagen MHT among current users (years/5)	combined	1019/ 8912		0.99	8.5E-01
rs4973768	Duration of estrogen only MHT among current users (years)	never	590/ 4577	0.93 (0.82-1.05)		
rs4973768	Duration of estrogen only MHT among current users (years)	>0-<5	26/ 303	1.09 (0.62-1.93)		
rs4973768	Duration of estrogen only MHT among current users (years)	5-<10	29/ 288	0.99 (0.59-1.68)		
rs4973768	Duration of estrogen only MHT among current users (years)	>=10	30/ 433	1.12 (0.67-1.87)		
rs4973768	Duration of estrogen only MHT among current users (years/5)	combined	1069/ 9072		1.05	5.2E-01
rs4973768	Mean lifetime intake of alcohol (g/day)	0	607/ 2940	0.95 (0.84-1.08)		
rs4973768	Mean lifetime intake of alcohol (g/day)	>0-<10	682/ 4912	1.06 (0.94-1.18)		
rs4973768	Mean lifetime intake of alcohol (g/day)	>=10-<20	131/ 1127	1.02 (0.78-1.32)		
rs4973768	Mean lifetime intake of alcohol (g/day)	>=20	107/ 787	1.18 (0.89-1.57)		
rs4973768	Mean lifetime intake of alcohol (10g/day)	combined	1527/ 9766		1.06	6.7E-02
rs4973768	Smoking (ever)	no	1709/ 9880	0.99 (0.92-1.07)		
rs4973768	Smoking (ever)	yes	1454/ 8383	1.06 (0.97-1.15)		
rs4973768	Smoking (ever/never)	combined	3163/18263		1.06	2.7E-01
rs4973768	Smoking (pack-years)	0	1637/ 9028	1.01 (0.93-1.09)		
rs4973768	Smoking (pack-years)	0-<10	625/ 3647	1.00 (0.88-1.13)		
rs4973768	Smoking (pack-years)	10-<20	310/ 1620	1.05 (0.88-1.27)		
rs4973768	Smoking (pack-years)	>=20	322/ 2137	1.19 (1.00-1.41)		
rs4973768	Smoking (pack-years/10)	combined	2894/16432		1.07	1.5E-02
rs4973768	Physical activity during year before reference date (h/week)	0	112/ 1072	1.10 (0.83-1.45)		
rs4973768	Physical activity during year before reference date (h/week)	0-<3.5	320/ 2807	1.06 (0.90-1.26)		
rs4973768	Physical activity during year before reference date (h/week)	3.5-<7	239/ 2211	0.87 (0.71-1.05)		
rs4973768	Physical activity during year before reference date (h/week)	>=7	345/ 3683	1.15 (0.98-1.34)		
rs4973768	Physical activity during year before reference date (square root of h/week)	combined	1016/ 9773		1.00	9.7E-01
rs614367	Age at menarche (years)	<=11	420/ 2358	1.07 (0.87-1.31)		
rs614367	Age at menarche (years)	12-13	1452/ 8055	1.14 (1.02-1.27)		

SNP	Variable	Stratum	N (cases/ controls)	OR (95% CI)[1]	OR int[2]	P int[3]
rs614367	Age at menarche (years)	>=14	1254/ 7651	0.94 (0.84-1.06)		
rs614367	Age at menarche (years/2)	combined	3126/18064		0.94	1.7E-01
rs614367	Parous	no	576/ 3050	0.94 (0.79-1.13)		
rs614367	Parous	yes	3123/18334	1.04 (0.97-1.13)		
rs614367	Parous (yes/no)	combined	3699/21384		1.10	3.2E-01
rs614367	Number of births (among parous)	1	820/ 3980	1.02 (0.88-1.19)		
rs614367	Number of births (among parous)	2	1338/ 7768	1.06 (0.94-1.19)		
rs614367	Number of births (among parous)	3	581/ 3706	1.04 (0.87-1.24)		
rs614367	Number of births (among parous)	>=4	319/ 2015	1.04 (0.82-1.31)		
rs614367	Number of births (among parous)	combined	3058/17469		0.99	6.9E-01
rs614367	Age at first birth (among parous, years)	<20	333/ 1857	1.37 (1.10-1.70)		
rs614367	Age at first birth (among parous, years)	20-24	1133/ 6999	1.06 (0.94-1.20)		
rs614367	Age at first birth (among parous, years)	25-29	808/ 4913	1.02 (0.87-1.18)		
rs614367	Age at first birth (among parous, years)	>=30	392/ 2093	0.91 (0.73-1.14)		
rs614367	Age at first birth (among parous, years/5)	combined	2666/15862		0.90	1.5E-02
rs614367	Ever breastfed (among parous, yes/no)	no	502/ 2304	1.32 (1.10-1.57)		
rs614367	Ever breastfed (among parous, yes/no)	yes	1607/ 9427	0.97 (0.87-1.07)		
rs614367	Ever breastfed (among parous, yes/no)	combined	2109/11731		0.73	3.0E-03
rs614367	Usual adult BMI, age<54	<25	838/ 2720	1.02 (0.88-1.19)		
rs614367	Usual adult BMI, age<54	25-<30	261/ 880	0.98 (0.75-1.28)		
rs614367	Usual adult BMI, age<54	>=30	114/ 418	1.41 (0.97-2.06)		
rs614367	Usual adult BMI (BMI/5), age<54	combined	1213/ 4018		1.14	6.1E-02
rs614367	Usual adult BMI, age>=54	<25	730/ 5948	1.05 (0.91-1.22)		
rs614367	Usual adult BMI, age>=54	25-<30	341/ 2494	1.07 (0.86-1.33)		
rs614367	Usual adult BMI, age>=54	>=30	114/ 811	1.05 (0.72-1.55)		
rs614367	Usual adult BMI (BMI/5), age>=54	combined	1185/ 9253		0.99	9.2E-01
rs614367	Usual adult height (cm)	<160	814/ 4895	1.18 (1.02-1.36)		
rs614367	Usual adult height (cm)	160-<165	981/ 5892	0.92 (0.80-1.05)		
rs614367	Usual adult height (cm)	165-<170	874/ 4851	1.05 (0.91-1.22)		
rs614367	Usual adult height (cm)	>=170	608/ 3204	1.05 (0.89-1.25)		
rs614367	Usual adult height (cm/5)	combined	3277/18842		0.99	8.1E-01
rs614367	Ever use of oral contraceptives	no	1151/ 7377	1.00 (0.89-1.13)		
rs614367	Ever use of oral contraceptives	yes	1582/10179	1.08 (0.98-1.20)		
rs614367	Ever use of oral contraceptives (yes/no)	combined	2733/17556		1.08	3.2E-01
rs614367	Duration of oral contraceptive use (years)	0	1151/ 7377	1.00 (0.89-1.13)		
rs614367	Duration of oral contraceptive use (years)	>0-<5	484/ 3377	1.08 (0.90-1.30)		
rs614367	Duration of oral contraceptive use (years)	5-<10	404/ 2481	1.10 (0.89-1.35)		
rs614367	Duration of oral contraceptive use (years)	>=10	665/ 4044	1.08 (0.92-1.28)		
rs614367	Duration of oral contraceptive use (years/5)	combined	2704/17279		1.03	3.0E-01
rs614367	Current use of combined estrogen/ progestagen MHT	never	546/ 4073	1.05 (0.89-1.25)		
rs614367	Current use of combined estrogen/ progestagen MHT	EPCurrent	139/ 1189	1.23 (0.89-1.71)		
rs614367	Current use of combined estrogen/ progestagen MHT (yes/no)	combined	989/ 8117		1.19	3.4E-01
rs614367	Current use of estrogen only MHT	never	570/ 4143	1.07 (0.91-1.26)		
rs614367	Current use of estrogen only MHT	ECurrent	85/ 916	1.24 (0.82-1.86)		
rs614367	Current use of estrogen only MHT (yes/no)	combined	1032/ 8280		1.19	4.3E-01
rs614367	Duration of combined estrogen/progestagen MHT among current users (years)	never	546/ 4073	1.05 (0.89-1.25)		
rs614367	Duration of combined estrogen/progestagen MHT among current users (years)	>0-<5	36/ 314	1.69 (0.85-3.34)		
rs614367	Duration of combined estrogen/progestagen MHT among current users (years)	5-<10	47/ 353	1.56 (0.93-2.63)		

SNP	Variable	Stratum	N (cases/ controls)	OR (95% CI)[1]	OR int[2]	P int[3]
rs614367	Duration of combined estrogen/progestagen MHT among current users (years)	>=10	48/ 464	0.78 (0.42-1.47)		
rs614367	Duration of combined estrogen/progestagen MHT among current users (years/5)	combined	980/ 8022		0.98	8.3E-01
rs614367	Duration of estrogen only MHT among current users (years)	never	570/ 4143	1.07 (0.91-1.26)		
rs614367	Duration of estrogen only MHT among current users (years)	>0-<5	27/ 280	1.23 (0.62-2.46)		
rs614367	Duration of estrogen only MHT among current users (years)	5-<10	30/ 255	1.09 (0.51-2.35)		
rs614367	Duration of estrogen only MHT among current users (years)	>=10	27/ 358	1.28 (0.64-2.58)		
rs614367	Duration of estrogen only MHT among current users (years/5)	combined	1028/ 8205		1.07	4.6E-01
rs614367	Mean lifetime intake of alcohol (g/day)	0	551/ 2800	1.05 (0.89-1.23)		
rs614367	Mean lifetime intake of alcohol (g/day)	>0-<10	639/ 4659	1.08 (0.92-1.26)		
rs614367	Mean lifetime intake of alcohol (g/day)	>=10-<20	114/ 1060	1.06 (0.72-1.57)		
rs614367	Mean lifetime intake of alcohol (g/day)	>=20	101/ 742	0.95 (0.62-1.46)		
rs614367	Mean lifetime intake of alcohol (10g/day)	combined	1405/ 9261		1.00	9.8E-01
rs614367	Smoking (ever)	no	1700/ 9641	1.02 (0.92-1.13)		
rs614367	Smoking (ever)	yes	1440/ 7791	1.04 (0.94-1.17)		
rs614367	Smoking (ever/never)	combined	3140/17432		1.02	7.5E-01
rs614367	Smoking (pack-years)	0	1575/ 8465	1.02 (0.91-1.13)		
rs614367	Smoking (pack-years)	0-<10	597/ 3368	0.96 (0.81-1.14)		
rs614367	Smoking (pack-years)	10-<20	303/ 1473	1.23 (0.98-1.55)		
rs614367	Smoking (pack-years)	>=20	298/ 1853	1.04 (0.82-1.32)		
rs614367	Smoking (pack-years/10)	combined	2773/15159		1.02	6.2E-01
rs614367	Physical activity during year before reference date (h/week)	0	112/ 680	1.29 (0.88-1.89)		
rs614367	Physical activity during year before reference date (h/week)	0-<3.5	330/ 2410	1.20 (0.95-1.50)		
rs614367	Physical activity during year before reference date (h/week)	3.5-<7	239/ 1969	1.05 (0.80-1.37)		
rs614367	Physical activity during year before reference date (h/week)	>=7	322/ 3344	1.04 (0.83-1.31)		
rs614367	Physical activity during year before reference date (square root of h/week)	combined	1003/ 8403		0.93	2.0E-01
rs6504950	Age at menarche (years)	<=11	649/ 3700	0.90 (0.79-1.04)		
rs6504950	Age at menarche (years)	12-13	2016/12333	1.02 (0.94-1.10)		
rs6504950	Age at menarche (years)	>=14	1683/12711	1.03 (0.95-1.12)		
rs6504950	Age at menarche (years/2)	combined	4348/28744		1.08	2.8E-02
rs6504950	Parous	no	724/ 4200	1.05 (0.92-1.19)		
rs6504950	Parous	yes	4288/27832	1.00 (0.95-1.06)		
rs6504950	Parous (yes/no)	combined	5012/32032		0.96	5.2E-01
rs6504950	Number of births (among parous)	1	1033/ 5733	0.99 (0.89-1.11)		
rs6504950	Number of births (among parous)	2	1946/12902	1.02 (0.95-1.10)		
rs6504950	Number of births (among parous)	3	815/ 5719	0.98 (0.86-1.10)		
rs6504950	Number of births (among parous)	>=4	422/ 2866	0.99 (0.83-1.17)		
rs6504950	Number of births (among parous)	combined	4216/27220		0.99	6.7E-01
rs6504950	Age at first birth (among parous, years)	<20	517/ 2802	0.94 (0.81-1.10)		
rs6504950	Age at first birth (among parous, years)	20-24	1616/11124	1.04 (0.96-1.14)		
rs6504950	Age at first birth (among parous, years)	25-29	1125/ 8202	0.99 (0.89-1.09)		
rs6504950	Age at first birth (among parous, years)	>=30	534/ 3535	0.97 (0.83-1.13)		
rs6504950	Age at first birth (among parous, years/5)	combined	3792/25663		1.01	8.0E-01
rs6504950	Ever breastfed (among parous, yes/no)	no	507/ 2839	1.05 (0.89-1.22)		
rs6504950	Ever breastfed (among parous, yes/no)	yes	1673/10042	1.02 (0.94-1.11)		
rs6504950	Ever breastfed (among parous, yes/no)	combined	2180/12881		0.97	7.8E-01
rs6504950	Usual adult BMI, age<54	<25	834/ 2970	1.03 (0.91-1.17)		
rs6504950	Usual adult BMI, age<54	25-<30	264/ 1001	0.91 (0.72-1.14)		
rs6504950	Usual adult BMI, age<54	>=30	103/ 493	1.11 (0.79-1.56)		

SNP	Variable	Stratum	N (cases/ controls)	OR (95% CI)[1]	OR int[2]	P int[3]
rs6504950	Usual adult BMI (BMI/5), age<54	combined	1201/ 4464		1.01	8.6E-01
rs6504950	Usual adult BMI, age>=54	<25	765/ 6375	1.05 (0.93-1.18)		
rs6504950	Usual adult BMI, age>=54	25-<30	356/ 2689	1.05 (0.88-1.25)		
rs6504950	Usual adult BMI, age>=54	>=30	115/ 907	1.04 (0.76-1.43)		
rs6504950	Usual adult BMI (BMI/5), age>=54	combined	1236/ 9971		1.04	5.2E-01
rs6504950	Usual adult height (cm)	<160	849/ 5173	0.97 (0.86-1.09)		
rs6504950	Usual adult height (cm)	160-<165	1030/ 6257	1.04 (0.93-1.16)		
rs6504950	Usual adult height (cm)	165-<170	884/ 5136	1.04 (0.93-1.17)		
rs6504950	Usual adult height (cm)	>=170	634/ 3404	1.15 (1.00-1.32)		
rs6504950	Usual adult height (cm/5)	combined	3397/19970		1.05	6.5E-02
rs6504950	Ever use of oral contraceptives	no	1225/ 8070	1.01 (0.91-1.11)		
rs6504950	Ever use of oral contraceptives	yes	1603/10355	1.10 (1.01-1.20)		
rs6504950	Ever use of oral contraceptives (yes/no)	combined	2828/18425		1.09	1.8E-01
rs6504950	Duration of oral contraceptive use (years)	0	1225/ 8070	1.01 (0.91-1.11)		
rs6504950	Duration of oral contraceptive use (years)	>0-<5	497/ 3444	1.12 (0.96-1.30)		
rs6504950	Duration of oral contraceptive use (years)	5-<10	389/ 2530	1.14 (0.96-1.35)		
rs6504950	Duration of oral contraceptive use (years)	>=10	686/ 4103	1.06 (0.93-1.20)		
rs6504950	Duration of oral contraceptive use (years/5)	combined	2797/18147		1.01	8.1E-01
rs6504950	Current use of combined estrogen/ progestagen MHT	never	575/ 4514	1.05 (0.92-1.21)		
rs6504950	Current use of combined estrogen/ progestagen MHT	EPCurrent	150/ 1373	0.97 (0.74-1.27)		
rs6504950	Current use of combined estrogen/ progestagen MHT (yes/no)	combined	1034/ 9028		0.92	5.8E-01
rs6504950	Current use of estrogen only MHT	never	599/ 4590	1.07 (0.93-1.22)		
rs6504950	Current use of estrogen only MHT	ECurrent	86/ 1067	0.97 (0.68-1.38)		
rs6504950	Current use of estrogen only MHT (yes/no)	combined	1079/ 9209		0.92	6.6E-01
rs6504950	Duration of combined estrogen/progestagen MHT among current users (years)	never	575/ 4514	1.05 (0.92-1.21)		
rs6504950	Duration of combined estrogen/progestagen MHT among current users (years)	>0-<5	38/ 376	0.69 (0.40-1.19)		
rs6504950	Duration of combined estrogen/progestagen MHT among current users (years)	5-<10	52/ 415	1.00 (0.62-1.61)		
rs6504950	Duration of combined estrogen/progestagen MHT among current users (years)	>=10	52/ 525	1.32 (0.85-2.04)		
rs6504950	Duration of combined estrogen/progestagen MHT among current users (years/5)	combined	1024/ 8934		1.06	3.7E-01
rs6504950	Duration of estrogen only MHT among current users (years)	never	599/ 4590	1.07 (0.93-1.22)		
rs6504950	Duration of estrogen only MHT among current users (years)	>0-<5	26/ 303	1.47 (0.81-2.69)		
rs6504950	Duration of estrogen only MHT among current users (years)	5-<10	29/ 288	0.82 (0.43-1.56)		
rs6504950	Duration of estrogen only MHT among current users (years)	>=10	30/ 437	0.73 (0.39-1.37)		
rs6504950	Duration of estrogen only MHT among current users (years/5)	combined	1074/ 9092		0.87	1.5E-01
rs6504950	Mean lifetime intake of alcohol (g/day)	0	605/ 2945	1.09 (0.95-1.25)		
rs6504950	Mean lifetime intake of alcohol (g/day)	>0-<10	684/ 4928	1.06 (0.93-1.20)		
rs6504950	Mean lifetime intake of alcohol (g/day)	>=10-<20	127/ 1129	0.93 (0.69-1.25)		
rs6504950	Mean lifetime intake of alcohol (g/day)	>=20	105/ 794	0.91 (0.65-1.27)		
rs6504950	Mean lifetime intake of alcohol (10g/day)	combined	1521/ 9796		0.96	3.1E-01
rs6504950	Smoking (ever)	no	1747/10012	1.05 (0.96-1.14)		
rs6504950	Smoking (ever)	yes	1465/ 8375	1.01 (0.92-1.11)		
rs6504950	Smoking (ever/never)	combined	3212/18387		0.97	5.9E-01
rs6504950	Smoking (pack-years)	0	1621/ 8830	1.05 (0.96-1.14)		
rs6504950	Smoking (pack-years)	0-<10	609/ 3548	1.03 (0.89-1.19)		
rs6504950	Smoking (pack-years)	10-<20	306/ 1599	0.98 (0.80-1.21)		
rs6504950	Smoking (pack-years)	>=20	319/ 2095	1.10 (0.91-1.33)		
rs6504950	Smoking (pack-years/10)	combined	2855/16072		1.01	7.7E-01

SNP	Variable	Stratum	N (cases/ controls)	OR (95% CI)[1]	OR int[2]	P int[3]
rs6504950	Physical activity during year before reference date (h/week)	0	112/ 1064	0.88 (0.63-1.22)		
rs6504950	Physical activity during year before reference date (h/week)	0-<3.5	330/ 2818	0.97 (0.80-1.16)		
rs6504950	Physical activity during year before reference date (h/week)	3.5-<7	242/ 2223	1.01 (0.81-1.24)		
rs6504950	Physical activity during year before reference date (h/week)	>=7	345/ 3681	1.06 (0.89-1.26)		
rs6504950	Physical activity during year before reference date (square root of h/week)	combined	1029/ 9786		1.01	7.4E-01
rs704010	Age at menarche (years)	<=11	436/ 2918	0.97 (0.83-1.13)		
rs704010	Age at menarche (years)	12-13	1556/10806	0.96 (0.88-1.04)		
rs704010	Age at menarche (years)	>=14	1397/10866	1.06 (0.97-1.15)		
rs704010	Age at menarche (years/2)	combined	3389/24590		1.05	1.9E-01
rs704010	Parous	no	616/ 3814	0.98 (0.86-1.12)		
rs704010	Parous	yes	3411/24165	1.01 (0.96-1.07)		
rs704010	Parous (yes/no)	combined	4027/27979		1.02	7.4E-01
rs704010	Number of births (among parous)	1	875/ 5140	0.98 (0.88-1.09)		
rs704010	Number of births (among parous)	2	1499/11018	1.02 (0.94-1.10)		
rs704010	Number of births (among parous)	3	632/ 4815	1.04 (0.92-1.18)		
rs704010	Number of births (among parous)	>=4	332/ 2319	0.97 (0.82-1.16)		
rs704010	Number of births (among parous)	combined	3338/23292		1.01	6.8E-01
rs704010	Age at first birth (among parous, years)	<20	362/ 2477	0.99 (0.84-1.17)		
rs704010	Age at first birth (among parous, years)	20-24	1243/ 9293	1.04 (0.95-1.14)		
rs704010	Age at first birth (among parous, years)	25-29	905/ 6910	1.02 (0.92-1.14)		
rs704010	Age at first birth (among parous, years)	>=30	440/ 2997	0.80 (0.68-0.93)		
rs704010	Age at first birth (among parous, years/5)	combined	2950/21677		0.94	5.4E-02
rs704010	Ever breastfed (among parous, yes/no)	no	500/ 2296	1.14 (0.99-1.32)		
rs704010	Ever breastfed (among parous, yes/no)	yes	1612/ 9410	0.95 (0.87-1.03)		
rs704010	Ever breastfed (among parous, yes/no)	combined	2112/11706		0.83	2.6E-02
rs704010	Usual adult BMI, age<54	<25	843/ 2719	1.00 (0.89-1.12)		
rs704010	Usual adult BMI, age<54	25-<30	259/ 879	1.02 (0.83-1.26)		
rs704010	Usual adult BMI, age<54	>=30	113/ 413	1.10 (0.81-1.49)		
rs704010	Usual adult BMI (BMI/5), age<54	combined	1215/ 4011		1.07	2.2E-01
rs704010	Usual adult BMI, age>=54	<25	730/ 5940	1.06 (0.94-1.18)		
rs704010	Usual adult BMI, age>=54	25-<30	341/ 2486	0.92 (0.77-1.09)		
rs704010	Usual adult BMI, age>=54	>=30	114/ 806	0.71 (0.52-0.97)		
rs704010	Usual adult BMI (BMI/5), age>=54	combined	1185/ 9232		0.87	1.2E-02
rs704010	Usual adult height (cm)	<160	811/ 4892	0.92 (0.82-1.03)		
rs704010	Usual adult height (cm)	160-<165	986/ 5891	1.02 (0.92-1.13)		
rs704010	Usual adult height (cm)	165-<170	872/ 4848	0.98 (0.88-1.10)		
rs704010	Usual adult height (cm)	>=170	610/ 3199	1.02 (0.90-1.16)		
rs704010	Usual adult height (cm/5)	combined	3279/18830		1.03	2.4E-01
rs704010	Ever use of oral contraceptives	no	1151/ 7376	0.96 (0.88-1.06)		
rs704010	Ever use of oral contraceptives	yes	1587/10185	1.00 (0.92-1.08)		
rs704010	Ever use of oral contraceptives (yes/no)	combined	2738/17561		1.03	6.0E-01
rs704010	Duration of oral contraceptive use (years)	0	1151/ 7376	0.96 (0.88-1.06)		
rs704010	Duration of oral contraceptive use (years)	>0-<5	487/ 3383	1.00 (0.87-1.16)		
rs704010	Duration of oral contraceptive use (years)	5-<10	405/ 2477	0.94 (0.80-1.10)		
rs704010	Duration of oral contraceptive use (years)	>=10	666/ 4043	1.05 (0.93-1.18)		
rs704010	Duration of oral contraceptive use (years/5)	combined	2709/17279		1.02	2.7E-01
rs704010	Current use of combined estrogen/ progestagen MHT	never	546/ 4067	1.05 (0.93-1.20)		
rs704010	Current use of combined estrogen/ progestagen MHT	EPCurrent	141/ 1187	1.16 (0.89-1.50)		
rs704010	Current use of combined estrogen/ progestagen MHT (yes/no)	combined	989/ 8109		1.17	2.7E-01

SNP	Variable	Stratum	N (cases/ controls)	OR (95% CI)[1]	OR int[2]	P int[3]
rs704010	Current use of estrogen only MHT	never	570/ 4138	1.04 (0.91-1.18)		
rs704010	Current use of estrogen only MHT	ECurrent	85/ 916	0.89 (0.64-1.24)		
rs704010	Current use of estrogen only MHT (yes/no)	combined	1032/ 8272		0.87	4.4E-01
rs704010	Duration of combined estrogen/progestagen MHT among current users (years)	never	546/ 4067	1.05 (0.93-1.20)		
rs704010	Duration of combined estrogen/progestagen MHT among current users (years)	>0-<5	36/ 313	1.32 (0.80-2.17)		
rs704010	Duration of combined estrogen/progestagen MHT among current users (years)	5-<10	48/ 353	1.09 (0.69-1.73)		
rs704010	Duration of combined estrogen/progestagen MHT among current users (years)	>=10	49/ 463	1.00 (0.64-1.56)		
rs704010	Duration of combined estrogen/progestagen MHT among current users (years/5)	combined	980/ 8014		1.00	9.7E-01
rs704010	Duration of estrogen only MHT among current users (years)	never	570/ 4138	1.04 (0.91-1.18)		
rs704010	Duration of estrogen only MHT among current users (years)	>0-<5	27/ 279	0.74 (0.40-1.37)		
rs704010	Duration of estrogen only MHT among current users (years)	5-<10	30/ 255	0.84 (0.47-1.51)		
rs704010	Duration of estrogen only MHT among current users (years)	>=10	27/ 359	1.02 (0.58-1.80)		
rs704010	Duration of estrogen only MHT among current users (years/5)	combined	1028/ 8197		0.97	7.4E-01
rs704010	Mean lifetime intake of alcohol (g/day)	0	554/ 2802	1.04 (0.91-1.19)		
rs704010	Mean lifetime intake of alcohol (g/day)	>0-<10	636/ 4653	0.98 (0.86-1.10)		
rs704010	Mean lifetime intake of alcohol (g/day)	>=10-<20	114/ 1060	1.01 (0.76-1.33)		
rs704010	Mean lifetime intake of alcohol (g/day)	>=20	102/ 741	0.89 (0.65-1.23)		
rs704010	Mean lifetime intake of alcohol (10g/day)	combined	1406/ 9256		0.95	1.5E-01
rs704010	Smoking (ever)	no	1701/ 9642	1.05 (0.97-1.13)		
rs704010	Smoking (ever)	yes	1442/ 7785	0.92 (0.85-1.01)		
rs704010	Smoking (ever/never)	combined	3143/17427		0.88	3.7E-02
rs704010	Smoking (pack-years)	0	1576/ 8468	1.06 (0.98-1.15)		
rs704010	Smoking (pack-years)	0-<10	597/ 3371	0.98 (0.86-1.12)		
rs704010	Smoking (pack-years)	10-<20	303/ 1474	0.84 (0.70-1.02)		
rs704010	Smoking (pack-years)	>=20	300/ 1846	0.96 (0.80-1.16)		
rs704010	Smoking (pack-years/10)	combined	2776/15159		0.94	3.5E-02
rs704010	Physical activity during year before reference date (h/week)	0	111/ 678	0.84 (0.62-1.13)		
rs704010	Physical activity during year before reference date (h/week)	0-<3.5	331/ 2413	0.91 (0.77-1.08)		
rs704010	Physical activity during year before reference date (h/week)	3.5-<7	239/ 1966	1.06 (0.87-1.29)		
rs704010	Physical activity during year before reference date (h/week)	>=7	322/ 3342	1.06 (0.90-1.26)		
rs704010	Physical activity during year before reference date (square root of h/week)	combined	1003/ 8399		1.06	1.6E-01
rs865686	Age at menarche (years)	<=11	585/ 3572	0.90 (0.78-1.03)		
rs865686	Age at menarche (years)	12-13	1741/11574	1.01 (0.93-1.09)		
rs865686	Age at menarche (years)	>=14	1401/11081	0.99 (0.91-1.08)		
rs865686	Age at menarche (years/2)	combined	3727/26227		1.01	6.7E-01
rs865686	Parous	no	657/ 4121	1.06 (0.93-1.20)		
rs865686	Parous	yes	3736/25672	0.98 (0.93-1.04)		
rs865686	Parous (yes/no)	combined	4393/29793		0.93	3.0E-01
rs865686	Number of births (among parous)	1	829/ 5006	1.04 (0.93-1.16)		
rs865686	Number of births (among parous)	2	1675/11760	0.94 (0.87-1.01)		
rs865686	Number of births (among parous)	3	766/ 5381	1.10 (0.98-1.24)		
rs865686	Number of births (among parous)	>=4	402/ 2657	0.91 (0.78-1.07)		
rs865686	Number of births (among parous)	combined	3672/24804		0.99	7.3E-01
rs865686	Age at first birth (among parous, years)	<20	450/ 2406	1.00 (0.85-1.16)		
rs865686	Age at first birth (among parous, years)	20-24	1335/ 9712	0.95 (0.87-1.04)		
rs865686	Age at first birth (among parous, years)	25-29	984/ 7727	0.97 (0.88-1.07)		

SNP	Variable	Stratum	N (cases/ controls)	OR (95% CI)[1]	OR int[2]	P int[3]
rs865686	Age at first birth (among parous, years)	>=30	477/ 3325	1.06 (0.91-1.23)		
rs865686	Age at first birth (among parous, years/5)	combined	3246/23170		1.02	5.8E-01
rs865686	Ever breastfed (among parous, yes/no)	no	377/ 1895	0.92 (0.78-1.10)		
rs865686	Ever breastfed (among parous, yes/no)	yes	1227/ 7795	0.96 (0.88-1.06)		
rs865686	Ever breastfed (among parous, yes/no)	combined	1604/ 9690		1.04	6.7E-01
rs865686	Usual adult BMI, age<54	<25	597/ 1921	0.91 (0.79-1.06)		
rs865686	Usual adult BMI, age<54	25-<30	206/ 728	1.00 (0.79-1.28)		
rs865686	Usual adult BMI, age<54	>=30	104/ 400	0.83 (0.59-1.16)		
rs865686	Usual adult BMI (BMI/5), age<54	combined	907/ 3049		0.97	6.5E-01
rs865686	Usual adult BMI, age>=54	<25	552/ 4973	0.99 (0.87-1.13)		
rs865686	Usual adult BMI, age>=54	25-<30	261/ 2174	1.01 (0.84-1.23)		
rs865686	Usual adult BMI, age>=54	>=30	102/ 766	0.98 (0.71-1.35)		
rs865686	Usual adult BMI (BMI/5), age>=54	combined	915/ 7913		0.99	9.1E-01
rs865686	Usual adult height (cm)	<160	625/ 4115	0.86 (0.75-0.98)		
rs865686	Usual adult height (cm)	160-<165	774/ 5003	1.01 (0.90-1.13)		
rs865686	Usual adult height (cm)	165-<170	752/ 4409	1.03 (0.91-1.16)		
rs865686	Usual adult height (cm)	>=170	549/ 2983	0.93 (0.81-1.07)		
rs865686	Usual adult height (cm/5)	combined	2700/16510		1.02	4.9E-01
rs865686	Ever use of oral contraceptives	no	677/ 5440	0.92 (0.81-1.03)		
rs865686	Ever use of oral contraceptives	yes	1482/ 9780	0.97 (0.89-1.06)		
rs865686	Ever use of oral contraceptives (yes/no)	combined	2159/15220		1.06	4.4E-01
rs865686	Duration of oral contraceptive use (years)	0	677/ 5440	0.92 (0.81-1.03)		
rs865686	Duration of oral contraceptive use (years)	>0-<5	436/ 3184	0.93 (0.80-1.08)		
rs865686	Duration of oral contraceptive use (years)	5-<10	379/ 2409	1.01 (0.86-1.19)		
rs865686	Duration of oral contraceptive use (years)	>=10	643/ 3926	1.00 (0.88-1.13)		
rs865686	Duration of oral contraceptive use (years/5)	combined	2135/14959		1.01	5.9E-01
rs865686	Current use of combined estrogen/ progestagen MHT	never	343/ 3082	1.07 (0.91-1.25)		
rs865686	Current use of combined estrogen/ progestagen MHT	EPCurrent	131/ 1112	0.99 (0.75-1.29)		
rs865686	Current use of combined estrogen/ progestagen MHT (yes/no)	combined	734/ 6820		0.99	9.3E-01
rs865686	Current use of estrogen only MHT	never	367/ 3152	1.09 (0.93-1.28)		
rs865686	Current use of estrogen only MHT	ECurrent	80/ 875	0.78 (0.54-1.12)		
rs865686	Current use of estrogen only MHT (yes/no)	combined	776/ 6985		0.76	1.6E-01
rs865686	Duration of combined estrogen/progestagen MHT among current users (years)	never	343/ 3082	1.07 (0.91-1.25)		
rs865686	Duration of combined estrogen/progestagen MHT among current users (years)	>0-<5	29/ 277	1.02 (0.59-1.76)		
rs865686	Duration of combined estrogen/progestagen MHT among current users (years)	5-<10	42/ 330	1.24 (0.78-1.99)		
rs865686	Duration of combined estrogen/progestagen MHT among current users (years)	>=10	52/ 450	0.77 (0.49-1.21)		
rs865686	Duration of combined estrogen/progestagen MHT among current users (years/5)	combined	725/ 6728		0.97	6.0E-01
rs865686	Duration of estrogen only MHT among current users (years)	never	367/ 3152	1.09 (0.93-1.27)		
rs865686	Duration of estrogen only MHT among current users (years)	>0-<5	24/ 263	0.78 (0.41-1.49)		
rs865686	Duration of estrogen only MHT among current users (years)	5-<10	28/ 240	1.04 (0.57-1.90)		
rs865686	Duration of estrogen only MHT among current users (years)	>=10	27/ 351	0.60 (0.31-1.16)		
rs865686	Duration of estrogen only MHT among current users (years/5)	combined	772/ 6914		0.86	1.0E-01
rs865686	Mean lifetime intake of alcohol (g/day)	0	197/ 1380	0.87 (0.70-1.09)		
rs865686	Mean lifetime intake of alcohol (g/day)	>0-<10	476/ 3999	1.01 (0.88-1.17)		
rs865686	Mean lifetime intake of alcohol (g/day)	>=10-<20	91/ 964	0.86 (0.62-1.19)		
rs865686	Mean lifetime intake of alcohol (g/day)	>=20	89/ 693	0.93 (0.67-1.28)		
rs865686	Mean lifetime intake of alcohol (10g/day)	combined	853/ 7036		1.04	2.7E-01

SNP	Variable	Stratum	N (cases/ controls)	OR (95% CI)[1]	OR int[2]	P int[3]
rs865686	Smoking (ever)	no	1456/ 8586	0.93 (0.85-1.01)		
rs865686	Smoking (ever)	yes	1110/ 6552	1.00 (0.91-1.10)		
rs865686	Smoking (ever/never)	combined	2566/15138		1.08	2.5E-01
rs865686	Smoking (pack-years)	0	1326/ 7418	0.92 (0.84-1.00)		
rs865686	Smoking (pack-years)	0-<10	411/ 2671	1.04 (0.89-1.22)		
rs865686	Smoking (pack-years)	10-<20	222/ 1199	0.99 (0.80-1.24)		
rs865686	Smoking (pack-years)	>=20	243/ 1600	0.97 (0.79-1.20)		
rs865686	Smoking (pack-years/10)	combined	2202/12888		1.03	3.2E-01
rs865686	Physical activity during year before reference date (h/week)	0	110/ 671	0.84 (0.62-1.14)		
rs865686	Physical activity during year before reference date (h/week)	0-<3.5	322/ 2375	0.81 (0.68-0.97)		
rs865686	Physical activity during year before reference date (h/week)	3.5-<7	233/ 1942	1.07 (0.88-1.30)		
rs865686	Physical activity during year before reference date (h/week)	>=7	328/ 3241	1.11 (0.94-1.30)		
rs865686	Physical activity during year before reference date (square root of h/week)	combined	993/ 8229		1.06	1.6E-01
rs889312	Age at menarche (years)	<=11	620/ 3407	1.01 (0.88-1.16)		
rs889312	Age at menarche (years)	12-13	1881/10500	1.03 (0.95-1.11)		
rs889312	Age at menarche (years)	>=14	1545/ 9970	1.14 (1.04-1.24)		
rs889312	Age at menarche (years/2)	combined	4046/23877		1.04	2.0E-01
rs889312	Parous	no	680/ 3512	1.10 (0.96-1.26)		
rs889312	Parous	yes	3978/23500	1.05 (1.00-1.11)		
rs889312	Parous (yes/no)	combined	4658/27012		0.96	5.4E-01
rs889312	Number of births (among parous)	1	940/ 4631	1.01 (0.90-1.13)		
rs889312	Number of births (among parous)	2	1804/10499	1.08 (1.00-1.17)		
rs889312	Number of births (among parous)	3	783/ 4998	1.06 (0.94-1.20)		
rs889312	Number of births (among parous)	>=4	411/ 2723	1.01 (0.86-1.19)		
rs889312	Number of births (among parous)	combined	3938/22851		1.00	9.7E-01
rs889312	Age at first birth (among parous, years)	<20	487/ 2280	1.12 (0.96-1.31)		
rs889312	Age at first birth (among parous, years)	20-24	1509/ 9480	1.07 (0.98-1.16)		
rs889312	Age at first birth (among parous, years)	25-29	1043/ 6690	1.06 (0.96-1.18)		
rs889312	Age at first birth (among parous, years)	>=30	480/ 2783	1.12 (0.96-1.31)		
rs889312	Age at first birth (among parous, years/5)	combined	3519/21233		1.01	8.4E-01
rs889312	Ever breastfed (among parous, yes/no)	no	458/ 2473	1.05 (0.89-1.23)		
rs889312	Ever breastfed (among parous, yes/no)	yes	1525/ 8517	1.12 (1.03-1.22)		
rs889312	Ever breastfed (among parous, yes/no)	combined	1983/10990		1.07	4.5E-01
rs889312	Usual adult BMI, age<54	<25	792/ 2771	1.07 (0.94-1.21)		
rs889312	Usual adult BMI, age<54	25-<30	249/ 941	1.11 (0.87-1.40)		
rs889312	Usual adult BMI, age<54	>=30	91/ 454	1.57 (1.09-2.28)		
rs889312	Usual adult BMI (BMI/5), age<54	combined	1132/ 4166		1.06	3.4E-01
rs889312	Usual adult BMI, age>=54	<25	649/ 4812	1.16 (1.02-1.33)		
rs889312	Usual adult BMI, age>=54	25-<30	310/ 2300	1.05 (0.87-1.27)		
rs889312	Usual adult BMI, age>=54	>=30	106/ 810	0.95 (0.68-1.33)		
rs889312	Usual adult BMI (BMI/5), age>=54	combined	1065/ 7922		0.96	4.7E-01
rs889312	Usual adult height (cm)	<160	779/ 4818	1.02 (0.90-1.15)		
rs889312	Usual adult height (cm)	160-<165	956/ 5487	0.97 (0.87-1.09)		
rs889312	Usual adult height (cm)	165-<170	795/ 4378	1.31 (1.16-1.47)		
rs889312	Usual adult height (cm)	>=170	551/ 2803	1.04 (0.89-1.20)		
rs889312	Usual adult height (cm/5)	combined	3081/17486		1.03	2.2E-01
rs889312	Ever use of oral contraceptives	no	1187/ 7370	1.11 (1.00-1.22)		
rs889312	Ever use of oral contraceptives	yes	1404/ 8656	1.06 (0.96-1.16)		
rs889312	Ever use of oral contraceptives (yes/no)	combined	2591/16026		0.95	5.0E-01

SNP	Variable	Stratum	N (cases/ controls)	OR (95% CI)[1]	OR int[2]	P int[3]
rs889312	Duration of oral contraceptive use (years)	0	1187/ 7370	1.11 (1.00-1.22)		
rs889312	Duration of oral contraceptive use (years)	>0-<5	451/ 2994	1.07 (0.92-1.26)		
rs889312	Duration of oral contraceptive use (years)	5-<10	350/ 2124	1.08 (0.90-1.30)		
rs889312	Duration of oral contraceptive use (years)	>=10	572/ 3271	1.03 (0.89-1.18)		
rs889312	Duration of oral contraceptive use (years/5)	combined	2560/15759		0.99	5.9E-01
rs889312	Current use of combined estrogen/ progestagen MHT	never	510/ 3776	1.07 (0.92-1.23)		
rs889312	Current use of combined estrogen/ progestagen MHT	EPCurrent	120/ 923	1.26 (0.93-1.69)		
rs889312	Current use of combined estrogen/ progestagen MHT (yes/no)	combined	870/ 6950		1.16	3.7E-01
rs889312	Current use of estrogen only MHT	never	534/ 3853	1.09 (0.94-1.25)		
rs889312	Current use of estrogen only MHT	ECurrent	72/ 802	1.44 (0.99-2.10)		
rs889312	Current use of estrogen only MHT (yes/no)	combined	914/ 7117		1.32	1.7E-01
rs889312	Duration of combined estrogen/progestagen MHT among current users (years)	never	510/ 3776	1.07 (0.92-1.23)		
rs889312	Duration of combined estrogen/progestagen MHT among current users (years)	>0-<5	32/ 292	1.13 (0.63-2.01)		
rs889312	Duration of combined estrogen/progestagen MHT among current users (years)	5-<10	45/ 280	1.04 (0.61-1.75)		
rs889312	Duration of combined estrogen/progestagen MHT among current users (years)	>=10	35/ 300	1.75 (1.04-2.96)		
rs889312	Duration of combined estrogen/progestagen MHT among current users (years/5)	combined	860/ 6865		1.15	6.9E-02
rs889312	Duration of estrogen only MHT among current users (years)	never	534/ 3853	1.09 (0.94-1.25)		
rs889312	Duration of estrogen only MHT among current users (years)	>0-<5	24/ 235	1.28 (0.67-2.48)		
rs889312	Duration of estrogen only MHT among current users (years)	5-<10	26/ 213	1.87 (0.97-3.60)		
rs889312	Duration of estrogen only MHT among current users (years)	>=10	21/ 318	1.59 (0.79-3.20)		
rs889312	Duration of estrogen only MHT among current users (years/5)	combined	910/ 7010		1.13	1.9E-01
rs889312	Mean lifetime intake of alcohol (g/day)	0	610/ 2807	1.00 (0.87-1.16)		
rs889312	Mean lifetime intake of alcohol (g/day)	>0-<10	593/ 3719	1.18 (1.03-1.34)		
rs889312	Mean lifetime intake of alcohol (g/day)	>=10-<20	116/ 775	1.11 (0.82-1.49)		
rs889312	Mean lifetime intake of alcohol (g/day)	>=20	95/ 544	1.11 (0.79-1.56)		
rs889312	Mean lifetime intake of alcohol (10g/day)	combined	1414/ 7845		1.05	2.3E-01
rs889312	Smoking (ever)	no	1565/ 8828	1.09 (1.00-1.18)		
rs889312	Smoking (ever)	yes	1327/ 7115	1.08 (0.98-1.19)		
rs889312	Smoking (ever/never)	combined	2892/15943		0.99	9.3E-01
rs889312	Smoking (pack-years)	0	1435/ 7637	1.11 (1.01-1.21)		
rs889312	Smoking (pack-years)	0-<10	569/ 3024	1.10 (0.95-1.27)		
rs889312	Smoking (pack-years)	10-<20	274/ 1308	1.34 (1.08-1.65)		
rs889312	Smoking (pack-years)	>=20	275/ 1692	0.92 (0.74-1.14)		
rs889312	Smoking (pack-years/10)	combined	2553/13661		0.97	4.1E-01
rs889312	Physical activity during year before reference date (h/week)	0	110/ 1059	1.11 (0.81-1.52)		
rs889312	Physical activity during year before reference date (h/week)	0-<3.5	270/ 2480	1.01 (0.83-1.24)		
rs889312	Physical activity during year before reference date (h/week)	3.5-<7	182/ 1708	1.16 (0.91-1.47)		
rs889312	Physical activity during year before reference date (h/week)	>=7	227/ 2104	1.24 (1.01-1.53)		
rs889312	Physical activity during year before reference date (square root of h/week)	combined	789/ 7351		1.03	5.0E-01
rs999737[8]	Age at menarche (years)	<=11	661/ 3486	0.97 (0.84-1.12)		
rs999737	Age at menarche (years)	12-13	1985/11398	1.03 (0.95-1.12)		
rs999737	Age at menarche (years)	>=14	1587/11067	0.92 (0.84-1.01)		
rs999737	Age at menarche (years/2)	combined	4233/25951		0.96	2.3E-01
rs999737	Parous	no	723/ 3775	0.83 (0.72-0.95)		
rs999737	Parous	yes	4156/25161	0.98 (0.92-1.03)		
rs999737	Parous (yes/no)	combined	4879/28936		1.18	3.5E-02

SNP	Variable	Stratum	N (cases/ controls)	OR (95% CI)[1]	OR int[2]	P int[3]
rs999737	Number of births (among parous)	1	974/ 4849	0.99 (0.88-1.12)		
rs999737	Number of births (among parous)	2	1876/11402	0.99 (0.91-1.07)		
rs999737	Number of births (among parous)	3	785/ 5135	0.90 (0.79-1.02)		
rs999737	Number of births (among parous)	>=4	388/ 2573	1.05 (0.88-1.26)		
rs999737	Number of births (among parous)	combined	4023/23959		1.00	9.2E-01
rs999737	Age at first birth (among parous, years)	<20	493/ 2385	0.94 (0.79-1.12)		
rs999737	Age at first birth (among parous, years)	20-24	1540/ 9682	0.96 (0.87-1.05)		
rs999737	Age at first birth (among parous, years)	25-29	1089/ 7344	1.06 (0.95-1.18)		
rs999737	Age at first birth (among parous, years)	>=30	529/ 3243	1.03 (0.88-1.21)		
rs999737	Age at first birth (among parous, years/5)	combined	3651/22654		1.00	1.0E+00
rs999737	Ever breastfed (among parous, yes/no)	no	483/ 2253	0.94 (0.79-1.13)		
rs999737	Ever breastfed (among parous, yes/no)	yes	1596/ 7767	1.01 (0.93-1.11)		
rs999737	Ever breastfed (among parous, yes/no)	combined	2079/10020		1.08	4.7E-01
rs999737	Usual adult BMI, age<54	<25	884/ 2862	0.96 (0.84-1.09)		
rs999737	Usual adult BMI, age<54	25-<30	275/ 989	0.89 (0.70-1.13)		
rs999737	Usual adult BMI, age<54	>=30	116/ 497	1.06 (0.75-1.50)		
rs999737	Usual adult BMI (BMI/5), age<54	combined	1275/ 4348		0.99	9.2E-01
rs999737	Usual adult BMI, age>=54	<25	643/ 3954	0.98 (0.85-1.13)		
rs999737	Usual adult BMI, age>=54	25-<30	303/ 1988	1.23 (1.01-1.51)		
rs999737	Usual adult BMI, age>=54	>=30	110/ 822	0.61 (0.41-0.89)		
rs999737	Usual adult BMI (BMI/5), age>=54	combined	1056/ 6764		0.93	2.8E-01
rs999737	Usual adult height (cm)	<160	809/ 4444	0.90 (0.79-1.03)		
rs999737	Usual adult height (cm)	160-<165	927/ 5155	0.99 (0.88-1.12)		
rs999737	Usual adult height (cm)	165-<170	819/ 4091	1.06 (0.93-1.20)		
rs999737	Usual adult height (cm)	>=170	579/ 2706	1.00 (0.86-1.17)		
rs999737	Usual adult height (cm/5)	combined	3134/16396		1.04	1.7E-01
rs999737	Ever use of oral contraceptives	no	1134/ 6663	0.99 (0.89-1.10)		
rs999737	Ever use of oral contraceptives	yes	1510/ 8157	1.00 (0.91-1.10)		
rs999737	Ever use of oral contraceptives (yes/no)	combined	2644/14820		1.01	8.6E-01
rs999737	Duration of oral contraceptive use (years)	0	1134/ 6663	0.99 (0.89-1.10)		
rs999737	Duration of oral contraceptive use (years)	>0-<5	465/ 2778	1.04 (0.88-1.23)		
rs999737	Duration of oral contraceptive use (years)	5-<10	380/ 2016	1.22 (1.01-1.46)		
rs999737	Duration of oral contraceptive use (years)	>=10	639/ 3112	0.87 (0.75-1.01)		
rs999737	Duration of oral contraceptive use (years/5)	combined	2618/14569		0.98	4.3E-01
rs999737	Current use of combined estrogen/ progestagen MHT	never	492/ 3115	0.96 (0.81-1.13)		
rs999737	Current use of combined estrogen/ progestagen MHT	EPCurrent	114/ 755	1.44 (1.06-1.97)		
rs999737	Current use of combined estrogen/ progestagen MHT (yes/no)	combined	853/ 5600		1.43	3.6E-02
rs999737	Current use of estrogen only MHT	never	516/ 3196	0.94 (0.80-1.11)		
rs999737	Current use of estrogen only MHT	ECurrent	71/ 640	1.38 (0.92-2.06)		
rs999737	Current use of estrogen only MHT (yes/no)	combined	898/ 5779		1.33	1.9E-01
rs999737	Duration of combined estrogen/progestagen MHT among current users (years)	never	492/ 3115	0.96 (0.81-1.13)		
rs999737	Duration of combined estrogen/progestagen MHT among current users (years)	>0-<5	29/ 234	1.09 (0.56-2.11)		
rs999737	Duration of combined estrogen/progestagen MHT among current users (years)	5-<10	43/ 229	1.24 (0.76-2.03)		
rs999737	Duration of combined estrogen/progestagen MHT among current users (years)	>=10	34/ 241	1.82 (1.03-3.20)		
rs999737	Duration of combined estrogen/progestagen MHT among current users (years/5)	combined	843/ 5516		1.21	2.2E-02
rs999737	Duration of estrogen only MHT among current users (years)	never	516/ 3196	0.94 (0.80-1.10)		
rs999737	Duration of estrogen only MHT among current users (years)	>0-<5	21/ 175	0.89 (0.41-1.95)		

SNP	Variable	Stratum	N (cases/ controls)	OR (95% CI)[1]	OR int[2]	P int[3]
rs999737	Duration of estrogen only MHT among current users (years)	5-<10	21/ 173	1.06 (0.49-2.29)		
rs999737	Duration of estrogen only MHT among current users (years)	>=10	28/ 258	2.12 (1.11-4.05)		
rs999737	Duration of estrogen only MHT among current users (years/5)	combined	893/ 5667		1.25	2.3E-02
rs999737	Mean lifetime intake of alcohol (g/day)	0	571/ 2533	1.10 (0.95-1.28)		
rs999737	Mean lifetime intake of alcohol (g/day)	>0-<10	586/ 2864	0.99 (0.86-1.15)		
rs999737	Mean lifetime intake of alcohol (g/day)	>=10-<20	115/ 590	0.75 (0.53-1.05)		
rs999737	Mean lifetime intake of alcohol (g/day)	>=20	95/ 394	1.10 (0.75-1.61)		
rs999737	Mean lifetime intake of alcohol (10g/day)	combined	1367/ 6381		0.95	3.0E-01
rs999737	Smoking (ever)	no	1603/ 8263	1.00 (0.91-1.10)		
rs999737	Smoking (ever)	yes	1388/ 6774	0.99 (0.90-1.10)		
rs999737	Smoking (ever/never)	combined	2991/15037		0.99	8.6E-01
rs999737	Smoking (pack-years)	0	1498/ 7263	1.02 (0.93-1.12)		
rs999737	Smoking (pack-years)	0-<10	590/ 2881	1.03 (0.88-1.20)		
rs999737	Smoking (pack-years)	10-<20	293/ 1292	0.84 (0.66-1.05)		
rs999737	Smoking (pack-years)	>=20	303/ 1629	0.98 (0.80-1.21)		
rs999737	Smoking (pack-years/10)	combined	2684/13065		0.98	5.6E-01
rs999737	Physical activity during year before reference date (h/week)	0	107/ 1004	0.83 (0.59-1.17)		
rs999737	Physical activity during year before reference date (h/week)	0-<3.5	293/ 2238	1.10 (0.90-1.35)		
rs999737	Physical activity during year before reference date (h/week)	3.5-<7	172/ 1392	1.00 (0.76-1.31)		
rs999737	Physical activity during year before reference date (h/week)	>=7	254/ 1342	1.18 (0.95-1.47)		
rs999737	Physical activity during year before reference date (square root of h/week)	combined	826/ 5976		1.02	7.1E-01

[1] Per-allele SNP effect in each stratum of environmental risk factors using case-control analysis adjusted for study and reference age
[2] OR for GxE interaction from case-control analysis stratified by study and adjusted for reference age
[3] p value for case-control analysis stratified by study and adjusted for reference age
[4] model used never use of MHT (menopausal hormone therapy) as the reference category and adjusted for former use of MHT and current use of other MHT type, as appropriate
[5] mean lifetime alcohol intake derived from duration and amount of alcohol intake in g/day at different age periods
[6] or the highly correlated SNP rs1975930 (r^2= 1 in HapMap CEU)
[7] or the highly correlated SNP rs1045485 (r^2= 1 in HapMap CEU)
[8] or the highly correlated SNP rs10483813 (r^2= 1 in HapMap CEU)
