# Supplementary material for: Evidence of Gene–Environment Interactions between Common Breast Cancer Susceptibility Loci and Established Environmental Risk Factors
Source: PLoS Genet. 2013 Mar 27;9(3):e1003284. doi: 10.1371/journal.pgen.1003284 (PMC3609648; doi:10.1371/journal.pgen.1003284)
Supplement: Table S7 — Gene-environment interactions between SNPs and breast cancer risk factors in Caucasians with interaction p-value<10−4, overall and by ER status, adjusted for additional covariates. (PDF) [file pgen.1003284.s007.pdf]

Table S7. Gene-environment interactions between SNPs and breast cancer risk factors in Caucasians with interaction p-value <10^{-3}, overall and by ER status, adjusted for additional covariates

			All			Estrogen receptor-positive			Estrogen receptor-negative		
**Variable**	**SNP**	**Locus**	**N (cases/controls)**	**OR (95%CI)[1]**	**P-value[2]**	**N (cases)**	**OR (95%CI)[1]**	**P-value[2]**	**N (cases)**	**OR (95%CI)[1]**	**P-value[2]**
Number of births (among parous)	rs3817198	LSP1	4392/6018	1.07 (1.01-1.14)	0.017	2935	1.10 (1.03-1.17)	0.006	1137	1.03 (0.93-1.03)	0.609
Parous (yes/no)	rs11249433	1p11.2	5413/5603	1.20 (1.03-1.40)	0.020	3590	1.28 (1.08-1.52)	0.005	1350	1.13 (0.88-1.45)	0.328
Mean lifetime intake of alcohol[3] (<20/ >=20g/day)	rs17468277[4]	*CASP8*	5188/ 8145	1.59 (1.21-2.09)	0.001	3738	1.55 (1.16-2.08)	0.003	1225	1.46 (0.86-2.47)	0.164

[1] Odds ratio (95% confidence interval) for GxE interaction from case-control analysis stratified by study, adjusted for reference age and additionally (if not variable of interest) for being parous (yes/no), number of births, BMI, age surrogate for postmenopausal status (≥54 years), interaction of BMI and postmenopausal status (≥54 years), current use of menopausal hormone therapy (MHT), past use of MHT, duration of oral contraceptives use, lifetime alcohol intake, smoking (pack-years). Subjects with missing covariable information were excluded.

[2] p-value for GxE interaction from case-control analysis stratified by study and adjusted for reference age

[3] Mean lifetime daily alcohol intake derived from duration and amount of alcohol intake in g/day at different age periods

[4] or the highly correlated SNP rs1045485 (r^2= 1 in HapMap CEU)
