# Supplementary material for: Evidence of Gene–Environment Interactions between Common Breast Cancer Susceptibility Loci and Established Environmental Risk Factors
Source: PLoS Genet. 2013 Mar 27;9(3):e1003284. doi: 10.1371/journal.pgen.1003284 (PMC3609648; doi:10.1371/journal.pgen.1003284)
Supplement: Table S8 — Gene-environment interactions between SNPs and breast cancer risk factors in Caucasians with interaction p-value<10−4, overall and by ER status, restricted to population-based studies. (PDF) [file pgen.1003284.s008.pdf]

Table S8. Gene-environment interactions between SNPs and breast cancer risk factors in Caucasians with interaction p-value $<10^{-3}$, overall and by ER status, restricted to population-based studies

			All			Estrogen receptor-positive			Estrogen receptor-negative		
**Variable**	**SNP**	**Locus**	**N (cases/controls)**	**OR (95%CI)[1]**	**P-value[2]**	**N (cases)**	**OR (95%CI)[1]**	**P-value[2]**	**N (cases)**	**OR (95%CI)[1]**	**P-value[2]**
Number of births (among parous)	rs3817198	LSP1	11730/14773	1.08 (1.05-1.12)	3.3×10^{-6}	6662	1.08 (1.04-1.13)	3.1×10^{-4}	2259	1.1 (1.03-1.17)	0.005
Parous (yes/no)	rs11249433	*1p11.2*	14741/18447	1.15 (1.05-1.26)	0.003	8983	1.18 (1.06-1.31)	0.003	2905	1.15 (0.97-1.35)	0.107
Mean lifetime intake of alcohol[4] (<20/ >=20g/day)	rs17468277[5]	*CASP8*	6081/ 9305	1.59 (1.24-2.05)	3.1×10^{-4}	4309	1.57 (1.20-2.06)	0.001	1398	1.39 (0.86-2.25)	0.182

[1] Odds ratio (95% confidence interval) for GxE interaction from case-control analysis stratified by study, adjusted for reference age

[2] p-value for GxE interaction from case-control analysis stratified by study and adjusted for reference age

[3] MHT: menopausal hormone therapy, model used never use of MHT as the reference category and adjusted for former use of MHT and current use of other MHT type, as appropriate.

[4] Mean lifetime daily alcohol intake derived from duration and amount of alcohol intake in g/day at different age periods

[5] or the highly correlated SNP rs1045485 (r^2= 1 in HapMap CEU)
