# Supplementary material for: Evidence of Gene–Environment Interactions between Common Breast Cancer Susceptibility Loci and Established Environmental Risk Factors
Source: PLoS Genet. 2013 Mar 27;9(3):e1003284. doi: 10.1371/journal.pgen.1003284 (PMC3609648; doi:10.1371/journal.pgen.1003284)
Supplement: Table S9 — False-positive reporting probability (FPRP) for interactions of SNPs and environmental risk factors of breast cancer showing interaction p-value<10−2. (PDF) [file pgen.1003284.s009.pdf]

Table S9. False-positive report probability (FPRP) for interactions of SNPs with environmental risk factors of breast cancer showing interaction p-value <10^{-2}

SNP (Gene)	Variable	OR (95%CI)[1]	Power[2]	$P_{interaction}$[3]	False-Positive Reporting Probability[4] (Prior Probability) 0.05	0.01	0.001	0.0001
rs3817198 (*LSP1*)	Number of births (among parous women)	1.06 (1.04-1.09)	0.9995	2.4×10^{-6}	<0.001	<0.001	<0.001	0.002
rs11249433 (1p11)	Parous	1.16 (1.08-1.24)	0.9943	5.3×10^{-5}	0.002	0.010	0.093	0.505
rs17468277[5] (*CASP8*)	Mean lifetime intake of alcohol[6] (<20/ >=20 g/day)	1.59 (1.24-2.05)	0.8350	3.1×10^{-4}	0.013	0.064	0.410	0.874
rs13387042 (2q35)	Current use of combined estrogen-progestagen therapy	0.83 (0.73-0.94)	0.8393	2.4×10^{-3}	0.116	0.405	0.873	0.986
rs999737[7] (*RAD51L1*)	Duration of estrogen-only therapy in current users (per 5 years)	1.13 (1.04-1.22)	0.8008	4.0×10^{-3}	0.090	0.340	0.839	0.981
rs2823093 (*NRIP1*)	Current use of estrogen-only therapy	1.35 (1.09-1.67)	0.9999	6.6×10^{-3}	0.191	0.552	0.926	0.992
rs614367 (11q13)	Age at first birth (among parous women, per 5 years)	0.94 (0.90-0.98)	0.9999	9.1×10^{-3}	0.129	0.435	0.886	0.987

[1] Odds ratio (95% confidence interval) for multiplicative GxE interaction from case-control analysis stratified by study and adjusted for reference age

[2] calculated using Quanto 1.2 (type I error rate of 0.05, two-sided), available at http://hydra.usc.edu/gxe

[3] P-value for GxE interaction from case-control analysis stratified by study and adjusted for reference age

[4] calculated according to Wacholder S, Chanock S, Garcia-Closas M, El GL, Rothman N. Assessing the probability that a positive report is false: an approach for molecular epidemiology studies. *J Natl Cancer Inst* 2004;96(6):434-42.

[5] or the highly correlated SNP rs1045485 (r^2= 1 in HapMap CEU)

[6] mean lifetime alcohol intake derived from duration and amount of alcohol intake in g/day at different age periods

[7] or the highly correlated SNP rs10483813 (r^2= 1 in HapMap CEU)
